# Design, Synthesis, and Evaluation of Inhibitors of Hedgehog Acyltransferase

**DOI:** 10.1021/acs.jmedchem.3c01363

**Published:** 2024-01-10

**Authors:** Markus Ritzefeld, Leran Zhang, Zhangping Xiao, Sebastian A. Andrei, Olivia Boyd, Naoko Masumoto, Ursula R. Rodgers, Markus Artelsmair, Lea Sefer, Angela Hayes, Efthymios-Spyridon Gavriil, Florence I. Raynaud, Rosemary Burke, Julian Blagg, Henry S. Rzepa, Christian Siebold, Anthony I. Magee, Thomas Lanyon-Hogg, Edward W. Tate

**Affiliations:** † Department of Chemistry, Imperial College London, London W12 0BZ, U.K.; ‡ National Heart and Lung Institute, Imperial College London, London SW7 2AZ, U.K.; § Division of Structural Biology, University of Oxford, Oxford OX3 7BN, U.K.; ∥ Division of Cancer Therapeutics, Centre for Cancer Drug Discovery, Institute of Cancer Research, London SM2 5NG, U.K.

## Abstract

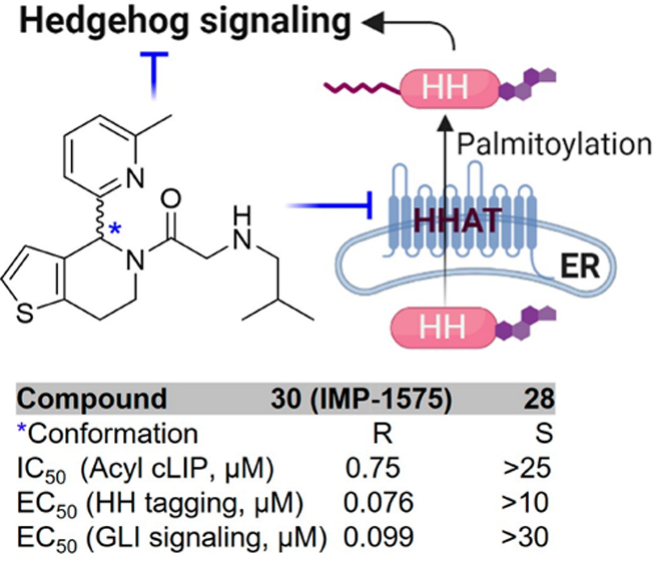

Hedgehog signaling is involved in embryonic development and cancer growth. Functional activity of secreted Hedgehog signaling proteins is dependent on *N*-terminal palmitoylation, making the palmitoyl transferase Hedgehog acyltransferase (HHAT), a potential drug target and a series of 4,5,6,7-tetrahydrothieno[3,2-*c*]pyridines have been identified as HHAT inhibitors. Based on structural data, we designed and synthesized 37 new analogues which we profiled alongside 13 previously reported analogues in enzymatic and cellular assays. Our results show that a central amide linkage, a secondary amine, and (*R*)-configuration at the 4-position of the core are three key factors for inhibitory potency. Several potent analogues with low- or sub-μM IC_50_ against purified HHAT also inhibit Sonic Hedgehog (SHH) palmitoylation in cells and suppress the SHH signaling pathway. This work identifies IMP-1575 as the most potent cell-active chemical probe for HHAT function, alongside an inactive control enantiomer, providing tool compounds for validation of HHAT as a target in cellular assays.

## Introduction

The membrane-bound *O*-acyltransferase (MBOAT) protein superfamily is found in all kingdoms of life and catalyzes transfer of acyl groups from acyl-coenzyme A (acyl-CoA) to a range of substrates. The majority of MBOATs transfer lipids to small molecule substrates such as glycerol; however, three MBOATs in mammals modify signaling peptides or proteins. Ghrelin *O*-acyltransferase (GOAT), Porcupine (PORCN), and Hedgehog acyltransferase (HHAT), acylate Ghrelin, Wnt, and Hedgehog proteins, respectively.^1,2^ These acyltransferase enzymes are potential drug targets in type II diabetes and obesity (GOAT), neurodegeneration (PORCN), and cancer (PORCN and HHAT). Inhibitors of PORCN are under active development for the treatment of neurological disorders and cancers, with inhibitors WNT974 and ETC-159 in early phase clinical trials.^3−5^ Inhibition of GOAT by peptide-coenzyme A product mimetic GO-CoA-Tat, along with triterpenoid-based inhibitors, indicate a potential route to target this MBOAT for therapeutic benefit in type II diabetes and obesity.^6,7^ However, inhibitor development programs targeting HHAT have shown more limited progress to-date.

**Figure 1:**
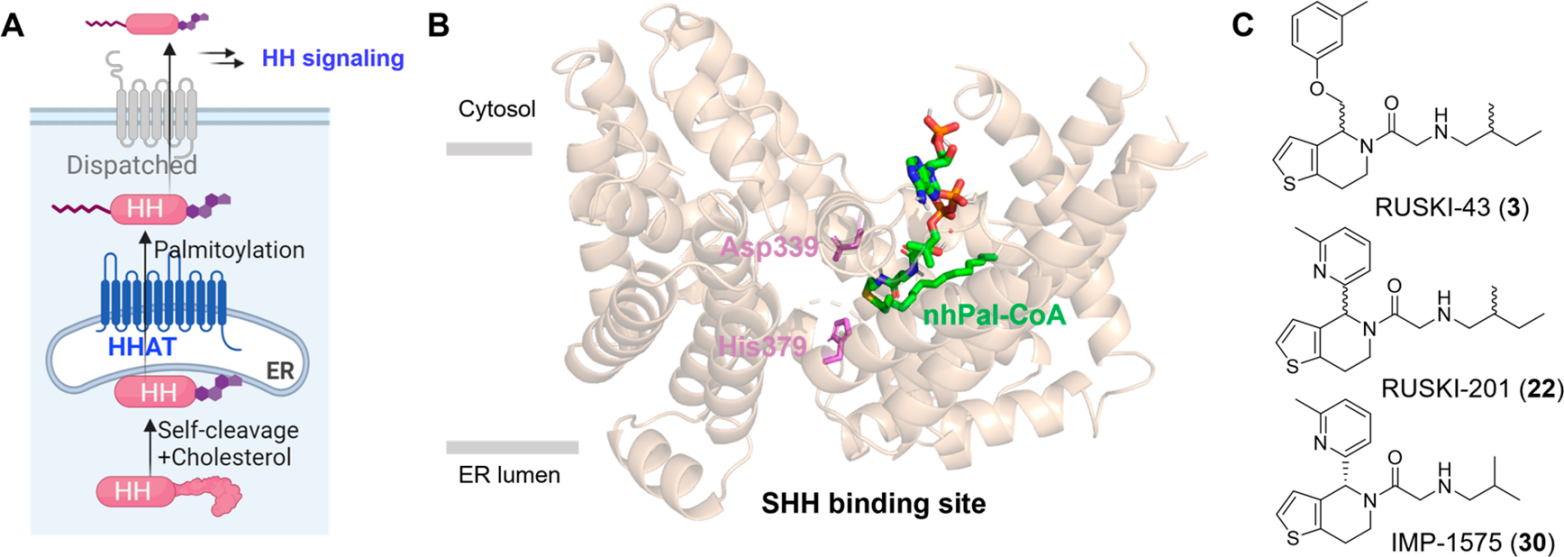
Biological function and structure of HHAT. (A) The maturation process of Hedgehog proteins. HHAT catalyzes *N*-palmitoylation of SHH. (B) Cryo-EM structure of HHAT bound to a nonhydrolyzable Pal-CoA analogue (nhPal-CoA). Asp339 and His379 are located at the center of the substrate binding pocket (PDB: 7Q1U).^14^ (C) Structures of HHAT inhibitors RUSKI-43 (**3**), RUSKI-201 (**22**), and IMP-1575 (**30**).

The Hedgehog (HH) signaling proteins Sonic (SHH), Indian (IHH), and Desert (DHH) Hedgehog, are secreted morphogens that play a critical role in development and disease, with SHH being the most studied homologue. SHH is post-translationally modified through covalent attachment of two lipids which are critical for functional signaling.^8^ Intein-like autocatalytic cleavage of SHH precursor proteins results in the formation of a cholesteryl ester at the C-terminus of the signaling domain;^9,10^ the signaling domain is subsequently *N*-palmitoylated by HHAT (Figure 1A), which is a multipass transmembrane protein that resides in the endoplasmic reticulum (ER) membrane.^11,12^ HHAT specifically recognizes the first 11 amino acids of processed SHH (CGPGRGFGKRR), and is proposed to act as an *S*-acyltransferase at the N-terminal cysteine thiol; the resulting *S*-acyl cysteine thioester is proposed to rearrange through an *S*-to-*N* acyl shift to form a stable *N*-acyl modification at the N-terminal amine.^13^ Recent structural determination of HHAT using cryogenic electron microscopy (cryo-EM) shows that HHAT is composed of 12-transmembrane (TM) helices with both termini located on the cytosolic side of the ER membrane (Figure 1B).^14,15^ These structures reveal that palmitoyl-CoA (Pal-CoA) binds to a continuous solvent cavity through HHAT. The structural changes caused by Pal-CoA binding from the cytosolic side leads to a rearrangement of the active site of HHAT and primes HHAT for SHH binding, positioning the two key residues (His379 and Asp339) in the catalytic core close to the thioester of Pal-CoA (Figure 1B). Subsequently, the SHH *N*-terminus binds to HHAT via a luminal cavity to access the active site for palmitoylation. Dually lipidated SHH is secreted from the signaling cell and binds its receptor Patched (PTCH) at the receiving cell, thereby releasing the inhibition of Smoothened (SMO). Subsequently, SMO accumulates in the primary cilium and activates downstream transcription factors of the GLI family that induce expression of HH-responsive genes.^16−18^

Dysregulation of the HH pathway is connected with a variety of diseases including several types of cancer, chronic cholecystitis, and pulmonary fibrosis.^19,20^ A synergistic combination of the Smoothened (SMO) inhibitor GDC-0449 (vismodegib) and doxorubicin-loaded PEG–PCL copolymer micelles as chemotherapeutic agent has recently been reported as a potential treatment strategy in fibroblast-enriched pancreatic cancer.^21^ In the case of basal cell carcinoma and medulloblastoma, treatment with vismodegib results in clinically useful and rapid tumor regression,^22,23^ although mutations in the target SMO rapidly drive resistance to vismodegib.^24,25^ These findings have led to a growing interest in inhibitors of other targets in the HH pathway as tools to further investigate the impact of inhibition of HH signaling on disease progression.^26−28^ HHAT is a potential drug target due to its key role in the maturation process and HHAT mediated SHH *N*-palmitoylation is essential for HH signaling. This hypothesis is supported by the observation that mutation of the *N*-terminal cysteine of SHH prevents palmitoylation and abolishes induction of neuronal cell differentiation and limb patterning in mice,^29^ and knockdown of HHAT produces antiproliferative effects on cells dependent on HH signaling for growth.^30^

A class of 5-acyl-6,7-dihydrothieno[3,2-*c*]pyridine small molecule HHAT inhibitors were previously identified.^27^ Early studies focused on compound RUSKI-43 (**3**);^31−33^ however, chemical biology profiling demonstrated that RUSKI-43 exhibits significant off-target toxicity independent of HHAT inhibition (Figure 1C).^26,34^ A related analogue RUSKI-201 (**22**) acts on-target within the concentration range required to inhibit HHAT and displays limited off-target toxicity.^35^ Metabolic labeling of cells with an alkyne palmitic acid analogue followed by bioorthogonal “click chemistry” functionalization and isolation of modified proteins for identification by quantitative proteomics demonstrated that treatment with RUSKI-201 only affects palmitoylation of HH proteins, supporting the hypothesis that HH proteins are the only substrates of HHAT and highlighting HHAT as a selective target to block HH signaling.^35^ A preliminary structure–activity relationship (SAR) study on this series identified IMP-1575 (**30**) as the most potent HHAT inhibitor to-date, with an IC_50_ of 0.75 μM for inhibition of purified HHAT. The binding mode of IMP-1575 was determined through both photoaffinity labeling and cryo-EM, indicating that IMP-1575 binds to the HHAT active site and is a potent competitive inhibitor of Pal-CoA (*K*_i_ = 38 nM), blocking substrate access to two key catalytic residues (Asp339 and His379) and causing rearrangement of a gatekeeper residue (Trp335) to block the Pal-CoA binding channel.^14,36^

Here we report the first comprehensive SAR study and profiling of tetrahydropyridine HHAT inhibitors in cells. We synthesized 37 novel derivatives and compared their potency alongside 13 known inhibitors^36−38^ in purified HHAT assays and cytotoxicity screens, and progressed compounds with high inhibitory potency and low cytotoxicity into a cell-based substrate tagging assay for in-cell target engagement, alongside a dual-luciferase reporter assay to assess downstream SHH pathway inhibition. We demonstrate that IMP-1575 and its inactive enantiomer are robust tool molecules to study HHAT inhibition in cells, and deliver a comprehensive pharmacophore determination for HHAT inhibition, off-target toxicity, and metabolic stability.

## Results and Discussion

### Design and Synthesis of HHAT Inhibitor Analogues

**Figure 2:**
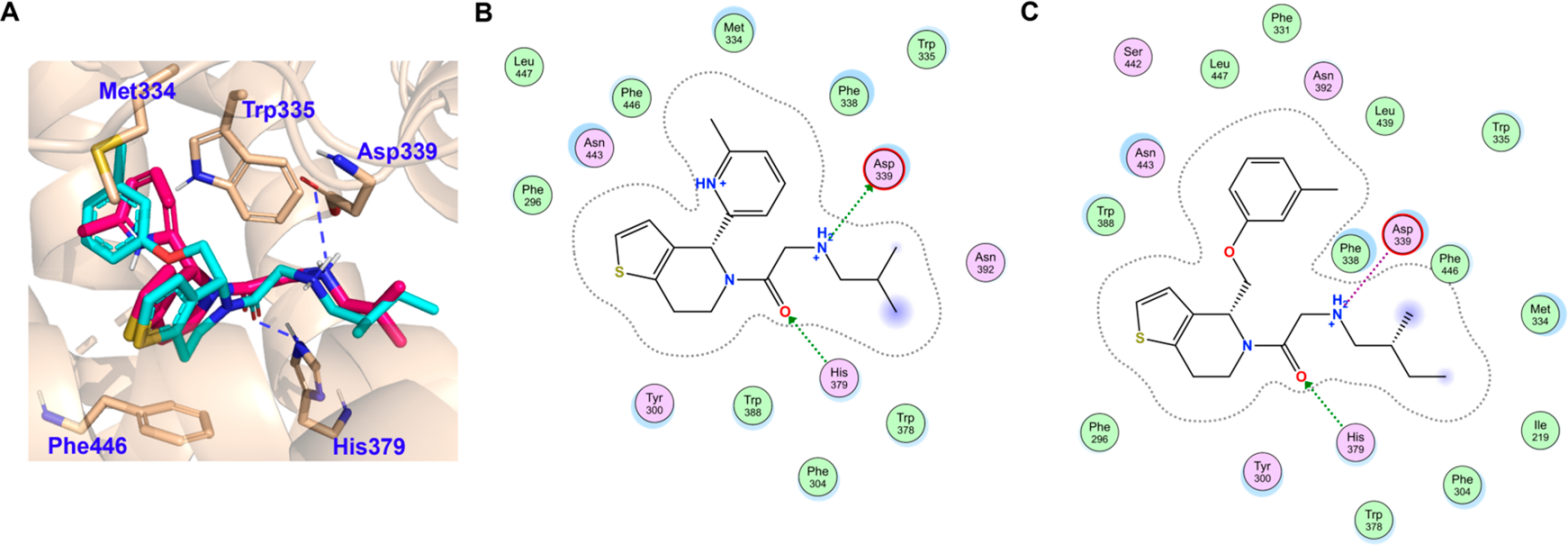
Structural analysis of IMP-1575 analogues. (A) Comparison of predicted HHAT binding modes of (*R*)-enantiomer of **3** (cyan) with reference compound IMP-1575 (**30**, pink, PDB 7Q6Z);^14^ (B) 2D interaction scheme of IMP-1575; (C) 2D interaction scheme of RUSKI-43 (**3**). Docking was performed with Molecular Operating Environment (MOE 2022), pictures were generated in MOE or PyMOL. Hydrogen bonds are labeled with dash lines.

Our inhibitor-bound HHAT cryo-EM structure demonstrates that IMP-1575 (**30**) binds to the active site of HHAT, forming two hydrogen bonds.^14^ One is between the carbonyl of IMP-1575 and HHAT His379; the other between the secondary amine of IMP-1575 and Asp339 (Figure 2). As noted above, IMP-1575 causes a rearrangement of Trp335 in the reaction center and thereby blocks Pal-CoA loading, consequently inhibiting HHAT activity. Our preliminary SAR investigation of analogues of RUSKI-201 also revealed that the amine is critical for inhibition, and changes to the nitrogen position or substitution lead to a decrease in activity.^36^ The cryo-EM structure also confirms that the stereogenic center of IMP-1575 is the (*R*) configuration. In addition, docking studies suggested that the (*R*)-enantiomer of RUSKI-43 (**3**) occupied a similar space in the pocket (Figure 2), forming two hydrogen bonds with the same two residues (Asp339 and His379) as IMP-1575. The binding modes of IMP-1575 and RUSKI-43 in HHAT show that there are additional binding cavities around the aliphatic amine chain, thiophene ring, and 4-position of the core. We synthesized and tested 50 analogues to investigate whether favorable binding contacts could be formed in these regions, using various amino acids to introduce substituents into the α-position of the aliphatic amine chain. The thiophene ring was removed or exchanged for different substituted aromatic systems, and the substituent at the 4-position of the tetrahydropyridine core was varied with differently substituted aromatic or aliphatic rings.

**Scheme 1:**
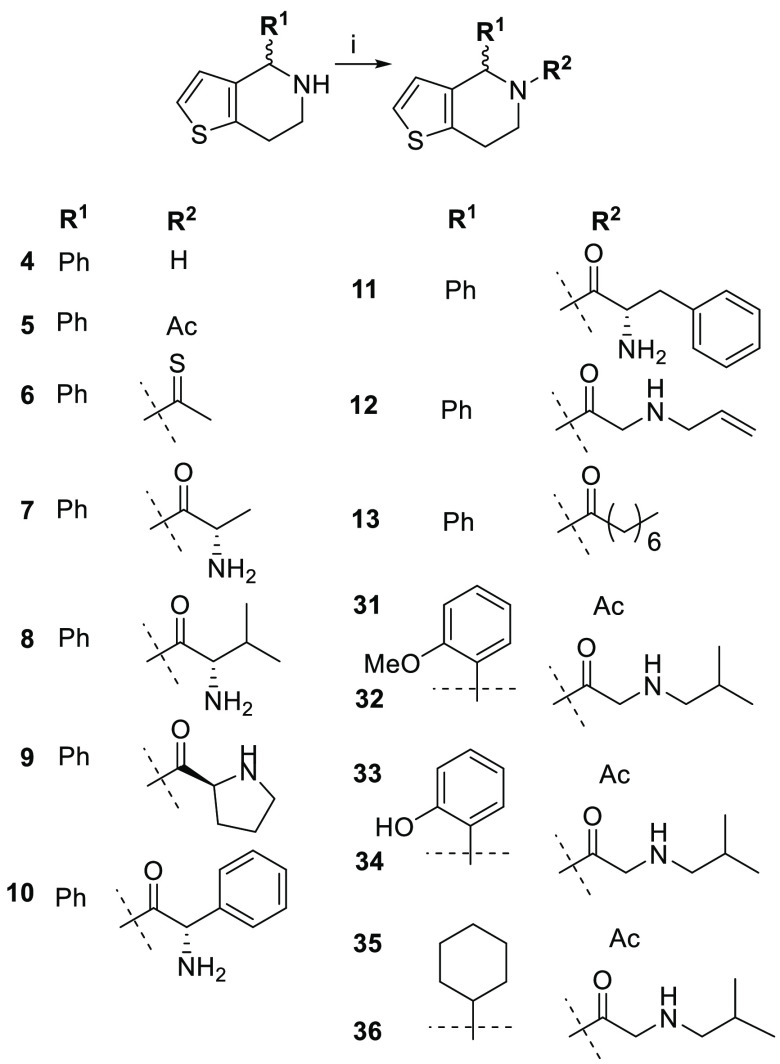
Synthesis of 4,5,6,7-Tetrahydrothieno[3,2-*c*]pyridines Reagent and conditions: (i) (a) carboxylic acids, EDC, HOBt, DIPEA, DCM, RT; (b) TFA, DCM, RT (for compounds **7**–**12**, **32**, **34**, and **36**).

**Scheme 2:**
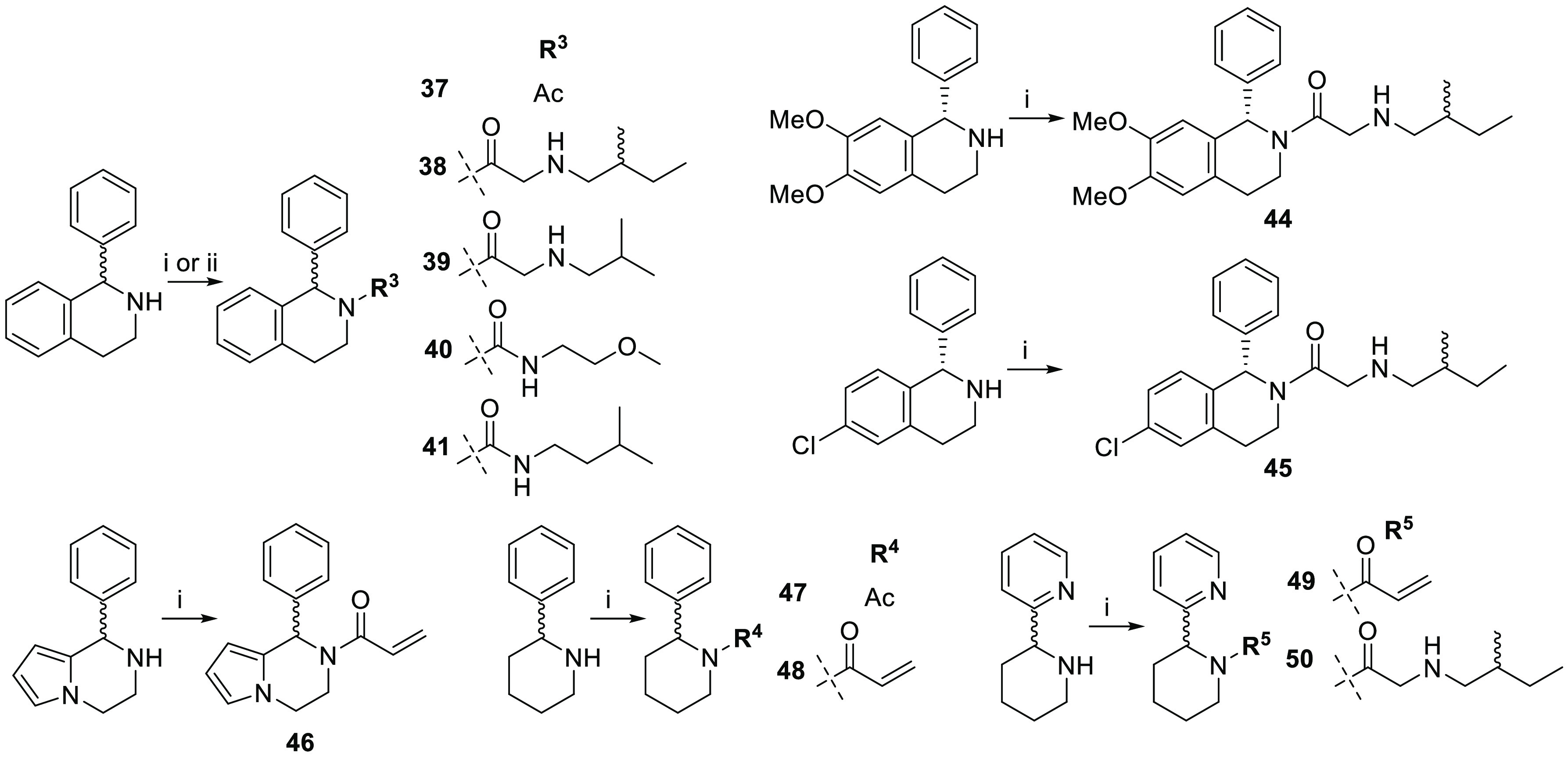
Synthesis of IMP-1575 Analogues Reagent and conditions: (i) (a) carboxylic acids, EDC, HOBt, DIPEA, DCM, RT; (b) TFA, DCM, RT (for compounds **38**, **39**, **44**, **45**, and **50**); (ii) 1,1′-carbonyldiimidazole, amines, DCM, RT (for **40** and **41**).

We have previously reported synthetic routes to this class of HHAT inhibitors,^36−38^ which can be divided into four groups based on pharmacophores. Compounds **1**–**36** are 4,5,6,7-tetrahydrothieno[3,2-*c*]pyridines and **37**–**45** are 1,2,3,4-tetrahydroisoquinolines. Compound **46** contains a 1,2,3,4-tetrahydropyrrolo[1,2-*a*]pyrazine core, while compounds **47**–**50** have a piperidine core. The synthesis of some analogues (**1**–**6**, **14**–**26**, **28**, **30**, **31**, **33**, and **35**) has been reported previously,^36−38^ and new analogues were prepared following the synthetic methods in Schemes 1 and 2. In general, Bischler–Napieralski cyclization and subsequent reduction of the imine^39^ were employed to prepare the heterocyclic cores, which were coupled with various carboxylic acids, followed by Boc-deprotection where required to afford the final compounds. Urea-linked analogues were synthesized via carbonyldiimidazole (CDI) mediated coupling reaction. Chiral preparative HPLC was used to obtain the (*S*)- or (*R*)-enantiomers in >95% *ee*. Analogues are numbered and grouped based on their structures (Table 1 and Table 2); all final products were characterized by ^1^H NMR, ^13^C NMR, and HRMS, with enantiomers further confirmed by chiral HPLC.

**Table 1:**
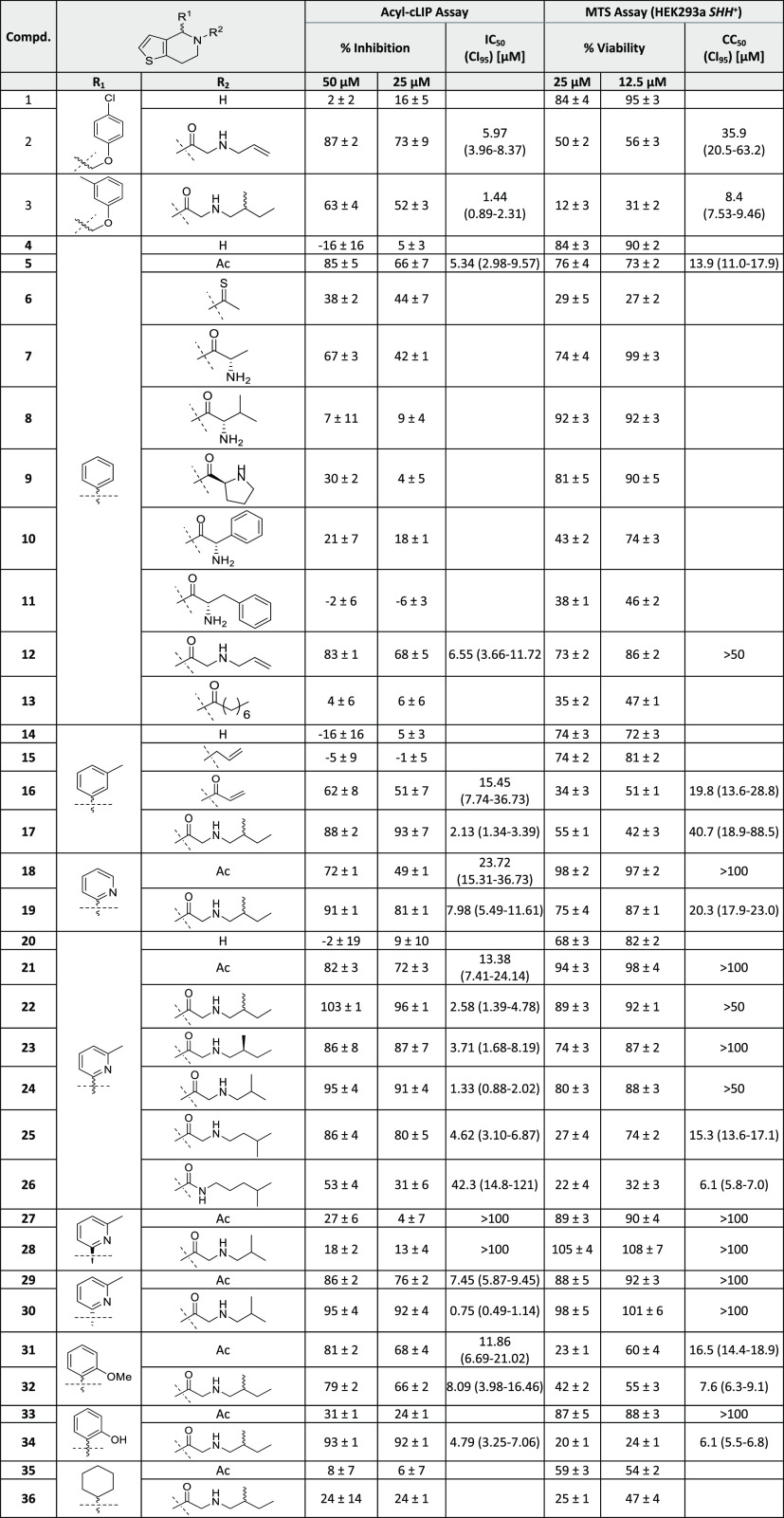
Summary of the Acyl-cLIP Assay and MTS Assay Results for 4,5,6,7-Tetrahydrothieno[3,2-*c*]pyridines

**Table 2:**
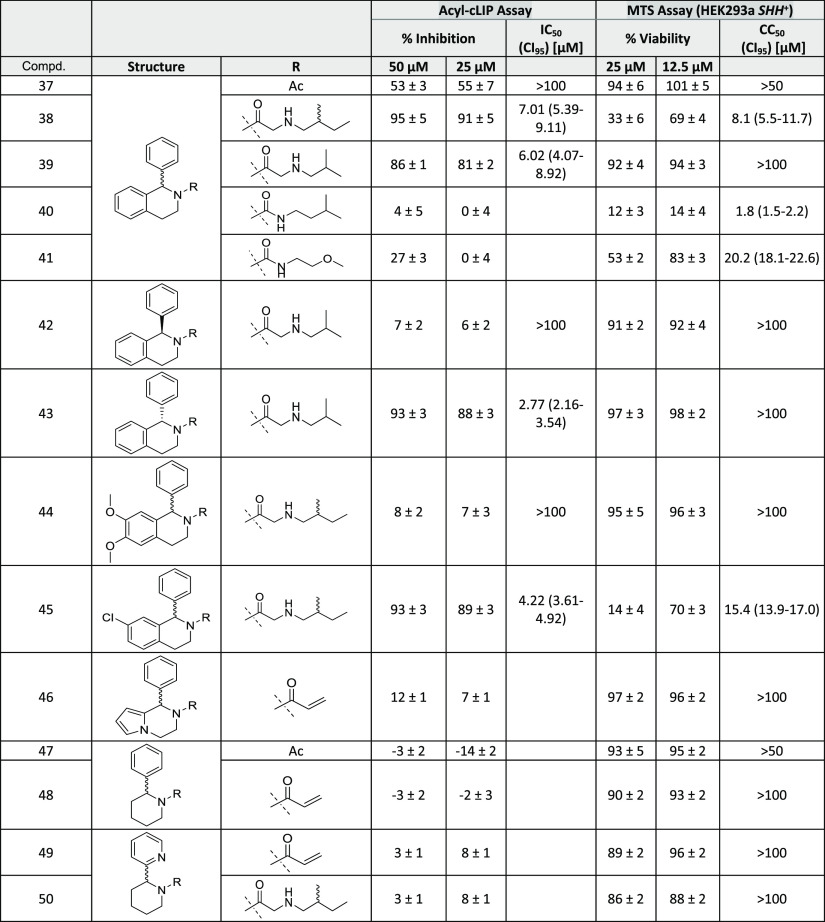
Summary of Acyl-cLIP Assay and MTS Assay Results for Piperidines

**Scheme 3:**
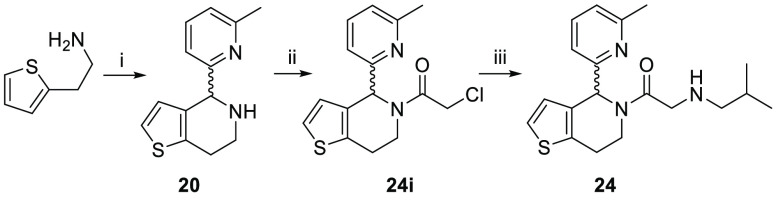
Synthesis of Compound **24** Reagent and conditions: (i) (a) 6-methylpicolinaldehyde, EtOH, TEA, RT, 15 h; (b) TFA, RT, 30 min; (ii) chloroacetyl chloride, TEA, RT; (iii) isobutylamine, neat, RT.

To further expedite analogue synthesis, we recently developed a more concise route toward the synthesis of compound **24**. In our optimized synthetic route, a Pictet–Spengler reaction is used to build the 4,5,6,7-tetrahydrothieno[3,2-*c*]pyridine core in a one-pot reaction from 2-thiopheneethylamine and 6-methylpicolinaldehyde with higher yields.^36^ To accelerate side chain synthesis, chloro acetamide was installed to provide a functional group that can be coupled to various amines (Scheme 3). This improved route offers the potential to rapidly prepare analogues with a diverse range of substitutions.

### Analysis of Purified HHAT Inhibition Using the Acyl-cLIP Assay

The inhibitory potency of all 50 compounds against purified HHAT was quantified using the acylation-coupled lipophilic induction of polarization (acyl-cLIP) assay recently developed in our lab, a universally applicable assay for high-throughput analysis of protein–lipid transferases and hydrolases.^31,40^ In brief, in this assay a fluorescently labeled peptide based on the N-terminal sequence of SHH is incubated with *N*-dodecyl β-d-maltoside (DDM) solubilized purified HHAT, palmitoyl-CoA, and HHAT inhibitors. Binding of the lipidated peptide product to the DDM micelle results in decreased tumbling rate and an increase in polarization of fluorescence emission, enabling facile quantification of HHAT activity. Initially, % HHAT inhibition was determined at 50 μM and 25 μM for all analogues. Subsequently, IC_50_ values were obtained for all compounds that showed an HHAT inhibition >50% at 25 μM (Table 1 and 2, more details in Supporting Information (SI), Table S1 and Figure S1). HHAT is a 12-pass TM protein,^14^ and we considered whether lipophilicity might bias inhibition; pIC_50_ values were compared to calculated log *D*, which indicated no significant correlation (SI, Table S1 and Figure S2). We concluded that lipophilicity is not a general driver of HHAT inhibitory potency.

We first explored the SAR of 4,5,6,7-tetrahydrothieno[3,2-*c*]pyridines. Acyl-cLIP assays revealed that nonacylated core molecules (**1**, **4**, **14**, and **20**) were inactive against HHAT. Allylated compound **15** also shows no inhibitory effect against HHAT, in line with structural analysis indicating the carbonyl oxygen of the side chain forms a hydrogen-bond to His379 (Figure 2A). The existence of this interaction is further supported by the different activity between acetamide compound **5** and the less potent thioamide derivative **6**, because thioamides are generally weaker hydrogen-bond acceptors than oxoamides.^41^ To further explore whether additional groups could be accommodated in the binding cavity, the side chain was replaced by α-functionalized natural or unnatural amino acids (**7**–**11**). Remarkably, no compound in this subseries showed more than 50% inhibition of HHAT at 25 μM, presumably due to the steric demand of the additional substituents at the α-carbon of the side chain. Additionally, the decreased activity of allyl compound **15** compared to acrylamide **16**, as well as the potency of **18** and **21**, all point to the conclusion that an amide bond at the 5-position is crucial for the potency of the analogues.

Although previously identified inhibitors contain a secondary amine in the side chain (e.g., **17** and **22**),^26,35^ which forms an ionic interaction between the secondary amine and Asp339, acetyl and acryloyl derivatives (**5**, **16**, **18**, and **21**) show potency with IC_50_ values of 5.3, 15, 24, and 13 μM against HHAT, respectively, despite their simplified structures. However, changing the side chain to a lipophilic C8 short chain fatty acid (**13**) resulted in a loss of activity, highlighting a size limit in the binding cavity. Analogue **26** in which the amide and the secondary amine was exchanged for a urea regioisomer significantly decreased inhibitory activity in comparison to **25**.^36^ Three different alkyl groups were investigated as secondary amine substituents (compounds **22**, **24**, and **25**); however, the isobutyl of IMP-1575 remained the most favorable substituent. We conclude that both position and substitution of the secondary amine is important to allow formation of the ionic interaction with Asp339.

The 4-position on the core tolerates various substituents containing an aromatic ring. Compounds **2** and **12** both contain a 2-(allylamino)acetyl side chain and exhibit similar potency, despite bearing (4-chlorophenoxy)methyl and phenyl substitutions on the 4-position, respectively. Another group of compounds **3**, **17**, **19**, **22**, **32**, and **34**, which only differ on the 4-position, show IC_50_ values between 1.4 and 8.1 μM, again illustrating that the 4-position has high tolerability toward substitution. Interestingly, compounds **3**, **17**, and **22**, which all feature a *meta*-methyl group on the 4-position aromatic ring, are about 2–5-fold more potent than analogues **19**, **32**, and **34**. Exchanging the aryl substituent with an aliphatic cyclohexane ring led to the loss of activity of compounds **35** and **36**, suggesting a potential π-stacking interaction in aryl derivatives. Taken together, aryl containing substituents are well tolerated on the 4-position of 4,5,6,7-tetrahydrothieno[3,2-*c*]pyridines and show potential for further optimization to improve binding potency.

The absolute configuration at the 4-position is critical for the inhibitory potency of 4,5,6,7-tetrahydrothieno[*3,2-c*]pyridines against HHAT. The (*S*)-enantiomers (**27** and **28**) have no inhibitory activity against HHAT, while the (*R*)-enantiomers (**29** and **30**) show a 2-fold increase in potency compared to racemates **21** and **24**. This important observation is in line with the binding mode and docking studies which show the (*R*)-configured core is critical for forming the two key hydrogen bonds. Docking of the (*S*)-enantiomer **28** showed a large deviation from the binding mode of IMP-1575 for the top 5 poses (SI, Figure S3) and suggested that the two key hydrogen bonds were lost, consistent with lack of inhibitory potency in **28**. In contrast, inhibitors containing racemic or (*S*)-enantiomer of the (2-methylbutyl)glycine side chain (**22** and **23**) show no difference in activity against HHAT, indicating this chiral center is not critical for binding.

**Figure 3:**
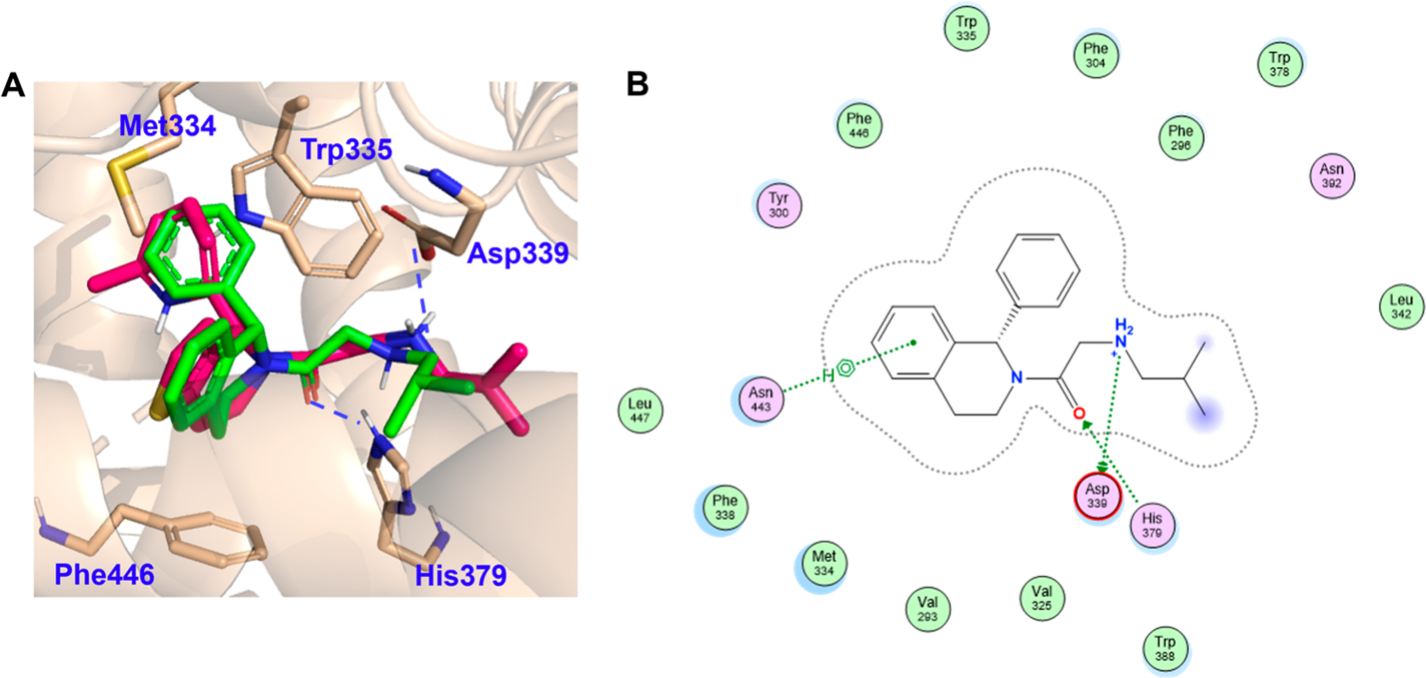
Binding mode analysis of **43**. (A) Comparison of (*R*)-enantiomer **43** (green) and HHAT binding modes with IMP-1575 (pink, PDB 7Q6Z);^14^ (B) 2D interaction scheme of **43**. Docking was performed with Molecular Operating Environment (MOE 2022), pictures were generated in PyMOL. Interactions are labeled with dash lines. More details are shown in SI, Figure S3.

Having analyzed the impact of the 4-position aromatic group and the secondary amine side chain on inhibitory potency, we next investigated the thiophene moiety of IMP-1575. HHAT inhibitory potency was preserved when changing the thiophene to a phenyl ring, as long as the favorable substituents on the side chain were present (**38** and **39**). However, with an acetyl group at this position, **37** shows decreased activity compared to **38**. Similar to compound **26**, replacing the amide with a urea also led to inactivity of the tetrahydroisoquinoline analogues (**40** and **41**). The importance of the absolute conformation of the 4-position was also confirmed with this new core; the (*R*)-enantiomer (**42**) had no inhibitory activity, while the (*S*)-enantiomer (**43**) was 2-fold more potent than the racemate (**39**). Electron-donating dimethoxy substituents on the phenyl ring (**44**) resulted in a complete loss of HHAT inhibition. Decreasing electron density in the core by introducing a *meta* chloro substituent (**45**) did not interfere with inhibitory activity, while exchanging the thiophene with a pyrrole moiety (**46**) resulted in a loss of potency. Removing the heteroaromatic ring (**47**–**50**) resulted in a loss of potency. These findings indicate that a thiophene or phenyl substituent is essential; however, the influence on rigidity of the core may be more important than the effective electron density for this moiety. This can be explained by the X–H···π interaction between HHAT Asn443 and the thiophene of IMP-1575 or phenyl of **43** (Figure 3).

### Toxicity of Compounds

**Figure 4:**
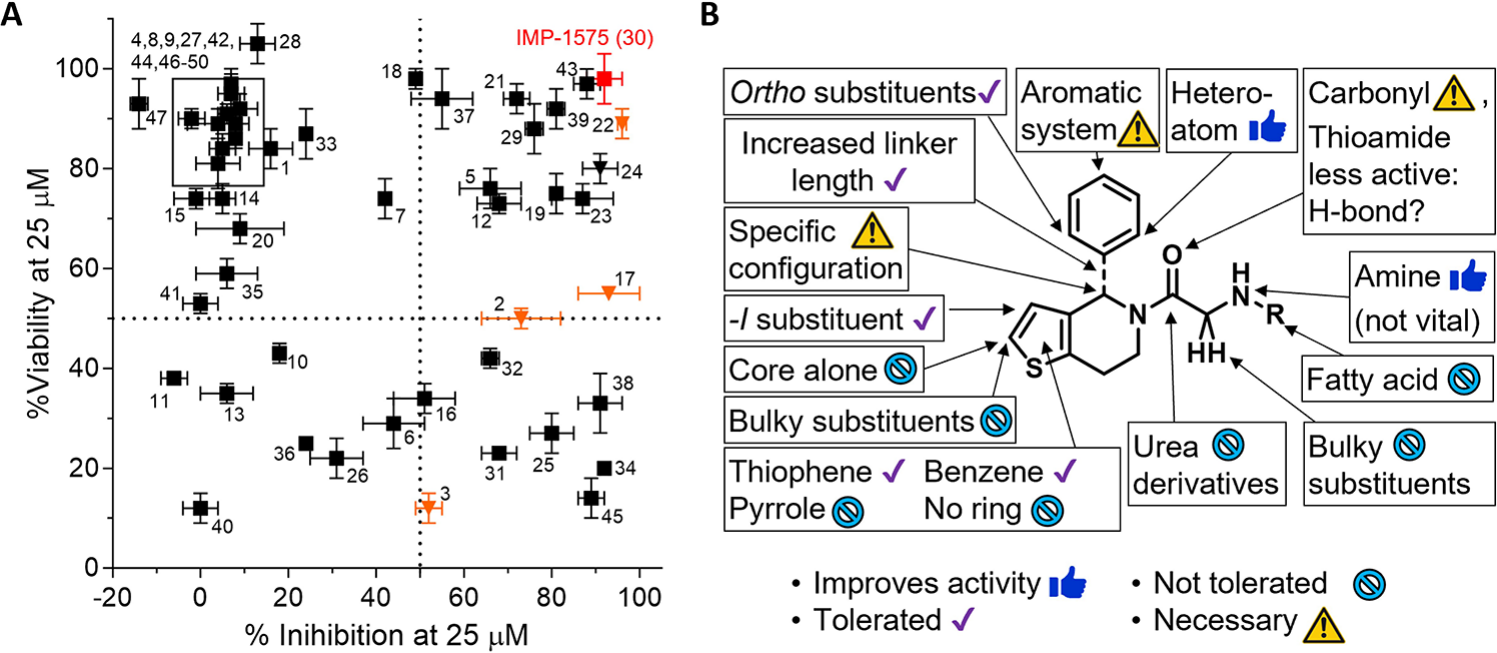
Cytotoxicity and structure–activity relationship of IMP-1575 analogues. (A) Plot of % viability at 25 μM determined by MTS proliferation assay using HEK293 *SHH*^*+*^ cells versus % inhibition at 25 μM from the Acyl-cLIP assay. Previously reported HHAT inhibitors (orange triangle) and IMP-1575 (red solid square) are highlighted. Results are shown as average ± SEM, *n* = 3.^27^ (B) Summary of the conclusions from the structure–activity relationship study.

The survival of HEK293a *SHH*^*+*^ cells is independent of HH signaling,^35^ therefore, the cytotoxicity of analogues was tested using the MTS assay to investigate off-target toxicity. In this assay, HEK293a *SHH*^*+*^ cells were treated with DMSO vehicle or varying concentrations of analogues for 72 h. Subsequently, a mixture of 1-methoxy phenazine methosulfate (PMS) and [3-(4,5-dimethylthiazol-2-yl)-5-(3-carboxymethoxyphenyl)-2-(4-sulfophenyl)-2*H*-tetrazolium] (MTS) was added to enable quantification of cell number via colorimetric analysis. The majority of active HHAT inhibitors exhibited minimal effect on cell viability (<30%) at 12.5 μM (compounds **5**, **12**, **18**, **19**, **21**-**25**, **29**, **30**, **39**, **43**, and **45**), while compounds **2** and **3** induced 44% and 69% decrease in cell survival, respectively, consistent with our previous finding that the mechanism-of-action for cytotoxicity of **2** and **3** is independent of HHAT (SI, Table S2 and Figure S4).^35^ Compounds **3**, **17**, **19**, **32**, **34**, **38**, and **45**, which all contain a 2-((2-methylbutyl)amino)acetyl, were toxic to HEK293a *SHH*^*+*^ cells, as were ureas **26**, **40**, and **41**. In contrast, analogues **22**, **23**, **24**, **29**, **30**, **39**, and **43** showed minimal toxicity in HEK293a *SHH*^*+*^ cells (50% cytotoxic concentration (CC_50_) > 50 μM) and good on-target potency in purified enzyme assays (HHAT IC_50_ < 10 μM), indicating these analogues were suitable for further progression toward target engagement studies (Figure 4).

### Characterizing Inhibitory Potency in Cellular Signaling

Compounds that showed both IC_50_ < 20 μM in the acyl-cLIP assay and CC_50_ > 10 μM in the MTS assay against HEK293a *SHH*^+^ were progressed into cellular HH signaling assays (SI, Figure S2). The second selection step was essential to exclude artifacts resulting from nonspecific cytotoxicity, which would decrease viability and therefore signaling from SHH expressing cells. Inactive (*S*)-enantiomer **28** was included as a control to investigate the stereochemical stability of the tetrahydrothieno[3,2-*c*]pyridine core in a cellular environment and to further control for off-target effects.

**Figure 5:**
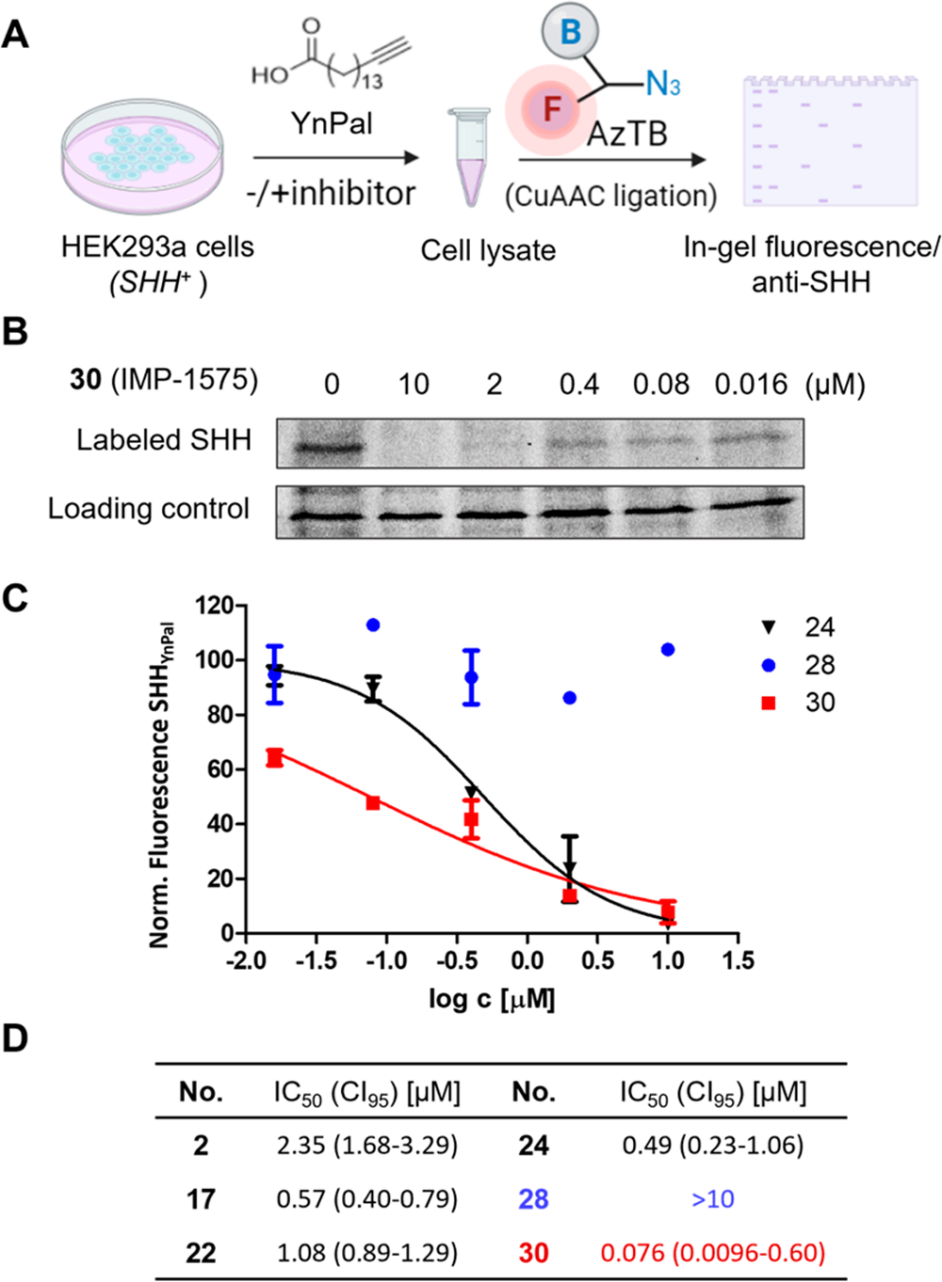
YnPal tagging assay with **2**, **17**, **22**, **24**, **28**, and **30**. (A) Schematic illustration of the tagging assay; (B) Inhibition of SHH-YnPal tagging by **30** using AzTB fluorescence signal; (C) IC_50_ values of tested inhibitors in tagging assay. (D) The densitometric results from two replicates were normalized using the two controls (no YnPal and DMSO vehicle) and plotted against the logarithm of the inhibitor concentration. Results are shown as average ± SEM, *n* ≥ 2; IC_50_ values were extracted by nonlinear regression using a sigmoidal dose response model. CI_95_ = 95% confidence interval.

We first quantified the direct impact of previously reported HHAT inhibitors on palmitoylation of SHH using a cell-based metabolic tagging assay.^27,36^ In this assay, HEK293a *SHH*^+^ cells were incubated with varying concentrations of inhibitors in the presence of alkyne palmitic acid derivative YnPal (Figure 5A).^35^ Following cell lysis and bioorthogonal derivatization of palmitoylated proteins with capture reagent azide-TAMRA-biotin (AzTB)^67^ using a copper catalyzed azide–alkyne cycloaddition (CuAAC) reaction, AzTB-labeled SHH is shifted to higher apparent molecular weight in sodium dodecyl sulfate polyacrylamide gel electrophoresis (SDS-PAGE), thereby enabling quantification of acylated SHH by α-SHH immunoblotting (compound **2**, **17**, and **22**) or fluorescence imaging (compound **24**, **28**, and **30**; SI, Figure S5–S6).^42^ HHAT inhibitors that exhibited high potency in the acyl-cLIP assay also strongly inhibited SHH tagging by YnPal. Five tested compounds (**2**, **17**, **22**, **24**, and **30**) showed low μM to nM potency (Figure 5B–D); notably, compound **30** (IMP-1575) was the most potent and inhibited cellular YnPal labeling of SHH with a IC_50_ of 76 nM, while the (*S*)-configuration counterpart **28** was inactive. These results demonstrate that compounds **28** and **30** remain stereochemically stable in a cellular environment and, moreover, **30** is highly potent in both enzymatic and cellular assays of HHAT function.

**Figure 6:**
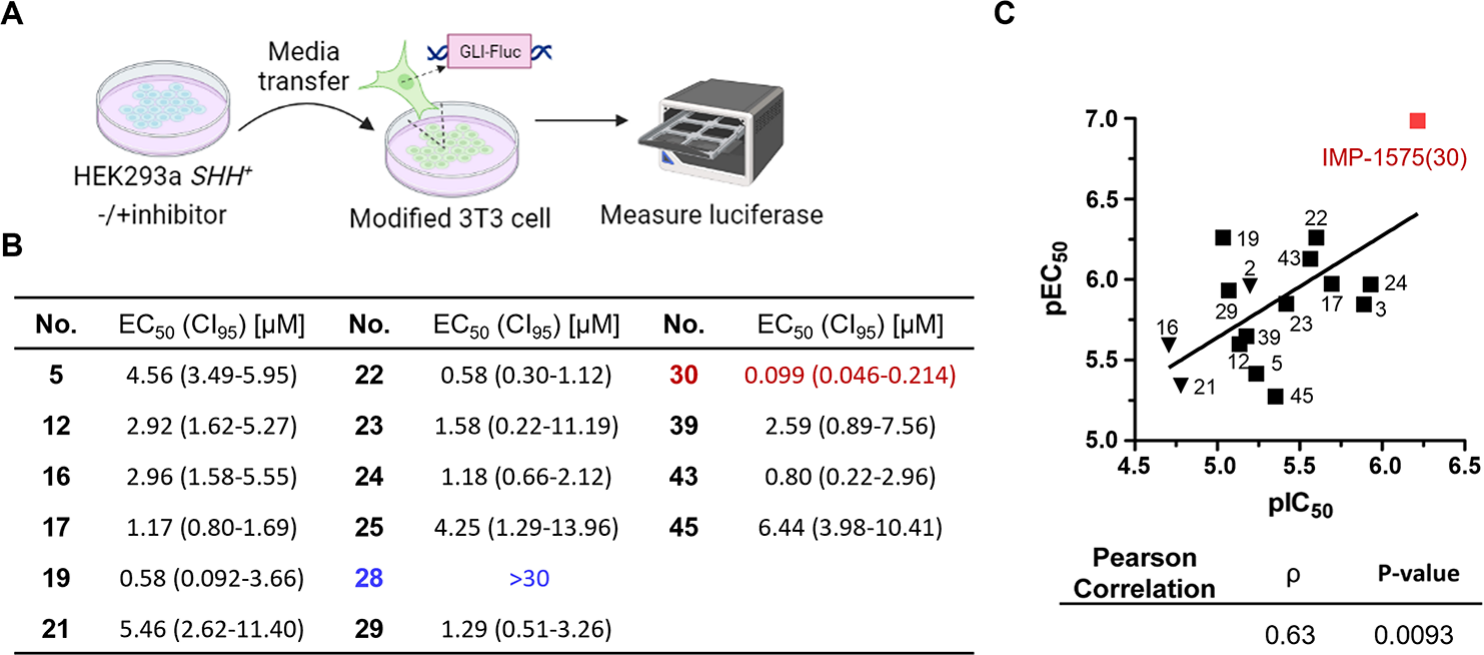
Effect of HHAT inhibitors on cellular HH signaling. (A) Schematic illustration of the luciferase-based HH signaling assay. (B) Cell-signaling assay results of selected analogues. The values were extracted from the dose–response curves shown in SI, Figure S7, using a sigmoidal dose–response model in GraphPad Prism 5. CI_95_ = 95% confidence intervals (*n* = 3). (C) Correlation of the cell based signaling assay results (pEC_50_) and the Acyl-cLIP assay results (pIC_50_);^35^ ρ = Pearson correlation coefficient. Previously reported HHAT inhibitors (orange triangle) and IMP-1575 (red solid square) are highlighted.

Subsequently, the effect of analogues on HH signaling was investigated using a cellular luciferase reporter assay. Light2 cells derived from NIH3T3 cells constitutively express Renilla luciferase as an internal control for cell density alongside Firefly luciferase under the control of a HH-responsive *Gli* promoter.^43^ HEK293a *SHH*^+^ cells were incubated with varying inhibitor concentrations and SHH-containing conditioned media transferred to the NIH3T3-Light2 cells to mimic paracrine signaling^30,35^ (Figure 6A). In this assay, **2**, **17**, **22**, and **24** showed low μM to nM potency,^35^ while **28** was inactive, in good agreement with the tagging assay, and highlighting the direct dependence of HH signaling on HHAT activity in cells (SI, Figure S6 and Table S3). Four compounds (**19**, **22**, **30**, and **43**) exhibited nM potency against HH signaling, with IMP-1575 (**30**) again exhibiting the most potent EC_50_ of 99 nM (Figure 6B, and SI, Figure S7). Furthermore, comparison of pEC_50_ values from the Light2 cellular signaling assay against the pIC_50_ data from the acyl-cLIP biochemical assay revealed a Pearson’s correlation coefficient ρ of 0.71 (*p* = 0.0021) (Figure 6C) between the cellular and enzymatic potencies of the inhibitors. Taking these data together, we conclude that IMP-1575 represents the optimal tool HHAT inhibitor to-date, with nM potency in both enzymatic assay and cellular assays and no detected off-target cytotoxicity.

### Metabolic Stability and Pharmacokinetic Analysis

**Figure 7:**
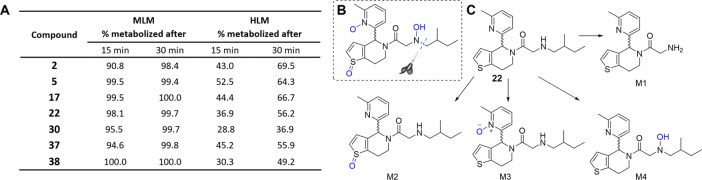
Metabolic stability of HHAT inhibitors in microsomes. (A) The rate of metabolism of compounds in mouse liver microsomes (MLM) and human liver microsomes (HLM). (B) Proposed labile sites of **22** identified in microsomal incubation assay (labeled blue). (C) Four single site metabolized products M1–4 are shown.

To assess the potential of HHAT inhibitors for in vivo target validation, drug metabolism and permeability studies were undertaken. The parallel artificial membrane permeability assay (PAMPA) was performed with compounds **2**, **5**, **17**, **22**, **37**, and **38** (SI, Tables S5 and S6), where all compounds except compound **17** showed high passive permeability at pH 7.4. The passive permeability of compounds containing the secondary amine in the side chain (**2**, **17**, **22**, and **38**) was pH dependent (SI, Table S4), while the acetylated derivatives **5** and **37** showed high permeability over the full pH range tested. The Caco-2 assay for compounds **2** and **22** indicated that these compounds had moderate-high permeability with no detectable efflux (SI, Table S5). However, microsomal stability experiments with inhibitors **2**, **5**, **17**, **22**, **30**, **37**, and **38** (Figure 7A) indicated that all tested compounds were rapidly metabolized (>90% after 15 min) in mouse liver microsomes (MLMs), which undermines the applications of this series for in vivo studies. Metabolite identification by mass spectrometry on **22** in MLM was performed to identify metabolically unstable sites, detecting five major metabolites and four possible labile sites (Figure 7B,C, and SI, Figure S8 and Table S6 and S7). Metabolites include *N*-dealkylation resulting from an isopentane loss in the side chain, two mono-oxidation and two bis-oxidation products that could be caused by *S*-oxidation at the thiophene moiety, *N*-oxidation at the secondary amine^44^ and *N*-oxidation of the pyridine moiety^45^ leading to hydroxylate derivatives. Taken together, compounds from this chemical series are cell permeable but metabolically unstable. Our findings on the pharmacophore for HHAT inhibition will help to guide future studies to improve the metabolic stability of this series of HHAT inhibitors.

## Conclusions

HHAT inhibition holds high promise to block HH signaling from cancer cells. Supported by information from recent cryo-EM structures of HHAT, we have designed, synthesized, and evaluated 50 inhibitor analogues, including the most potent HHAT inhibitor reported in the literature to-date, IMP-1575. SAR analysis demonstrated that an aromatic ring with (*R*)- absolute configuration at the 4-position of the core and the 5-position amide carbonyl are essential for HHAT inhibition. Cellular assays show that IMP-1575 has no detectable off-target toxicity in vitro and inhibits the palmitoylation of SHH as well HH signaling with nM potency in cells; an interesting question for future investigation remains whether HHAT inhibition impacts HH secretion as well as signaling. The (*S*)-enantiomer **28** shows no inhibition in both enzyme and cellular assays and, in combination with IMP-1575, therefore provides a powerful set of tool molecules for investigation of HHAT activity in cells. We further determined that compounds from this series are highly metabolically unstable in mouse and human microsomes, at positions within the pharmacophore required for target inhibition. We conclude that new series of HHAT inhibitors are likely to be required to progress to HHAT target validation in vivo. In summary, we present IMP-1575 as an optimum molecule to investigate SHH signaling via HHAT inhibition in vitro (Table S7), along with extended SAR understanding to guide development of HHAT inhibitors in future.

## Experimental Section

### Synthesis of HHAT Inhibitor Analogues

#### General

Chemicals were obtained from Sigma-Aldrich (Irvine, UK), Fluorochem (Hadfield, UK), or Enamine (Riga, Latvia) and used without further purification. ^1^H and ^13^C NMR spectra were recorded at room temperature (RT) at 500 and 125 MHz, or 400 and 101 MHz, respectively. Chemical shifts (δ) are reported in parts per million (ppm) relative to residual solvent peaks as internal standard. Coupling constants (*J*) are reported in hertz (Hz). High resolution mass spectrometry (HRMS) was performed using electrospray ionization (ESI) and time-of-flight (TOF) mass analysis. Analytical chiral HPLC was performed on an Agilent 1260 Infinity Series equipped with a CHIRALPAK-IF 4.6 mm × 250 mm (eluent: isocratic hexane:propan-2-ol 90:10 or 80:20; flow rate 1 mL/min, method A). Preparative chiral HPLC was performed on an Agilent 1200 Series equipped with a Chiralpak-IF 250 mm × 20 mm column (eluent: isocratic hexane:propan-2-ol 90:10, flow rate 18 mL/min, method B). The synthesis of intermediates **12i**, **13i** and compounds **1**–**6**, **14**–**18**, **20**–**26**, **28**, **30**, **31**, **33m**, and **35** has been reported elsewhere.^36−38^ The details on the intermediate’s synthesis are reported in the Supporting Information. The purity of all final compounds was >95%.

#### General Procedure A (Coupling of Side Chain Using EDC/HOBt)

The corresponding acid (1 equiv), HOBt (1 equiv), and 1-ethyl-3-(3-(dimethylamino)propyl)carbodiimide (EDC, 1.5 equiv) were dissolved in DMF (5 mL), and the reaction mixture was stirred at RT for 30 min. Subsequently, the corresponding amine core (1 equiv) and *N*,*N*-diisopropylethylamine (4 equiv) were added, and the reaction mixture was stirred overnight at RT. The mixture was diluted with dichloromethane (DCM, 8 mL) and washed with water, 5% LiCl solution, and brine. The organic layer was dried over MgSO_4_ and concentrated under reduced pressure to yield the crude product that was further purified by column chromatography.

#### General Procedure B (Boc Deprotection)

The protected amine was dissolved in DCM (5 mL), trifluoroacetic acid (TFA, 5 mL) was added, and the reaction mixture was stirred for 3 h at RT. The solvent was removed under reduced pressure, and the residual was neutralized using a saturated solution of NaHCO_3_. The product was extracted with DCM and the organic layer was dried over MgSO_4_ and concentrated under reduced pressure. Compounds were either used without further purification or purified by column chromatography.

#### General Procedure C (Boc Deprotection Variant b)

The protected amine was dissolved in DCM (5 mL), TFA (5 mL) was added, and the reaction mixture was stirred for 3 h at RT. The deprotected amine was purified using an ISOLUTE SCX column. After addition of the reaction mixture, the column was washed with 4 column volumes (CV) of methanol, followed by elution using 3 CV of 7 M ammonia in methanol. The elution fractions were concentrated under reduced pressure to yield the deprotected compound.

#### General Procedure D (Coupling of Acid Chloride Derivatives)

The corresponding acid chloride (3–4 equiv) was added slowly to a mixture of the corresponding amine core (1 equiv) and trimethylamine (6 equiv) in dry DCM. The reaction was stirred at RT for 2 h. Subsequently, the solvent was removed in vacuum and the residual was purified by column chromatography.

#### General Procedure E (Synthesis of Urea Derivatives)

The primary amine (1 equiv) was added to a solution of CDI (1.5 equiv) in DCM (1 mL) and the mixture was stirred at RT for 1 h. The amine core (1.25 equiv) was added, and the reaction mixture was stirred at RT overnight. The solvent was removed in vacuo and the urea compound was purified via flash column chromatography.

##### 2-Chloro-1-(4-(6-methylpyridin-2-yl)-6,7-dihydrothieno[3,2-*c*]pyridin-5(4*H*)-yl)ethan-1-one (**24i**)

Chloroacetyl chloride (15 μL, 0.18 mmol) was added to a solution of compound **20** (42 mg, 0.18 mmol) in DCM (8 mL). The resulting mixture was stirred at RT for 0.5 h. The reaction mixture was extracted with water (20 mL) and DCM (20 mL). The organic layer was collected and the crude residue was purified by chromatography using *n*-hexane: EtOAc (3:1) to give compound **24i** as a colorless oil (0.14 mmol, 42 mg, 75%). *R*_*f*_ = 0.2 (*n*-hexane/EtOAc 5:1). ^1^H NMR (400 MHz, CDCl_3_) δ 7.53 (dt, ^3^*J* = 12.7, 7.6 Hz, 1H), 7.19 (t, ^2^*J* = 5.6 Hz, 1H), 7.10 (t, ^3^*J* = 6.2 Hz, 1H), 6.85 (d, ^3^*J* = 5.1 Hz, 1H), 6.78 (d,^3^*J* = 7.7 Hz, 1H), 6.12 (s, 1H), 4.95 (d, ^3^*J* = 12.7 Hz, 1H), 4.55 (d, ^3^*J* = 12.8 Hz, 1H), 4.21–4.09 (m, 1H), 3.08–2.83 (m, 3H), 2.52 (s, 3H). MS (ESI) *m*/*z*: calculated for C_15_H_16_ClN_2_OS [M + H]^+^: 307.06, found: 307.06.

##### (2*S*)-2-Amino-1-(4-phenyl-6,7-dihydrothieno[3,2-*c*]pyridin-5(4*H*)-yl)propan-1-one (**7**)

The Boc-protected **7** precursor **7a** was prepared following general procedure A from *N*-(*tert*-butoxycarbonyl)-l-alanine (0.26 mmol, 48 mg) and compound **4** (0.23 mmol, 50 mg). The crude product was purified by column chromatography to give compound **7a** as a colorless oil (0.21 mmol, 82 mg, 82%). *R*_*f*_ = 0.73 (*n*-hexane/EtOAc 1:1). ^1^H NMR (400 MHz, CDCl_3_) δ (ppm): 7.31–7.26 (m, 4H), 7.21–7.20 (m, 1H), 7.16 (d, ^3^*J* = 5.1 Hz, 1H), 6.88 (s, 1H), 6.82 (s, 1H), 6.71 (t, ^3^*J* = 5.7 Hz, 1H), 5.66 (d, ^3^*J* = 7.7 Hz, 1H), 5.55 (d, ^3^*J* = 8.2 Hz, 1H), 4.67–4.63 (m, 1H), 3.99–3.88 (m, 1H), 3.46–3.29 (m, 1H), 3.12–2.87 (m, 2H), 1.46–1.42 (m, 9H), 1.36 (d, ^3^*J* = 6.8 Hz, 2H), 1.27 (d, ^3^*J* = 6.8 Hz, 1H). ^13^C NMR (101 MHz, CDCl_3_) δ (ppm): 171.4, 155.1, 141.0, 140.7, 134.0, 133.6, 128.7, 128.5, 128.4, 128.0, 172.9, 127.3, 126.7, 123.7, 123.5, 79.6, 54.4, 53.88, 46.8, 46.6, 39.3, 28.5, 26.0, 20.1, 19.3.

Compound **7** was obtained from the Boc-protected derivative **7a** (0.19 mmol, 70 mg) using general procedure B as an orange oil (0.12 mmol, 35 mg, 64%) and used without further purification. ^1^H NMR (400 MHz, CDCl_3_) δ (ppm): 7.35–7.24 (m, 4H), 7.23–7.21 (m, 1H), 7.16 (d, ^3^*J* = 5.2 Hz, 1H), 6.88 (d, ^3^*J* = 10.62, 1H), 6.73–6.69 (m, 1H), 3.94–3.82 (m, 2H), 3.44–3.32 (m, 1H), 3.03–2.89 (m, 2H), 2.45 (s, 2H), 1.33 (d, ^3^*J* = 6.4 Hz, 3H), 1.27 (d, ^3^*J* = 6.8 Hz, 3H). ^13^C NMR (101 MHz, CDCl_3_) δ (ppm): 174.3, 141.2, 140.8, 133.7, 133.4, 128.6, 128.4, 127.8, 126.6, 123.5, 54.3, 453.9, 47.3, 38.8, 30.2, 25.9, 21.5. HRMS (ESI) *m*/*z*: calculated for C_16_H_19_N_2_OS [M + H]^+^: 287.1219; found, 287.1218.

##### (2*S*)-2-Amino-3-methyl-1-(4-phenyl-6,7-dihydrothieno[3,2-*c*]pyridin-5(4*H*)-yl)butan-1-one (**8**)

The Boc-protected **8** precursor **8a** was prepared following general procedure A from *N*-(*tert*-butoxycarbonyl)-l-valine (0.26 mmol, 56 mg) and compound **4** (0.23 mmol, 50 mg). The crude product was purified by column chromatography to give compound **8a** as a colorless oil (0.17 mmol, 72 mg, 74%). *R*_*f*_ = 0.40 (*n*-hexane/EtOAc 6:4). ^1^H NMR (400 MHz, CDCl_3_) δ (ppm): 7.34–7.33 (m, 1H) 7.31–7.22 (m, 5H), 7.19 (d, ^3^*J* = 5.2 Hz, 1H), 7.17–7.12 (m, 1H), 7.07 (d, ^3^*J* = 5.2 Hz, 1H), 6.94 (s, 1H), 6.82 (s, 1H), 6.77 (d, ^3^*J* = 5.2 Hz, 1H), 6.73 (d, ^3^*J* = 5.2 Hz, 1H), 6.70 (d, ^3^*J* = 5.2 Hz, 1H), 6.35 (s, 1H), 6.21 (s, 1H), 5.44 (d, ^3^*J* = 9.2 Hz, 1H), 5.37 (d, ^3^*J* = 9.2 Hz, 1H), 5.05 (d, ^3^*J* = 9.2 Hz, 1H), 4.97 (dd, ^3^*J* = 9.3, ^3^*J* = 6.8 Hz, 1H), 4.81–4.69 (m, 1H), 4.55–4.44 (m, 1H), 3.47–3.30 (m, 1H), 3.11–2.81 (m, 2H), 2.04–1.88 (m, 1H), 1.83 (s, 1H), 1.47–1.35 (m, 9H), 1.26 (s, 1H), 0.97 (d, ^3^*J* = 6.8 Hz, 1H), 0.90 (d, ^3^*J* = 6.8 Hz, 2H), 0.89–0.82 (m, 3H). ^13^C NMR (101 MHz, CDCl_3_) δ (ppm): 171.0, 170.8, 156.0, 141.0, 140.7, 134.2, 133.6, 128.9, 128.7, 128.6, 127.8, 126.6, 123.3, 79.5, 55.6, 55.0, 54.7, 53.8, 39.6, 28.3, 26.1, 19.7.

Compound **8** was obtained from the Boc-protected derivative **8a** (0.18 mmol, 75 mg) using general procedure B as an orange oil (0.13 mmol, 42 mg, 72%) and used without further purification. ^1^H NMR (400 MHz, CDCl_3_) δ (ppm): 7.29–7.21 (m, 5H), 7.15 (dd, ^3^*J* = 5.1 Hz, ^3^*J* = 2.6 Hz, 1H), 6.96 (s, 1H), 6.87 (s, 1H), 6.79 (d, ^3^*J* = 5.1 Hz, 1H), 6.71–6.69 (m, 1H), 4.85–4.78 (m, 1H), 3.96–3.86 (m, 1H), 3.67–3.55 (m, 1H), 3.45–3.28 (m, 1H), 3.09–2.79 (m, 2H), 2.50 (s, 2H), 1.97–1.79 (m, 1H), 1.22–1.11 (m, 1H), 1.01–0.84 (m, 6H). ^13^C NMR (101 MHz, CDCl_3_) δ (ppm): 173.3, 172.8, 141.2, 141.0, 134.4, 133.8, 133.5, 128.7, 128.4, 127.8, 126.7, 123.6, 47.3, 56.3, 54.2, 39.3, 39.1, 31.7, 26.6, 20.3, 19.7, 16.4. HRMS (ESI) *m*/*z*: calculated for C_18_H_23_N_2_OS [M + H]^+^: 315.1531; found, 315.1530.

##### (*S*)-4-Phenyl-5-prolyl-4,5,6,7-tetrahydrothieno[3,2-*c*]pyridine (**9**)

The Boc-protected **9** precursor **9a** was prepared following general procedure A from *N*-(*tert*-butoxycarbonyl)-l-proline (0.26 mmol, 56 mg) and compound **4** (0.23 mmol, 50 mg). The crude product was purified by column chromatography to give compound **9a** as a colorless oil (0.10 mmol, 42 mg, 38%). *R*_*f*_ = 0.31 (*n*-hexane/EtOAc 6:4). ^1^H NMR (400 MHz, CDCl_3_) δ (ppm): 7.35–7.29 (m, 1H), 7.30–7.27 (m, 4H), 7.16–7.14 (m, 1H), 7.11 (d, ^3^*J* = 5.2 Hz, 1H), 6.88–6.87 (m, 1H), 6.84 (s, 1H), 6.75 (d, ^3^*J* = 5.2 Hz, 1H), 6.68 (dd, ^3^*J* = 5.1 Hz, ^3^*J* = 1.9 Hz, 1H), 4.71–4.69 (m, 1H), 4.62 (dd, ^3^*J* = 8.5 Hz, ^3^*J* = 3.9 Hz, 1H), 4.52 (dd, ^3^*J* = 8.5 Hz, ^3^*J* = 3.9 Hz, 1H), 4.50–4.01 (m, 1H), 3.93 (dd, ^2^*J* = 13.4 Hz, ^2^*J* = 4.7 Hz, 1H), 3.80 (dd, ^2^*J* = 14.2 Hz, ^3^*J* = 5.2 Hz, 1H), 3.68–3.59 (m, 1H), 3.55–3.38 (m, 2H), 3.30 (td, ^2^*J* = 14.0 Hz,^3^*J* = 12.6, ^3^*J* = 3.8 Hz, 1H), 3.18 (td, ^2^*J* = 14.0, ^2^*J* = 12.6, ^3^*J* = 5.1 Hz, 1H), 3.07 (td, ^2^*J* = 14.0, ^2^*J* = 12.6, ^3^*J* = 5.1 Hz, 1H), 3.01–2.93 (m, 1H), 2.93–2.83 (m, 1H), 2.80–2.73 (m, 1H), 2.24–2.05 (m, 1H), 2.01–1.62 (m, 3H), 1.50–1.13 (m, 9H).

Compound **9** was obtained was obtained from **9a** (0.043 mmol, 18 mg) following general procedure B as an orange oil (0.035 mmol, 10 mg, 81%) and used without further purification. ^1^H NMR (400 MHz, CDCl_3_) δ (ppm): 7.33–7.22 (m, 5H), 7.17 (t, ^3^*J* = 5.1 Hz, 1H), 6.84–6.80 (m, 1H), 6.72–6.70 (m, 1H), 6.01 (s, 1H), 4.71–4.69 (m, 1H), 4.57–4.53 (m, 1H), 4.33–4.27 (m, 1H), 3.92–3.85 (m, 3H), 3.46–3.20 (m, 2H), 3.12–2.80 (m, 3H), 2.32–2.17 (m, 1H), 1.97–1.75 (m, 2H), 1.66–1.59 (m, 1H), 1.32–1.25 (m, 2H), 0.88–0.83 (m, 1H). ^13^C NMR (101 MHz, CDCl_3_) δ (ppm): 128.5, 128.2, 127.9, 126.5, 126.2, 123.6, 58.4, 54.5, 47.4, 47.2, 39.0, 30.6, 26.0, 25.7, 1.0. HRMS (ESI) *m*/*z*: calculated for C_18_H_21_N_2_OS [M + H]^+^: 313.1375; found, 313.1382.

##### (2*S*)-2-Amino-2-phenyl-1-(4-phenyl-6,7-dihydrothieno[3,2-*c*]pyridin-5(4*H*)-yl)ethan-1-one (**10**)

The Boc-protected **10** precursor **10a** was prepared following general procedure A from *N*-(*tert*-butoxycarbonyl)-l-phenylglycine (0.26 mmol, 65 mg) and compound **4** (0.23 mmol, 50 mg). The crude product was purified by column chromatography to give compound **10a** as a colorless oil (0.20 mmol, 91 mg, 87%). *R*_*f*_ = 0.31 (*n*-hexane/EtOAc 8:2). ^1^H NMR (400 MHz, CDCl_3_) δ (ppm): 7.40–7.38 (m, 1H), 7.33–7.21 (m, 8H), 7.14 (d, ^3^*J* = 5.1 Hz, 1H), 7.07 (d, ^3^*J* = 5.1 Hz, 1H), 6.91 (s, 1H), 6.88 (s, 1H), 6.70 (d, ^3^*J* = 5.2 Hz, 1H), 6.66 (d, ^3^*J* = 5.2 Hz, 1H), 6.28 (d, ^3^*J* = 7.3 Hz, 1H), 6.01 (d, ^3^*J* = 8.0 Hz, 1H), 5.62 (d, ^3^*J* = 8.0 Hz, 1H), 5.57 (d, ^3^*J* = 7.3 Hz, 1H), 3.86 (dd, ^2^*J* = 14.0 Hz, ^3^*J* = 5.2 Hz, 1H), 3.79 (dd, ^2^*J* = 14.0 Hz, ^3^*J* = 5.2 Hz, 1H), 3.31–3.24 (m, 1H), 3.17–3.08 (m, 1H), 2.99 (td, ^2^*J* = 13.0 Hz, ^3^*J* = 12.2 Hz, ^3^*J* = 3.4 Hz, 1H), 2.84 (dd,^2^*J* = 15.7 Hz, ^3^*J* = 2.7 Hz, 1H), 2.42 (dd, ^2^*J* = 15.7 Hz, ^3^*J* = 3.4 Hz), 1.65–1.56 (m, 1H), 1.44–1.36 (m, 9H).

Compound **10** was obtained from **10a** (0.17 mmol, 75 mg) following general procedure B followed by column chromatography (EtOAc/NEt_3_ 99:1) as an orange oil (0.13 mmol, 51 mg, 76%). ^1^H NMR (400 MHz, CDCl_3_) δ (ppm): 7.72–7.70 (m, 1H), 7.54–7.51 (m, 1H), 7.32–7.14 (m, 8H), 7.11 (d, ^3^*J* = 5.1 Hz, 1H), 7.08 (d, ^3^*J* = 4.9 Hz, 1H), 6.92 (s, 1H) 6.81–6.76 (m, 1H), 6.71 (d, ^3^*J* = 5.1 Hz, 1H), 6.67 (d, ^3^*J* = 4.9 Hz, 1H), 6.31 (d, ^3^*J* = 4.9 Hz, 1H), 5.77 (s, 1H), 5.04 (s, 1H), 4.84–4.78 (m, 1H), 3.80–3.65 (m, 1H), 3.59–3.51 (m, 1H), 3.43 (s, 1H), 3.25–3.19 (m, 1H), 3.09–2.92 (m, 3H), 2.83–2.79 (m, 1H), 2.42–2.37 (m, 1H), 1.67–1.61 (m, 1H), 1.44–1.38 (m, 1H). ^13^C NMR (101 MHz, CDCl_3_) δ (ppm): 171.1, 168.7, 168.6, 155.1, 154.9, 140.6, 140.4, 138.5, 137.9, 133.9, 133.7, 133.6, 133.4, 129.6, 128.9, 128.8, 128.7, 128.6, 128.4, 128.3, 128.3, 128.1, 128.0, 127.9, 127.8, 127.8, 127.5, 127.2, 126.4, 126.4, 125.6, 123.5, 123.2, 122.9, 79.8, 79.7, 60.4, 57.5, 57.5, 56.0, 55.8, 55.3, 54.6, 54.4, 39.4, 39.3, 36.8, 36.8, 28.4, 28.4, 25.8, 24.3, 21.1, 14.2. HRMS (ESI) *m*/*z*: calculated for C_21_H_21_N_2_OS [M + H]^+^: 349.1375; found, 349.1386.

##### (2*S*)-2-Amino-3-phenyl-1-(4-phenyl-6,7-dihydrothieno[3,2-*c*]pyridin-5(4*H*)-yl)propan-1-one (**11**)

The Boc-protected **11** precursor **11a** was prepared following general procedure A from *N*-(*tert*-butoxycarbonyl)-l-phenylalanine (0.26 mmol, 67 mg) and compound **4** (0.23 mmol, 50 mg; synthesis previously reported^38^). The crude product was purified by column chromatography to give compound **11a** as a colorless oil (0.20 mmol, 93 mg, 87%). *R*_*f*_ = 0.41 (*n*-hexane/EtOAc 8:2). ^1^H NMR (400 MHz, CDCl_3_) δ (ppm): 7.33–7.32 (m, 1H), 7.26–7.21 (m, 5H), 7.14–7.10 (m, 5H), 6.95 (d, ^3^*J* = 7.3 Hz, 1H), 6.85 (s, 1H), 6.82 (s, 1H), 6.68 (d, ^3^*J* = 5.2 Hz, 1H), 6.63 (d, ^3^*J* = 5.2 Hz, 1H), 5.55–5.45 (m, 1H), 4.85–4.79 (m, 1H), 3.88 (dd, ^2^*J* = 14.2 Hz, ^3^*J* = 5.4 Hz, 1H), 3.53–3.47 (m, 1H), 3.25 (m, 1H), 3.14–3.08 (m, 1H), 2.99–2.95 (m, 1H), 2.74 (s, 1H), 2.55–2.50 (m, 1H), 1.78–1.70 (m, 1H), 1.49 (s, 1H), 1.49–1.41 (m, 9H), 1.31–1.24 (m, 3H).

Compound **11** was obtained from **11a** (0.17 mmol, 80 mg) following general procedure B followed by column chromatography (EtOAc/NEt_3_ 99:1) as an orange oil (0.12 mmol, 43 mg, 69%). ^1^H NMR (400 MHz, CDCl_3_) δ (ppm): 7.31–6.96 (m, 10H), 6.89 (d, ^3^*J* = 4.8 Hz, 1H), 6.66 (dd, ^3^*J* = 10.9 Hz, ^3^*J* = 5.1 Hz, 1H), 6.49 (d, ^3^*J* = 5.1 Hz, 1H), 6.19 (s, 1H), 5.61 (s, 1H), 5.28 (s, 1H), 4.85–4.74 (m, 1H), 4.18 (s, 1H), 3.94 (s, 1H), 3.85 (dd, ^2^*J* = 14.3 Hz, ^3^*J* = 5.3 Hz, 1H), 3.65 (s, 1H), 3.52 (dd, ^2^*J* = 13.7 Hz, ^3^*J* = 4.4 Hz, 1H), 3.31–3.24 (m, 1H), 3.07–2.74 (m, 3H), 2.65 (dd, ^2^*J* = 16.5 Hz, ^3^*J* = 3.5 Hz, 1H), 2.16–2.06 (m, 1H), 1.88 (m, 2H). ^13^C NMR (101 MHz, CDCl_3_) δ (ppm): 173.6, 173.2, 140.9, 140.7, 137.7, 137.1, 134.0, 133.9, 133.8, 133.6, 129.3, 129.3, 128.9, 128.7, 128.7, 128.6, 128.4, 128.3, 128.2, 127.8, 127.8, 127.3, 126.8, 126.6, 126.5, 123.3, 123.2, 54.2, 54.1, 53.4, 53.2, 43.1, 39.2, 38.4, 25.9, 25.4. HRMS (ESI) *m*/*z*: calculated for C_22_H_23_N_2_OS [M + H]^+^: 363.1531; found, 363.1550.

##### 2-(Allylamino)-1-(4-phenyl-6,7-dihydrothieno[3,2-*c*]pyridin-5(4*H*)-yl)ethan-1-one (**12**)

The Boc-protected **12** precursor **12a** was prepared following general procedure A from *N*-allyl-*n*-(*tert*-butoxycarbonyl)glycine (0.42 mmol, 100 mg; synthesis previously reported^38^) and compound **4** (0.42 mmol, 100 mg; synthesis previously reported^38^). The crude product was purified by column chromatography to give compound **12a** as a colorless oil (0.41 mmol, 170 mg, 98%). *R*_*f*_ = 0.41 (*n*-hexane/EtOAc 7:3). ^1^H NMR (400 MHz, CDCl_3_) δ (ppm): 7.28 (s, 5H), 7.14 (t, ^3^*J* = 5.3 Hz, 1H), 6.86–6.84 (m, 1H), 6.72–6.69 (m, 1H), 5.97 (s, 1H), 5.78–5.74 (m, 1H), 5.15–5.01 (m, 2H), 4.83–4.79 (m, 1H), 4.46–4.42 (m, 1H), 4.28 (d, ^2^*J* = 15.9 Hz, 1H), 4.03–3.76 (m, 4H), 3.35–3.30 (m, 1H), 3.04–2.85 (m, 2H), 1.46–1.34 (m, 9H).

Compound **12** was obtained from **12a** (0.29 mmol, 120 mg) following general procedure B followed by column chromatography (EtOAc/NEt_3_ 99:1) as an orange oil (0.19 mmol, 60 mg, 65%). ^1^H NMR (400 MHz, CDCl_3_) δ (ppm): 7.28 (s, 5H), 7.16 (d, ^3^*J* = 5.2 Hz, 1H), 6.89 (s, 1H), 6.71 (d, ^3^*J* = 5.2 Hz, 1H), 5.94–5.84 (m, 1H), 5.20 (d, ^3^*J* = 17.3 Hz, 1H), 5.11 (d, ^3^*J* = 10.0 Hz, 1H), 4.86 (s, 1H), 3.78 (dd, ^2^*J* = 14.1 Hz, ^3^*J* = 5.2 Hz, 1H), 3.49 (d, ^3^*J* = 3.6 Hz, 2H), 3.36–3.29 (m, 3H), 3.05–2.96 (m, 1H), 2.90 (dd, ^2^*J* = 16.2 Hz, ^3^*J* = 3.6 Hz, 1H), 2.12 (2H). ^13^C NMR (101 MHz, CDCl_3_) δ (ppm): 140.9, 136.2, 128.6, 128.4, 127.8, 126.6, 123.4, 116.7, 110.0, 54.0, 52.2, 49.7, 38.5, 25.8, 3.3. HRMS (ESI) *m*/*z*: calculated for C_18_H_21_N_2_OS [M + H]^+^: 313.1375; found, 313.1374.

##### 1-(4-Phenyl-6,7-dihydrothieno[3,2-*c*]pyridin-5(4*H*)-yl)octan-1-one (**13**)

Compound **13** was prepared following general procedure A from octanoic acid (0.14 mmol, 14 mg) and compound **4** (0.09 mmol, 20 mg). The crude product was purified by column chromatography eluting with *n*-hexane/EtOAc 7:3 to give compound **13** as a colorless oil (0.047 mmol, 16 mg, 52%). *R*_*f*_ = 0.4 (*n*-hexane/EtOAc 9:1). ^1^H NMR (400 MHz, CDCl_3_) δ (ppm): 7.34–7.23 (m, 5H), 7.14 (d, ^3^*J* = 5.1 Hz, 1H), 6.93 (s, 1H), 6.79 (d, ^3^*J* = 5.1 Hz, 1H), 6.71 (d, ^3^*J* = 5.1 Hz, 1H), 6.01 (s, 1H), 4.87 (d, ^3^*J* = 8.6 Hz, 1H), 3.89 (dd, ^2^*J* = 14.0 Hz, ^3^*J* = 5.0 Hz, 1H), 3.33 (td, ^2^*J* = 14.0, ^3^*J* = 13.0, 4.3 Hz, 1H), 3.03–2.82 (m, 2H), 2.60–2.47 (m, 2H), 2.43–2.26 (m, 2H), 1.68–1.64 (m, 2H), 1.38–1.19 (m, 9H), 0.88–0.85 (m, 3H). ^13^C NMR (101 MHz, CDCl_3_) δ (ppm): 171.5, 141.4, 134.4, 133.9, 128.6, 128.3, 127.6, 127.3, 126.7, 123.1, 53.4, 39.4, 33.8, 31.7, 29.4, 29.2, 29.1, 25.9, 25.3, 22.6, 14.1. HRMS (ESI) *m*/*z*: calculated for C_21_H_28_NOS [M + H]^+^: 342.1886; found, 342.1886.

##### 2-(2-Methylbutylamino)-1-[4-(pyridin-2-yl)-6,7-dihydro-4*H*-thieno[3,2-*c*]pyridin-5-yl]ethanone (**19**)

The boc-protected **19** was prepared following general procedure A from [(*N*-tert-butoxycarbonyl)(2-methylbutyl)amino]acetic acid (0.23 mmol, 60 mg) and 2-{4*H*,5*H*,6*H*,7*H*-thieno[3,2-*c*]pyridin-4-yl}pyridine (0.23 mmol, 50 mg). The crude product was purified by column chromatography eluting with *n*-hexane/EtOAc 7:3 to give product as a yellow oil (0.088 mmol, 39 mg, 38%). *R*_*f*_ = 0.7 (*n*-hexane/EtOAc 7:3). ^1^H NMR (400 MHz, CDCl_3_) δ (ppm): 8.59–8.50 (m, 1H), 7.67–7.60 (m, 1H), 7.52–7.47 (m, 1H), 7.21–7.05 (m, 2H), 6.87–6.84 (m, 1H), 6.72 (s, H), 6.61 (s, 1H), 6.05–5.99 (m, 1H), 4.97–4.88 (m, 1H), 4.64–4.23 (m, 1H), 4.06–3.84 (m, 2H), 3.33–2.80 (m, 4H), 1.66–1.58 (m, 1H), 1.49–1.28 (m, 9H), 1.11–1.04 (m, 1H), 0.90–0.83 (m, 6H).

Compound **19** was obtained from the boc-protected intermediate (0.089 mmol, 39 mg) according to general procedure C as an orange oil (0.043 mmol, 15 mg, 48%) and used without further purification. ^1^H NMR (400 MHz, CDCl_3_) δ (ppm): 8.59 (d, ^3^*J* = 4.1 Hz, 1H), 8.52 (d, ^3^*J* = 4.1 Hz, 1H), 7.66–7.61 (m, 1H), 7.45 (d, ^3^*J* = 7.9 Hz, 1H), 7.20 (dd, ^3^*J* = 7.9, 5.2 Hz, 1H), 7.17–7.14 (m, 1H), 7.10 (d, ^3^*J* = 5.2 Hz, 1H), 7.07 (d, ^3^*J* = 7.9 Hz, 1H), 6.90 (d, ^3^*J* = 5.2 Hz, 1H), 6.75 (d, ^3^*J* = 5.2 Hz, 1H), 6.66 (s, 1H), 6.04 (s, 1H), 4.97 (dd, ^2^*J* = 12.8 Hz, ^3^*J* = 4.4 Hz, 1H), 4.04–3.94 (m, 1H), 3.97 (dd, ^2^*J* = 14.5 Hz, ^3^*J* = 4.4 Hz, 1H), 3.92–3.83 (m, 1H), 3.68 (dd, ^2^*J* = 16.0 Hz, ^3^*J* = 3.6 Hz, 1H), 3.55–3.49 (m, 1H), 3.11–2.94 (m, 2H), 2.87 (dd, ^2^*J* = 16.0 Hz, ^3^*J* = 2.6 Hz, 1H), 2.56–2.49 (m, 1H), 2.43–2.36 (m, 1H), 2,23 (s, 1H), 1.56–1.49 (m, 1H), 1.46–1.38 (m, 1H), 1.17–1.10 (m, 1H), 0.92–0.85 (m, 6H). ^13^C NMR (101 MHz, CDCl_3_) δ (ppm): 171.3, 167.0, 159.7, 159.5, 149.7, 149.6, 136.8, 136.6, 135.5, 133.7, 133.3, 132.2, 126.2, 126.2, 123.4, 123.1, 122.9, 122.8, 122.5, 121.2, 58.8, 56.3, 56.2, 56.1, 56.1, 51.5, 51.4, 51.3, 40.2, 37.0, 35.1, 35.1, 34.9, 27.4, 27.4, 27.3, 25.6, 24.8, 17.6, 17.6, 11.3, 11.3, 11.2. HRMS (ESI): calculated for C_19_H_26_N_3_OS [M + H]^+^: 344.1797; found, 344.1786.

##### (*S*)-1-(4-(6-Methylpyridin-2-yl)-6,7-dihydrothieno[3,2-*c*]pyridin-5(4*H*)-yl)ethan-1-one (**27**)

Compound **27** was obtained from compound **21** via preparative chiral HPLC (method B). Chiral HPLC (method A): 11.8 min (100%). [α]^23^_D_ (*c* = 0.1, CHCl_3_): −288. ^1^H NMR (500 MHz, CDCl_3_) δ (ppm): 7.53 (t, ^3^*J* = 7.6 Hz, 1H), 7.20–7.15 (m, 1H), 7.13–7.07 (m, 1H), 7.06–6.96 (m, 1H), 6.94–6.83 (m, 1H), 6.67 (s, 0.4H), 6.06 (s, 1H), 5.05–4.97 (m, 1H), 4.05 (s, 1H), 3.10–2.84 (m, 3H), 2.59 (s, 2H), 2.55 (s, 1H), 2.38 (s, 2H), 2.25 (s, 1H). ^13^C NMR (125 MHz, CDCl_3_) δ (ppm): 170.8, 159.0, 158.5, 135.5, 132.8, 126.7, 126.4, 123.2, 122.9, 122.3, 117.9, 77.3, 77.0, 76.7, 60.5, 55.8, 42.5, 36.5, 29.7, 25.6, 24.9, 24.5, 22.6, 22.2. HRMS (ESI): calculated for C_15_H_17_N_2_O [M + H^+^]: 273.1062; found, 273.1069.

##### (*R*)-1-(4-(6-Methylpyridin-2-yl)-6,7-dihydrothieno[3,2-*c*]pyridin-5(4*H*)-yl)ethan-1-one (**29**)

Compound **29** was obtained from compound **21** via preparative chiral HPLC (method B). Chiral HPLC (method A): 10.9 min (100%). [α]^23^_D_ (*c* = 0.1, CHCl_3_): +118.4. ^1^H NMR (500 MHz, CDCl_3_) δ (ppm): 7.60–7.50 (m, 1H), 7.22–7.13 (m, 1H), 7.13–7.07 (m, 1H), 7.06–6.97 (m, 1H), 6.95–6.85 (m, 1H), 6.66 (s, 1H), 6.06 (s, 1H), 5.05–4.98 (m, 1H), 4.14–4.00 (m, 1H), 3.10–2.84 (m, 3H), 2.59 (s, 2H), 2.54 (s, 1H), 2.38 (s, 2H), 2.25 (s, 1H). ^13^C NMR (125 MHz, CDCl_3_) δ (ppm): 170.8, 159.0, 158.5, 135.5, 133.2, 132.8, 126.7, 126.4, 123.2, 122.9, 122.3, 119.0, 117.9, 60.5, 55.9, 42.5, 36.6, 29.4, 25.7, 24.9, 24.5, 22.7, 22.6, 22.2, 14.1. HRMS (ESI): calculated for: C_15_H_17_N_2_O [M + H]^+^: 273.1062; found, 273.1058.

##### 1-(4-(2-Methoxyphenyl)-6,7-dihydrothieno[3,2-*c*]pyridin-5(4*H*)-yl)-2-((2-methylbutyl)amino)ethan-1-one (**32**)

The Boc-protected **32** precursor **32a** was prepared following general procedure A from [(*N*-*tert*-butoxycarbonyl)(2-methylbutyl)amino]acetic acid (0.29 mmol, 76 mg) and 4-(2-methoxyphenyl)-4*H*,5*H*,6*H*,7*H*-thieno[3,2-*c*]pyridine (0.29 mmol, 70 mg).^38^ The crude product was purified by column chromatography to give compound **32a** as a yellow oil (0.068 mmol, 32 mg, 23%). *R*_*f*_ = 0.48 (*n*-hexane/EtOAc 7:3). ^1^H NMR (400 MHz, CDCl_3_) δ (ppm): 7.28–7.24 (m, 1H), 7.09–7.07 (m, 1H), 6.99–6.81 (m, 3H), 6.67–6.61 (m, 1H), 6.26 (d, ^3^*J* = 5.7 Hz, 1H), 4.74 (d, ^3^*J* = 7.8 Hz, 1H), 4.57–4.17 (m, 1H), 4.03–3.93 (m, 2H), 3.85–3.72 (m, 1H), 3.54–3.19 (m, 1H), 3.12–2.96 (m, 2H), 2.85–2.78 (m, 1H), 1.77 (s, 1H), 1.62–1.50 (m, 1H), 1.50–1.41 (m, 6H), 1.30–1.23 (m, 5H), 1.14–0.98 (m, 1H), 0.90–0.76 (m, 6H).

Compound **32** was obtained from **32a** (0.068 mmol, 32 mg) following general procedure C as an orange oil (0.056 mmol, 21 mg, 83%) and used without further purification. ^1^H NMR (500 MHz, CDCl_3_) δ (ppm): 7.29–7.23 (m, 2H), 7.10 (d, ^3^*J* = 5.2 Hz, 1H), 7.07 (d, ^3^*J* = 4.5 Hz, 1H), 7.01 (s, 1H), 6.93 (d, ^3^*J* = 8.2 Hz, 1H), 6.89 (d, ^3^*J* = 7.3 Hz, 1H), 6.85–6.84 (m, 2H), 6.67 (d, ^3^*J* = 4.5 Hz, 1H), 6.64 (d, ^3^*J* = 5.2 Hz, 1H), 6.27 (s, 1H), 4.78 (dd, ^2^*J* = 11.9 Hz, ^3^*J* = 4.5 Hz, 1H), 3.92 (s, 3H), 3.88 (s, 1H), 3.83 (s, 3H), 3.77 (dd, ^2^*J* = 16.2 Hz, ^3^*J* = 3.3 Hz, 1H), 3.62 (dd, ^2^*J* = 16.2 Hz, ^3^*J* = 4.3 Hz, 1H), 3.54–3.43 (m, 1H), 3.09 (dd, ^2^*J* = 12.0 Hz, ^3^*J* = 3.8 Hz, 1H), 3.05 (dd, ^2^*J* = 9.4 Hz, ^3^*J* = 3.8 Hz, 1H), 3.00 (dd, ^2^*J* = 12 Hz, ^3^*J* = 5.2 Hz, 1H), 2.91–2.88 (m, 1H), 2.83 (dd, ^2^*J* = 16.2 Hz, ^3^*J* = 3.8 Hz, 1H), 2.51–2.43 (m, 1h), 2.38–2.32 (m, 1H), 1.85 (s, 2H), 1.54–1.48 (m, 1H), 1.46–1.38 (m, 1H), 1.16–1.10 (m, 1H), 0.91–0.85 (m, 6H). ^13^C NMR (125 MHz, CDCl_3_) δ (ppm): 171.6, 169.7, 156.7, 135.7, 135.3, 134.1, 129.9, 129.7, 129.6, 129.1, 128.7, 126.6, 125.9, 123.4, 123.0, 120.6, 120.1, 111.0, 110.6, 56.5, 56.4, 55.7, 55.4, 51.9, 51.6, 50.7, 50.3, 39.5, 36.9, 35.3, 29.8, 27.6, 27.6, 26.0, 24.8, 17.8, 11.5, 11.4, 1.2. HRMS (ESI): calculated for C_21_H_29_N_2_O_2_S [M + H]^+^: 373.1950; found, 373.1949.

##### 1-(4-(2-Hydroxyphenyl)-6,7-dihydrothieno[3,2-*c*]pyridin-5(4*H*)-yl)-2-((2-methylbutyl)amino)ethan-1-one (**34**)

BBr_3_ (1.1 mL of 1 M BBr_3_ in DCM, 1.08 mmol, 9 equiv) was added dropwise to a solution of **32a** (0.12 mmol, 58 mg, 1 equiv) in 2 mL dry DCM at −78 °C (2-propanol–dry ice bath) under argon atmosphere. The reaction mixture was allowed to warm to RT while stirring overnight under argon. Subsequently, a 1:1 mixture of methanol and water (1 mL) was added to quench the reaction, the organic phase was washed with brine, dried over MgSO4, and the solvent was removed in vacuum. The residual was purified by column chromatography to obtain the product (**34**) as a white solid (12 mg, 0.033 mmol, 28%). *R*_*f*_ = 0.28 (*n*-hexane/EtOAc/NEt_3_ 80:20:1). ^1^H NMR (500 MHz, CDCl_3_) δ (ppm): 8.59 (d, ^3^*J* = 4.1 Hz, 1H), 8.52 (d, ^3^*J* = 4.1 Hz, 1H), 7.66–7.61 (m, 1H), 7.45 (d, ^3^*J* = 7.8 Hz, 1H), 7.21–7.06 (m, 2H), 6.90 (d, ^3^*J* = 5.2 Hz, 1H), 6.75 (d, ^3^*J* = 5.2 Hz, 1H), 6.66 (s, 1H), 6.04 (s, 1H), 4.97 (dd, ^2^*J* = 12.8 Hz, ^3^*J* = 4.4 Hz, 1H), 3.96 (d, ^3^*J* = 3.6 Hz, 1H), 3.92–3.83 (m, 1H), 3.68 (dd, ^2^*J* = 16.0 Hz, ^3^*J* = 3.6 Hz, 1H), 3.58–3.49 (m, 1H), 3.11–2.94 (m, 2H), 2.87(dd, ^2^*J* = 16.0 Hz, ^3^*J* = 2.6 Hz, 1H), 2.56–2.49 (m, 1H), 2.43–2.36 (m, 1H), 2.23 (s, 1H), 1.56–1.49 (m, 1H), 1.46–1.38 (m, 1H), 1.17–1.10 (m, 1H), 0.92–0.86 (m, 6H). ^13^C NMR (125 MHz, CDCl_3_) δ (ppm): 171.3, 170.0, 159.7, 159.5, 149.7, 149.6, 136.8, 136.6, 135.5, 133.7, 133.3, 132.2, 126.2, 126.2, 123.4, 123.1, 122.9, 122.8, 122.5, 121.2, 58.8, 56.3, 56.2, 56.1, 56.1, 51.5, 51.4, 51.3, 40.2, 37.0, 35.1, 35.1, 34.9, 27.4, 27.4, 27.3, 25.6, 24.8, 17.6, 17.6, 11.3, 11.3, 11.2. HRMS (ESI): calculated for C_20_H_27_N_2_O_2_S [M + H]^+^: 359.1793; found, 359.1787.

##### 1-(4-Cyclohexyl-6,7-dihydrothieno[3,2-*c*]pyridin-5(4*H*)-yl)-2-((2-methylbutyl)amino)ethan-1-one (**36**)

The Boc-protected **36** precursor **36a** was prepared following general procedure A from [(*N*-*tert*-butoxycarbonyl)(2-methylbutyl)amino]acetic acid (0.23 mmol, 55 mg) and 4-cyclohexyl-4,5,6,7-tetrahydrothieno[3,2-*c*]pyridine (0.23 mmol, 50 mg).^38^ The crude product was purified by column chromatography to give compound **36a** as a colorless oil (0.069 mmol, 31 mg, 30%). *R*_*f*_ = 0.40 (*n*-hexane/EtOAc 8:2). ^1^H NMR (400 MHz, CDCl_3_) δ (ppm): 7.09–7.05 (m, 1H), 6.82–6.75 (m, 1H), 5.34 (d, ^3^*J* = 7.9 Hz, 1H), 4.86–4.83 (m, 1H), 4.39–3.99 (m, 3H), 3.91–3.83 (m, 1H), 3.56–3.46 (m, 1H), 3.31–3.22 (m, 1H), 3.15–2.72 (m, 4H), 1.88 (s, 1H), 1.78–1.62 (m, 6H), 1.45–1.34 (m, 9H), 1.25–0.97 (m, 6H), 0.89–0.78 (m, 6H).

Compound **36** was obtained from **36a** (0.085 mmol, 38 mg) according to general procedure C as an orange oil (0.055 mmol, 19 mg, 65%) and used without further purification. ^1^H NMR (500 MHz, CDCl_3_) δ (ppm): 7.07 (t, ^3^*J* = 5.7 Hz, 1H), 6.82 (d, ^3^*J* = 5.2 Hz, 1H), 6.78 (d, ^3^*J* = 5.2 Hz, 1H), 5.36 (d, ^3^*J* = 8.5 Hz, 1H), 4.86 (dd, ^2^*J* = 13.1 Hz, ^3^*J* = 6.3 Hz, 1H), 4.30 (d, ^3^*J* = 9.0 Hz, 1H), 3.91 (dd, ^2^*J* = 14.1 Hz, ^3^*J* = 5.8 Hz, 1H), 3.60 (dd, ^2^*J* = 15.7 Hz, ^3^*J* = 7.8 Hz, 1H), 3.53–3.46 (m, 1H), 3.44–3.37 (m, 1H), 3.10 (td, ^2^*J* = 12.5, ^3^*J* = 4.8 Hz, 1H), 2.99–2.90 (m, 1H), 2.83 (dd, ^2^*J* = 16.1 Hz, ^3^*J* = 3.9 Hz, 1H), 2.76 (dd, ^2^*J* = 16.3 Hz, ^3^*J* = 4.4 Hz, 1H), 2.55–2.46 (m, 1H), 2.42–2.33 (m, 1H), 2.21 (s, 1H), 1.90–1.88 (m, 1H), 1.83–1.60 (m, 5H), 1–58–1.51 (m, 1H), 1.47–1.38 (m, 1H), 1.23–1.10 (m, 6H), 0.93–0.86 (m, 6H). ^13^C NMR (125 MHz, CDCl_3_) δ (ppm): 170.5, 170.0, 135.9, 135.0, 134.6, 132.6, 127.4, 126.8, 122.2, 122.1, 59.3, 56.1, 56.5, 56.4, 56.4, 56.0, 51.2, 51.2, 51.1, 43.0, 42.7, 39.3, 36.2, 35.2, 35.1, 35.1, 30.9, 30.7, 30.5, 29.8, 27.6, 27.5, 27.5, 26.4, 26.4, 26.3, 26.3, 26.2, 25.8, 24.6, 17.8, 17.8, 11.5, 11.4, 11.4, 1.2. HRMS (ESI): calculated for C_20_H_33_N_2_OS [M + H]^+^: 349.2314; found, 349.2320.

##### 1-(1-Phenyl-3,4-dihydroisoquinolin-2(1*H*)-yl)ethan-1-one (**37**)

Compound **37** was obtained from triethylamine (1.44 mmol, 146 mg), 1-phenyl-1,2,3,4-tetrahydro-isoquinoline (0.24 mmol, 50 mg) and acetyl chloride (0.72 mmol, 57 mg) according to general procedure D as colorless oil (0.10 mmol, 26 mg, 43%). ^1^H NMR (500 MHz, CDCl_3_) δ (ppm): 7.31–7.17 (m, 8H), 7.07 (d. ^3^*J* = 7.6 Hz, 1H), 6.95 (s, 1H), 5.97 (s, 1H), 4.20–4.15 (m, 1H), 3.73–3.72 (m, 1H), 3.48–3.38 (m, 1H), 3.06–2.99 (m, 1H), 2.96–2.92 (m, 1H), 2.90–2.85 (m, 1H), 2.76–2.71 (m, 1H), 2.31 (s, 3H), 2.18 (s, 3H). ^13^C NMR (125 MHz, CDCl_3_) δ (ppm): 169.9, 169.1, 162.5, 142.3, 141.1, 135.4, 135.3, 135.2, 134.3, 128.9, 128.8, 128.6, 128.6, 128.5, 128.2, 127.9, 127.6, 127.6, 127.3, 127.1, 127.0, 126.3, 126.2, 60.8, 54.9, 40.5, 37.9, 28.8, 27.7, 21.9, 21.7. HRMS (ESI): calculated for C_17_H_18_NO [M + H]^+^: 252.1388; found, 252.1392.

##### 2-((2-Methylbutyl)amino)-1-(1-phenyl-3,4-dihydroisoquinolin-2(1*H*)-yl)ethan-1-one (**38**)

The Boc-protected **38** precursor **38a** was prepared following general procedure A from [(*N*-*tert*-butoxycarbonyl)(2-methylbutyl)amino]acetic acid (0.089 mmol, 22 mg) and 1-phenyl-1,2,3,4-tetrahydroisoquinoline (0.089 mmol, 19 mg). The crude product was purified by column chromatography to give compound **38a** as a colorless oil (0.040 mmol, 17 mg, 45%). ^1^H NMR (400 MHz, CDCl_3_) δ (ppm): 7.30–7.17 (m, 8H), 7.10–7.06 (m, 1H), 6.91–6.88 (m, 1H), 6.02–5.89 (m, 1H), 4.47–4.38 (m, 1H), 4.27–3.65 (m, 3H), 3.51–2.74 (m, 5H), 1.79–2.58 M, 1H), 1.47–1.33 (m, 10H), 1.22–1.16 (m, 1H), 1.13–1.03 (m, 1H), 0.91–0.79 (m, 6H).

Compound **38** was obtained from **38a** (0.040 mmol, 17 mg) following general procedure C as an orange oil (0.028 mmol, 10 mg, 70%) and used without further purification. ^1^H NMR (500 MHz, CDCl_3_) δ (ppm): 7.31–7.08 (m, 8H), 7.09 (d, ^3^*J* = 7.4 Hz, 1H), 6.90 (s, 1H), 5.96 (s, 1H), 4.28–4.24 (m, 1H), 3.69–3.42 (m, 4H), 3.01–2.96 (m, 1H), 2.83 (dt, ^3^*J* = 16.2, 4.1 Hz, 1H), 2.76–2.71 (m, 1H), 2–59–2.54 (m, 2H), 2.46–2.42 (m, 1H), 1.58–1.55 (m, 1H), 1.46–1.43 (m, 1H), 1.21–1.14 (m, 1H), 0.95–0.87 (m, 6H). ^13^C NMR (125 MHz, CDCl_3_) δ (ppm): 169.5, 142.2, 135.3, 134.4, 129.1, 129.0, 128.8, 128.7, 128.4, 128.1, 127.8, 127.5, 127.3, 127.3, 126.5, 126.4, 77.4, 77.2, 76.9, 59.1, 56.4, 55.7, 51.3, 51.1, 39.0, 38.5, 35.1, 35.0, 28.9, 27.8, 27.6, 27.5, 17.7, 11.4, 11.4. HRMS (ESI): calculated for C_22_H_29_N_2_O [M + H]^+^: 337.2280; found, 337.2278.

##### 2-((2-Methylpropyl)amino)-1-(1-phenyl-3,4-dihydroisoquinolin-2(1*H*)-yl)ethan-1-one (**39**)

The Boc-protected **39** precursor **39a** was prepared following general procedure A from [(*N*-*tert*-butoxycarbonyl)(2-methylpropyl)amino]acetic acid (0.116 mmol, 27 mg) and 1-phenyl-1,2,3,4-tetrahydroisoquinoline (0.14 mmol, 30 mg). The crude product was purified by column chromatography to give compound **39a** as a colorless oil (0.11 mmol, 30 mg, 97%). ^1^H NMR (400 MHz, CDCl_3_) δ (ppm): 7.33–7.10 (m, 7H), 7.08 (s, 1H), 6.89 (d, ^3^*J* = 9.3 Hz, 1H), 4.51–3.82 (m, 2H), 3.81–3.56 (m, 1H), 3.53–3.28 (m, 1H), 3.24–2.91 (m, 3H), 2.90–2.77 (m, 1H), 1.86 (dt, ^2^*J* = 15.1 Hz, ^3^*J* = 7.6 Hz, 1H), 1.68 (s, 1H), 1.54–1.19 (m, 9H), 0.87 (s, 6H). HRMS (ESI): calculated for C_26_H_35_N_2_O_3_ [M + H]^+^: 423.2648, found: 423.2631.

Compound **39** was obtained from **39a** (0.11 mmol, 48 mg) following general procedure C as an orange oil (0.099 mmol, 32 mg, 87%) and used without further purification. ^1^H NMR (500 MHz, CDCl_3_) δ (ppm): 7.32–7.14 (m, 7H), 7.12–7.06 (m, 1H), 6.90 (s, 1H), 5.96 (s, 1H), 4.29–4.22 (m, 1H), 3.71–3.38 (m, 4H), 3.05–2.89 (m, 1H), 2.83 (dt, ^2^*J* = 16.2 Hz, ^3^*J* = 4.1 Hz, 1H), 2.77–2.70 (m, 1H), 2.49–2.40 (m, 2H), 1.82–1.75 (m, 1H), 0.97–0.90 (m, 6H). ^13^C NMR (125 MHz, CDCl_3_) δ (ppm): 169.3, 142.1, 135.3, 135.1, 134.2, 128.8, 128.6, 128.5, 128.3, 128.0, 127.7, 127.4, 127.2, 127.1, 126.4, 126.3, 159.0, 58.1, 55.6, 51.1, 50.9, 38.8, 38.3, 28.8, 28.4, 27.6, 20.6. HRMS (ESI): calculated for C_21_H_27_N_2_O [M + H]^+^: 323.2123; found. 323.2128.

##### *N*-Isopentyl-1-phenyl-3,4-dihydroisoquinoline-2(1*H*)-carboxamide (**40**)

Compound **40** was obtained from isopentylamine (0.10 mmol, 9 mg) and 1-phenyl-1,2,3,4-tetrahydro-isoquinoline (0.13 mmol, 47 mg) following general procedure E as colorless oil (0.08 mmol, 25 mg, 80%). *R*_*f*_ = 0.51 (*n*-hexane/EtOAc 7:3). ^1^H NMR (500 MHz, CDCl_3_) δ (ppm): 7.30–7.17 (m, 9H), 6.32 (s, 1H), 4.47 (s, 1H), 3.66–3.58 (m, 2H), 3.37–3.22 (m, 2H), 2.94–2.79 (m, 2H), 1.60–1.52 (m, 1H), 1.38 (d, *J*^3^ = 7.2 Hz, 2H), 0.90 (dd, *J*^3^ = 6.6 Hz, *J*^3^ = 2.3 Hz, 6H). ^13^C NMR (125 MHz, CDCl_3_) δ (ppm): 157.8, 143.1, 136.7, 135.3, 128.6, 128.5, 128.3, 127.5, 127.3, 127.2, 126.5, 57.9, 40.2, 39.4, 39.3, 28.5, 26.0, 22.6. HRMS (ESI): calculated for C_21_H_27_N_2_O [M + H]^+^: 323.2123; found, 323.2120.

##### *N*-(2-Methoxyethyl)-1-phenyl-3,4-dihydroisoquinoline-2(1*H*)-carboxamide (**41**)

Compound **41** was obtained from 2-methoxyethan-1-amine (0.14 mmol, 11 mg) and 1-phenyl-1,2,3,4-tetrahydro-isoquinoline (0.27 mmol, 100 mg) following general procedure E as colorless oil (0.14 mmol, 43 mg, 100%). *R*_*f*_ = 0.45 (*n*-hexane/EtOAc 7:3). ^1^H NMR (400 MHz, CDCl_3_) δ (ppm): 7.30–7.17 (m, 9H), 6.36 (s, 1H), 4.94 (s, 1H), 3.68–3.54 (m, 2H), 3.52–3.40 (m, 4H), 3.32 (s, 3H), 2.95–2.88 (m, 1H), 2.83–2.77 (m, 1H). ^13^C NMR (101 MHz, CDCl_3_) δ (ppm): 157.6, 142.8, 136.6, 135.1, 128.4, 128.3, 127.5, 127.1, 127.1, 126.3, 71.7, 58.7, 57.7, 40.7, 40.0, 28.3. HRMS (ESI): calculated for C_19_H_23_N_2_O_2_ [M + H]^+^: 311.1760; found, 311.1762.

##### (*S*)-2-((2-Methylpropyl)amino)-1-(1-phenyl-3,4-dihydroisoquinolin-2(1*H*)-yl)ethan-1-one (**42**)

Compound **42** was obtained from compound **39** via preparative chiral HPLC (method B). Chiral HPLC (method A): 12.5 min (100%). [α]^23^_D_ (*c* = 0.1, CHCl_3_): −129.5. ^1^H NMR (500 MHz, CDCl_3_) δ (ppm): 7.33–7.13 (m, 7H), 7.10 (d, *J*^3^ = 6.6 Hz, 1H), 6.91 (s, 1H), 5.95 (s, 1H), 4.28–4.25 (m, 1H), 3.69–3.61 (m, 2H), 3.59–3.40 (m, 4H), 3.00 (m, 1H), 2.86–2.81 (m, 1H), 2.76–3.73 (m, 1H), 2.45 (d, ^3^*J* = 6.7 Hz, 2H), 1.83–1.75 (m, 1H), 0.97–0.90 (m, 6H). ^13^C NMR (125 MHz, CDCl_3_) δ (ppm): 169.5, 142.1, 141.0, 135.3, 135.1, 134.2, 128.8, 128.6, 128.5, 128.3, 128.0, 127.7, 127.4, 127.2, 127.1, 126.4, 126.3, 59.0, 58.2, 58.1, 55.6, 51.2, 51.0, 38.8, 38.3, 29.7, 28.8, 28.5, 27.6, 20.7, 20.6. HRMS (ESI): calculated for C_21_H_27_N_2_O [M + H]^+^: 323.2123; found, 323.2131.

##### (*R*)-2-((2-Methylpropyl)amino)-1-(1-phenyl-3,4-dihydroisoquinolin-2(1*H*)-yl)ethan-1-one (**43**)

Compound **43** was obtained from compound **39** via preparative chiral HPLC (method B). Chiral HPLC (method A): 11.8 min (100%). [α]^23^_D_ (*c* = 0.1, CHCl_3_): +189.3. ^1^H NMR (500 MHz, CDCl_3_) δ (ppm): 7.33–7.14 (m, 7H), 7.13–7.07 (m, 1H), 6.91 (s, 1H), 5.95 (s, 1H), 4.32–4.21 (m, 1H), 3.71–3.37 (m, 4H), 3.05–2.91 (m, 1H), 2.89–2.84 (m, 1H), 2.78–2.71 (m, 1H), 2.48–2.40 (m, 2H), 1.86–1.78 (m, 1H), 0.97–0.90 (m, 6H). ^13^C NMR (125 MHz, CDCl_3_) δ (ppm): 170.2, 169.4, 142.1, 141.0, 135.3, 135.1, 134.2, 128.9, 128.7, 128.7, 128.6, 128.3, 128.0, 127.7, 127.4, 127.2, 127.1, 126.4, 126.3, 59.0, 58.2, 55.6, 51.2, 51.0, 38.9, 38.4, 29.7, 28.8, 28.5, 27.7, 20.7, 20.6. HRMS (ESI): calculated for C_21_H_27_N_2_O [M + H]^+^: 323.2123, found: 323.2125.

##### 1-(6,7-Dimethoxy-1-phenyl-3,4-dihydroisoquinolin-2(1*H*)-yl)-2-((2-methylbutyl)amino)ethan-1-one (**44**)

The Boc-protected **44** precursor **44a** was prepared following general procedure A from [(*N*-*tert*-butoxycarbonyl)(2-methylbutyl)amino]acetic acid (0.30 mmol, 75 mg) and 6,7-dimethoxy-1-phenyl-1,2,3,4-tetrahydroisoquinoline (0.30 mmol, 83 mg). The crude product was purified by column chromatography to give compound **44a** as a colorless oil (0.25 mmol, 125 mg, 84%). *R*_*f*_ = 0.3 (*n*-hexane/EtOAc 1:1). ^1^H NMR (400 MHz, CDCl_3_) δ (ppm): 7.33–7.23 (m, 5H), 6.86–6.83 (m, 1H), 6.67 (s, 1H), 6.54–6.52 (m, 1H), 4.31–4.23 (m, 1H), 4.07–3.93 (m, 1H), 3.90 (s, 3H), 3.82–3.65 (m, 4H), 3.43–3.33 (m, 1H), 3.20–3.12 (m, 1H), 2.96–2.91 (m, 1H), 2.76–2.71 (m, 1H), 2.05 (s, 1H), 1.68–1.63 (m, 1H), 1.45–1.35 (m, 10H), 1.12–1.06 (m, 1H), 0.91–0.84 (m, 6H). HRMS (ESI): calculated for: C_29_H_41_N_2_O_5_ [M + H]^+^: 497.3015, found: 497.3000.

Compound **44** was obtained from **44a** (0.12 mmol, 62 mg) following general procedure C as colorless oil (0.080 mmol, 32 mg, 67%) and used without further purification. ^1^H NMR (500 MHz, CDCl_3_) δ (ppm): 7.33–7.20 (m, 5H), 6.85 (s, 1H), 6.69 (s, 1H), 6.62 (s, 1H), 6.54 (s, 1H), 5.89 (s, 1H), 4.38–4.35 (m, 1H), 3.91 (s, 3H), 3.83 (s, 3H), 3.81 (s, 3H), 3.67–3.55 (m, 5H), 3.41–3.36 (m, 1H), 3.24–3.20 (m, 2H), 3.01–2.91 (m, 1H), 2.79–2.63 (m, 2H), 2.51 (dd, ^3^*J* = 11.3 Hz, ^3^*J* = 7.3 Hz, 1H), 1.66–1.62 (m, 1H), 1.50–1.45 (m, 1H), 1.23–1.18 (m, 1H), 0.98–0.89 (m, 6H). ^13^C NMR (125 MHz, CDCl_3_) δ (ppm): 169.2, 168.6, 148.6, 148.3, 147.8, 142.1, 141.2, 128.8, 128.4, 127.9, 127.7, 127.6, 126.7, 126.3, 111.6, 111.3, 111.1, 111.0, 58.7, 56.2, 56.2, 56.1, 56.0, 55.3, 51.0, 50.7, 38.7, 37.7, 34.7, 34.7, 28.5, 27.5, 27.4, 17.6, 11.4, 11.3. HRMS (ESI): calculated for C_24_H_33_N_2_O_3_ [M + H]^+^: 397.2491; found, 397.2485.

##### 1-(7-Chloro-1-phenyl-3,4-dihydroisoquinolin-2(1*H*)-yl)-2-((2-methylbutyl)amino)ethan-1-one (**45**)

The Boc-protected **45** precursor **45a** was prepared following general procedure A from [(*N*-*tert*-butoxycarbonyl)(2-methylbutyl)amino]acetic acid (0.066 mmol, 16 mg) and 7-chloro-1-phenyl-1,2,3,4-tetrahydro-isoquinoline (0.026 mmol, 12 mg). The crude product was purified by column chromatography to give compound **45a** as a colorless oil (0.040 mmol, 17 mg, 40%). *R*_*f*_ = 0.47 (*n*-hexane/EtOAc 7:3). ^1^H NMR (400 MHz, CDCl_3_) δ (ppm): 7.40–7.09 (m, 8H), 6.88 (s, 1H), 6.07 (s, 1H), 4.31–4.22 (m, 1H), 4.12–4.03 (m, 1H), 3.76–3.69 (m, 1H), 3.48–2.79 (m, 5H), 1.69–1.63 (m, 2H), 1.48–1.30 (m, 10H), 1.14–1.10 (m, 1H), 0.96–0.84 (m, 6H). ^13^C NMR (101 MHz, CDCl_3_) δ (ppm): 167.5, 141.5, 137.0, 130.0, 128.7, 128.4, 127.7, 127.4, 80.0, 55.3, 54.3, 53.8, 49.0, 38.8, 33.9, 28.4, 27.0, 26.9, 17.0, 11.3. HRMS (ESI): calculated for C_27_H_36_N_2_O_3_Cl [M + H]^+^: 471.2414; found, 471.2415.

Compound **45** was obtained from **45a** (0.026 mmol, 17 mg) according to general procedure C as colorless oil (0.024 mmol, 9 mg, 95%) and used without further purification. ^1^H NMR (500 MHz, CDCl_3_) δ (ppm): 7.35–7.29 (m, 3H), 7.26–7.18 (m, 3H), 7.15 (d, ^3^*J* = 8.2 Hz, 1H), 7.11–7.10 (m, 1H), 6.89 (s, 1H), 5.96 (s, 1H), 4.36–4.28 (m, 1H), 3.90–3.85 (m, 1H), 3.71–3.68 (m, 1H), 3.62–3.52 (m, 2H), 3.47–3.41 (m, 1H), 3.02–2.92 (m, 1H), 2.85–2.82 (m, 1H), 2.75–2.72 (m, 1H), 2.62–2.55 (m, 3H), 2.50–2.47 (m, 1H), 1.64–1.59 (m, 1H), 1.51–1.42 (m, 1H), 1.35–1.32 (m, 1H), 1.30–1.28 (m, 1H), 1.25–1.44 (m, 1H), 0.98–0.89 (m, 6H). ^13^C NMR (125 MHz, CDCl_3_) δ (ppm): 169.2, 141.5, 136.9, 132.8, 132.3, 130.2, 129.0, 128.7, 128.6, 127.9, 127.6, 127.4, 56.3, 55.5, 51.0, 38.7, 34.9, 34.9, 28.5, 27.5, 27.4, 17.7, 11.4, 11.4. HRMS (ESI): calculated for C_22_H_28_N_2_OCl [M + H]^+^: 371.1890; found, 371.1895.

##### 1-(1-Phenyl-3,4-dihydropyrrolo[1,2-*a*]pyrazin-2(1*H*)-yl)prop-2-en-1-one (**46**)

Compound **46** was obtained from acryloyl chloride (0.10 mmol, 20 mg) and 1-phenyl-1,2,3,4-tetrahydropyrrolo[1,2-*a*]pyrazine (0.12 mmol, 20 mg) following general procedure D as an orange oil (0.045 mmol, 11 mg, 44%) followed by flash column chromatography purification using gradient *n*-hexane/EtOAc elution. *R*_*f*_ = 0.20 (*n*-hexane/EtOAc 4:6). ^1^H NMR (500 MHz, (CD_3_)_2_CO, 298 K) δ (ppm): 7.33–7.27 (m, 5H), 6.98–6.94 (m, 2H), 6.73 (dd, ^3^*J* = 2.7, 1.6 Hz, 1H), 6.51 (s, 1H), 6.32 (dd, ^3^*J* = 16.6, 2.3, 1H), 6.14–6.13 (m, 1H), 5.98 (s, 1H), 5.75 (dd, ^3^*J* = 10.4, 2.3 Hz, 1H), 4.56 (s, 1H), 4.26 (s, 1H), 4.12–4.09 (m, 2H), 3.62 (s, 1H), 3.42–3.33 (m, 1H). ^13^C NMR (125 MHz, (CD_3_)_2_CO, 295 K) δ (ppm): 128.9, 128.2, 127.2, 106.6. HRMS (ESI): calculated for C_16_H_17_N_2_O [M + H]^+^: 253.1341, found: 253.1354.

##### 1-(2-Phenylpiperidin-1-yl)ethan-1-one (**47**)

Compound **47** was obtained from acetyl chloride (1.86 mmol, 146 mg, 0.14 mL) and 2-phenylpiperidine (0.62 mmol, 100 mg, 0.10 mL) following general procedure D and purified by column chromatography using an *n*-hexane/EtOAc gradient as an orange oil (0.59 mmol, 120 mg, 95%). *R*_*f*_ = 0.25 (*n*-hexane/EtOAc 4:6). ^1^H NMR (400 MHz, CH_3_OD) δ (ppm): 7.38–7.20 (m, 5H), 5.87 (s, 1H), 5.21 (s, 1H), 4.49 (d, ^3^*J* = 13.3 Hz, 1H), 3.76 (d, ^3^*J* = 13.6 Hz, 1H), 3.03 (t, ^3^*J* = 11.2 Hz, 1H), 2.64 (t, ^3^*J* = 12.1 Hz, 1H), 2.44 (d, ^3^*J* = 13.9 Hz, 1H), 2.27–2.10 (m, 3H), 2.00–1.79 (m, 2H), 1.62–1.43 (m, 4H). ^13^C NMR (101 MHz, CH_3_OD) δ (ppm): 171.7, 171.0, 138.9, 138.8, 128.7, 128.4, 126.7, 126.4, 126.3, 126.2, 125.8, 56.5, 51.2, 50.9, 43.1, 42.8, 37.9, 28.9, 28.7, 27.0, 26.9, 25.7, 24.7, 20.2, 20.0, 19.1, 19.0, 18.8. HRMS (ESI): calculated for C_13_H_18_NO [M + H]^+^: 204.1388; found, 204.1386.

##### 1-(2-Phenylpiperidin-1-yl)prop-2-en-1-one (**48**)

Compound **48** was obtained from acryloyl chloride (2.48 mmol, 224 mg, 0.20 mL) and 2-phenylpiperidine (0.62 mmol, 100 mg, 0.10 mL) following general procedure D and purified by column chromatography using an *n*-hexane/EtOAc gradient as an orange oil (0.53 mmol, 115 mg, 86%). *R*_*f*_ = 0.32 (*n*-hexane/EtOAc 4:6). ^1^H NMR (400 MHz, CDCl_3_) δ (ppm): 7.39–7.36 (m, 2H), 7.28–7.24 (m, 3H), 6.66–6.53 (m, 1H), 6.36 (d, ^3^*J* = 16.3 Hz, 1H), 6.07 (s, 1H), 5.69 (s, 1H), 5.27 (s, 1H), 4.64 (s, 1H), 3.86 (s, 1H), 3.03–2.74/m, 1H), 2.45 (d, ^3^*J* = 14.4 Hz, 1H), 1.98–1.89 (m, 1H), 1.67–1.56 (m, 4H). ^13^C NMR (101 MHz, CDCl_3_) δ (ppm): 166.5, 139.1, 128.8, 128.2, 127.7, 126.5, 55.7, 51.1, 42.4, 38.3, 29.0, 26.9, 25.2, 19.5. HRMS (ESI): calculated for C_14_H_18_NO [M + H]^+^: 216.1388; found, 216.1392.

##### 1-(2-(Pyridin-2-yl)piperidin-1-yl)prop-2-en-1-one (**49**)

Compound **49** was obtained from acryloyl chloride (1.28 mmol, 116 mg, 0.11 mL) and 2-(piperidin-2-yl)pyridine hydrochloride (0.42 mmol, 100 mg) following general procedure D and purified by column chromatography using an *n*-hexane/EtOAc gradient as an orange oil (0.23 mmol, 50 mg, 55%). *R*_*f*_ = 0.30 (*n*-hexane/EtOAc 4:6). ^1^H NMR (400 MHz, CDCl_3_) δ (ppm): 8.56 (s, 1H), 7.61 (s, 1H), 7.17–7.12 (2H), 6.67–6.29 (m, 1H), 6.30 (d, ^3^*J* = 11.6 Hz, 1H), 5.98 (s, 1H), 5.68–5.61 (m, 1H), 5.22 (s, 1H), 4.65 (s, 1H), 3.87 (d, ^3^*J* = 10.6 Hz, 1H), 3.16–3.10 (m, 1H), 2.72–2.66 (m, 2H), 1.82 (s, 1H), 1.62–1.22 (m, 4H). ^13^C NMR (101 MHz, CDCl_3_) δ (ppm): 167.3, 166.6, 159.4, 149.6, 149.1, 136.7, 128.2, 127.8, 121.7, 121.0, 110.1, 57.9, 53.3, 43.2, 38.9, 28.4, 27.1, 26.2, 25.1, 19.9. HRMS (ESI): calculated for C_13_H_17_N_2_O [M + H]^+^: 217.1341, found: 217.1349.

##### 2-((2-Methylbutyl)amino)-1-(2-(pyridin-2-yl)piperidin-1-yl)ethan-1-one (**50**)

The Boc-protected **50** precursor **50a** was prepared following general procedure A from [(*N*-*tert*-butoxycarbonyl)(2-methylbutyl)amino]acetic acid (0.21 mmol, 52 mg) and 2-(piperidin-2-yl)pyridine hydrochloride (0.21 mmol, 50 mg). The crude product was purified by column chromatography to give compound **50a** as a colorless oil (0.087 mmol, 34 mg, 42%). *R*_*f*_ = 0.29 (*n*-hexane/EtOAc 7:3). ^1^H NMR (400 MHz, CDCl_3_) δ (ppm): 8.57–8.54 (m, 1H), 7.67–7.59 (m, 1H), 7.21–7.11 (m, 2H), 5.89 (s, 1H), 5.06–4.99 (m, 1H), 4.58–4.56 (m, 1H), 4.27–4.00 (m, 2H), 3.91–3.84 (m, 1H), 3.70–3.60 (m, 1H), 3.23 (dd, ^2^*J* = 14.4 Hz, ^3^*J* = 6.6 Hz, 1H), 3.13–3.03 (m, 1H), 2.67–2.62 (m, 1H), 1.98 (s, 1H), 1.66–1.57 (m, 3H), 1.55–1.50 (m, 1H), 1.45–1.34 (m, 10H), 1.13–1.06 (m, 1H), 0.91–0.77 (m, 6H).

Compound **50** was obtained from **50a** (0.087 mmol, 34 mg) following general procedure C as an orange oil (0.059 mmol, 17 mg, 68%) and used without further purification. ^1^H NMR (400 MHz, CDCl_3_) δ (ppm): 8.57 (d, ^3^*J* = 14.4 Hz, 1H), 7.66–7.61 (m, 1H), 7.17–7.13 (m, 2H), 5.92 (s, 1H), 5.05 (s, 1H), 4.61 (d, ^3^*J* = 11.6 Hz, 1H), 3.65–3.56 (m, 2H), 3.30 (d, ^3^*J* = 14.1 Hz, 1H), 3.13 (t, ^3^*J* = 10.35 Hz, 1H), 2.70–2.39 (m, 5H), 1.89–1.82 (m, 1H), 1.66–1.32 (m, 6H), 1.18–1.13 (m, 1H), 0.92–0.89 (m, 6H). ^13^C NMR (101 MHz, CDCl_3_) δ (ppm): 170.5, 159.4, 149.7, 149.1, 136.5, 121.9, 121.6, 120.8, 56.3, 53.3, 51.0, 41.8, 39.0, 35.0, 28.3, 27.4, 27.4, 27.2, 25.9, 19.8, 17.6, 11.3. HRMS (ESI): calculated for C_17_H_28_N_3_O [M + H]^+^: 290.2232, found: 290.2245.

#### Acyl-cLIP Assay

The acyl-cLIP assay has been described previously.^40^ Briefly, SHH-FAM peptide was diluted in buffer A (100 mM MES, 20 mM NaCl, 1 mM DTT, 1 mM TCEP, 0.1% BSA, pH 6.5) to a concentration of 2 μM. Palmitoyl-CoA (Sigma-Aldrich) was diluted in buffer A to 7.5 μM and recombinant HHAT (obtained by following the published protocol^14^) in buffer B (20 mM HEPES, 350 mM NaCl, 1% DDM, 5% glycerol, pH 7.3) diluted to 44 μg/mL. 1.5 μL of a 50 mM inhibitor DMSO stock was diluted with 18.5 μL of DMSO and further serial diluted (1:1). For each condition, 6 μL of the HHAT working stock and 57 μL of the SHH-peptide working stock were mixed with 3 μL of the corresponding inhibitor concentration or DMSO vehicle. Twelve μL/well of this mixture was split into four wells of a black 384 well plate. To start the reaction, 8 μL of the Pal-CoA working stock were added to each well and fluorescence anisotropy and the total fluorescence recorded on an EnVision Xcite 2104 (PerkinElmer) over 30 min (emission filter 1 = FITC FP P-pol 535; emission filter 2 = FITC S-pol 535; excitation filter: FITC FP 480; measurement height = 6.5 mm; detector gain = 0; high concentration gain). Initial rate constants were determined by linear regression and normalized to DMSO control and samples with buffer B instead of HHAT. IC_50_ values were extracted from the corresponding dose response curves using a “sigmoidal dose response (variable slope)” model in GraphPad Prism 5 (GraphPad Software, Inc.).

#### Molecular Docking

All molecular modeling studies were performed in the software MOE version 2022 using the published cryo-EM structure of HHAT IMP-1575 complex (PDB 7Q6Z). The active site was defined using the ligand IMP-1575. Minimization was applied on both the receptor and ligands before docking using QuickPrep Panel with default values. The Triangle Matcher placing method and Rigid Receptor refinement method were employed, and docking results were scored with London dG and GBVI/WSA dG. The top 5 poses from a total of 100 docked poses were selected for analysis. The 2D ligand interactions images were generated with the following cutoffs: H-bond is < −0.5 kcal/mol; ionic is < −0.5 kcal/mol; maximum distance cutoff is <4.0 Å. One of the top-scored poses was selected for image representation.

#### Cell Lines and Tissue Culture Reagents

HEK293a cells stably transfected with SHH (HEK293a *SHH*^*+*^) were maintained in DMEM supplemented with 10% FBS.^35^ SHH-Light2 cells were a generous gift from Prof. James K. Chen (Stanford University, USA) and were maintained in high glucose and sodium pyruvate containing DMEM (GIBCO), supplemented with iron fortified calf serum (ATCC), 400 μg/mL G418 (Geneticin) and 150 μg/mL Zeocin (Invitrogen). Both cell lines were cultured at 37 °C and 5% CO_2._

#### MTS Assay

HEK293a *SHH*^*+*^ cells were plated at 5000 cells/well in a 96-well plate (Nunc) using a volume of 50 μL/well and cultured for 24 h as described. Cells were treated with 100 μL/well of either DMSO vehicle, Puromycin (2 μg/mL), or stated analogues (0.14–100 μM final) in DMEM supplemented with 10% FBS. After 72 h, 20 μL/well of a mixture of 1-methoxy phenazine methosulfate (PMS) and [3-(4,5-dimethylthiazol-2-yl)-5-(3-carboxymethoxyphenyl)-2-(4-sulfophenyl)-2*H*-tetrazolium] (MTS) at a ratio of MTS/PMS = 2/0.92 mg/mL in PBS was added and the plates were incubated for 3 h at 37 °C. Subsequently, the absorption at 490 nm was recorded using an EnVision Xcite 2104 (PerkinElmer), and the response was normalized to vehicle control and Puromycin treated samples. Dose response curves were fitted to a “sigmoidal dose response (variable slope)” model using GraphPad Prism 5 (GraphPad Software, Inc.).

#### Cell-Based Tagging Assay

HEK293a *SHH*^*+*^ cells were plated at 500,000 cells/well of a 6-well plate in a volume of 3 mL/well and cultured for 24 h as described. Cells were treated with DMSO vehicle, or RUSKI compounds in DMSO (0.016–10 μM final). After 1 h, 3 μL of YnPal (20 mM DMSO stock; 20 μM final) were added and the cells were cultured for another 6 h. Subsequently, cells were washed with PBS and lysed using PBS supplemented with 1% Triton, 0.1% SDS and Complete EDTA-free protease inhibitor cocktail (Roche Diagnostics). Cells were scraped off the plates and lysates centrifuged at 13 000*g* for 10 min at 4 °C to remove cell debris. Protein concentration was determined using the DC Protein Assay (Bio-Rad) following the manufacturer’s procedure.

The click reaction in cell lysate was performed as previously described.^42^ Briefly, 1 μL AzTB (10 mM in DMSO, 100x), 2 μL CuSO_4_ (50 mM in H_2_O, 50x), 2 μL tris(2 carboxyethyl)phosphine hydrochloride (TCEP) (50 mM in H_2_O, 50×), and 1 μL tris[(1-benzyl-1*H*-1,2,3-triazol-4-yl)methyl]amine (TBTA, 10 mM in DMSO, 100×) were mixed. Then 6 μL of this click mixture were added to 100 μL of lysate, adjusted with lysis buffer to a protein concentration of 1 mg/mL. The click reaction was shaken at RT for 1 h. Subsequently, proteins were precipitated by adding methanol, chloroform, and water at a ratio of 2:0.5:1 to remove click reagents and excess of AzTB. The mixture was centrifuged (13 000*g*, 5 min at 4 °C) and the methanol and water containing top layer removed. Following the addition of an excess of methanol, the precipitated proteins were isolated by centrifugation (13 000*g*, 10 min at 4 °C) and washed twice with methanol. The protein pellets were air-dried and redissolved in PBS containing 0.2% SDS and 0.1 mM DTT using sonication, and adjusted to a final protein concentration of 1 mg/mL.

For enrichment of YnPal-labeled proteins, 100 μg of lysate were incubated with 30 μL of prewashed (3 × 500 μL of 0.2% SDS in PBS) Dynabeads MyOne Streptavidin C1 beads (Invitrogen) for 1 h at room temperature. Beads ware washed twice with 500 μL of 0.2% SDS in PBS and boiled for 10 min in 15 μL of PBS containing SDS-PAGE sample buffer. The entire pull-down sample and 10 μg of total lysate and supernatant fractions were loaded on a 15% SDS-PAGE gel. Fluorescence was scanned using a Typhoon imager (GE Healthcare; excitation laser: 532 nm, emission filter: LPG (575–700 nm); PMT: 750 V).

Proteins were transferred to nitrocellulose membrane using a wet transfer apparatus (100 V, 1 h). Membranes were blocked with 5% BSA in TBS-T (0.1% Tween-20, 50 mM Tris, 150 mM NaCl, pH 7.5) for 1 h at room temperature, followed by overnight incubation with SHH H-160 primary antibody (Santa Cruz Biotechnology; sc-9024; 1:200 in TBS-T + 0.5% BSA) or α-tubulin primary antibody (Santa Cruz Biotechnology; sc-8035; 1:200 in TBS-T + 0.5% BSA) at 4 °C. Membranes were washed with TBS-T and further incubated at RT for 1 h with the appropriate secondary antibody (HRP conjugated goat-antimouse IgG (H+L) or goat-antirabbit IgG (H+L); Advansta; 1:10,000 in TBS-T + 0.5% BSA). Western blots were washed and developed with Luminata Crescendo Western HRP substrate (Millipore) on a Fujifilm LAS-3000 imager.

To determine the amount of YnPal labeled SHH, intensities of the corresponding bands in the fluorescence images were quantified using ImageJ 1.50i (National Institute of Health, USA). Corresponding values from biological duplicates were further evaluated using a “sigmoidal dose response (variable slope)” model in GraphPad Prism 5 (GraphPad Software, Inc.).

#### Cell-Based Signaling Assay

The Light2 cell-based signaling assay has been described previously.^35^ Briefly, HEK293a *SHH*^*+*^ cells were plated at 120,000 cells/well of a 12-well plate in a volume of 1 mL/well and cultured as described above. After 24 h, cells were washed with PBS, followed by addition of 1 mL of media containing 0.2% DMSO or varying inhibitor concentrations in DMSO (0.041–30 μM final). Additionally, SHH-Light2 cells were plated at 20,000 cells/well of a 96-well plate in 50 μL media. After another 24 h, 300 μL of the conditioned media were collected from each well of the treated HEK293a *SHH*^*+*^ cells, centrifuged at 1000*g* for 10 min to remove detached cells and split into three wells of the SHH-Light2 cells (100 μL/well; 150 μL final volume in each well). The treated Light2 cells were cultured for another 48 h and subsequently washed with PBS. *Firefly* and *Renilla* luciferase activity was measured using the Dual-Luciferase reporter system (Promega Corporation, USA). Then 20 μL of 1× passive lysis buffer were added to each well. After 30 min at RT, 5 μL of each lysate were transferred to a white opaque 96-well plate. Then 20 μL/well of luciferase assay reagent II was added and the luminescence immediately recorded using a SpectraMax i3x plate reader (Molecular Devices LLC). Subsequently, 20 μL/well of 1× Stop&Glo substrate in Stop&Glo buffer was added and the luminescence immediately recorded for a second time. Data was evaluated using a “sigmoidal dose response (variable slope)” model in GraphPad Prism 5 (GraphPad Software, Inc.). The luminescence signal of the *Firefly* luciferase was normalized to the DMSO vehicle (100%) and the unconditioned media (0%) controls.

#### Caco-2 Permeability Assay

Apparent permeability (*P*_app_) was determined in the Caco-2 human colon carcinoma cell line. Cells were maintained in DMEM containing 10% fetal bovine serum, penicillin and streptomycin in a humidified atmosphere with 5% CO_2_ for 10 days. Cells were plated onto a cell culture assembly plate (Millipore, UK) and monolayer confluency was checked using a TEER electrode prior to the assay. Medium was washed off and replaced in the appropriate apical and basal wells with HBSS buffer (pH 7.4) containing compounds (10 μM, 1% DMSO). The Caco-2 plate was incubated for 2 h at 37 °C, and Lucifer yellow was used to confirm membrane integrity postassay. Samples from the apical and basolateral chambers were analyzed against an external, matrix-matched, standard curve by LC-MS/MS using a Waters (Elstree, Herts. UK) Acquity H-class LC system coupled to a Waters TQ-S mass spectrometer. *P*_app_ was determined as follows:
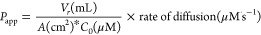
where *V*_r_ = volume of receptical; *A* = surface area of monolayer; *C*_0_ = Initial compound concentration in donor.

#### PAMPA Assay

Passive diffusion was estimated using the PAMPA method. The assay used an artificial membrane consisting of 2% phosphatidyl choline (Sigma-Aldrich, no. P3556) in dodecane. The donor plate was a MultiScreen-IP Plate with 0.45 μm hydrophobe Immobilon-P Membrane (Millipore, #MAIPNTR10) and the acceptor plate was a MultiScreen 96-well Transport Receiver Plate (Millipore, #MATRNPS50). Permeability of 10 μM test compound was measured postincubation at 30 °C for 16 h at 3 different donor pH levels (pH 5, 6.5 and pH 7.4). Acceptor pH was 7.4. All samples were analyzed against an external, matrix-matched, standard curve by LC-MS/MS with a Waters (Elstree, Herts. UK) Acquity H-class LC system coupled to a Waters TQ-S mass spectrometer. Permeability values (cm/s) were calculated using the following equation:
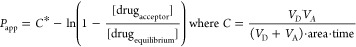
and *V*_D_ = volume of donor; *V*_A_ = volume of acceptor; area = surface area of the membrane · porosity.

#### Microsomal Incubations

Metabolic stability assays were performed using a Microlab Star liquid handling workstation (Hamilton Robotics, Bonaduz, Switzerland). Test compounds (1 μM, 1% DMSO) were preincubated at 37 °C for 10 min in 0.5 mg/mL female CD1 mouse and mixed gender human liver microsomes in 10 mM phosphate buffered saline (PBS; Sigma-Aldrich Company Ltd. (Dorset, UK)). Microsomes were purchased either from Sekisui XenoTech, LLC (Kansas City, USA) or BioreclamationIVT (Frankfurt Am Main, Germany). Reactions were initiated by the addition of NADPH (final concentration 1 mM). At 0, 15, and 30 min, aliquots were removed from each incubation and quenched in 3 volumes of ice-cold methanol containing olomoucine (Sigma-Aldrich, Dorset, UK) as an internal standard. Inactive control incubations (without NADPH) were conducted in parallel. Samples were centrifuged at 3700 rpm at 4 °C for 30 min and the supernatant taken for analysis by LC-MS using an Agilent (Stockport, UK) 1290 LC system coupled to an Agilent 6520 QTOF mass spectrometer. The percentage metabolized was calculated by comparing peak area ratio (peak area test compound/peak area of internal standard) at *t* = 15 and 30 min versus *t* = 0 min.

## Supporting Information Available

- Acyl-cLIP assay results; molecular docking; MTS assay results; cell-based tagging assay results; cell-signaling assay; results; drug metabolism and pharmacokinetics (DMPK) results; profile of IMP-1575; HPLC chromatograms; conformational characteristics; NMR spectra; references (PDF)
- SMILES molecular formula strings and the enzyme-based and cellular activity data (csv) (ZIP)
- PDB coordinates for the initial homology models; 28 top 5 pose.pdb; 43 top 5 pose.pdb; compound 3R docking.pdb (ZIP)

## ABBREVIATIONS USED

Ac: acetyl
Bn: benzyl
Boc: *tert*-butyloxycarbonyl
BSA: bovine serum albumin
CDI: carbonyldiimidazole
DCM: dichloromethane
DIPEA: *N*,*N*-diisopropylethylamine
DMEM: Dulbecco’s Modified Eagle Medium
DMF: *N*,*N*-dimethylformamide
DMSO: dimethyl sulfoxide
DTT: dithiothreitol
EDC: 1-ethyl-3-(3-(dimethylamino)propyl)carbodiimide
EtOAc: ethyl acetate
EtOH: ethanol
h: hour
HOBt: hydroxybenzotriazole
HPLC: high-performance liquid chromatography
LCMS: HPLC with mass spectral detection
NMR: nuclear magnetic resonance spectroscopy
M: molar
mM: millimole
μM: micromole
MeOH: methanol
MES: 2-(*N*-morpholino)ethanesulfonic acid
PAMPA: parallel artificial membrane permeability assay
PBS: Phosphate-buffered saline
RT: room temperature
SDS: sodium dodecyl sulfate
SDS-PAGE: sodium dodecyl sulfate polyacrylamide gel electrophoresis
TCEP: tris(2-chloroethyl) phosphate
TFA: trifluoroacetic acid
