# Supplementary material for: Design, Synthesis, and Evaluation of Inhibitors of Hedgehog Acyltransferase
Source: J Med Chem. 2024 Jan 10;67(2):1061–78. doi: 10.1021/acs.jmedchem.3c01363 (PMC10823475; doi:10.1021/acs.jmedchem.3c01363)
Supplement: Supplementary file 1 — jm3c01363_si_001.pdf [file jm3c01363_si_001.pdf]

Supporting Information for:

Design, Synthesis and Evaluation of Inhibitors of Hedgehog Acyltransferase

Markus Ritzefeld[a], Leran Zhang[a], Zhangping Xiao[a], Sebastian A. Andrei[a], Olivia Boyd[a], Naoko Masumoto[a], Ursula R. Rodgers[b], Markus Artelsmair[a], Lea Sefer[c], Angela Hayes[d], Efthymios-Spyridon Gavriil[a], Florence I. Raynaud[d], Rosemary Burke[d], Julian Blagg[d], Henry S. Rzepa[a], Christian Siebold[c], Anthony I. Magee[b], Thomas Lanyon-Hogg[a,e*], Edward W. Tate[a*]

[a]Department of Chemistry, Imperial College London, London W12 0BZ, UK.

[b]National Heart and Lung Institute, Imperial College London, London SW7 2AZ, UK.

[c]Division of Structural Biology, University of Oxford, Oxford OX3 7BN, UK.

[d]Division of Cancer Therapeutics, Centre for Cancer Drug Discovery, Institute of Cancer Research, London SM2 5NG, UK

[e]Current address: Department of Pharmacology, University of Oxford, Oxford OX1 3QT, UK

*thomas.lanyon-hogg@pharm.ox.ac.uk and e.tate@imperial.ac.uk

1. Table of Contents

1. Table of Contents 1
2. Acyl-cLIP Assay Results 2
3. Molecular docking 5
4. MTS Assay Results 6
5. Cell-based Tagging Assay Results 8
6. Cell-Signaling Assay Results 10
7. Drug Metabolism and Pharmacokinetics (DMPK) Results 12
8. Profile of IMP-1575 16
9. HPLC Chromatograms 17
10. Conformational Characteristics 24
11. NMR Spectra 26
12. References 144

2. Acyl-cLIP Assay Results

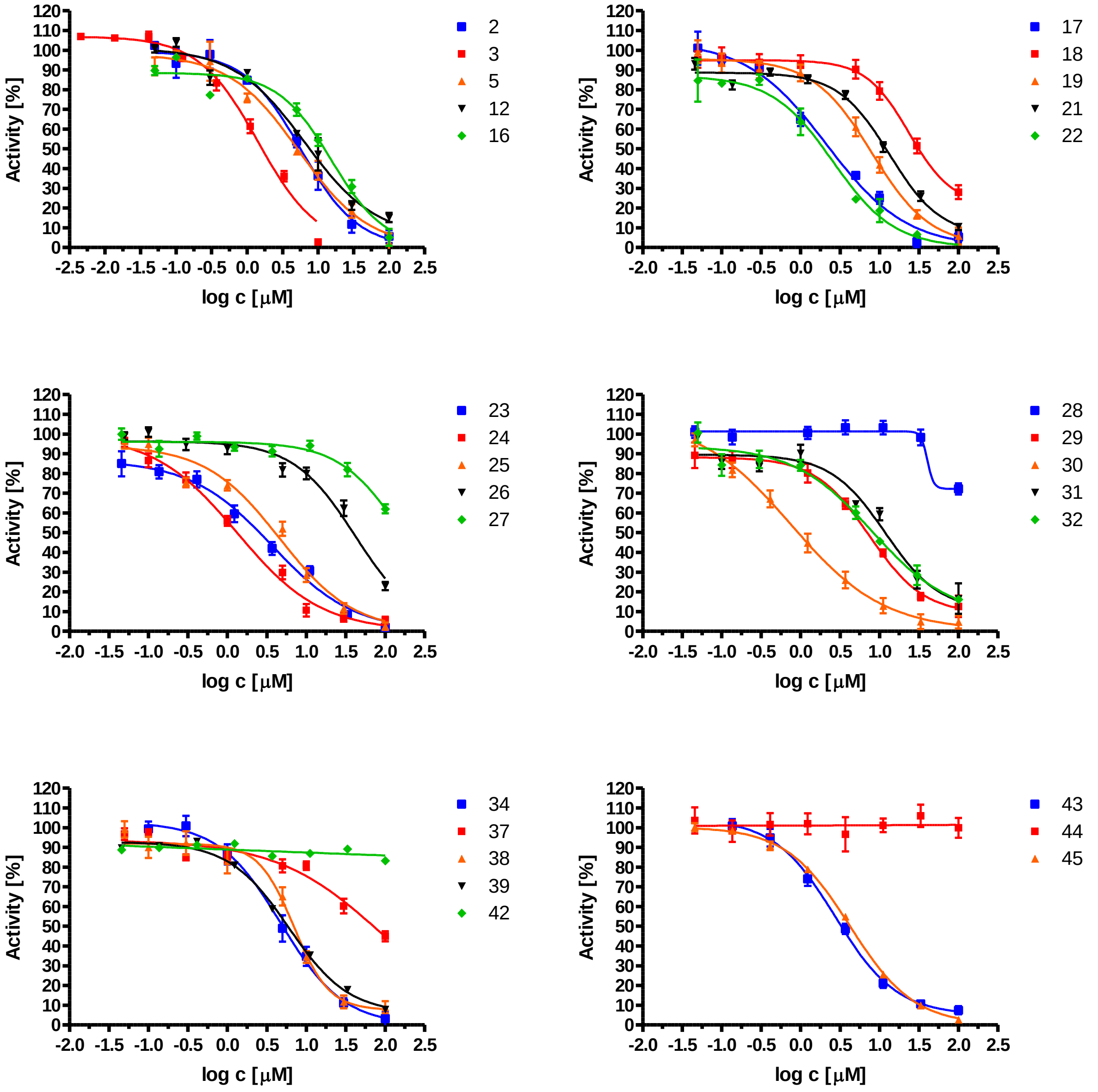

Figure S1. Inhibitory potency of selected analogues against HHAT determined via Acyl-cLIP assay (n=3).

Table S1: Inhibitory potency of selected analogues against HHAT determined via Acyl-cLIP assay. The values were extracted from the dose-response curves shown in **Figure S1** using a sigmoidal dose-response model in GraphPad Prism 5. CI_{95} = 95% confidence interval. $LLE_{Acyl\text{-}cLIP}$ = ligand-lipophilicity efficiency determined from the IC_{50} values in the Acyl-cLIP assay (n=3) and $clogD_{pH7.4}$ calculated at pH 7.4 using the 'JClogD' function of JChem (ChemAxon Ltd.). The lead HHAT inhibitor **30** (IMP-1575) is highlighted.

Compound No.	IC_{50} [µM]	CI_{95} [µM]	R^2 [%]	$clogD_{pH7.4}$	$LLE_{Acyl\text{-}cLIP}$
**2**	5.76	3.96-8.37	96.9	2.76	2.48
**3**	1.44	0.89-2.31	98.2	2.68	3.16
**5**	5.34	2.98-9.57	95.5	2.88	2.39
**12**	6.55	3.66-11.72	95.8	2.24	2.94
**16**	15.45	7.74-30.82	95.3	4.15	0.66
**17**	2.13	1.34-3.39	97.2	2.76	2.91
**18**	23.72	15.31-36.73	95.3	1.89	2.73
**19**	7.98	5.49-11.61	96.5	1.26	3.84
**21**	13.38	7.41-24.14	99.1	2.03	2.84
**22**	2.58	1.39-4.78	94.4	1.39	4.20
**23**	3.71	1.68-8.19	99.1	1.39	4.04
**24**	1.33	0.88-2.02	98.6	0.98	4.90
**25**	4.62	3.10-6.87	97.5	1.44	3.90
**26**	>50			3.53	
**27**	>100			2.03	
**28**	>100			0.98	
**29**	7.45	5.87-9.45	99.9	2.03	3.10
**30**	0.75	0.49-1.14	99.8	0.98	5.14
**31**	11.86	6.69-21.02	93.1	2.72	2.21
**32**	8.09	3.98-16.46	94.6	2.09	3.00
**34**	4.79	3.19-7.21	97.0	1.97	3.35
**37**	>100			2.97	
**38**	7.01	5.39-9.11	97.1	2.33	2.82
**39**	6.02	4.07-8.92	99.6	1.92	3.30
**42**	>100			1.92	
**43**	2.77	2.16-3.54	99.9	1.92	3.64
**44**	>100			2.02	
**45**	4.22	3.61-4.92	99.9	2.94	2.43

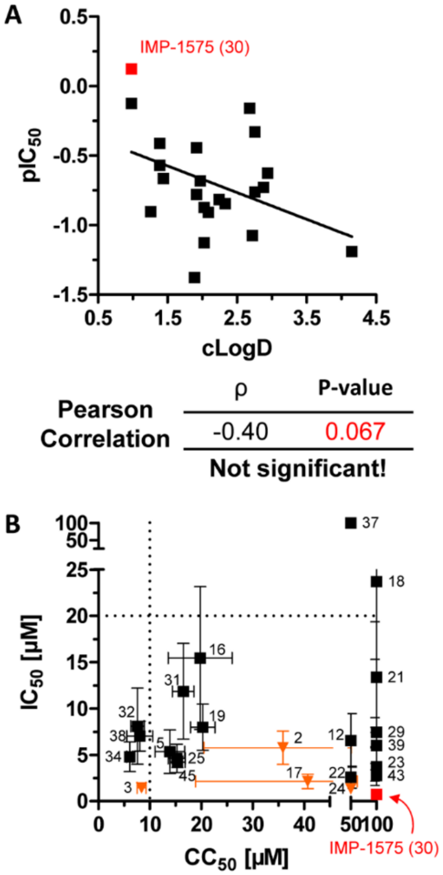

Figure S2. A) Plot of the pIC_{50} value obtained by Acyl-cLIP assay versus the corresponding $cLogD_{7.4}$. No significant Pearson correlation between the data sets was detectable (ρ = Pearson correlation coefficient). The new lead compound IMP-1575 (**30**) is highlighted in red. **B)** Plot of IC_{50} [µM] values obtained from the Acyl-cLIP assay, versus CC_{50} [µM] values determined for HEK293 *SHH*$^{+}$ cells via MTS assay. Previously reported inhibitors are highlighted in orange (▼) and the new lead compound IMP-1575 (**30**) in red (■). Compounds with an IC_{50} below 20 µM and a CC_{50} above 10 µM were propagated into cellular activity assays.

3. Molecular docking

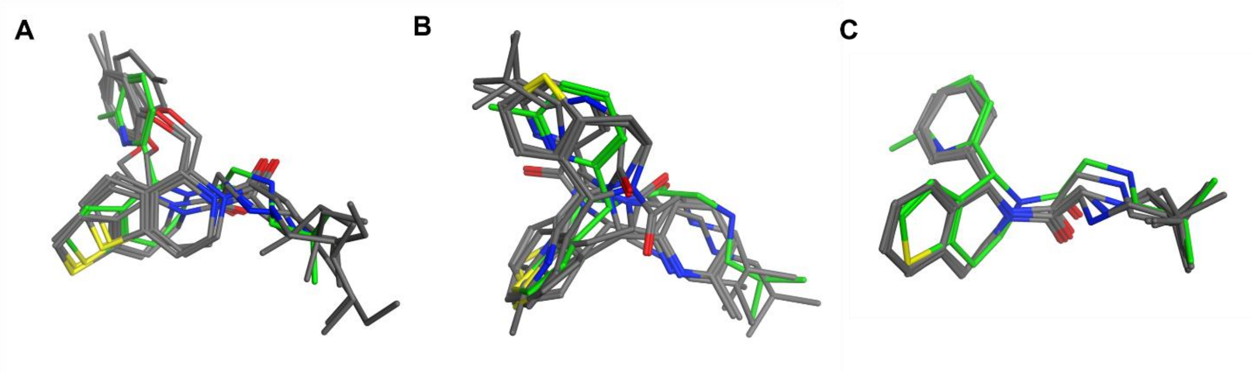

Figure S3. Top docking poses of IMP-1575 analogues binding to HHAT. **A)** Poses of **3**; **B)** Poses of **28**; **C)** Poses of **43**. Docking studies were performed with the Molecular Operating Environment (MOE) software version 2022. Minimization was applied on both the receptor and ligands before docking using QuickPrep Panel with its default values. The Triangle Matcher placing method and the Rigid Receptor refinement method were employed, and docking results were scored with London dG and GBVI/WSA dG. Top 5 poses from in total 100 docked poses are selected for analysis. The 2D ligand interactions images were generated with the following cutoffs. H-bond is -0.5 kcal/mol; ionic is -0.5 kcal/mol; maximum distance cutoff is 4.0 angstroms.

4. MTS Assay Results

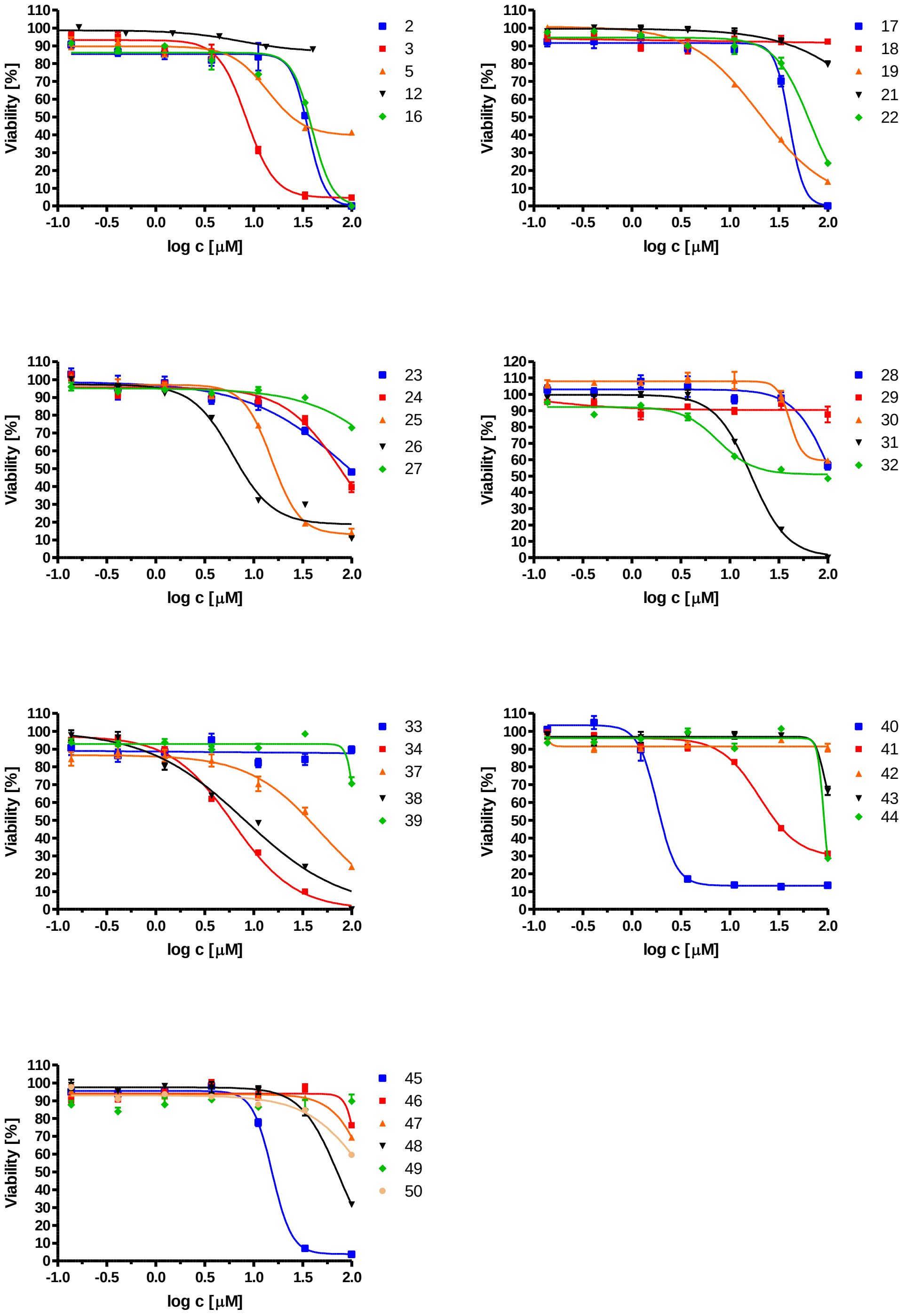

Figure S4. MTS assay dose-response curves for toxicity of selected analogues against HEK293a SHH⁺ cells (n=3).

Table S2: Toxicity data of selected analogues against HEK293a SHH^+ cells using the MTS assay. Values were extracted from dose-response curves shown in **Figure S4** using a sigmoidal dose-response model in GraphPad Prism 5. CI_{95} = 95% confidence interval; AUC = area under the curve normalized to the area at a viability of 100% over the investigated concentration range (n=3).

Compound No.	CC_{50} [µM]	CI_{95} [µM]	AUC	R^2 [%]
**2**	35.9	20.5-63.2	0.72	93.6
**3**	8.4	7.53-9.46	0.60	93.6
**5**	13.9	11.0-17.9	0.74	93.7
**12**	>50		0.95	/
**16**	19.8	13.6-28.8	0.68	95.3
**17**	40.7	18.9-88.5	0.80	95.9
**18**	>100		0.93	/
**19**	20.3	17.9-23.0	0.56	99.3
**21**	>100		1.02	/
**22**	>50		0.77	/
**23**	>100		0.86	/
**24**	>50		0.86	/
**25**	15.3	13.6-17.1	0.73	97.1
**26**	6.1	5.8-7.0	0.64	96.8
**27**	>100		0.98	/
**28**	>100		0.98	/
**29**	>100		0.98	/
**30**	>100		1.00	/
**31**	16.5	14.4-18.9	0.73	97.6
**32**	7.6	6.3-9.1	0.76	94.8
**33**	>100		0.83	/
**34**	6.1	5.5-6.8	0.56	98.8
**37**	47.9	13.4-171.9	0.73	84.7
**38**	8.1	5.5-11.7	0.60	95.6
**39**	>100		0.97	/
**40**	1.8	1.5-2.2	0.49	96.3
**41**	20.2	18.1-22.6	0.79	97.8
**42**	>100		0.98	/
**43**	>100		1.00	/
**44**	>100		0.96	/
**45**	15.4	13.9-17.0	0.70	97.9
**46**	>100		0.93	/
**47**	>50		0.92	/
**48**	>100		0.90	/
**49**	>100		0.87	/
**50**	>100		0.88	/

5. Cell-based Tagging Assay Results

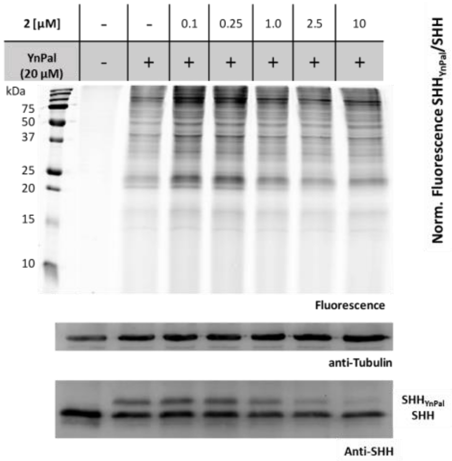

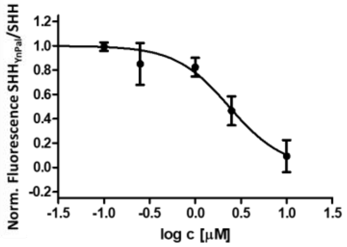

Compound 2	
EC_{50} [µM]	2.35
95% Confidence Interval [µM]	1.68-3.29
R^2 [%]	92.7

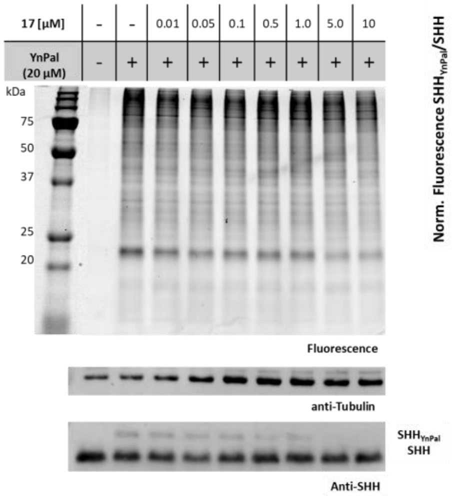

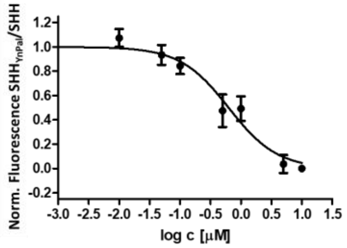

Compound 17	
EC_{50} [µM]	0.57
95% Confidence Interval [µM]	0.40-0.79
R^2 [%]	95.9

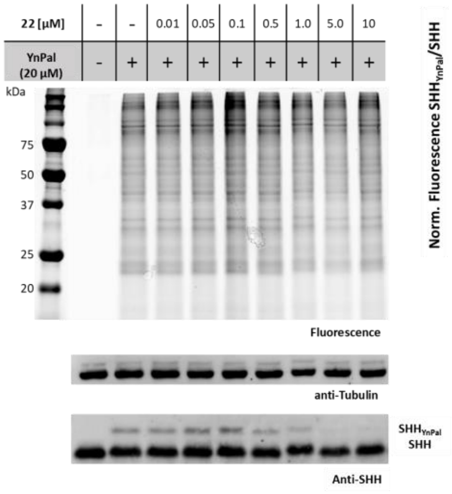

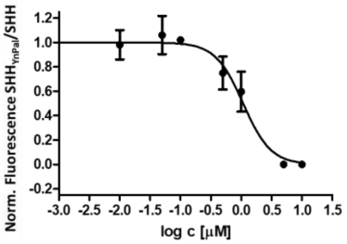

Compound 22	
EC_{50} [µM]	1.08
95% Confidence Interval [µM]	0.89-1.29
R^2 [%]	96.3

Figure S5. Tagging assay results of **2**, **17** and **22**. Quantification of the inhibitory potency was performed using the ratio between the unlabeled SHH and YnPal-labeled SHH modified with AzTB (YnPal-SHH) using anti-SHH immunoblotting. The corresponding ratios were normalized using the two controls (-YnPal and -inhibitor) and plotted against the logarithm of the concentration. EC_{50} values were extracted by nonlinear regression using a sigmoidal dose response model (n =3).

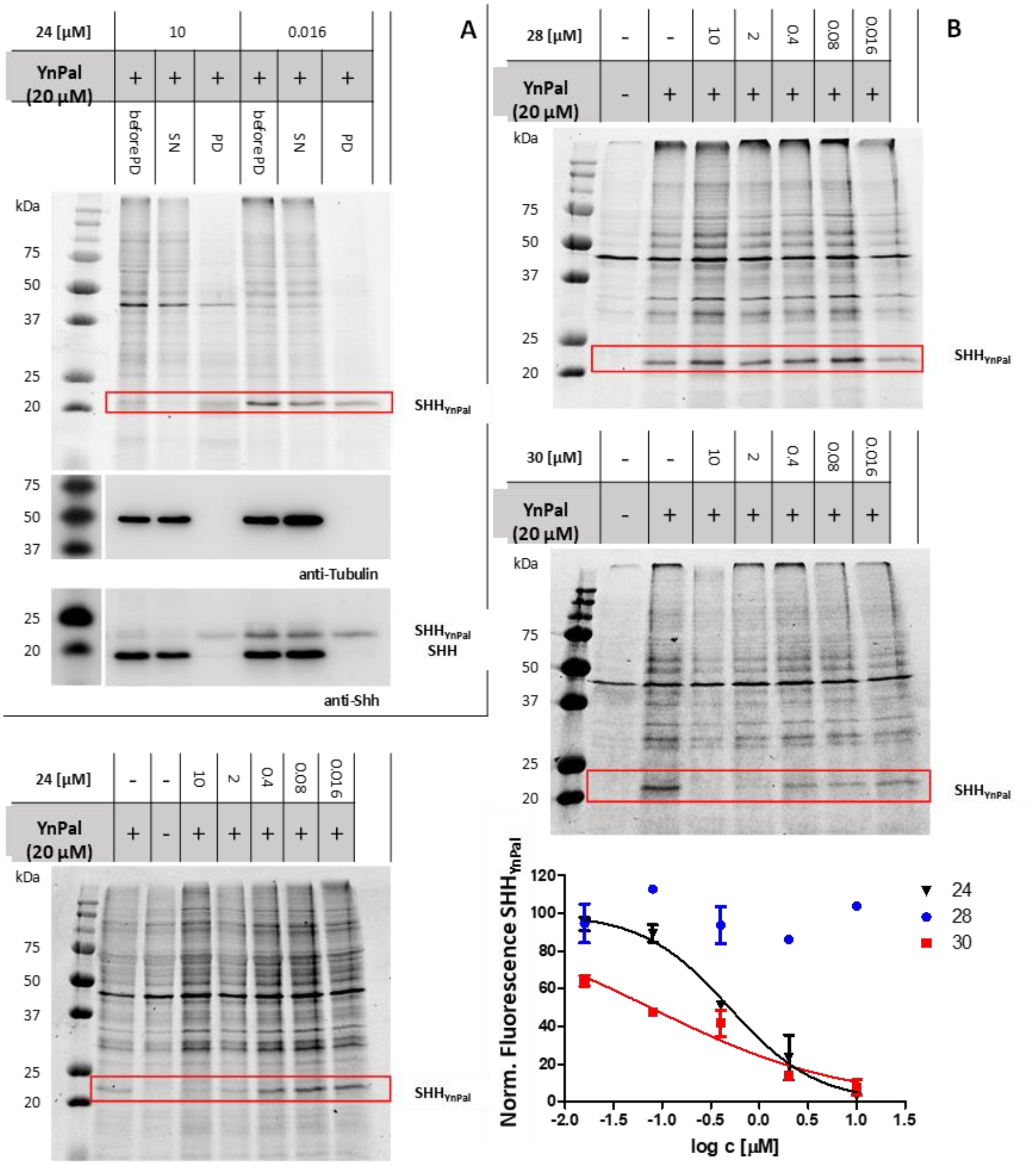

Compound	24 ▼	28 (S) ●	30 (R) ■
EC_{50} (CI_{95}) [µM]	0.49 (0.23-1.06)	>10	0.076 (0.0096-0.60)
R^2 [%]	96.5		91.2

Figure S6. Tagging assay results of **24**, **28**, and **30**. **A)** Findings from pull-down experiments at high and low concentrations of **24** indicate that the fluorescent band at ~19 kDa corresponds to YnPal-tagged and AzTB modified SHH, and that the fluorescence signal can be used to quantify the inhibitory potency. PD = pull-down; SN = supernatant. **B)** Quantification of the inhibitory potency of **24**, **28**, and **30** using the fluorescence signal of YnPal-labeled SHH modified with AzTB. The densitometric results from two replicates were normalized using the two controls (-YnPal and -inhibitor) and plotted against the logarithm of the concentration. EC_{50} values were extracted by nonlinear regression using a sigmoidal dose response model. CI_{95} = 95% confidence interval (n=2).

6. Cell-Signaling Assay Results

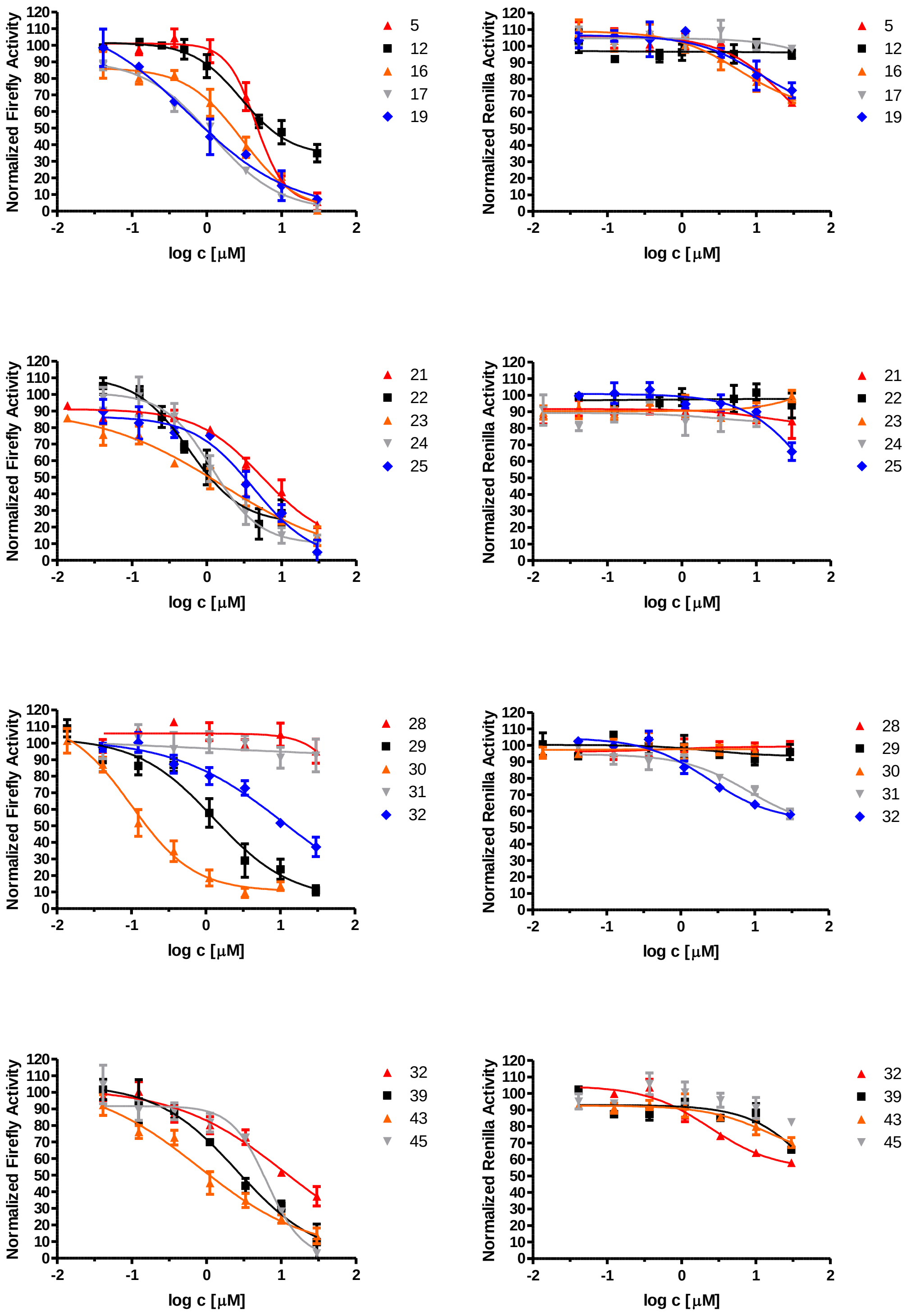

Figure S7. Cell-signaling assay dose-response curves. The left graphs show *Firefly* luciferase activities normalized to *Renilla* luciferase activity and, subsequently, to vehicle (DMSO) control and Light2 cells treated with media that does not contain SHH The right graphs show *Renilla* luciferase activities normalized to vehicle (DMSO) control (n=3).

Table S3: Cell-signaling data for selected HHAT inhibitor analogues. Values were extracted from dose-response curves shown in **Figure S7** using a sigmoidal dose-response model in GraphPad Prism 5. CI_{95} = 95% confidence interval. LLE_{Light2} = ligand-lipophilicity efficiency determined from the EC_{50} values of the cell-signaling assay. AUC = area under the curve normalized to the area at 100% *Renilla* activity over a concentration range from 10 to 0.041 µM (n=3).

**Compound No.**	***Firefly***					***Renilla***
	**EC_{50} [µM]**	**CI_{95} [µM]**	**R^2 [%]**	**$clogD_{pH7.4}$**	**LLE_{Light2}**	**AUC [%]**
**5**	4.56	3.49-5.95	97.5	2.88	2.46	101.3
**12**	2.92	1.62-5.27	92.8	2.24	3.29	115.2
**16**	2.96	1.58-5.55	96.3	4.15	1.38	100.1
**17**	1.17	0.80-1.69	98.8	2.76	3.17	104.2
**19**	0.58	0.092-3.66	91.2	1.26	4.98	101.6
**21**	5.46	2.62-11.40	97.0	2.03	3.23	91.2
**22**	0.58	0.30-1.12	80.1	1.39	4.85	116.8
**23**	1.58	0.22-11.19	93.8	1.39	4.41	91.2
**24**	1.18	0.66-2.12	90.8	0.98	4.95	88.1
**25**	4.25	1.29-13.96	90.4	1.44	3.93	97.9
**28**	>30			0.98		97.7
**29**	1.29	0.51-3.26	92.9	2.03	3.86	98.7
**30**	0.099	0.046-0.214	95.5	0.98	6.02	98.3
**31**	>30			2.72		87.5
**32**	>30			2.09		89.6
**39**	2.59	0.89-7.56	92.3	1.92	3.67	90.1
**43**	0.80	0.22-2.96	92.5	1.92	4.18	89.8
**45**	6.44	3.98-10.41	94.3	2.94	2.25	97.9

7. Drug Metabolism and Pharmacokinetics (DMPK) Results

Table S4: Parallel Artificial Membrane Permeability Assay (PAMPA) results. Data for selected HHAT inhibitor analogues and control compounds at 10 µM.

Compound	pH 5			pH 6.5			pH 7.4		
	P_{app} *10^{-6} [cm/s]	Permeability Class	Recovery [%]	P_{app} *10^{-6} [cm/s]	Permeability Class	Recovery [%]	P_{app} *10^{-6} [cm/s]	Permeability Class	Recovery [%]
**2**	<1.5	low	<31%	10.9	medium	<10%	20.4	high	<10%
**5**	>200	high	95%	117.0	high	98%	>200	high	91%
**17**	<0.7	low	<31%	<9.7	medium	<10%	<4.5	low	<10%
**22**	<0.4	low	<65%	13.0	medium	<23%	42.4	high	<15%
**37**	>200	high	95%	>200	high	92%	>200	high	89%
**38**	1.3	low	91%	24.4	high	45%	53.9	medium	17%
**Controls**									
**Antipyrine**	6.5	medium	109%	6.6	medium	119%	7.3	medium	145%
**Nadolol**	<0.4	low	97%	<0.4	low	99%	<0.3	low	135%
**Verapamil**	<0.5	low	82%	19.2	medium	33%	116.9	high	39%
**Coumarin**	124.4	high	99%	>eqm	>eqm	99%	>eqm	>eqm	114%

PAMPA Classifications	
**low**	<5
**medium**	5-20
**high**	>20

Table S5: Caco-2 results. Data for selected HHAT inhibitor analogues at 10 µM. Compounds were analyzed in the presence and absence of P-glycoprotein inhibitor Verapamil.

Compound	A>B		B>A		Efflux Ratio (B>A/A>B)
	P_{app} *10-6 [cm/s]	Recovery [%]	P_{app} *10-6 [cm/s]	Recovery [%]	
**2**	14.5 ± 0.4	56.7 ± 1.9	12.4 ± 2.1	87.8 ± 1.2	0.86
**2 + Verapamil**	8.3 ± 0.9	41.1 ± 1.0	12.5 ± 1.9	51.6 ± 1.0	1.49
**17**	17.0 ± 2.5	60.5 ± 2.4	17.4 ± 4.1	80.4 ± 2.2	1.02
**17 + Verapamil**	14.2 ± 0.8	58.6 ± 0.9	19.8 ± 2.0	66.6 ± 1.9	1.40
**22**	39.2 ± 0.7	88.7 ± 0.1	29.7 ± 0.2	107.9 ± 3.1	0.76
**22 + Verapamil**	35.5 ± 2.0	70.0 ± 2.5	22.7 ± 3.3	75.7 ± 2.0	0.64
**Controls**					
**CCT352141 (Efflux)**	1.8 ± 0.0	67.6 ± 3.3	37.0 ± 10.6	93.3 ± 3.5	20.5
**CCT352141 + Verapamil (Efflux)**	7.1 ± 0.4	69.7 ± 6-.4	10.1 ± 0.5	92.2 ± 3.3	1.4
**CCT251981 (low P_{app})**	<0.8 ± 0.0	81.2 ± 5.6	1.0 ± 0.2	112.3 ± 2.5	1.3
**CCT138379 (high P_{app})**	39.9 ± 1.7	81.2 ± 4.1	34.6 ± 4.5	99.8 ± 6.0	0.9

Caco-2 Classifications	
**low**	<2
**medium**	2-20
**high**	>20

Table S6: Metabolic stability in microsomes from mouse (MLM) and human (HLM). Data for selected HHAT inhibitor analogues and verapamil control at 1 µM

Compound	MLM % metabolized after		HLM % metabolized after	
	15 min	30 min	15 min	30 min
**2**	90.8	98.4	43.0	69.5
**5**	99.5	99.4	52.5	64.3
**17**	99.5	100.0	44.4	66.7
**22**	98.1	99.7	36.9	56.2
**37**	94.6	99.8	45.2	55.9
**38**	100.0	100.0	30.3	49.2
**Verapamil (control)**	2.2	99.8	6.0	94.6

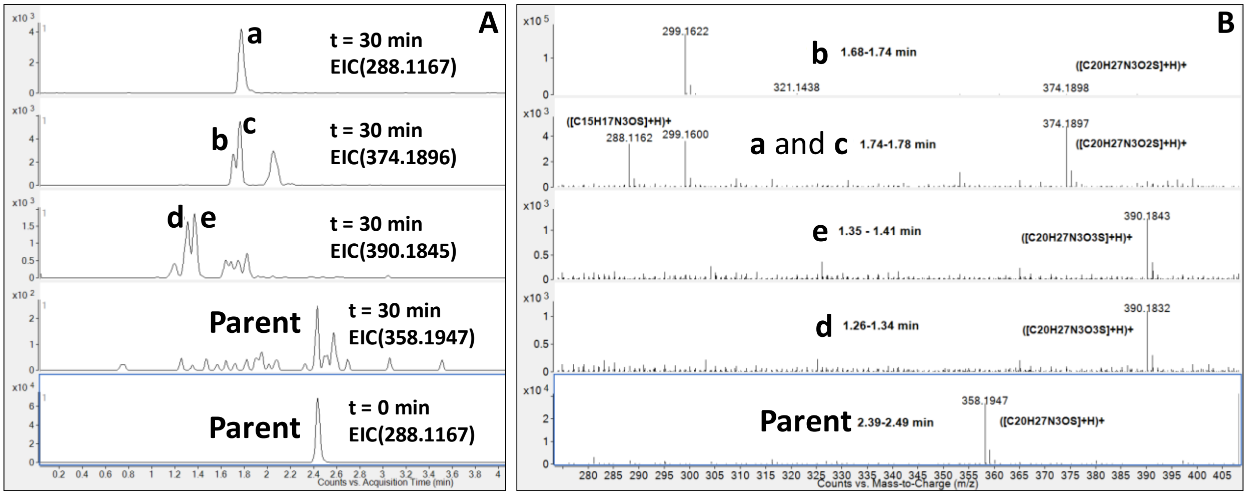

C

	Formula	Calculated [m/z]	Measured [m/z]	Formula difference to parent
**Parent**	$C_{20}H_{28}N_3OS$ [M+H]$^+$	358.1953	358.1947	-
**a**	$C_{15}H_{18}N_3OS$ [M+H]$^+$	288.1170	288.1162	Parent - C_5H_{10}
**b + c**	$C_{20}H_{28}N_3O_2S$ [M+H]$^+$	374.1898	374.1898 ; 374.1897	Parent + O
**d + e**	$C_{20}H_{28}N_3O_3S$ [M+H]$^+$	390.1851	390.1832 ; 390.1843	Parent + 2O

D

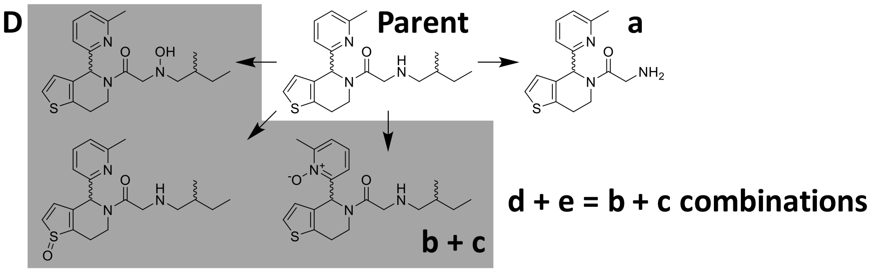

Figure S8. Metabolite identification results of 22. Extracted ion chromatograms (EIC, **A**) and corresponding high-resolution mass spectra (**B**) of **22** (**parent**) at t = 0 and 30 minutes. Table **C** and **D** show molecular formulas and possible structures of the major metabolites i.e. *N*-dealkylated metabolite (**a**), mono-oxidative metabolites (**b** and **c**) and bis-oxidative metabolites (**d** and **e**).

8. Profile of IMP-1575

Compound 30 (IMP-1575)	
**HHAT IC_{50} (µM)**	0.75
**Cell signalling EC_{50} (µM)**	0.10
**LLE (based on IC_{50} - $clogD_{pH7.4}$)**	5.14
**LLE (based on EC_{50} - $clogD_{pH7.4}$)**	6.02
**MW (g/mol)**	343.496
**clogP**	2.78
**$clogD_{pH7.4}$**	0.98
**H-bond donors**	1
**H-bond acceptors**	4
**Lipinski rule of 5**	Satisfied (4 out of 4)
**Fsp^3**	0.47
**TPSA [$Å^2$]**	45.2
**Heavy atom count**	24
**Rotatable bonds**	5

Table S7. Profile of compound **30 (IMP-1575).** Potency in Acyl-cLIP and cellular signalling assays, respective Ligand Lipophilicity Efficiency (LLE) values and drug-like properties. Properties were calculated using CDD Vault (Collaborative Drug Discovery Inc.) and JChem (ChemAxon Ltd.) for clogP and clogD values. clogD values were calculated at pH 7.4.

9. HPLC Chromatograms

Analytial HPLC was performed on an Agilent 1260 Infinity Series with a C18 4.6 mm x 250 mm (eluent: CH_3CN (5-90% in 4 min) and H_2O with 0.1 % TFA; flow rate 1 mL/min). Analytial chiral HPLC was performed on an Agilent 1260 Infinity Series equipped with a CHIRALPAK-IA 4.6 mm x 250 mm (eluent: hexane:propan-2-ol 90:10 or 80:20; flow rate 1 mL/min, Method **A**), a CHIRALPAK-ID 4.6 mm x 250 mm (eluent: isocratic hexane:propan-2-ol 90:10; flow rate 1 mL/min, Method **B**) or a CHIRALPAK-IF 4.6 mm x 250 mm (eluent: isocratic hexane:propan-2-ol 90:10 or 80:20; flow rate 1 mL/min, Method **C**).

5 (Rt=2.2; 97.2%)

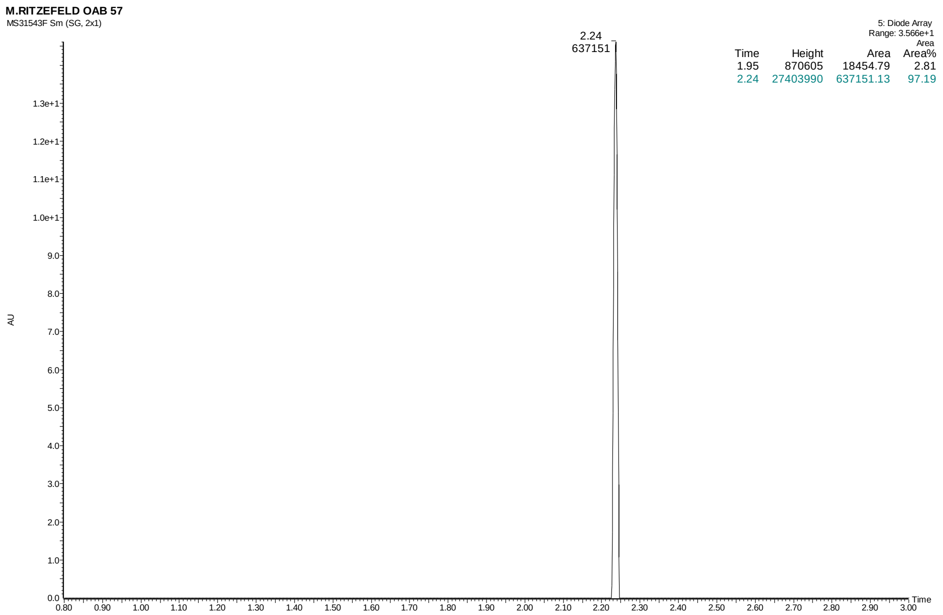

12 (Rt=1.6; 89.7 %)

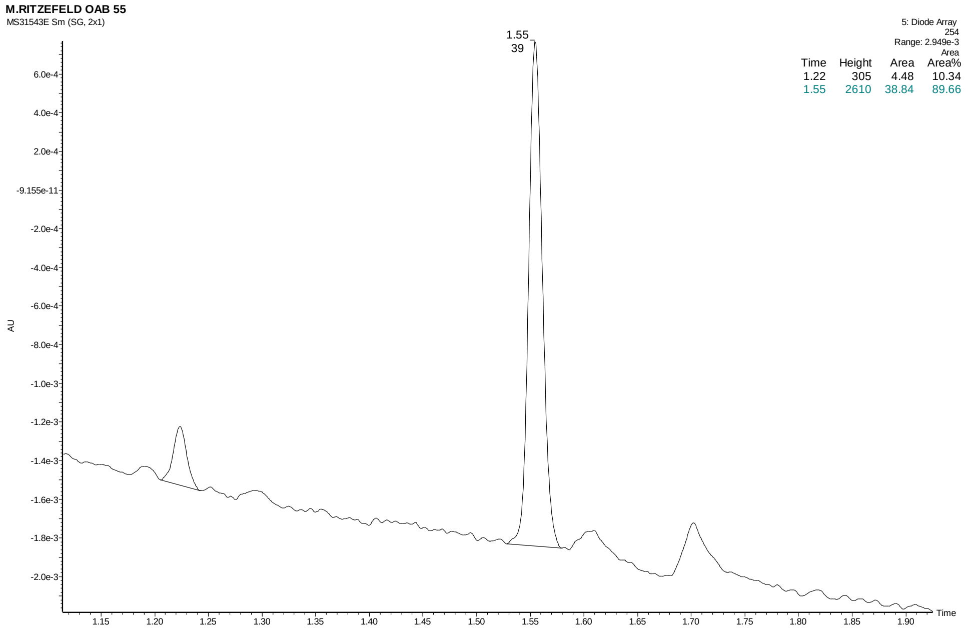

16 (Rt=3.2 min; 96.0%)

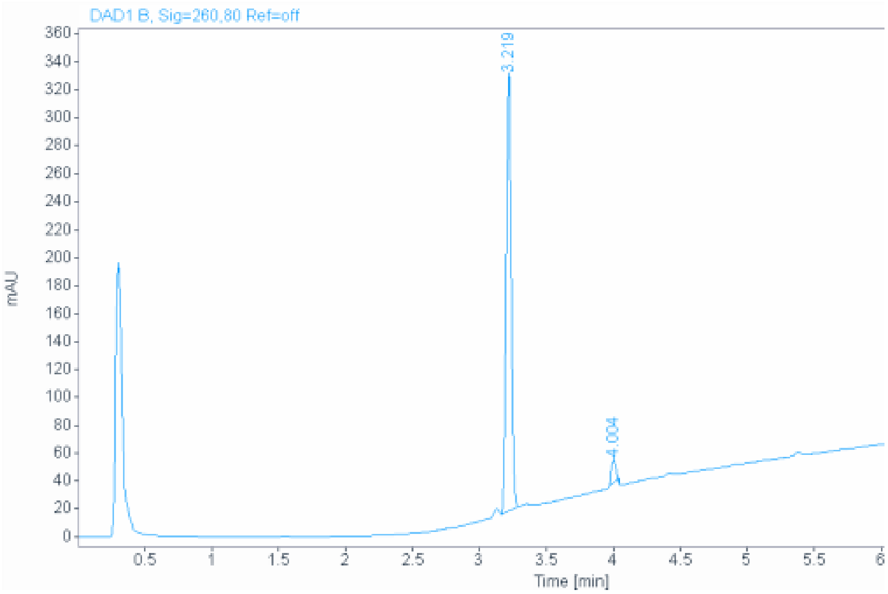

19 (Rt=1.4min; 100%)

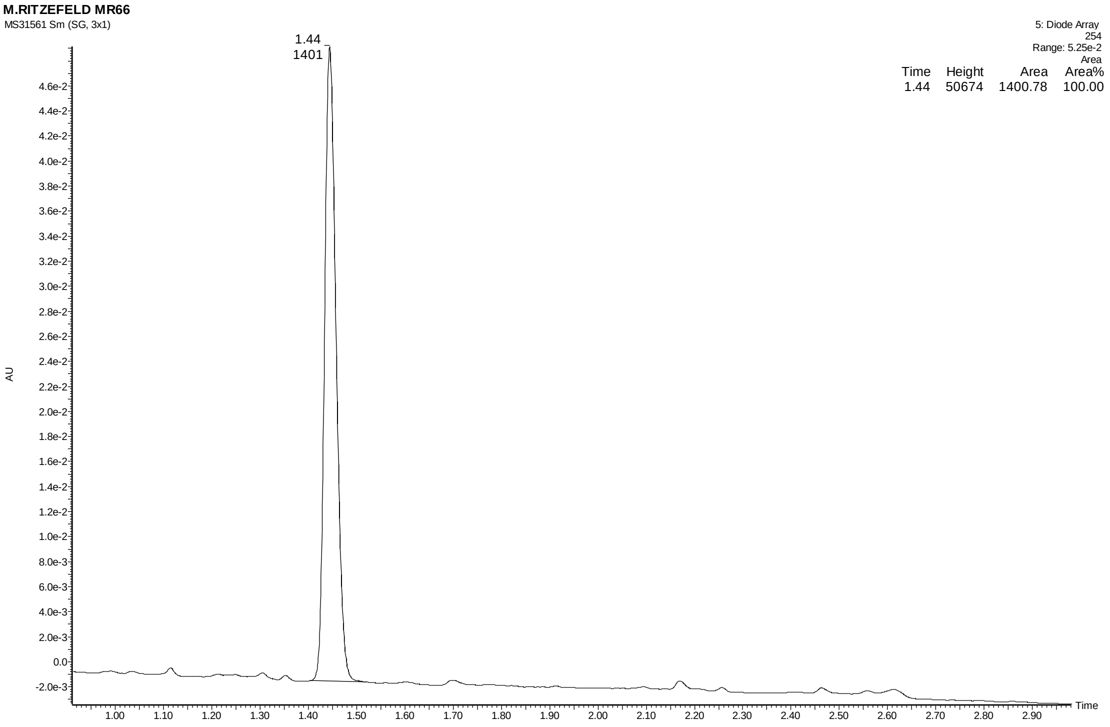

45 (Rt=4.5 min; 95.4%)

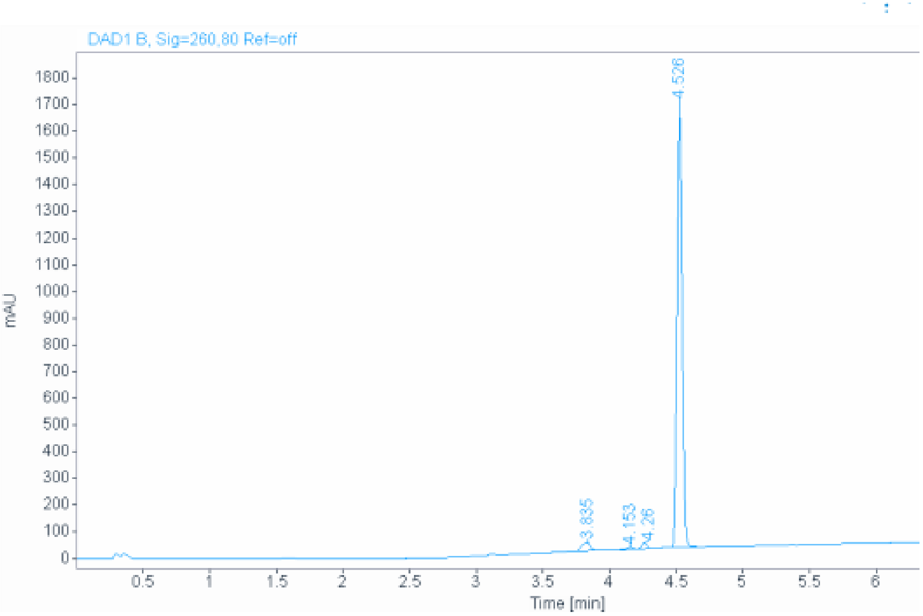

17 (Method A)

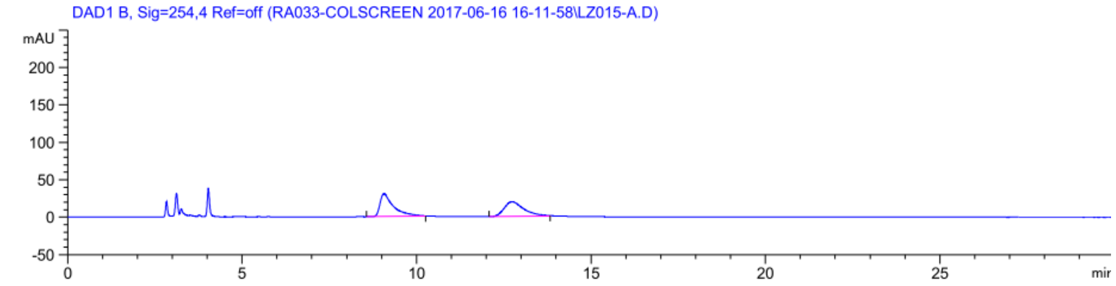

Peak #	RetTime [min]	Type	Width [min]	Area [mAU*s]	Height [mAU]	Area %
1	9.068	BB	0.3833	831.69897	30.58439	51.9091
2	12.740	BB	0.5801	770.52209	19.53974	48.0909

20 (Method A)

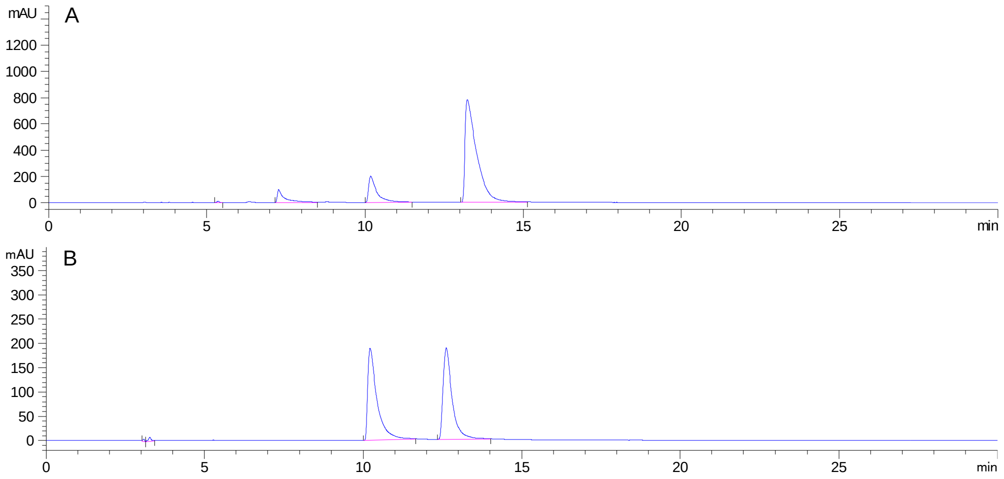

Chromatogram	Peak [min]	Area [%]
**A (20-S)**	10.2	16
	13.2	84
**B (20)**	10.2	50
	12.6	

22 and **23 (Method A)**

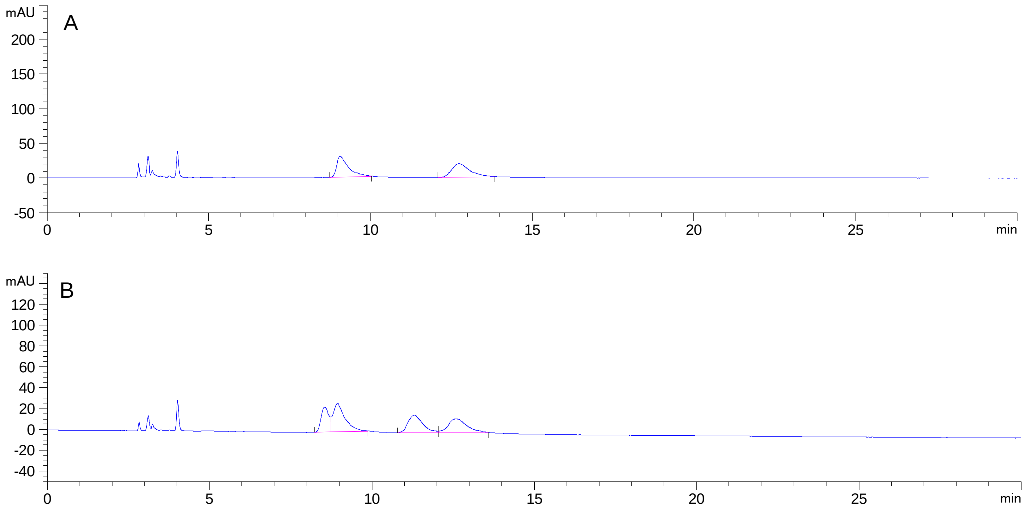

Chromatogram	Peak [min]	Area [%]
**A (23)**	9.1	50
	12.7	50
**B (22)**	8.6	50
	8.9	
	11.3	25
	12.6	25

25 (Method B)

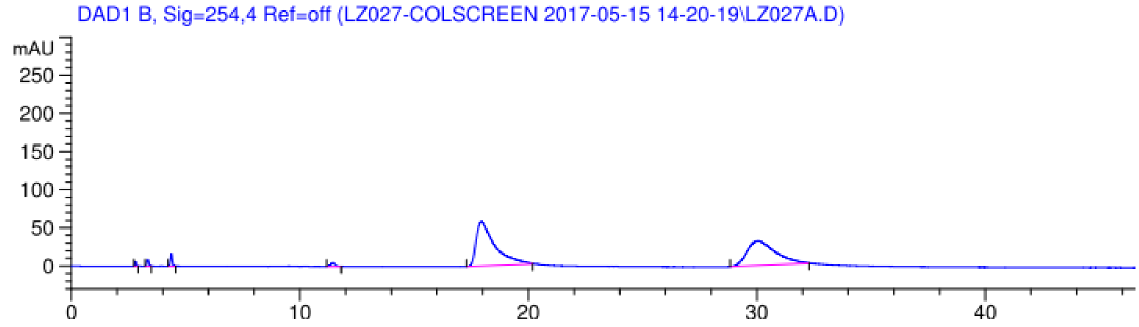

Peak #	RetTime [min]	Type	Width [min]	Area [mAU*s]	Height [mAU]	Area %
1	3.354	BV	0.0948	51.37249	8.70924	0.8130
2	4.387	BB	0.0839	91.74111	16.74321	1.4518
3	17.942	BB	0.7923	3351.63818	58.50987	53.0384
4	30.037	BB	1.0366	2824.51001	32.16299	44.6968

24 (Method A)

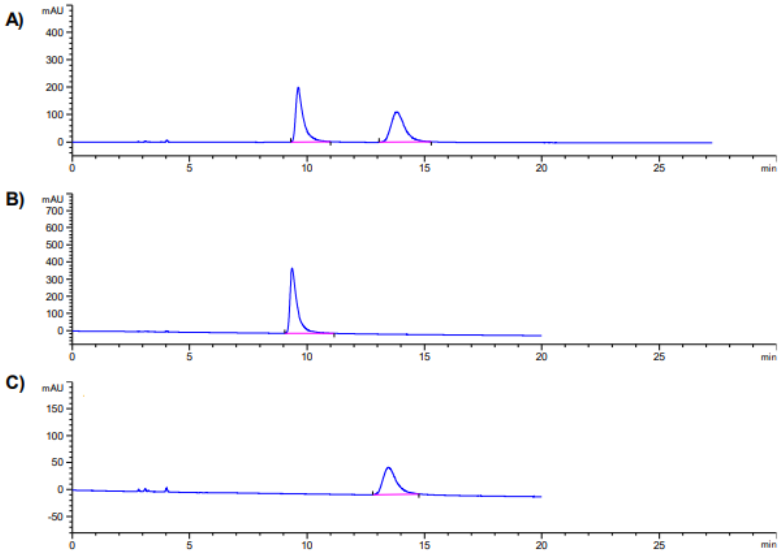

Chromatogram	Peak [min]	Area [%]
**A (24)**	9.6	50
	13.8	50
**B (30)**	9.4	100
**C (28)**	13.8	100

27 (Method C)

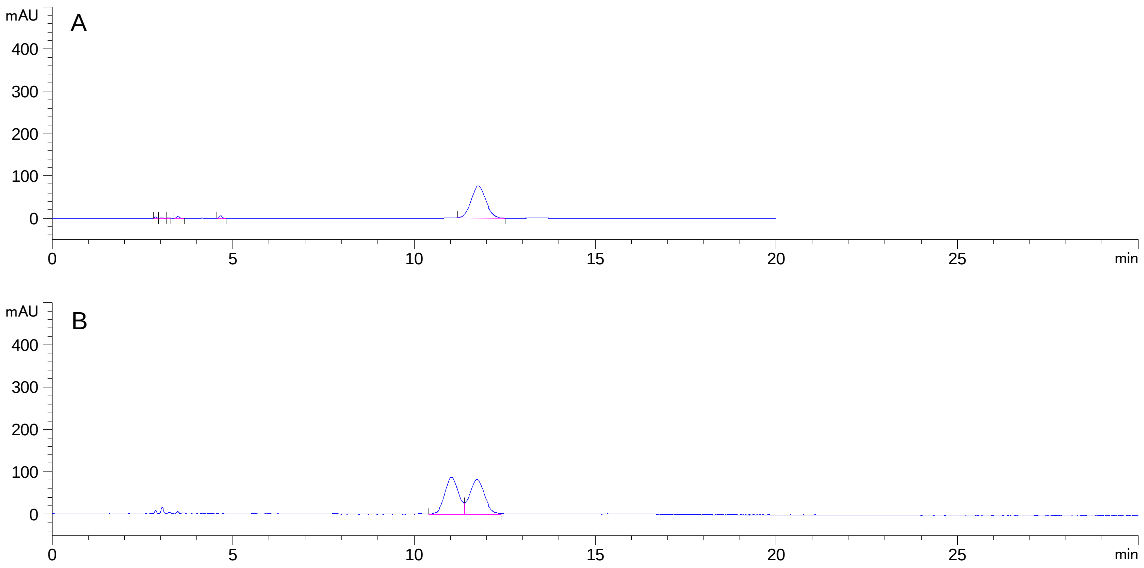

Chromatogram	Peak [min]	Area [%]
**A (27)**	11.8	100
**B (21)**	11.0	50
	11.7	50

29 (Method C)

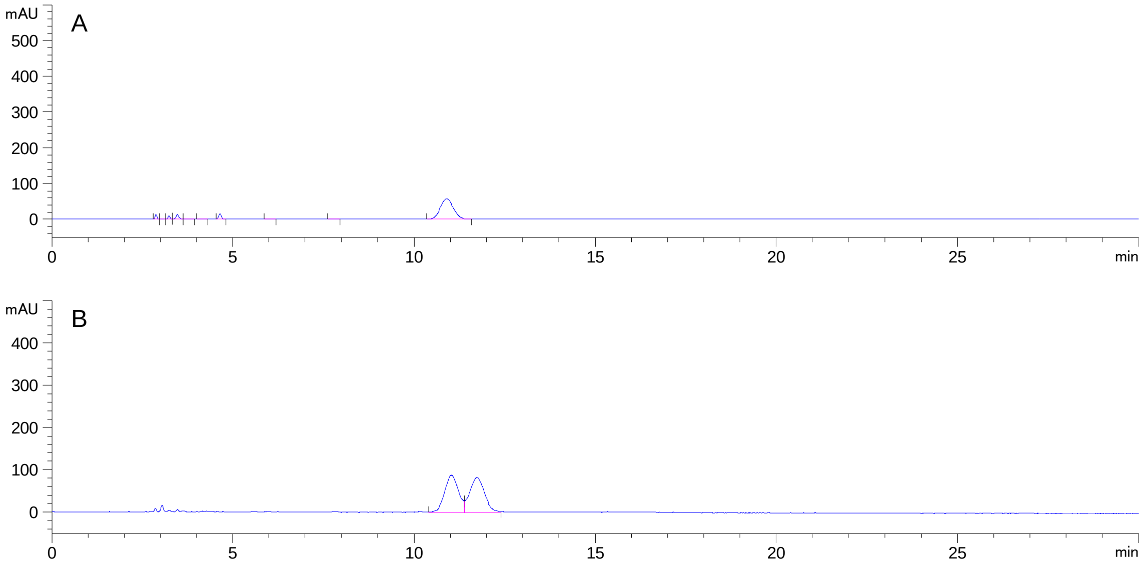

Chromatogram	Peak [min]	Area [%]
**A (29)**	10.9	100
**B (21)**	11.0	50
	11.7	50

42 (Method A)

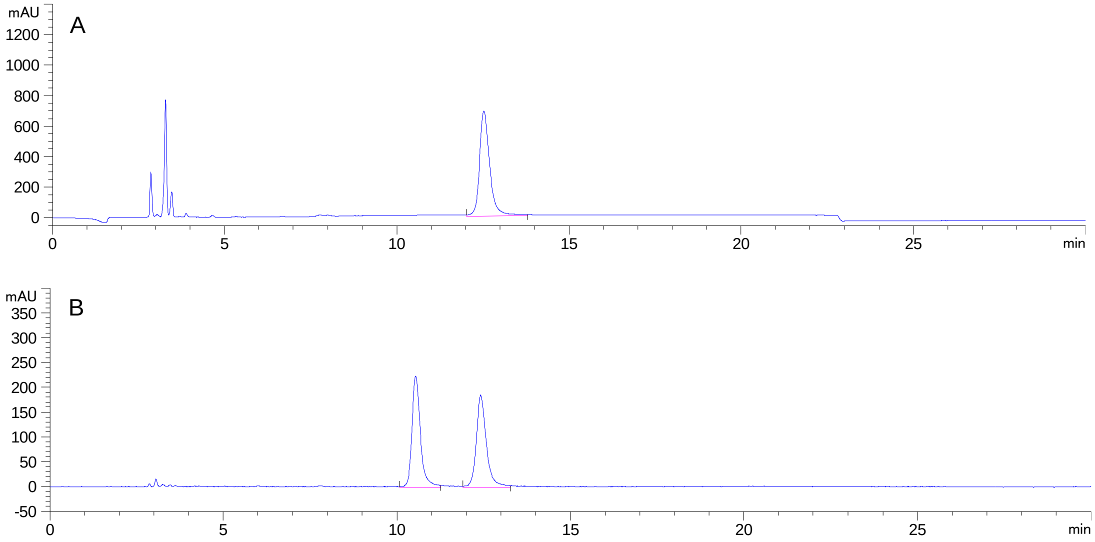

Chromatogram	Peak [min]	Area [%]
**A (42)**	12.5	100
**B (39)**	10.5	50
	12.4	50

43 (Method A)

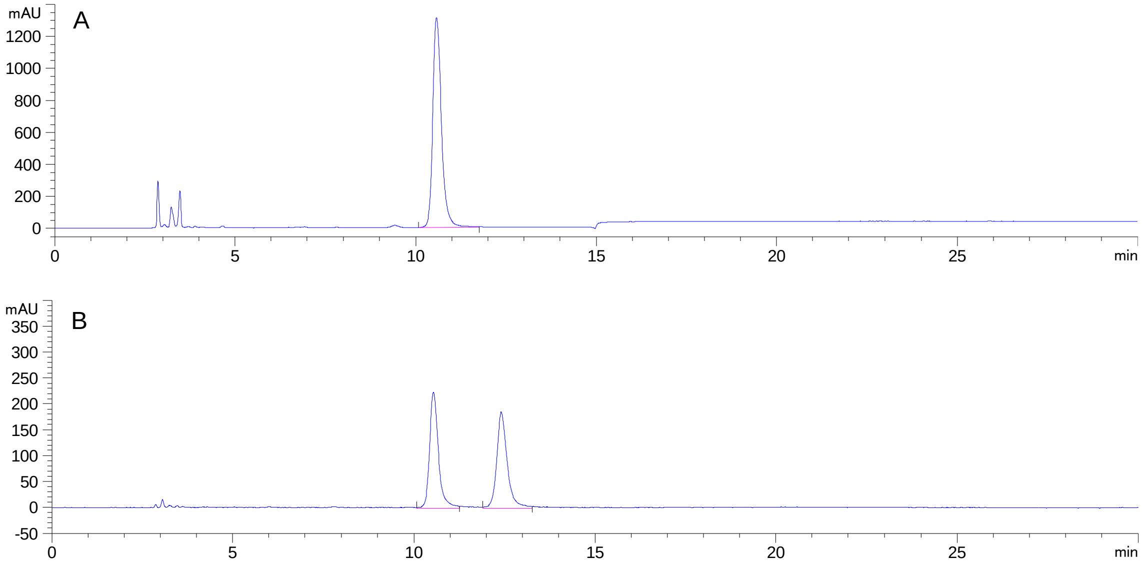

Chromatogram	Peak [min]	Area [%]
**A (43)**	10.6	100
**B (39)**	10.5	50
	12.4	50

10. Conformational Characteristics

Table S8. Observed *E/Z* rotamer ratios of selected HHAT inhibitor analogues in ^{1}H NMR at 298K. 2-Substituted *N*-acyl-piperidines can display unequal distributions of *E*- or *Z*-amide conformational rotamers in ^{1}H NMR due to non-covalent interactions between the amide oxygen and the adjacent 2-substituent.[2]

Compound No	*E/Z* ratio	Solvent
**2**[1]	**50 : 50**	$CDCl_3$
**3**[1]	**40 : 60**	$CDCl_3$
**5**[2]	**22 : 78**	$CDCl_3$
**6**[2]	**24 : 75**	$CDCl_3$
**16**[2]	**29 : 71**	$CDCl_3$
**17**[2]	**20 : 80**	$CDCl_3$
**18**[2]	**48 : 52**	$CDCl_3$
**19**	**46 : 54**	$CDCl_3$
**21**	**64 : 36**	$CDCl_3$
**22**[1]	**70 : 30**	$CDCl_3$
**23**	**66 : 34**	$CDCl_3$
**24**	**65 : 35**	$CDCl_3$
**25**	**67 : 33**	$CDCl_3$
**26**	**100 : 0**	$CDCl_3$
**27**	**63 : 37**	$CDCl_3$
**28**	**63 : 37**	$CDCl_3$
**29**	**62 : 38**	$CDCl_3$
**30**	**64 : 36**	$CDCl_3$
**31**[2]	**80 : 20**	$CDCl_3$
**32**	**73 : 27**	$CDCl_3$
**33**[2]	**0 : 100**	$CDCl_3$
	**40 : 60**	DMSO-d6
**34**	**53 : 47**	$CDCl_3$
**35**[2]	**57 : 43**	$CDCl_3$
**36**	**62 : 38**	$CDCl_3$
**37**	**80 : 20**	$CDCl_3$
**38**	**78 : 21**	$CDCl_3$
**39**	**86 : 14**	$CDCl_3$
**40**	**100 : 0**	$CDCl_3$
**41**	**100 : 0**	$CDCl_3$
**42**	**78 : 22**	$CDCl_3$
**43**	**78 : 22**	$CDCl_3$
**44**	**82 : 18**	$CDCl_3$
**45**	**80 : 20**	$CDCl_3$
**46**	**30 : 70**	$(CD_3)_2CO$
**47**	**52 : 48**	$CDCl_3$
**48**	**49 : 51**	$CDCl_3$
**49**	**54 : 46**	$CDCl_3$
**50**	**59 : 41**	$CDCl_3$

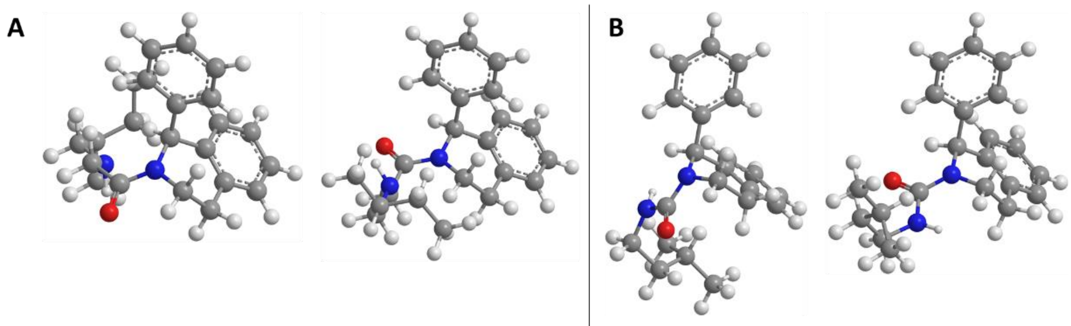

Conformation	*E*-38	*Z*-38	*E*-40	*Z*-40
**Energy** [kcal/mol]	**0**	**0.32**	**0**	**1.5**

**Compound No**	**Calculated *E/Z***	**Observed *E/Z***
**38**	75 : 25	78 : 21
**40**	83 : 17	100 : 0

Figure S9. Effect of non-covalent interactions on observed *E*/*Z* amide rotamers in ^{1}H NMR. Computed lowest energy conformations for exemplar 1,2,3,4-tetrahydroisoquinolines **38 (A)** and **40 (B)**, and comparison of calculated and observed amide *E/Z* ratios in ^{1}H NMR at 298 K. Lowest energy conformations are calculated by density functional theory (DFT) modelling as previously described. [2]

11. NMR Spectra

(2*S*)-2-amino-1-(4-phenyl-6,7-dihydrothieno[3,2-*c*]pyridin-5(4H)-yl)propan-1-one (7)

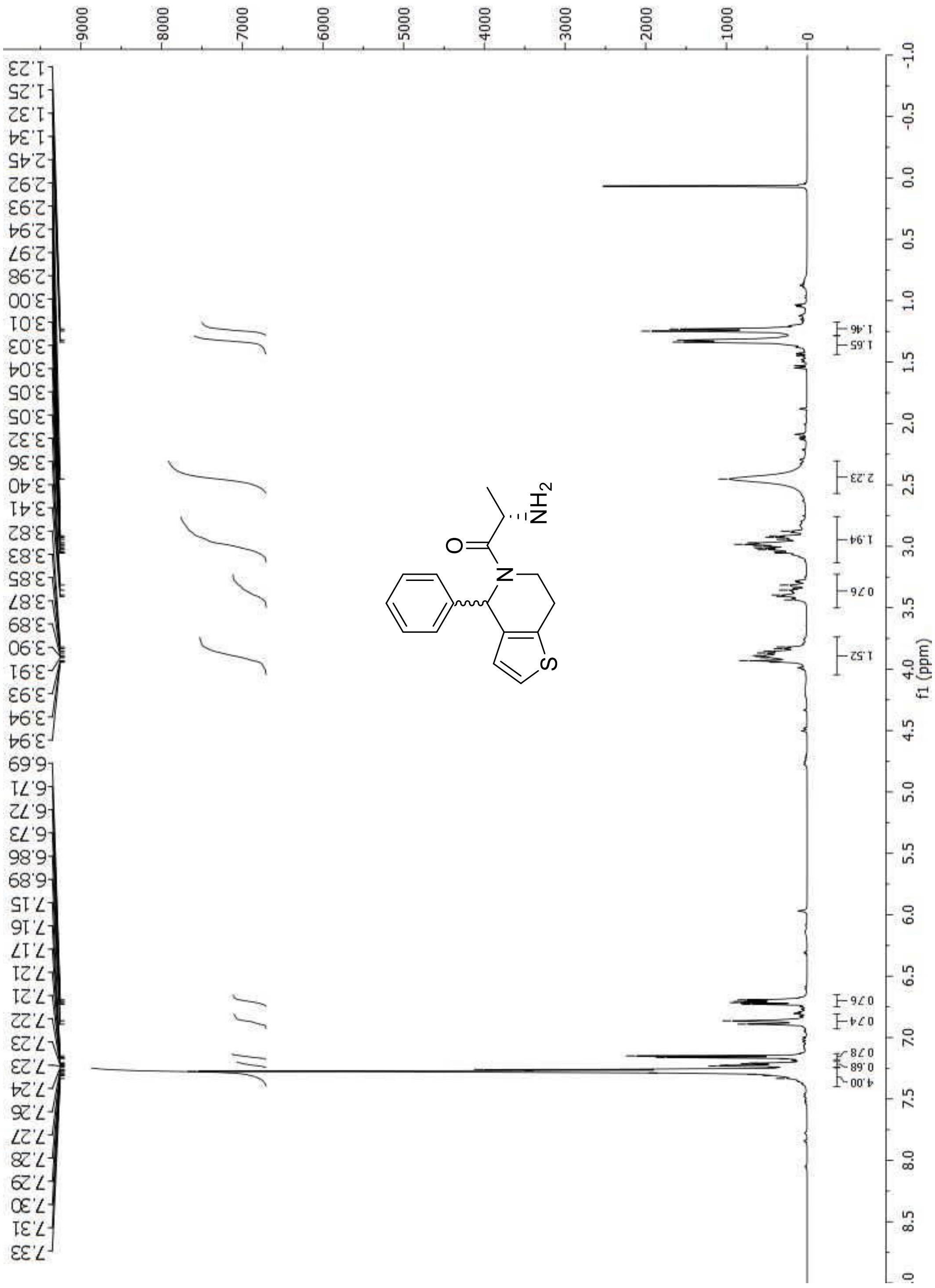

Figure S19. ^{1}H NMR (400 MHz, $CDCl_3$, 298K) of **7**.

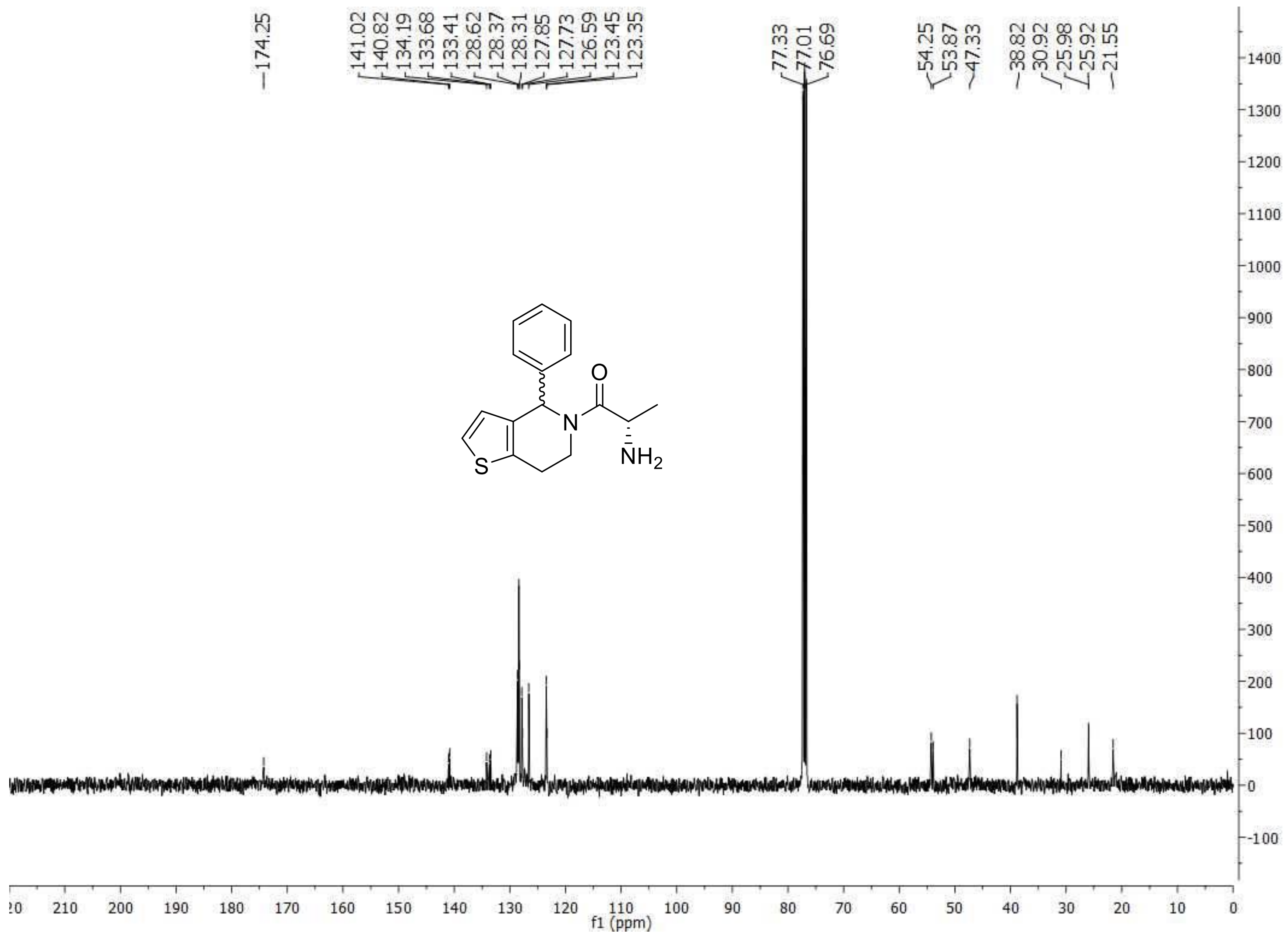

Figure S20. ^{13}C NMR (400 MHz, $CDCl_3$, 298K) of **7**.

(2*S*)-2-amino-3-methyl-1-(4-phenyl-6,7-dihydrothieno[3,2-*c*]pyridin-5(4H)-yl)butan-1-one (8)

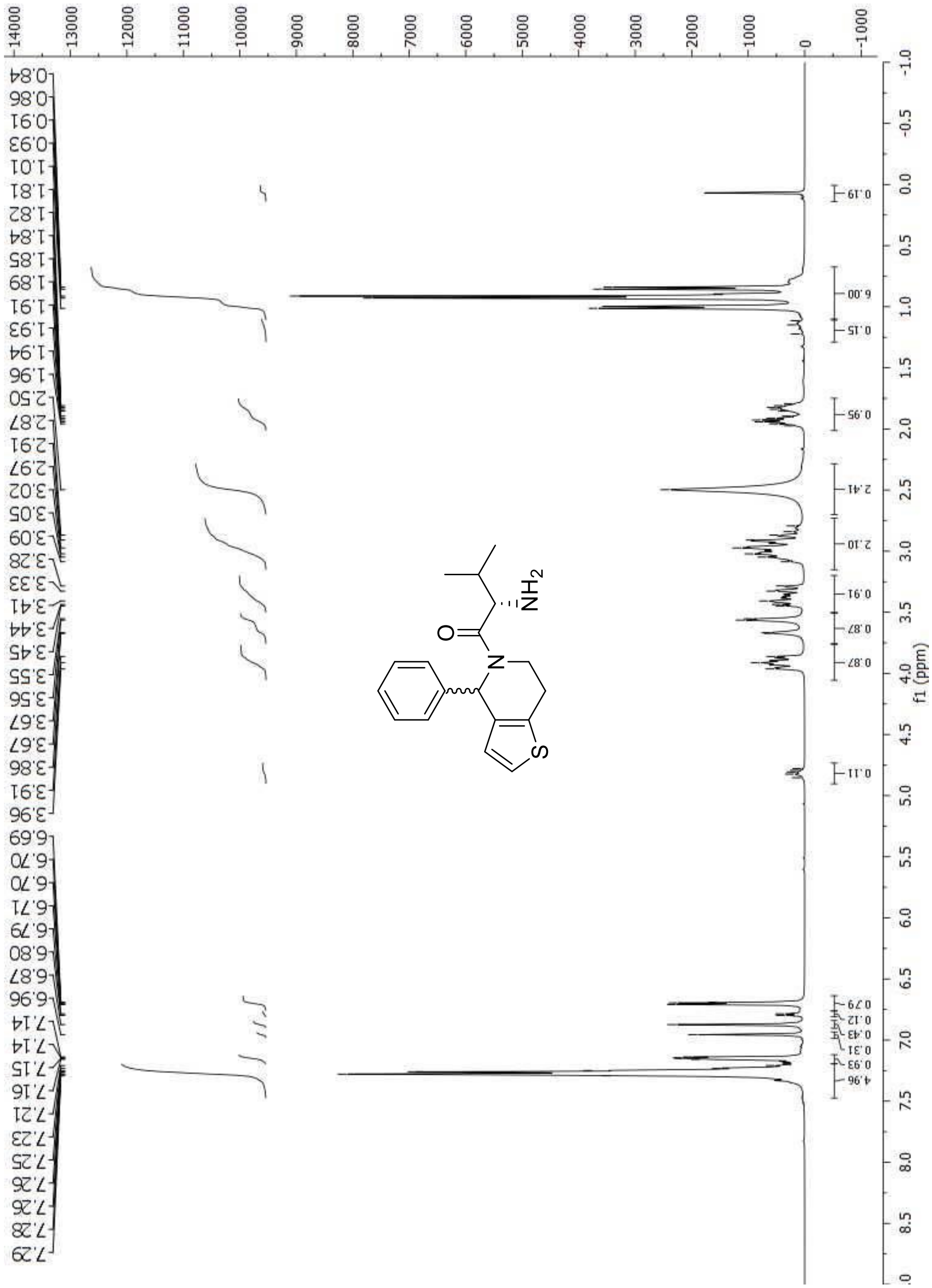

Figure S21. ^{1}H NMR (400 MHz, $CDCl_3$, 298K) of **8**.

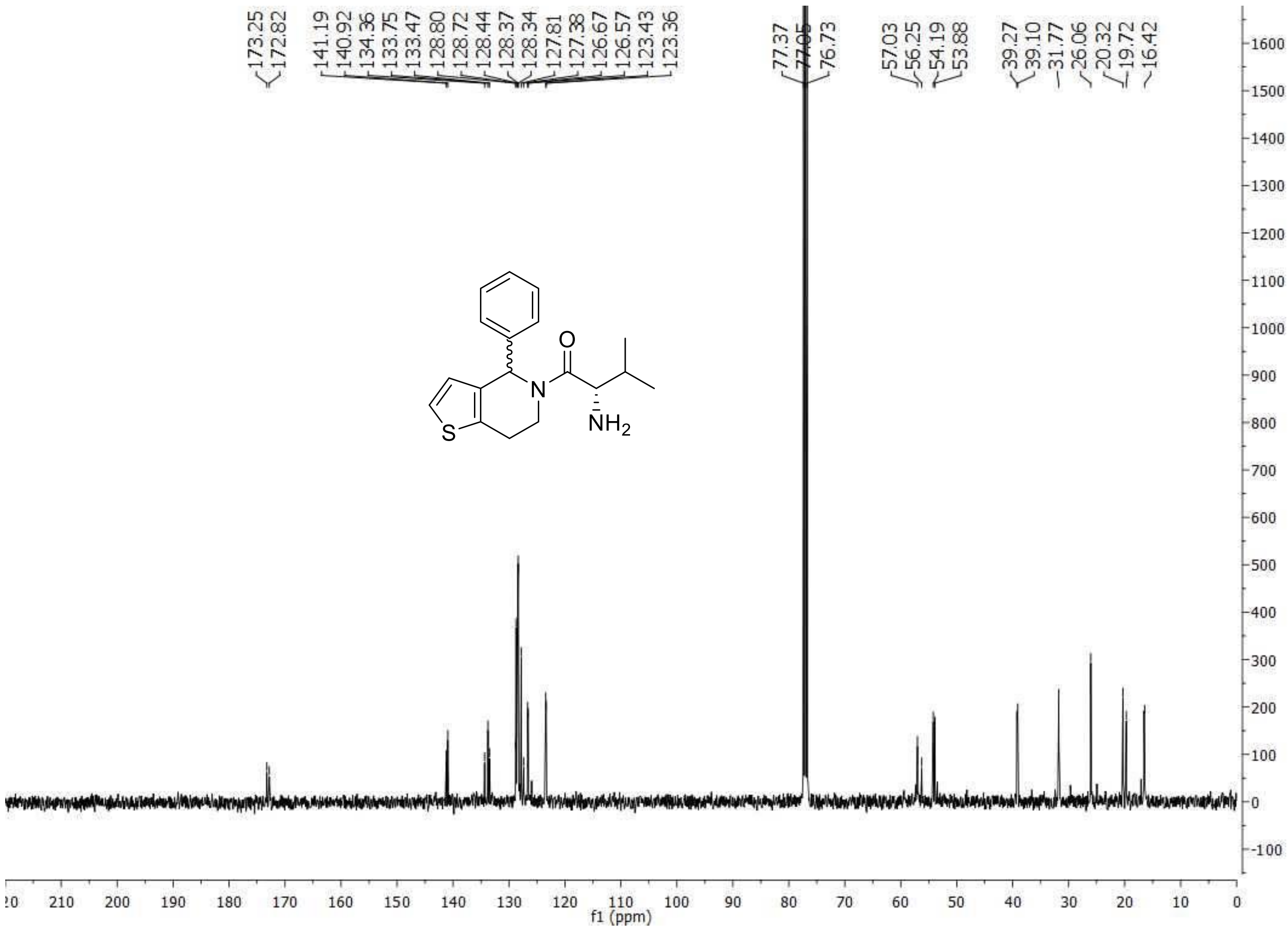

Figure S22. ^{1}H NMR (400 MHz, $CDCl_3$, 298K) of **8**.

(*S*)-4-phenyl-5-prolyl-4,5,6,7-tetrahydrothieno[3,2-*c*]pyridine (9)

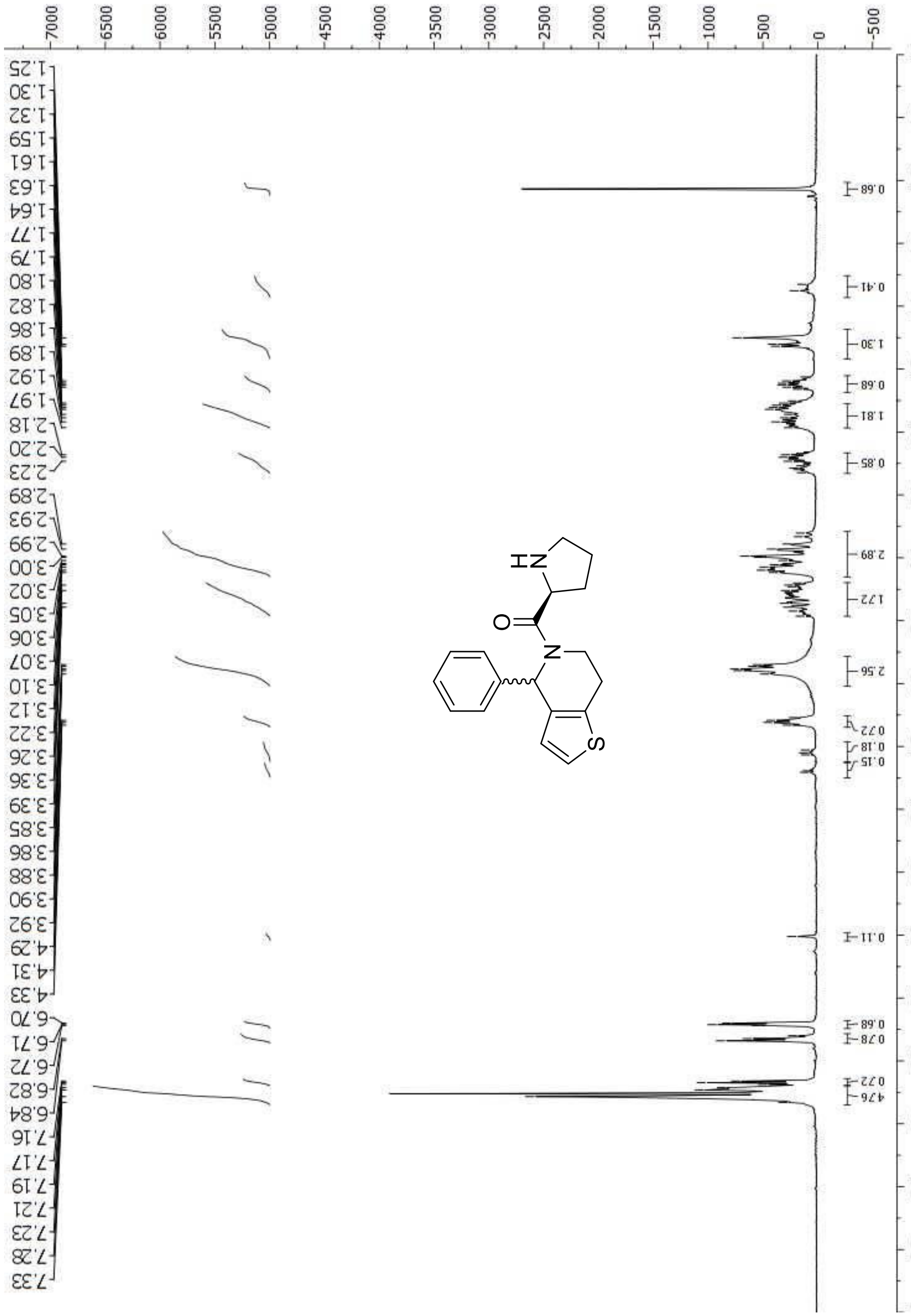

Figure S23. ^{1}H NMR (400 MHz, $CDCl_3$, 298K) of **9**.

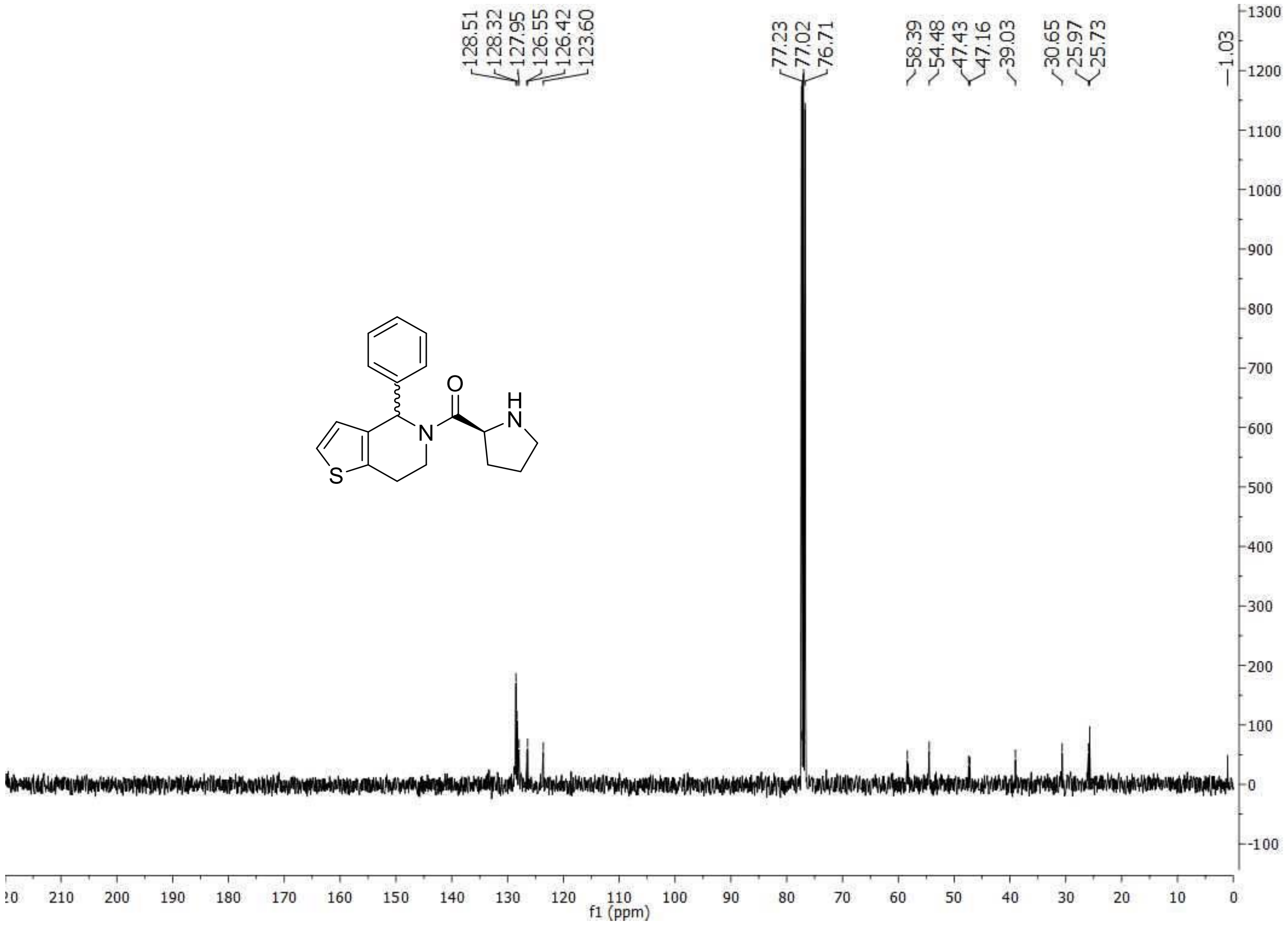

Figure S24. ^{13}C NMR (400 MHz, $CDCl_3$, 298K) of **9**.

(2*S*)-2-amino-2-phenyl-1-(4-phenyl-6,7-dihydrothieno[3,2-*c*]pyridin-5(4*H*)-yl)ethan-1-one (10)

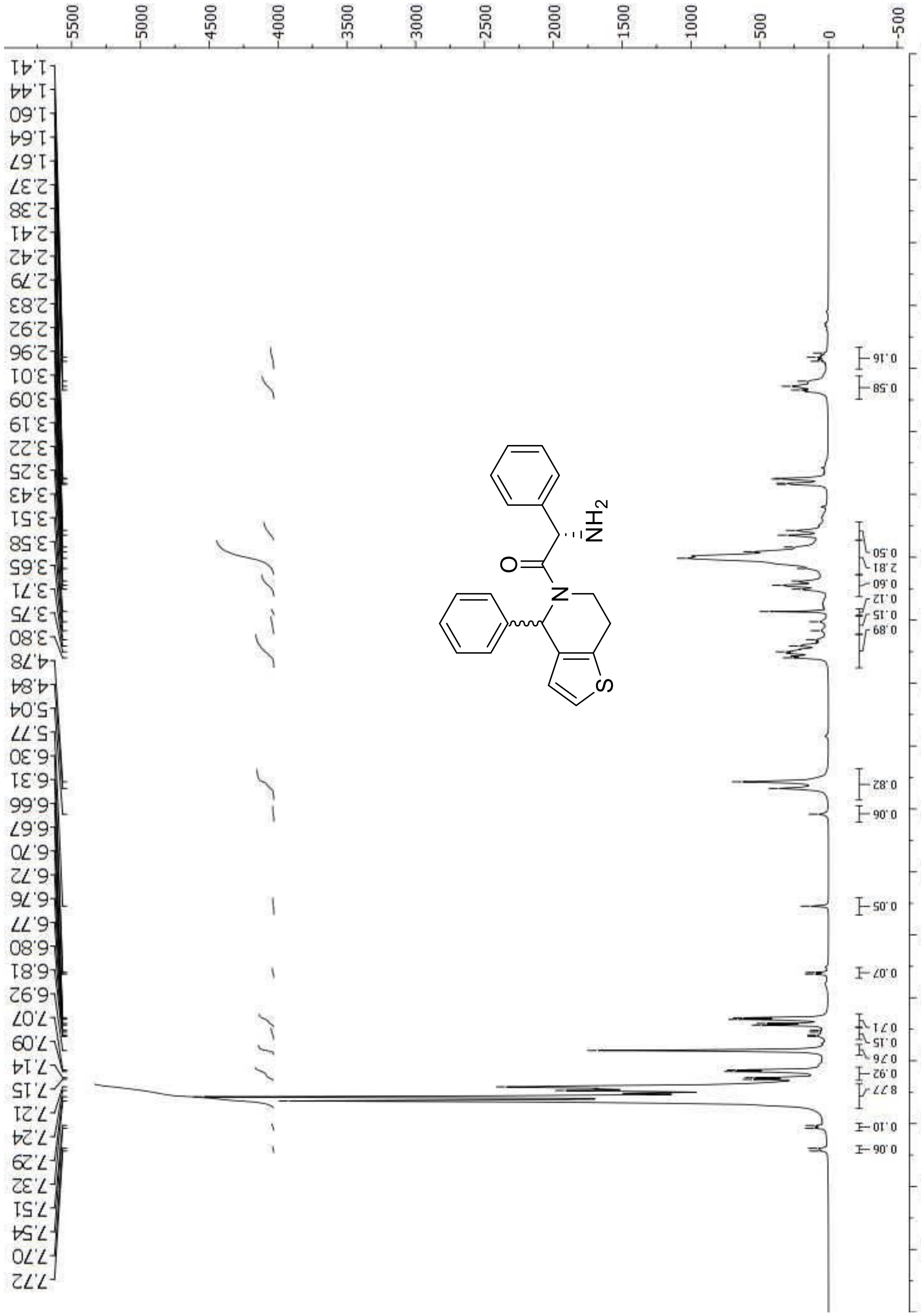

Figure S25. ^{1}H NMR (400 MHz, $CDCl_3$, 298K) of **10**.

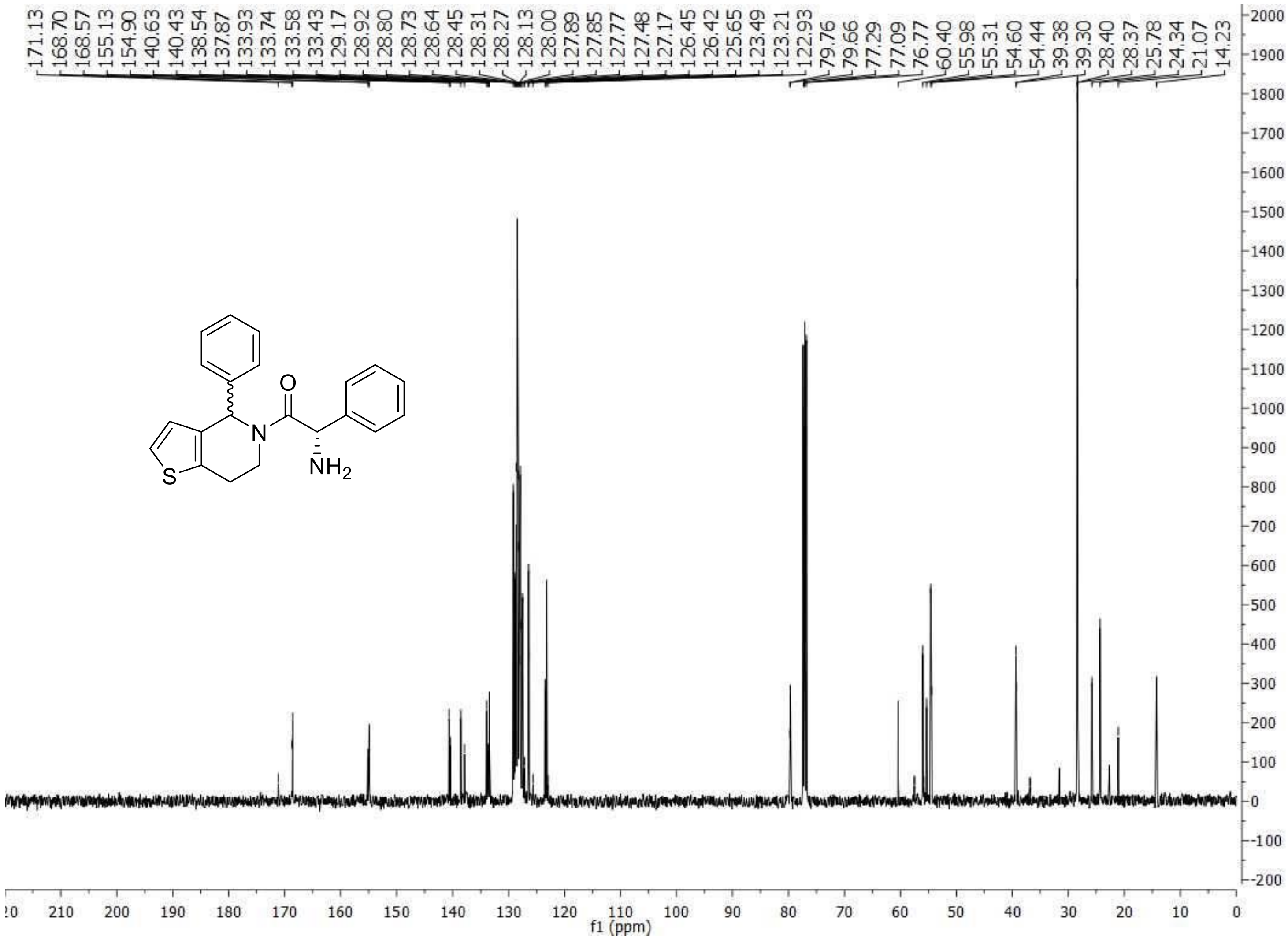

Figure S26. ^{13}C NMR (400 MHz, $CDCl_3$, 298K) of **10**.

(2S)-2-amino-3-phenyl-1-(4-phenyl-6,7-dihydrothieno[3,2-*c*]pyridin-5(4*H*)-yl)propan-1-one (11)

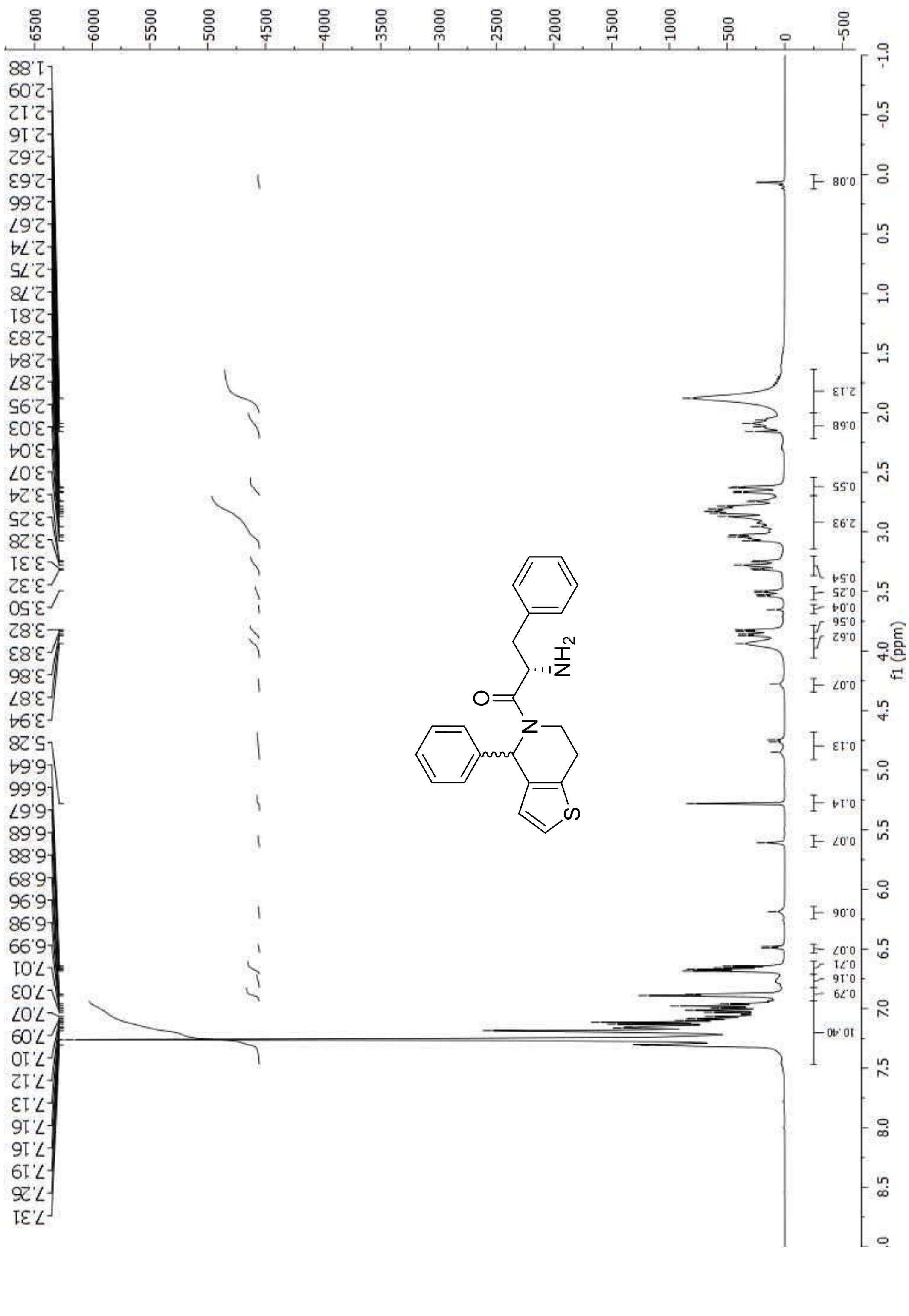

Figure S27. ^{1}H NMR (400 MHz, $CDCl_3$, 298K) of **11**.

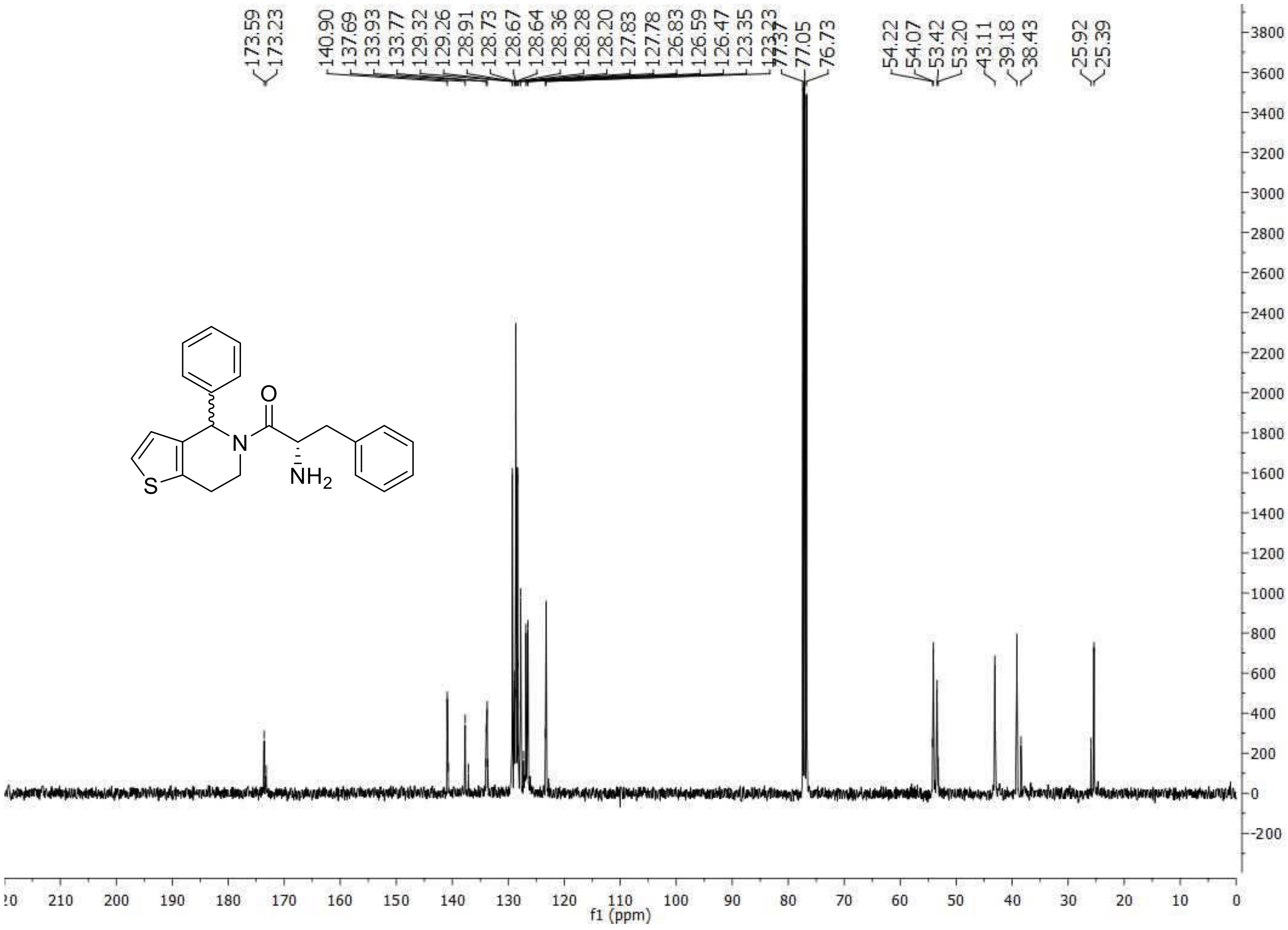

Figure S28. ^{13}C NMR (400 MHz, $CDCl_3$, 298K) of **11**.

(2-(allylamino)-1-(4-phenyl-6,7-dihydrothieno[3,2-*c*]pyridin-5(4*H*)-yl)ethan-1-one (12)

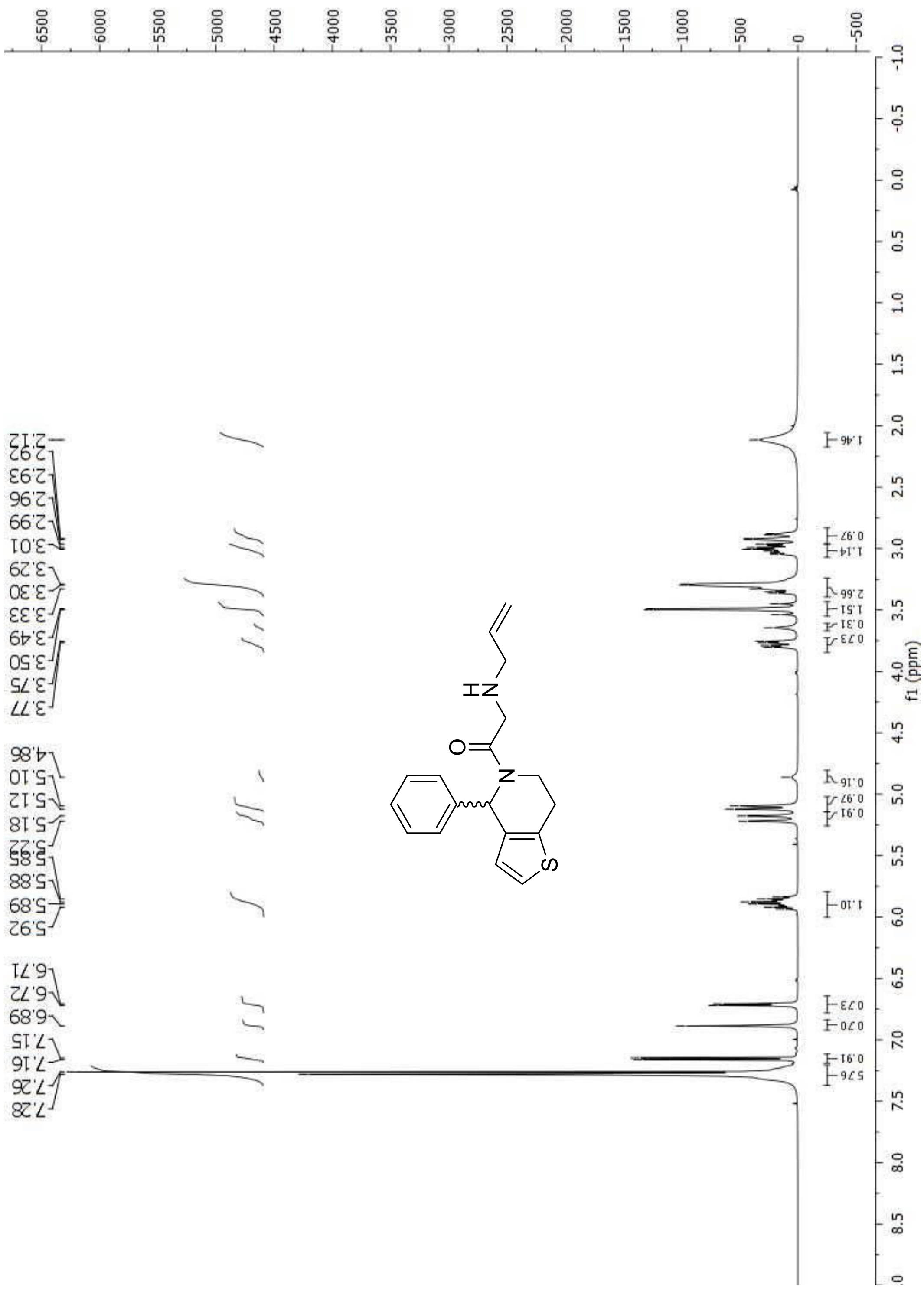

Figure S29. ^{1}H NMR (400 MHz, $CDCl_3$, 298K) of **12**.

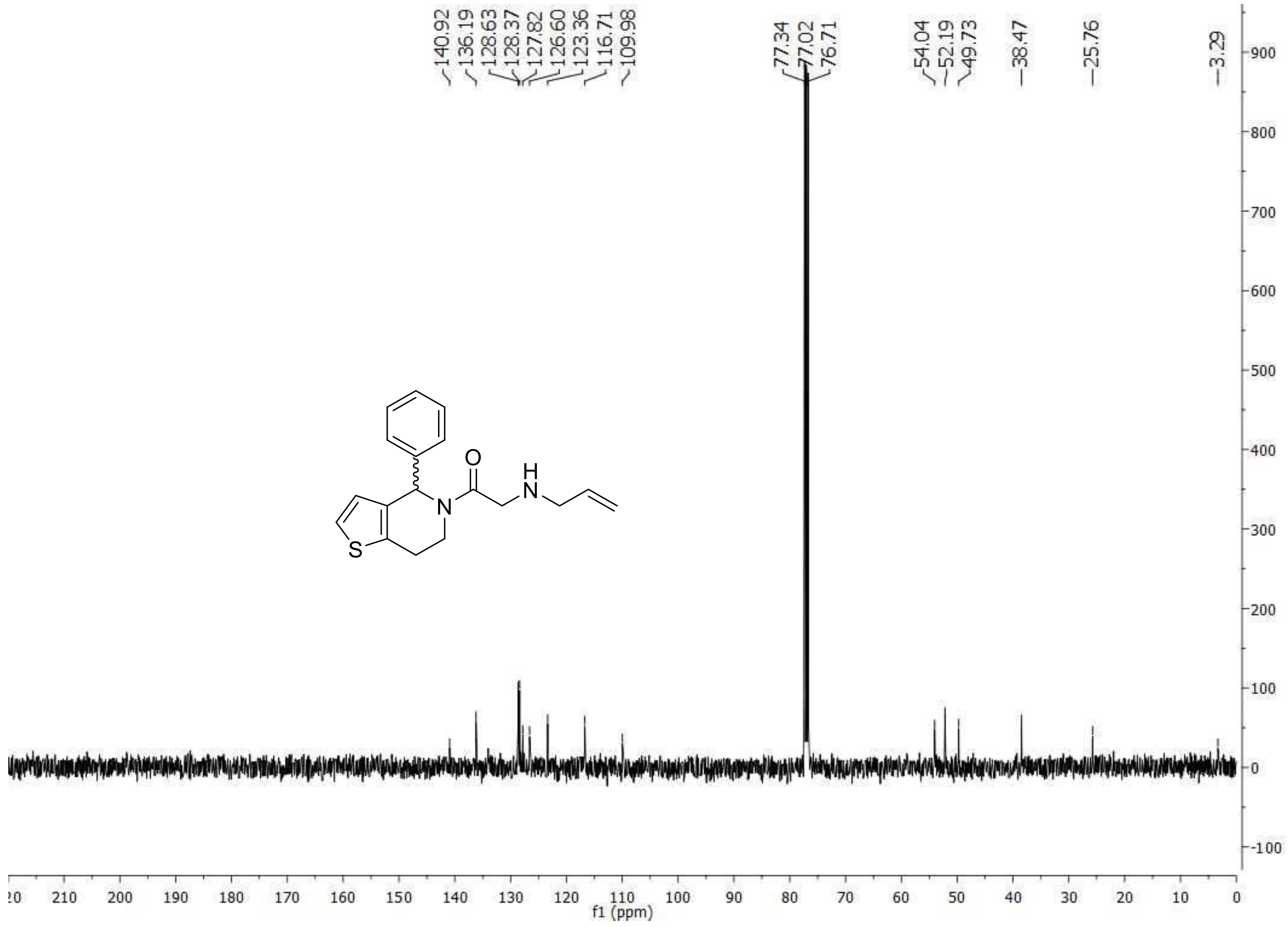

Figure S30. ^{13}C NMR (400 MHz, $CDCl_3$, 298K) of **12**.

1-(4-phenyl-6,7-dihydrothieno[3,2-*c*]pyridin-5(4H)-yl)octan-1-one (13)

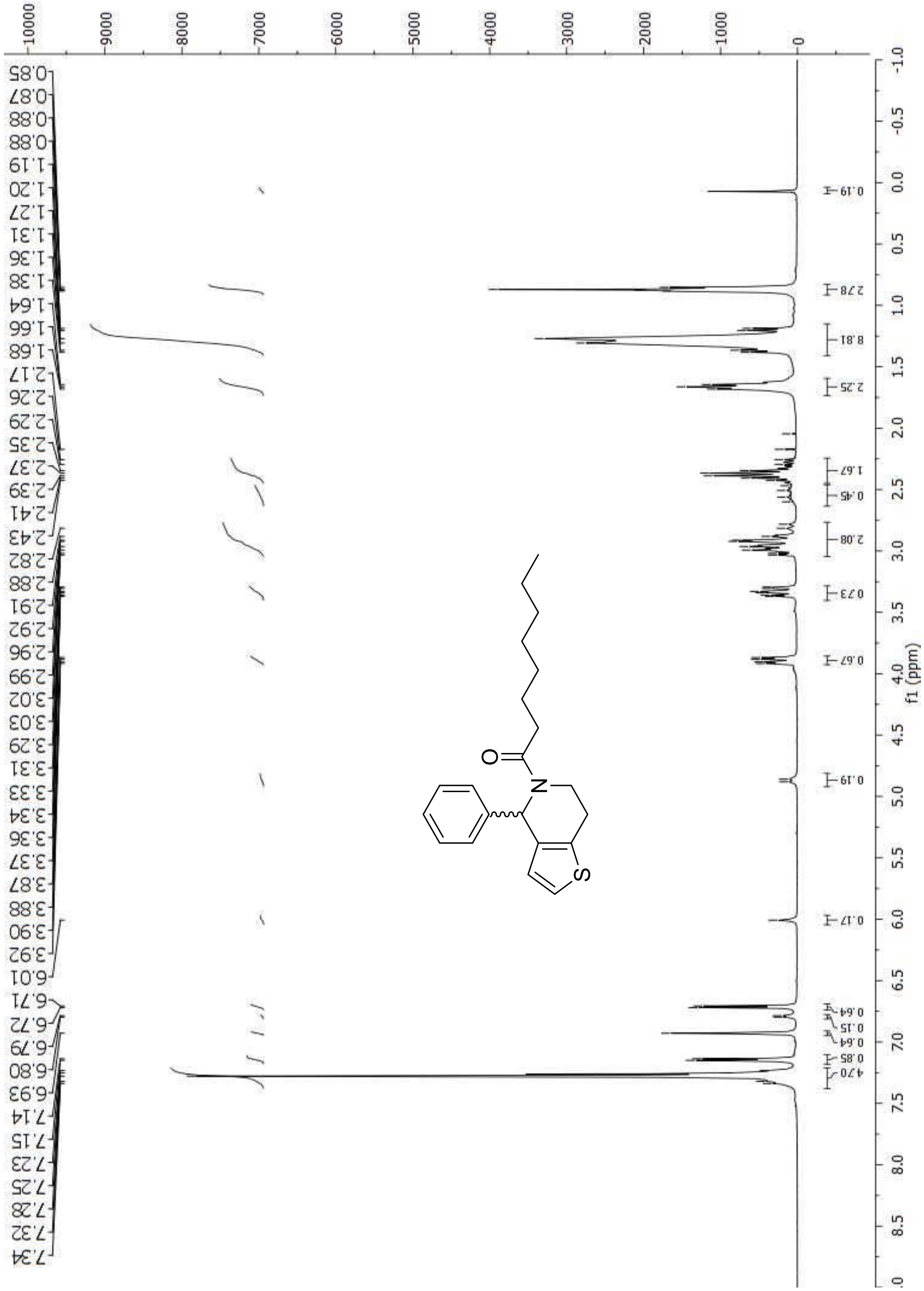

Figure S31. ^1H NMR (400 MHz, $CDCl_3$, 298K) of **13**.

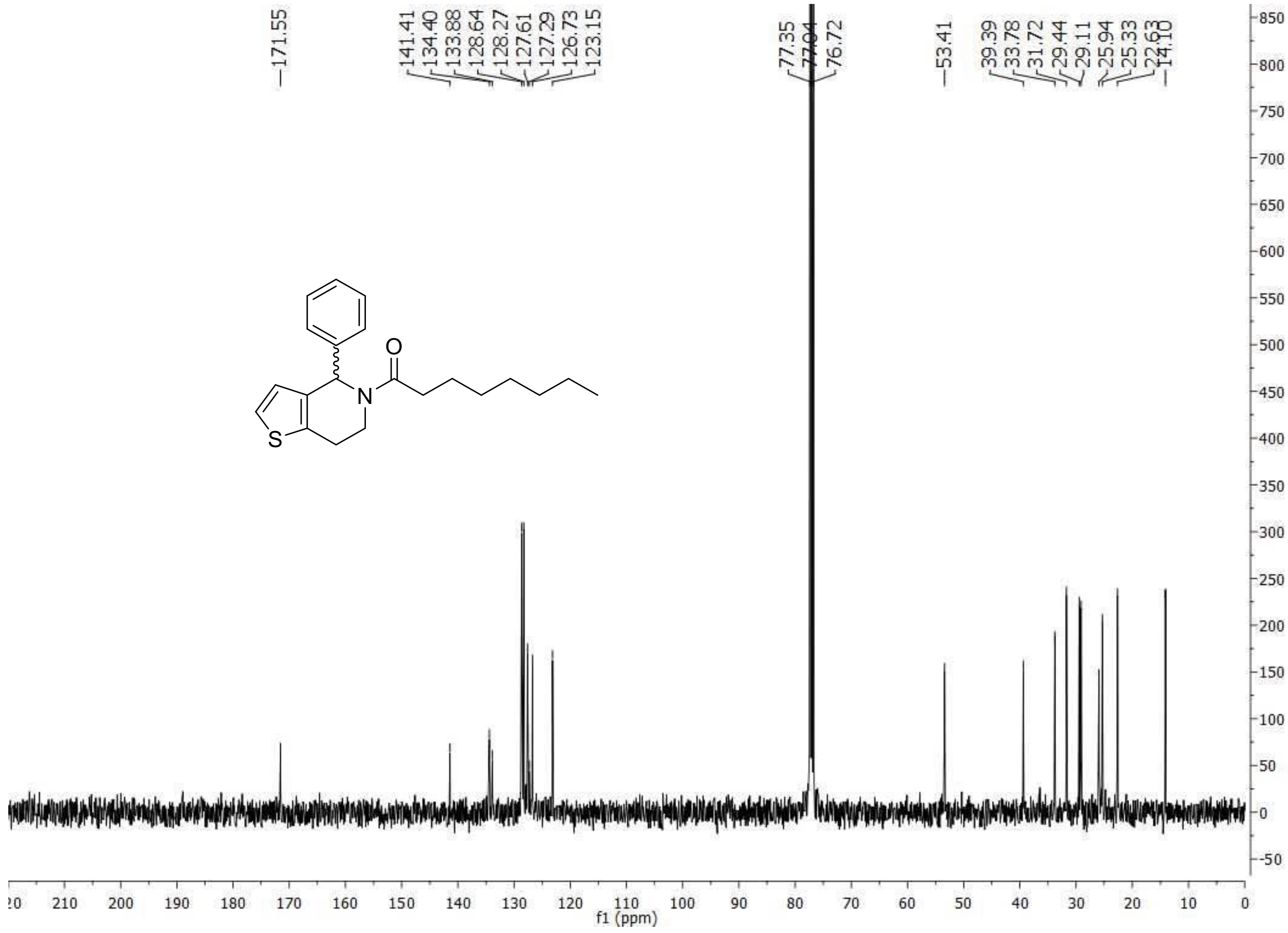

Figure S32. ^{13}C NMR (400 MHz, $CDCl_3$, 298K) of **13**.

2-(2-methylbutylamino)-1-[4-(6-methylpyridin-2-yl)-6,7-dihydro-4H-thieno[3,2-*c*]pyridin-5-yl]ethanone (19)

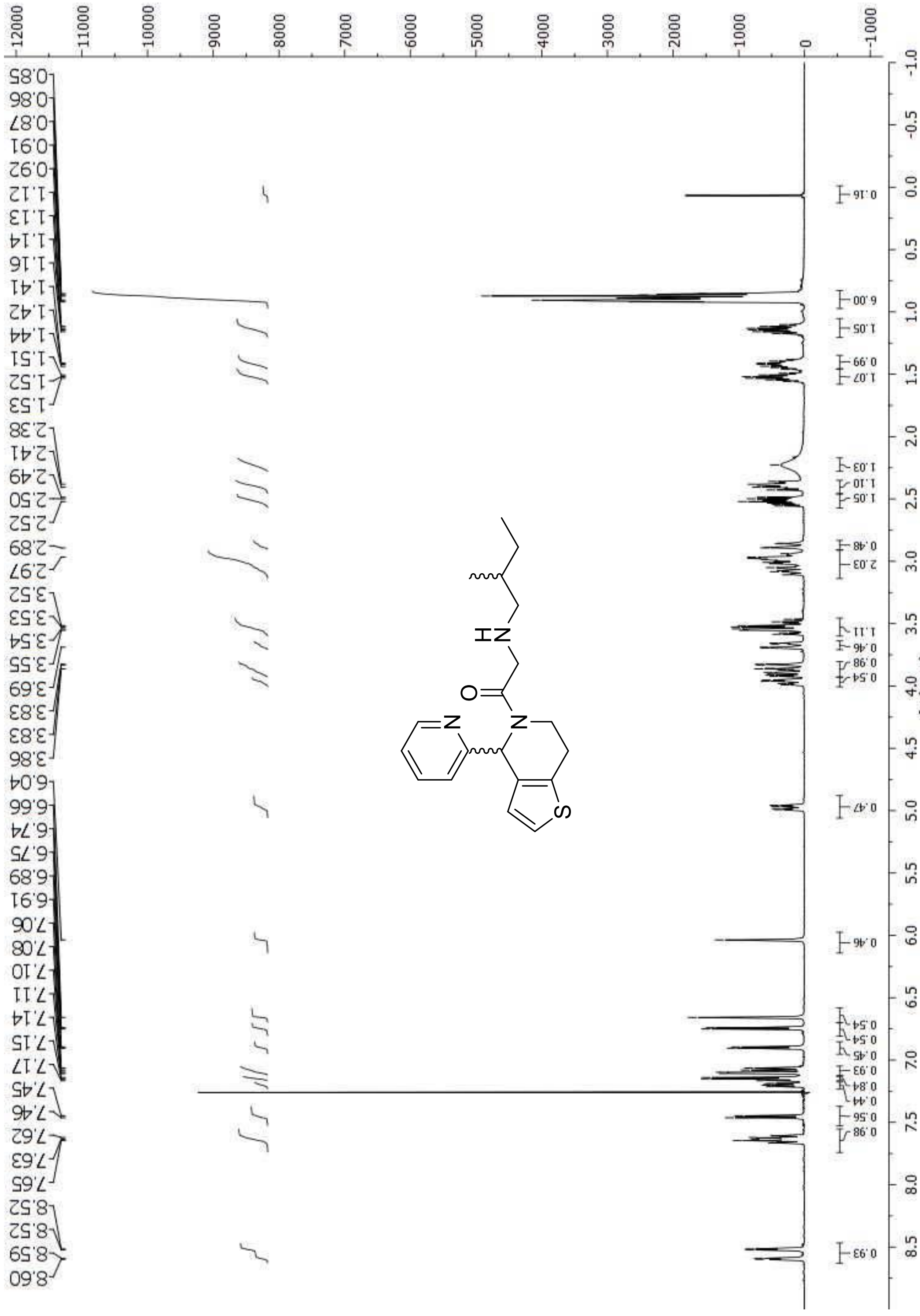

Figure S33. ^{1}H NMR (500 MHz, $CDCl_3$, 298K) of **19**.

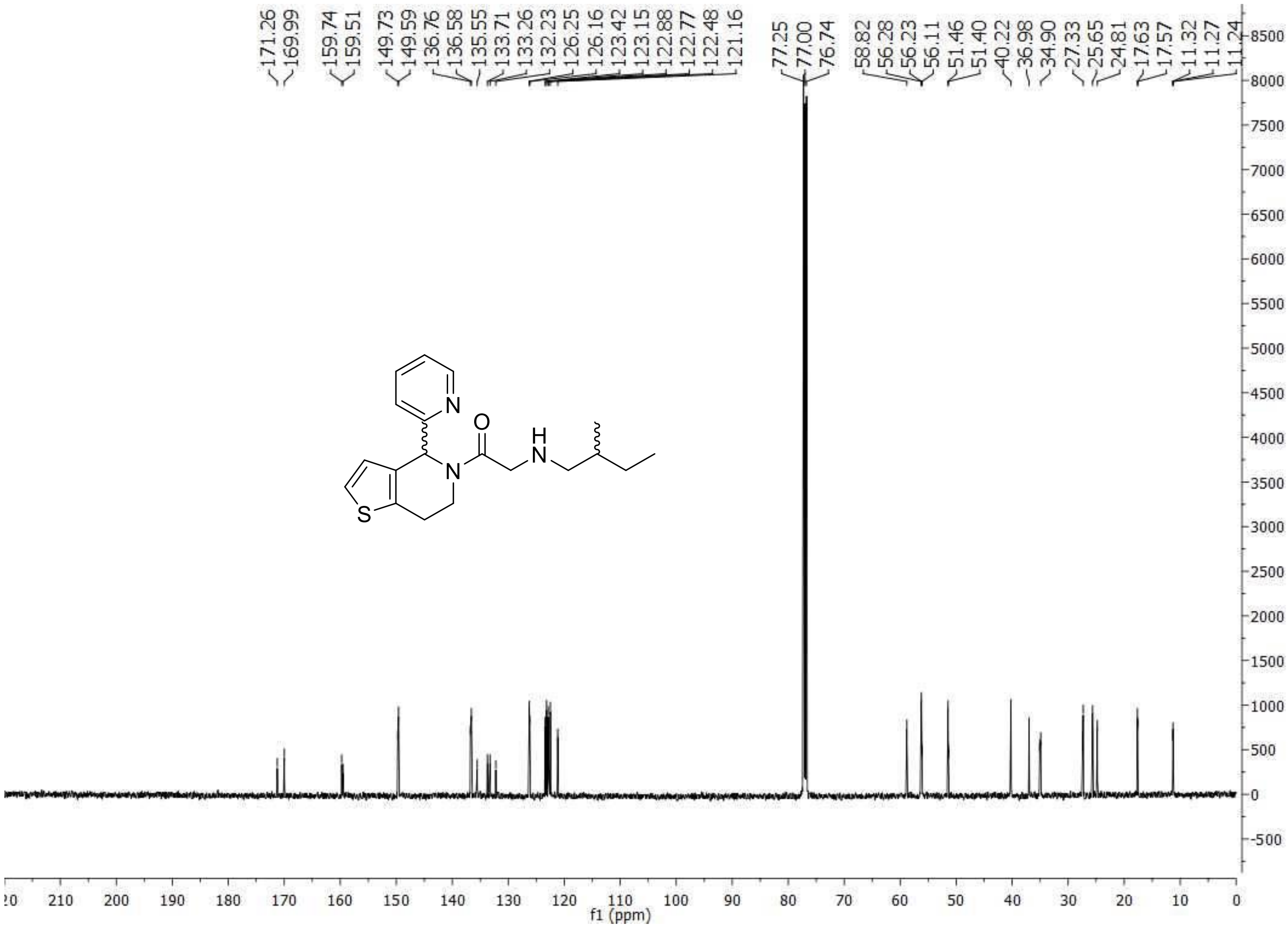

Figure S34. ^{13}C NMR (500 MHz, $CDCl_3$, 298K) of **19**.

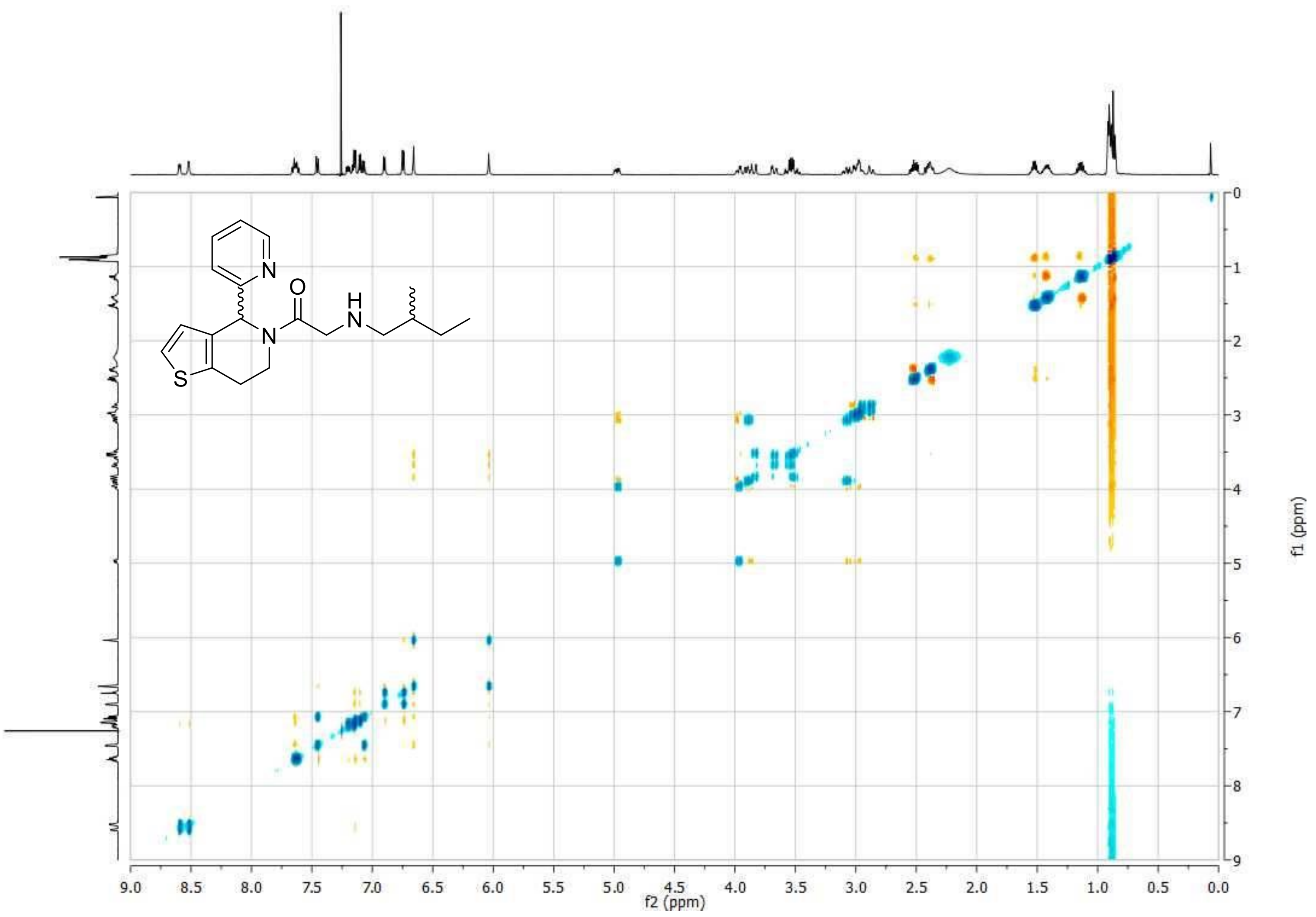

Figure S35. 2D-NOESY (500 MHz, $CDCl_3$, 298K) of **19**.

1-(4-(6-methylpyridin-2-yl)-6,7-dihydrothieno[3,2-c]pyridin-5(4H)-yl)ethan-1-one (21)

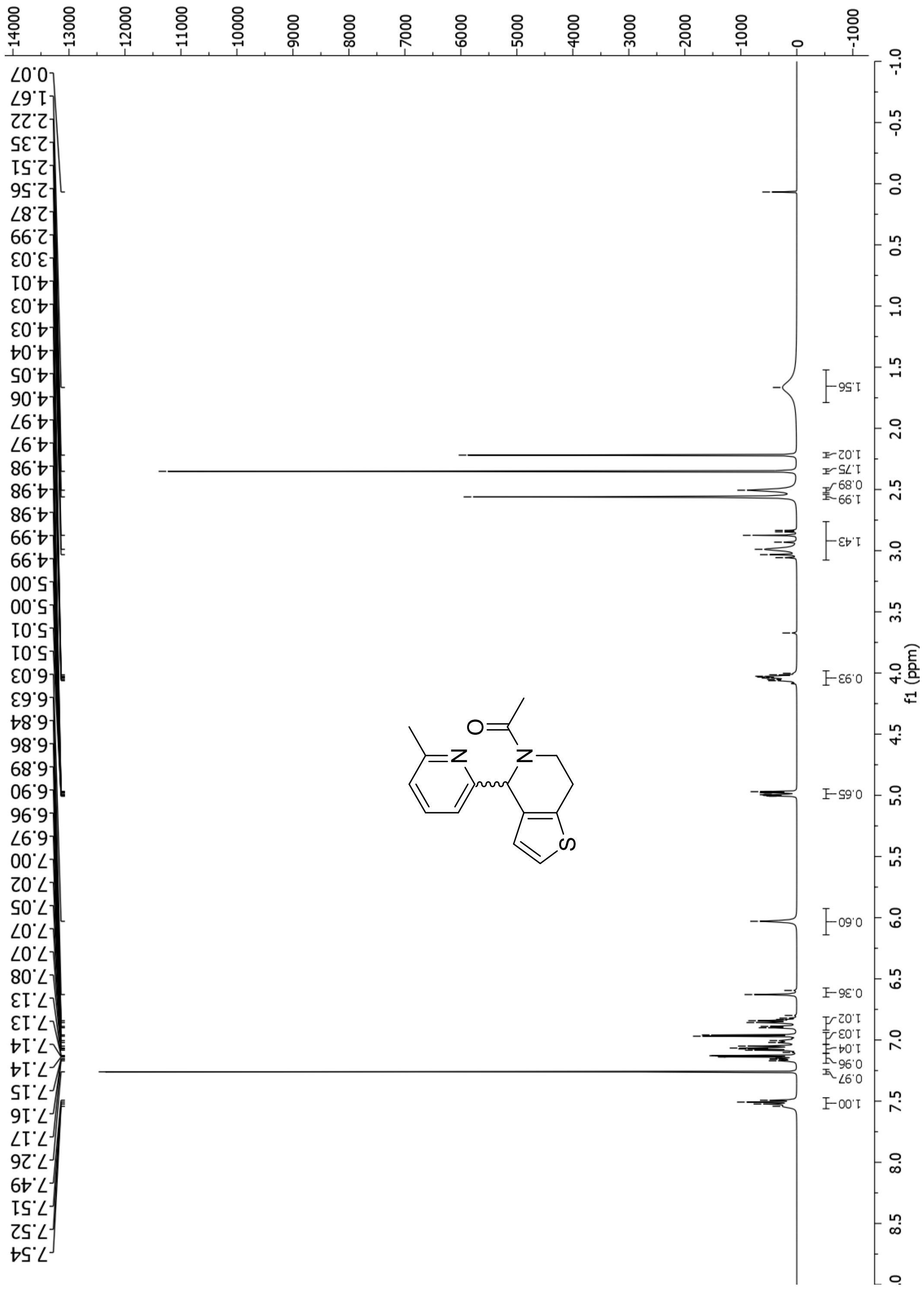

Figure S36. ^{1}H NMR (500 MHz, $CDCl_3$, 298K) of **21**.

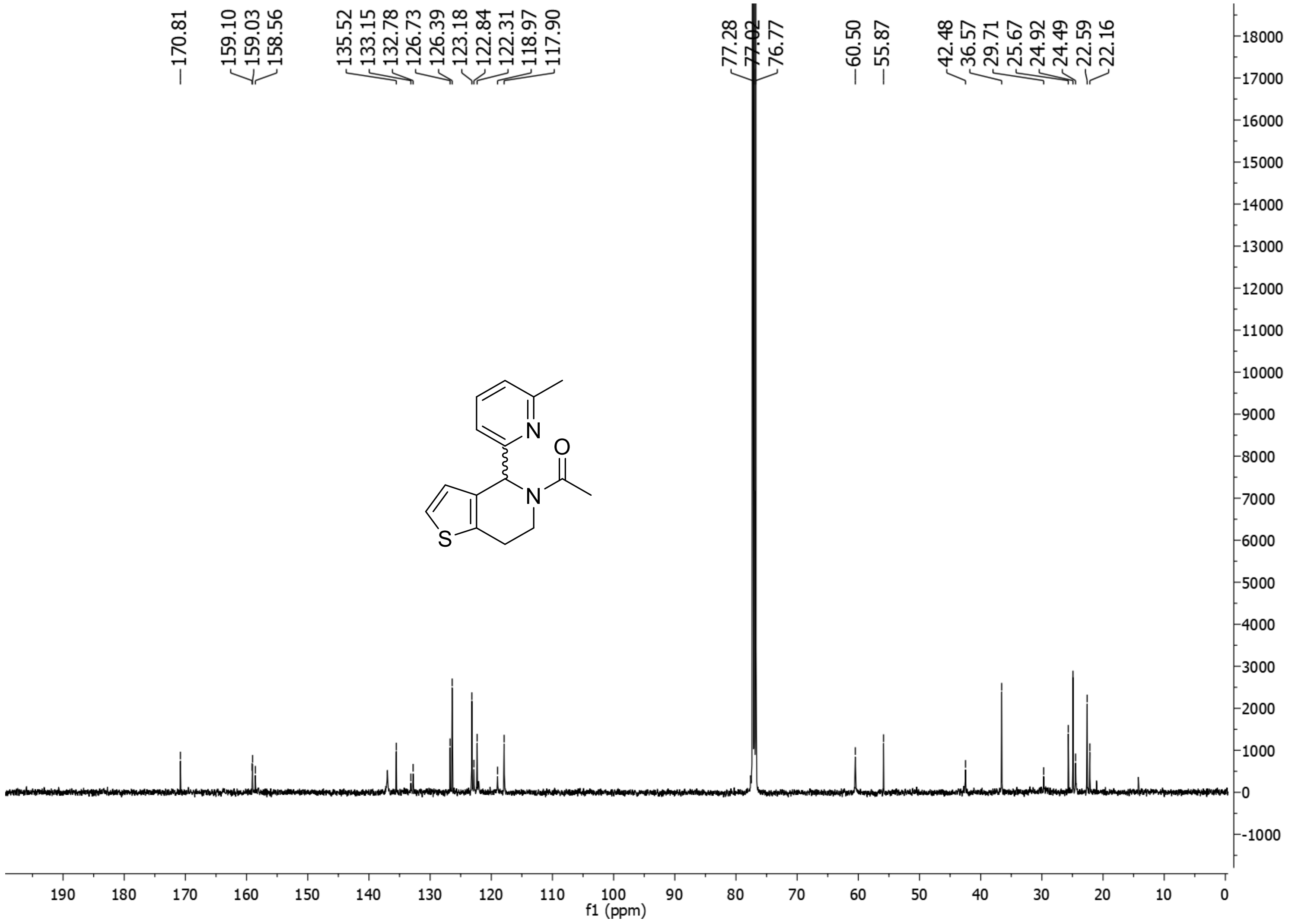

Figure S37. ^{13}C NMR (500 MHz, $CDCl_3$, 298K) of **21**.

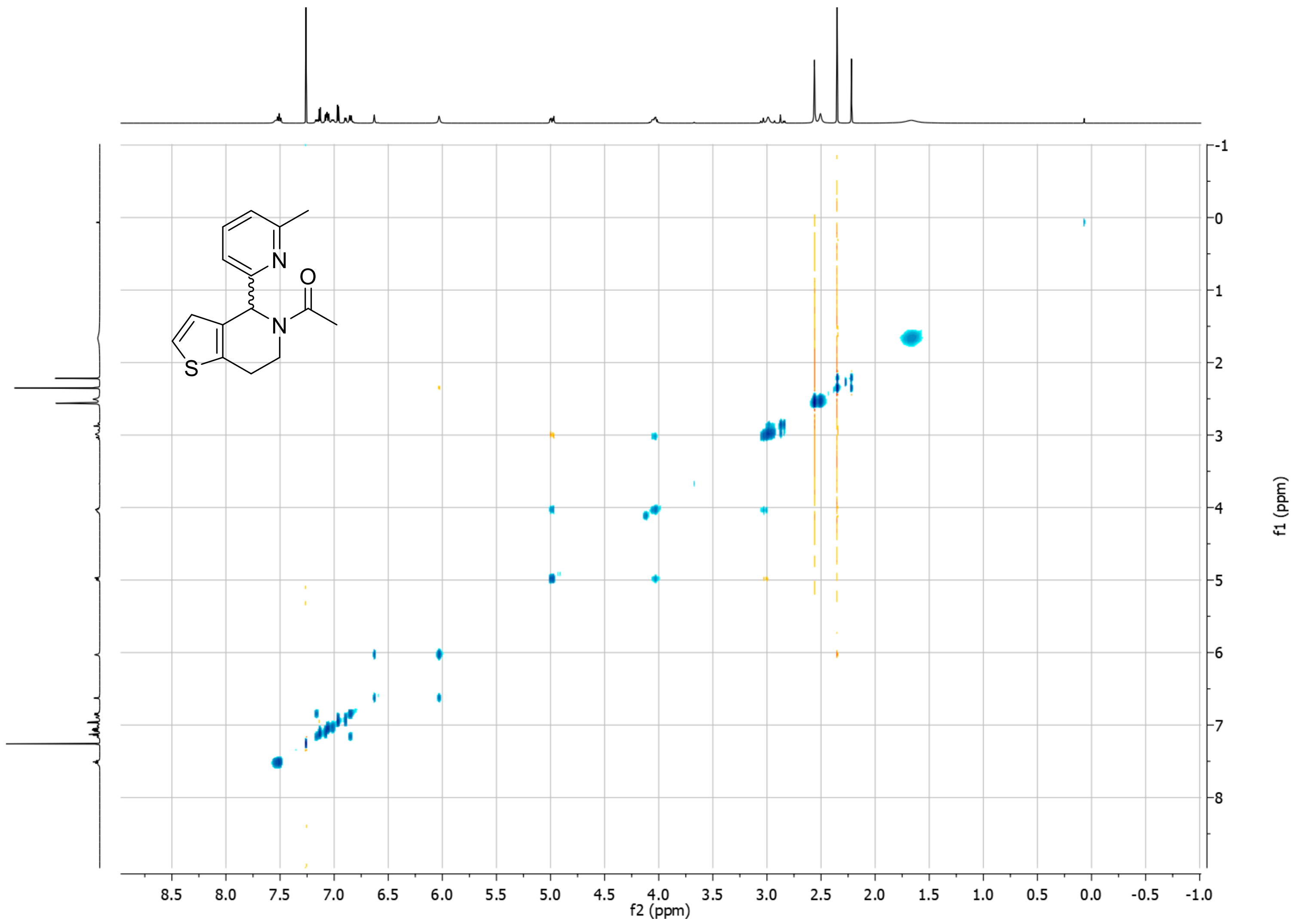

Figure S38. 2D-NOESY (500 MHz, $CDCl_3$, 298K) of **21**.

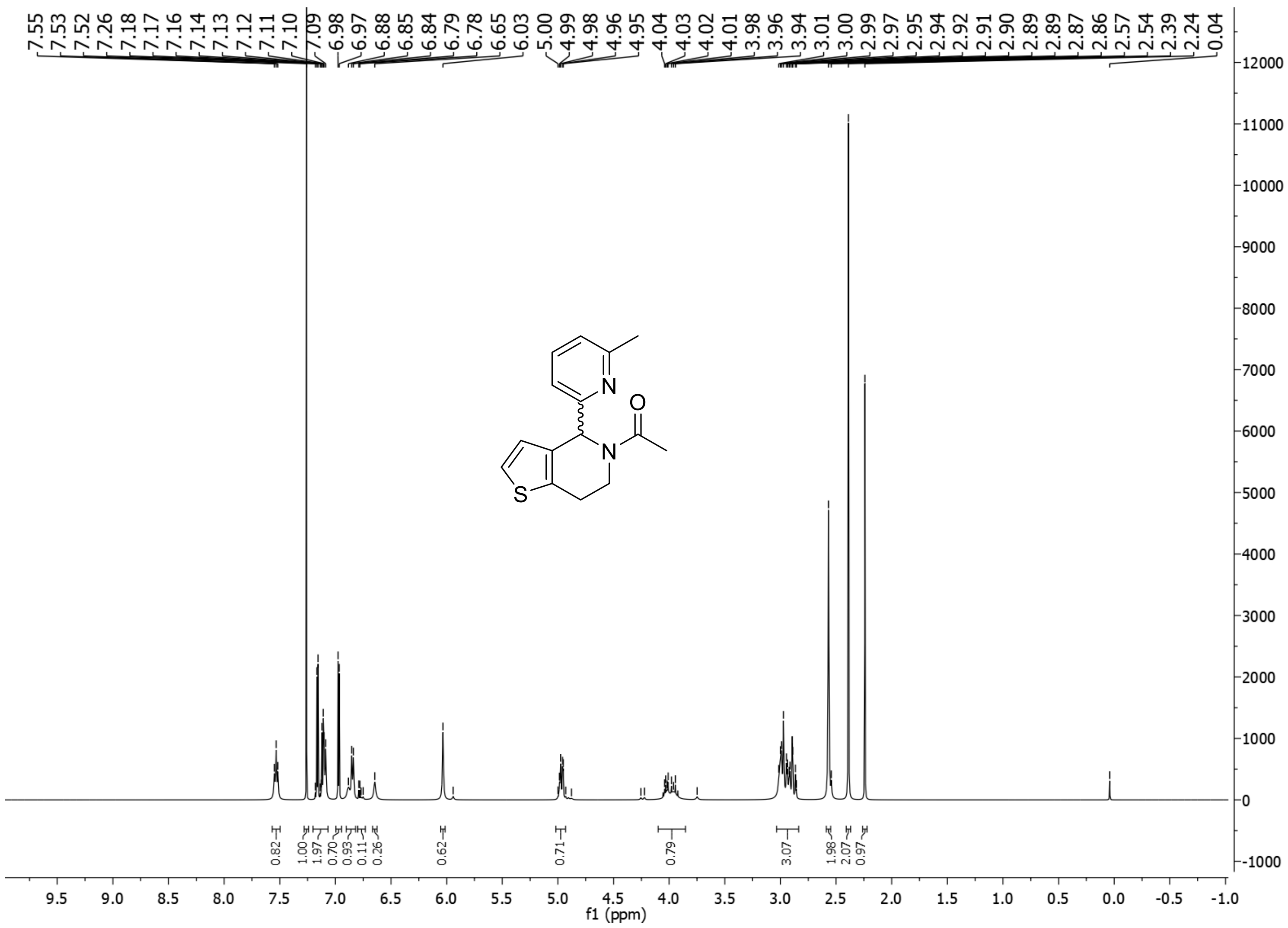

Figure S39. ^{1}H NMR (500 MHz, $CDCl_3$, 218K) of **21**.

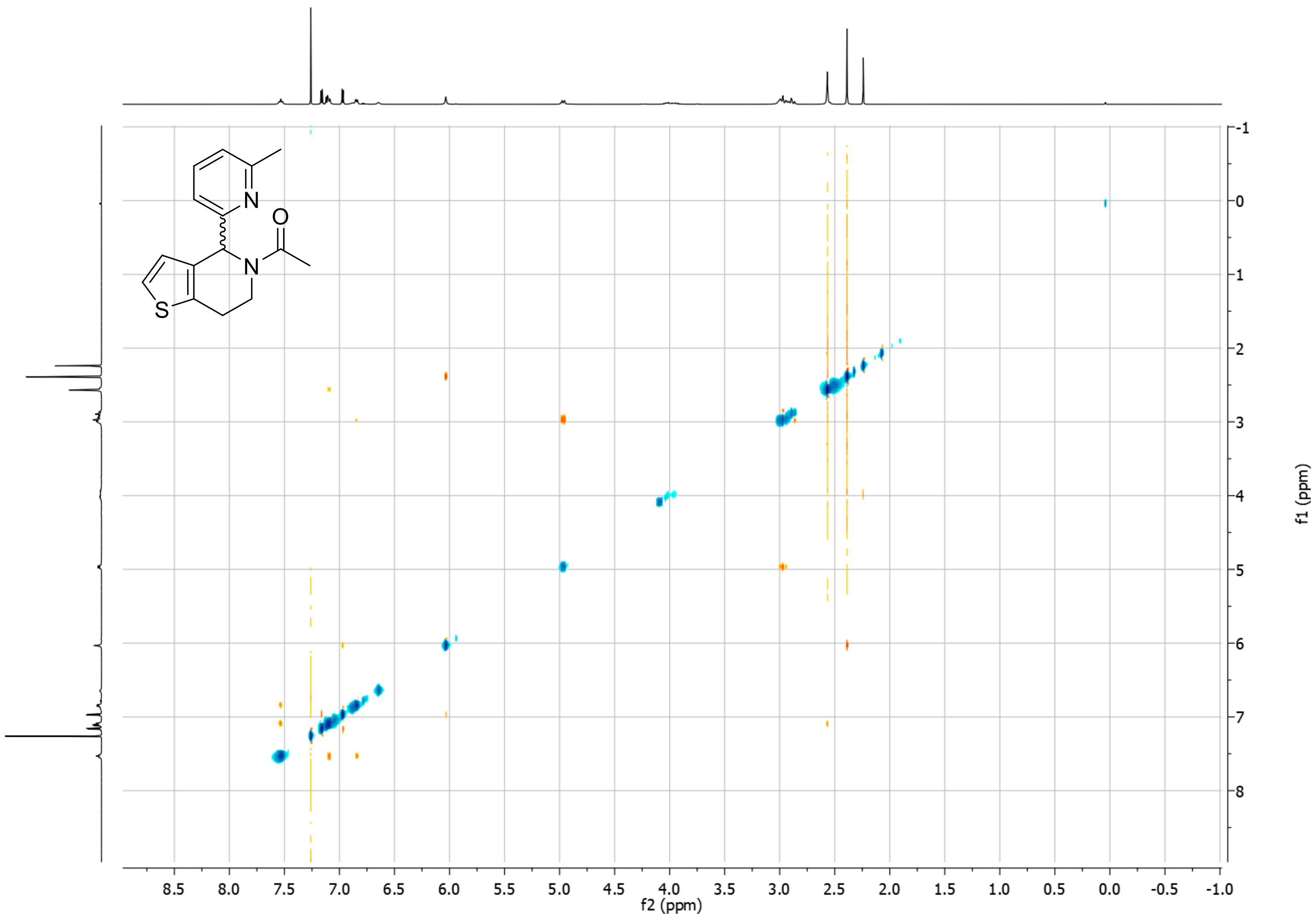

FigFigure S40. 2D-NOESY (500 MHz, $CDCl_3$, 218K) of **21**.

2-(S)-(2-methylbutylamino)-1-[4-(6-methylpyridin-2-yl)-6,7-dihydro-4H-thieno[3,2-c]pyridin-5-yl]ethanone (23)

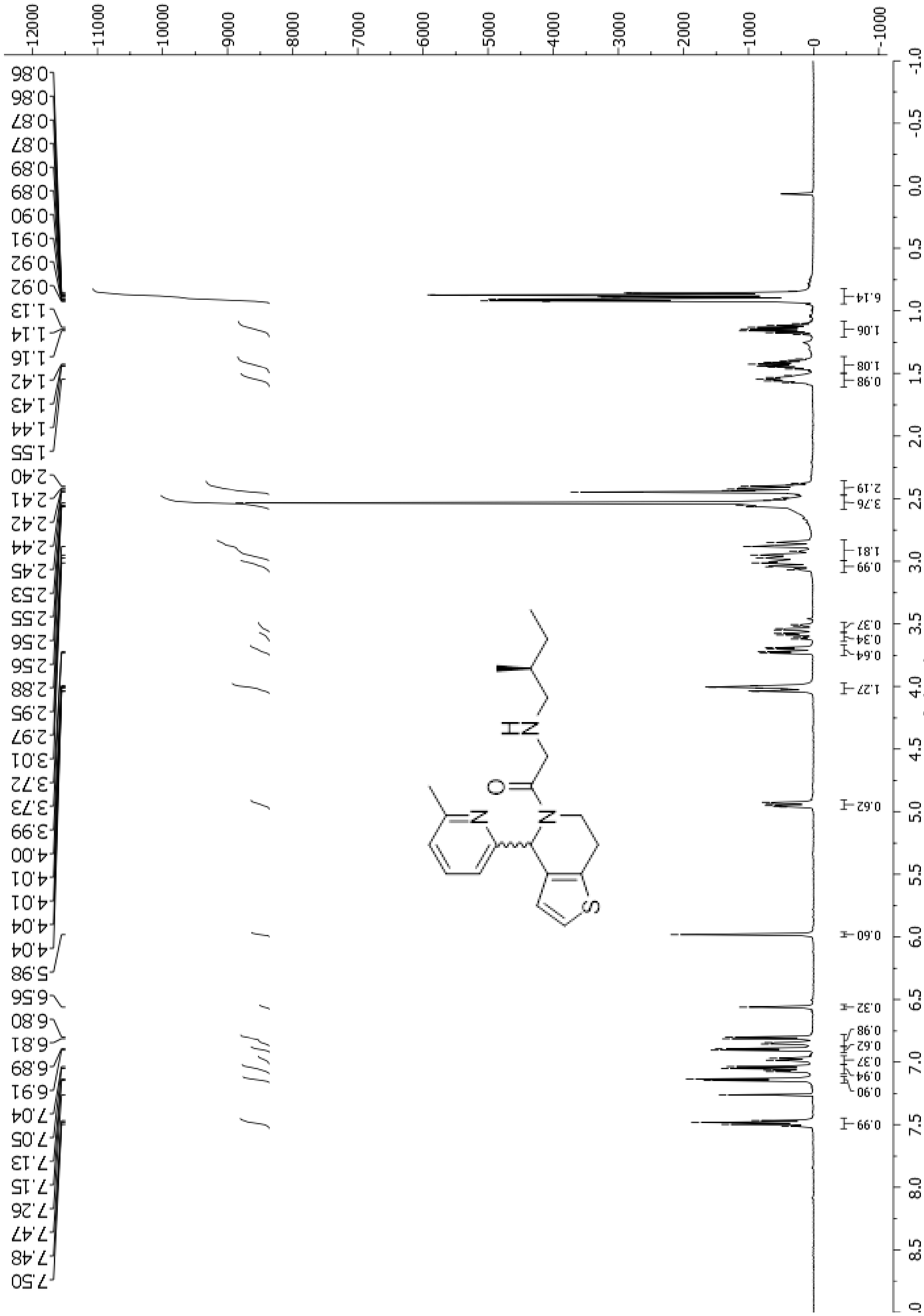

Figure S41. ^{1}H NMR (500 MHz, $CDCl_3$, 298K) of **23**.

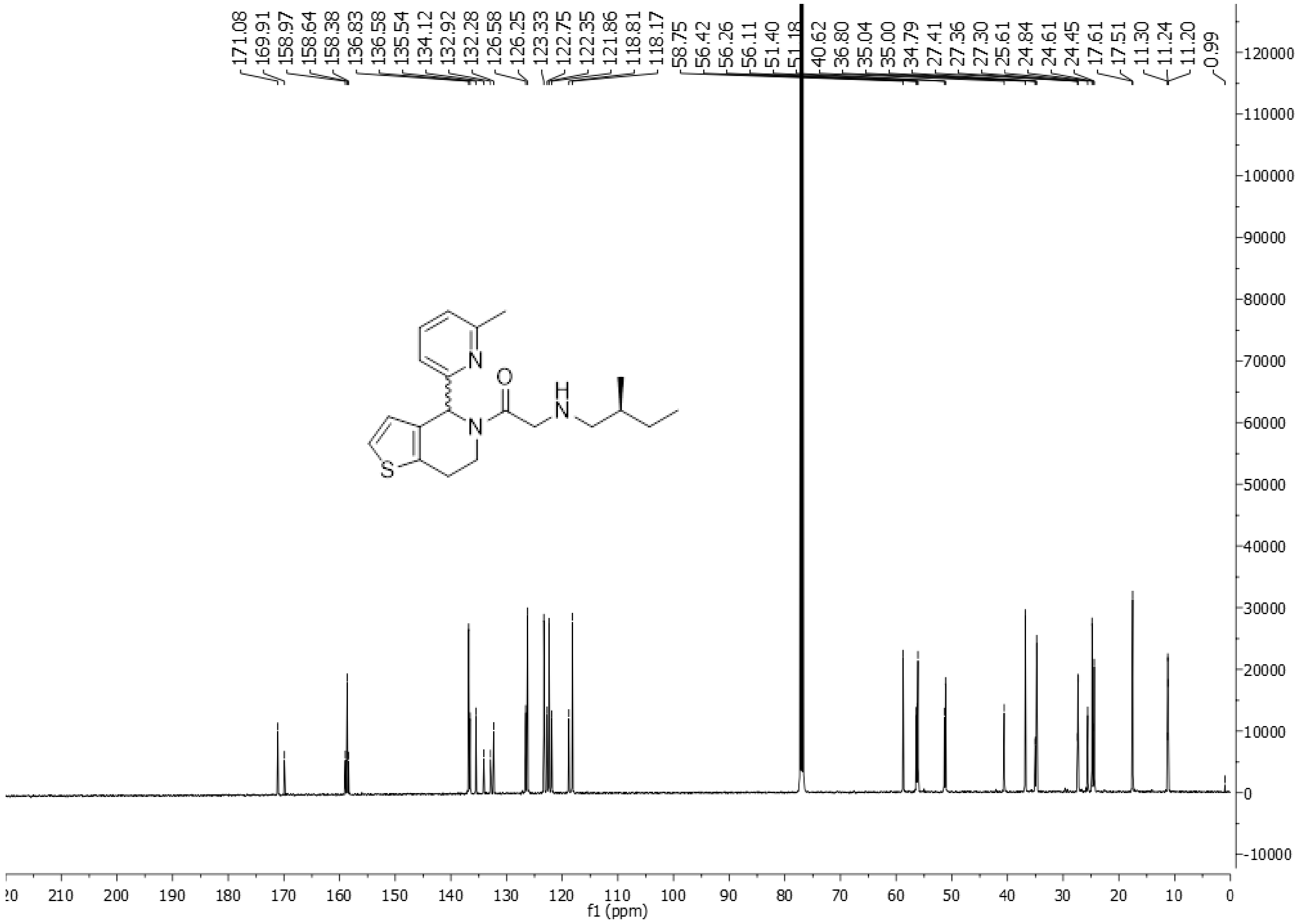

Figure S42. ^{13}C NMR (500 MHz, $CDCl_3$, 298K) of **23**.

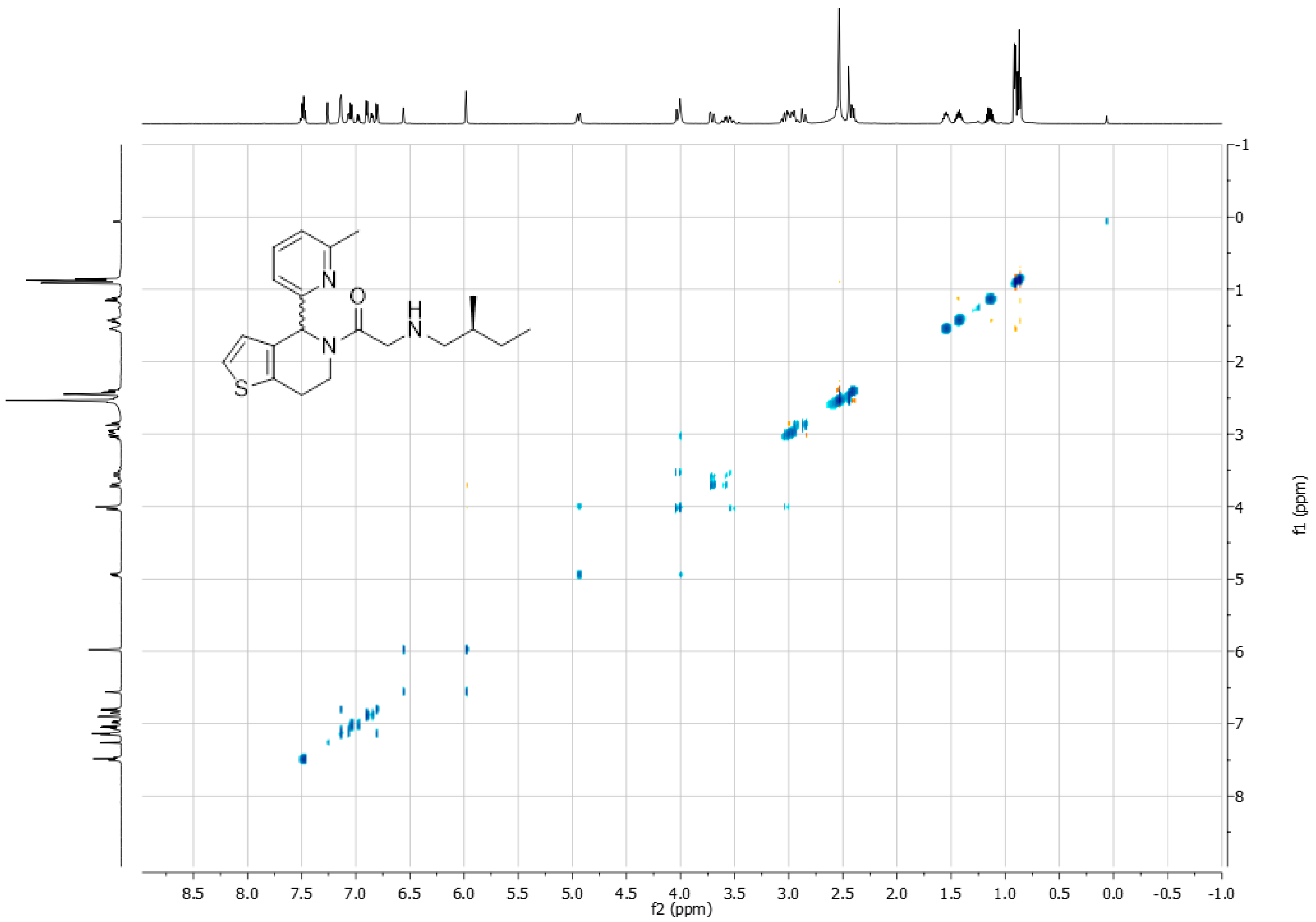

Figure S43. 2D-NOESY (500 MHz, $CDCl_3$, 298K) o **23**.

2-(2-methylpropylamino)-1-[4-(6-methylpyridin-2-yl)-6,7-dihydro-4*H*-thieno[3,2-c]pyridin-5-yl]ethanone (24)

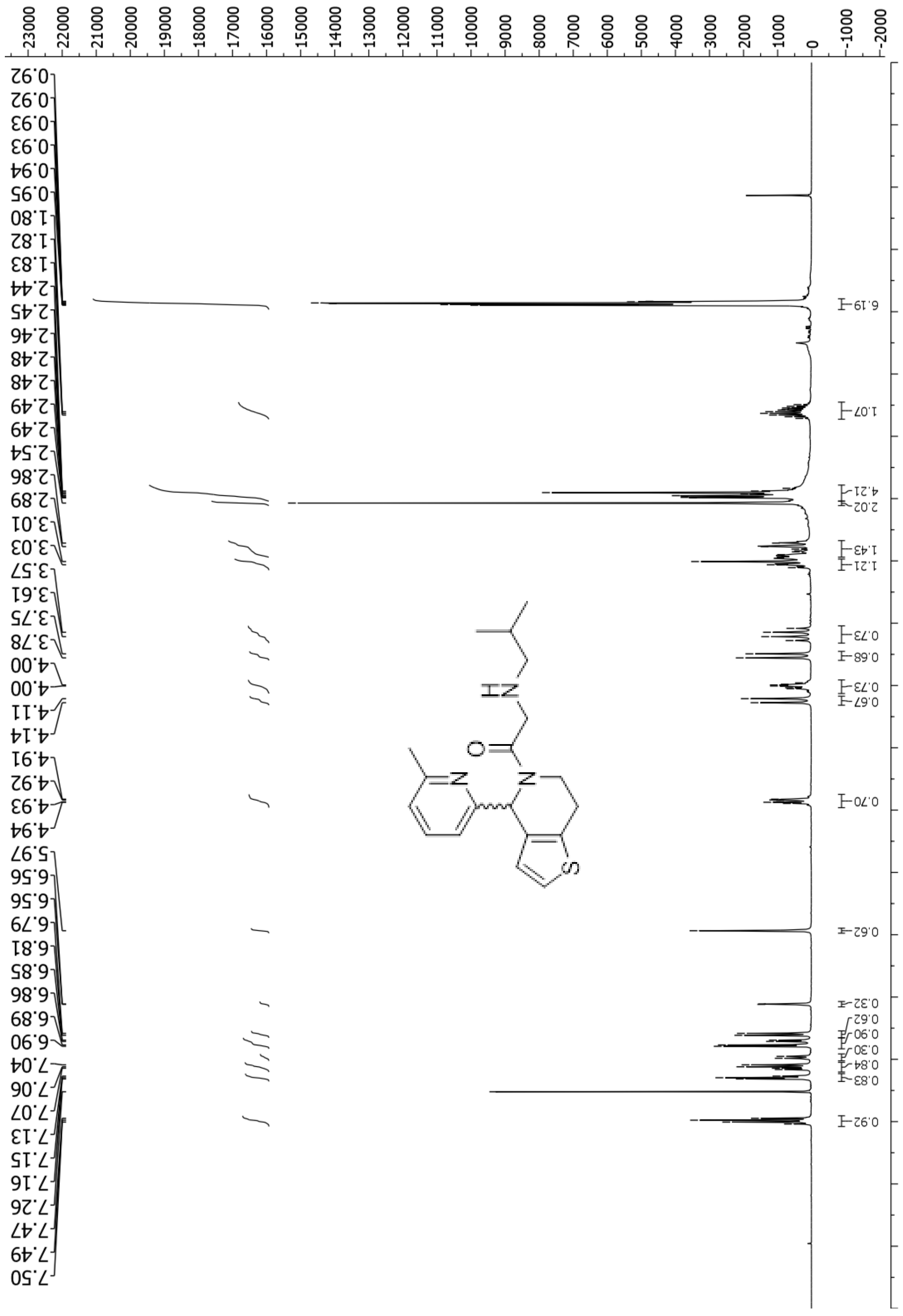

Figure S44. ^{1}H NMR (500 MHz, $CDCl_3$, 298K) of **24**.

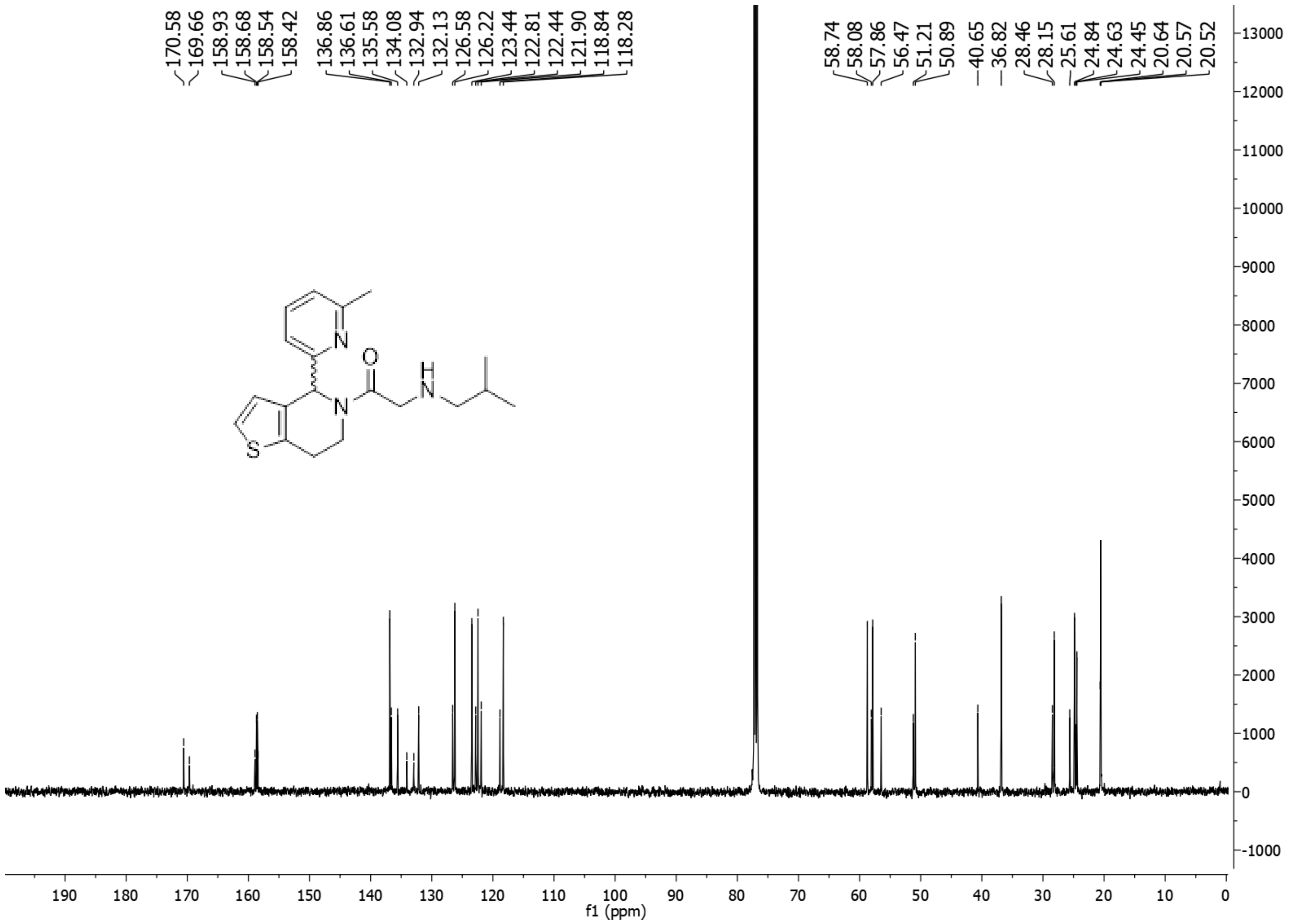

Figure S45. ^{13}C NMR (500 MHz, $CDCl_3$, 298K) of **24**.

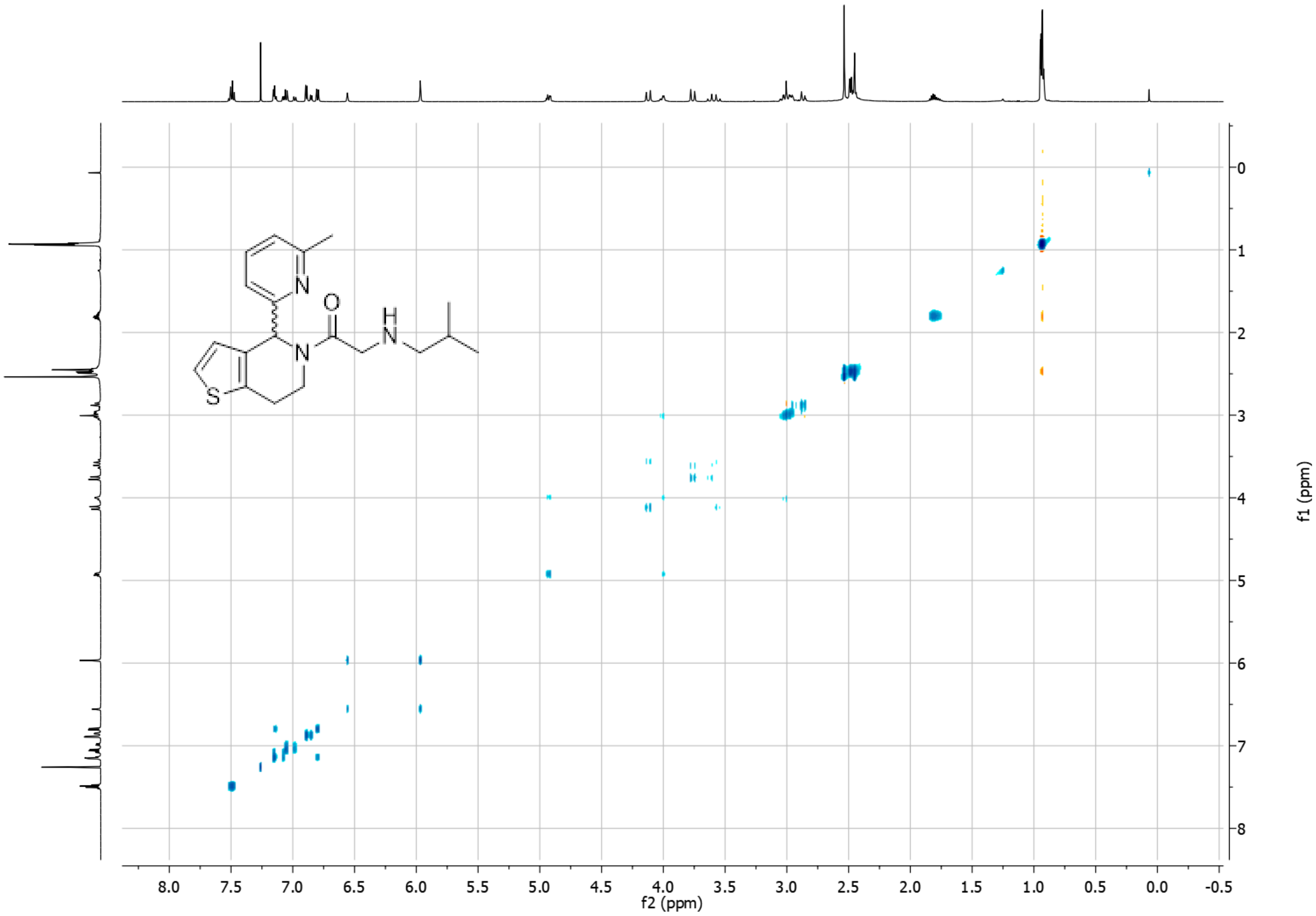

Figure S46. 2D-NOESY (500 MHz, $CDCl_3$, 298K) o **24**.

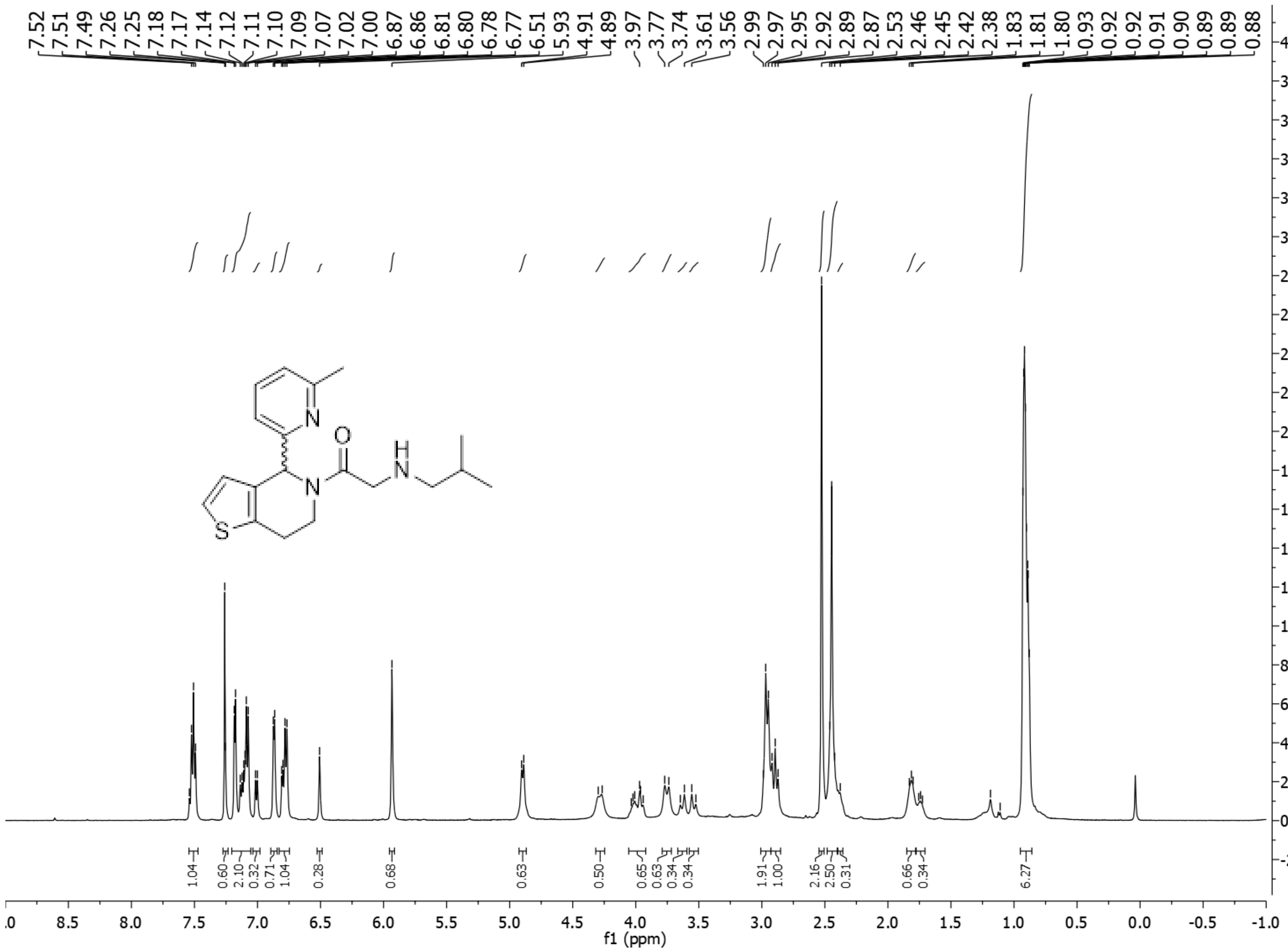

Figure S47. ^{1}H NMR (500 MHz, $CDCl_3$, 218K) of **24**.

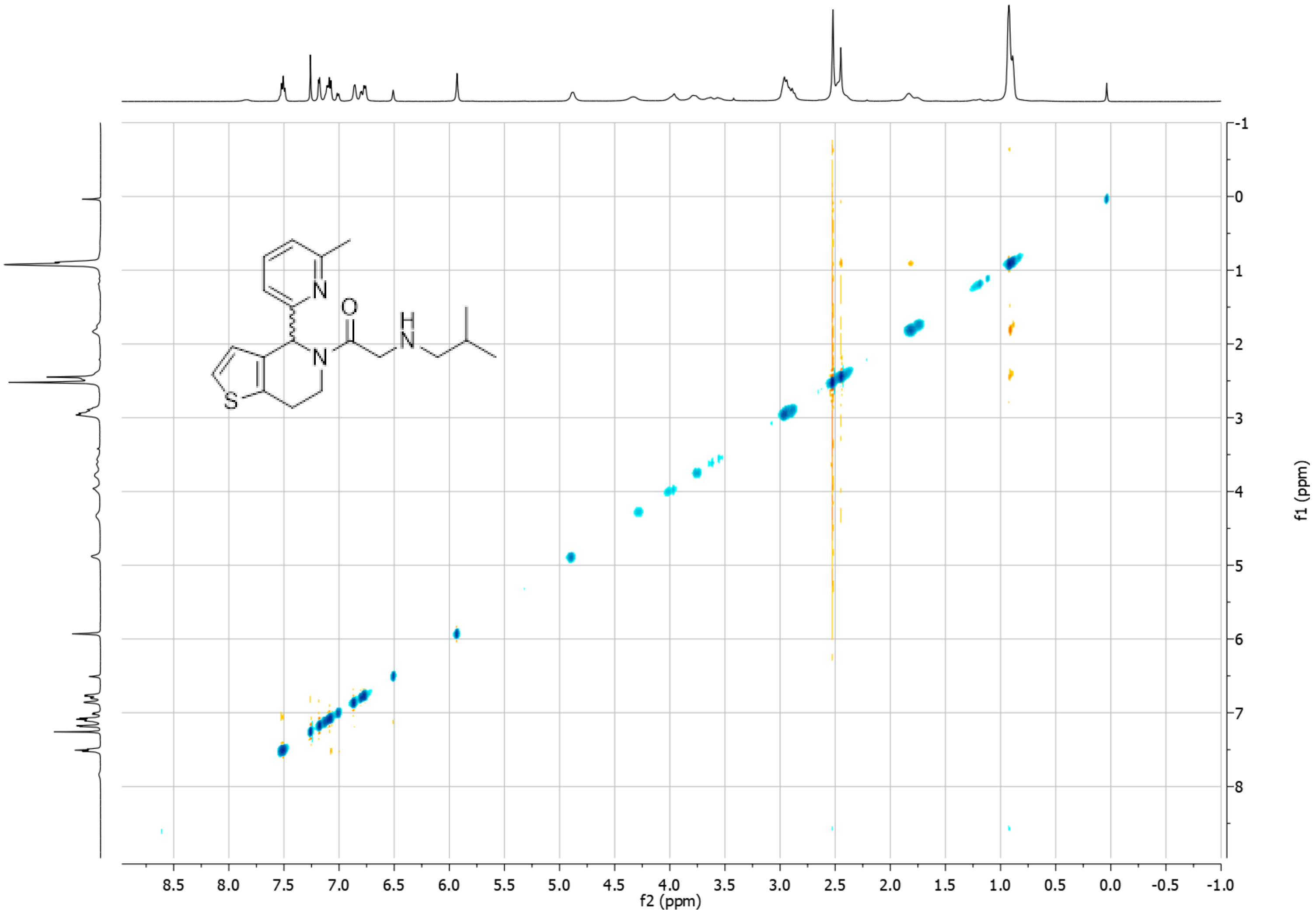

Figure S48. 2D-NOESY (500 MHz, $CDCl_3$, 218K) o **24**.

2-(isopentylamino)-1-(4-(6-methylpyridin-2-yl)-6,7-dihydrothieno[3,2-*c*]pyridin-5(4*H*)-yl)ethan-1-one (25)

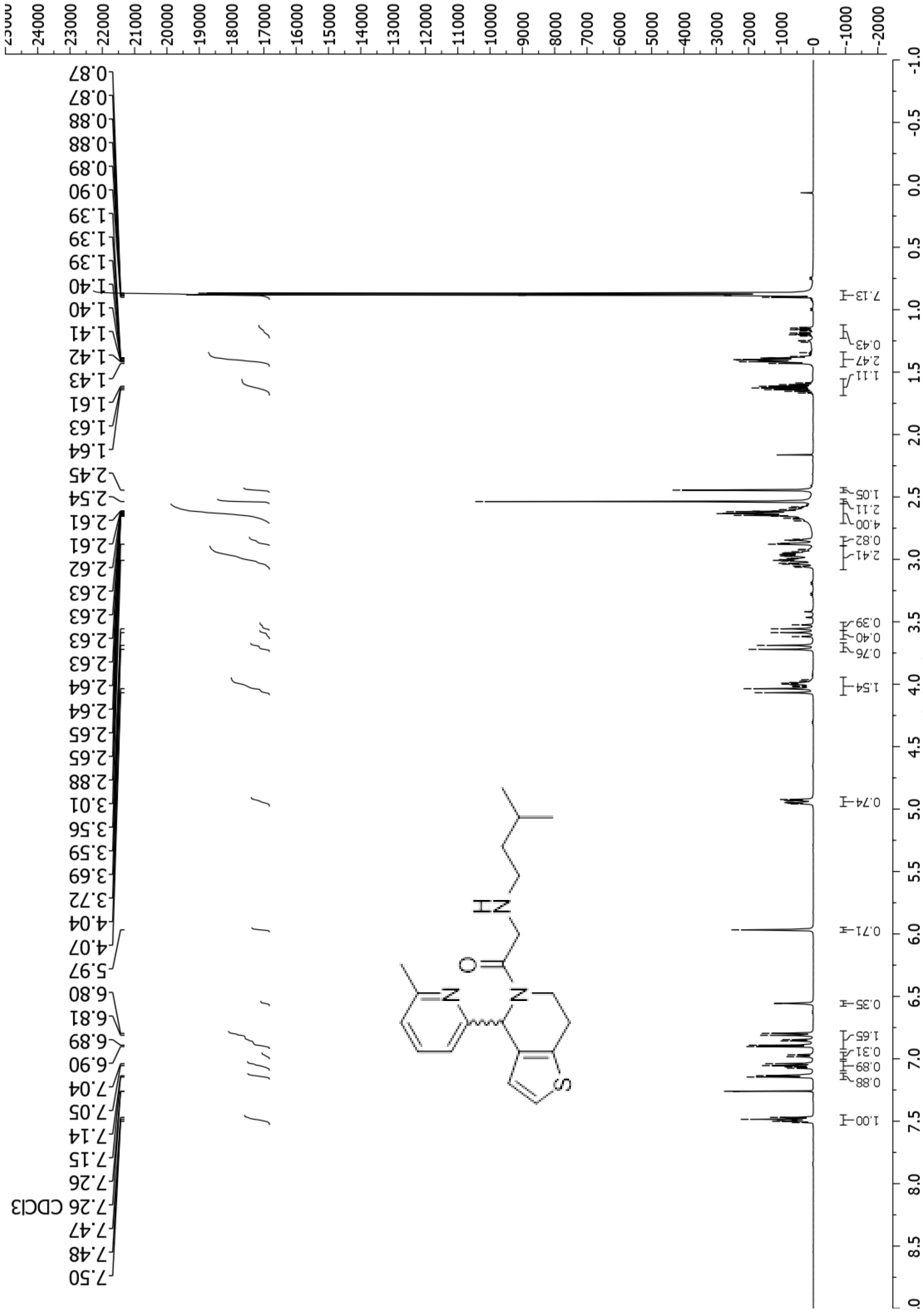

Figure S49. ^{1}H NMR (500 MHz, $CDCl_3$, 298K) of **25**.

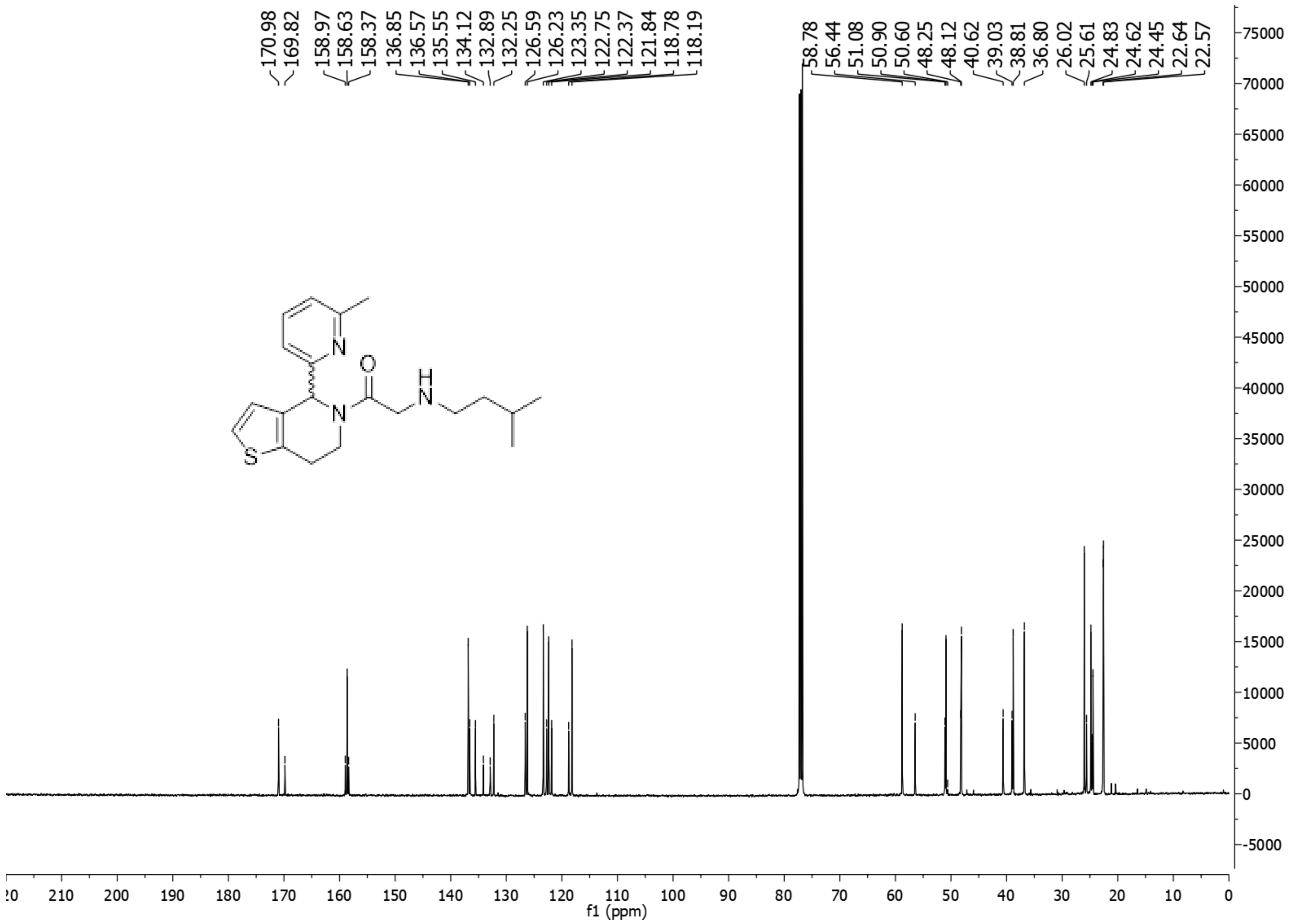

Figure S50. ^{13}C NMR (500 MHz, $CDCl_3$, 298K) of **25**.

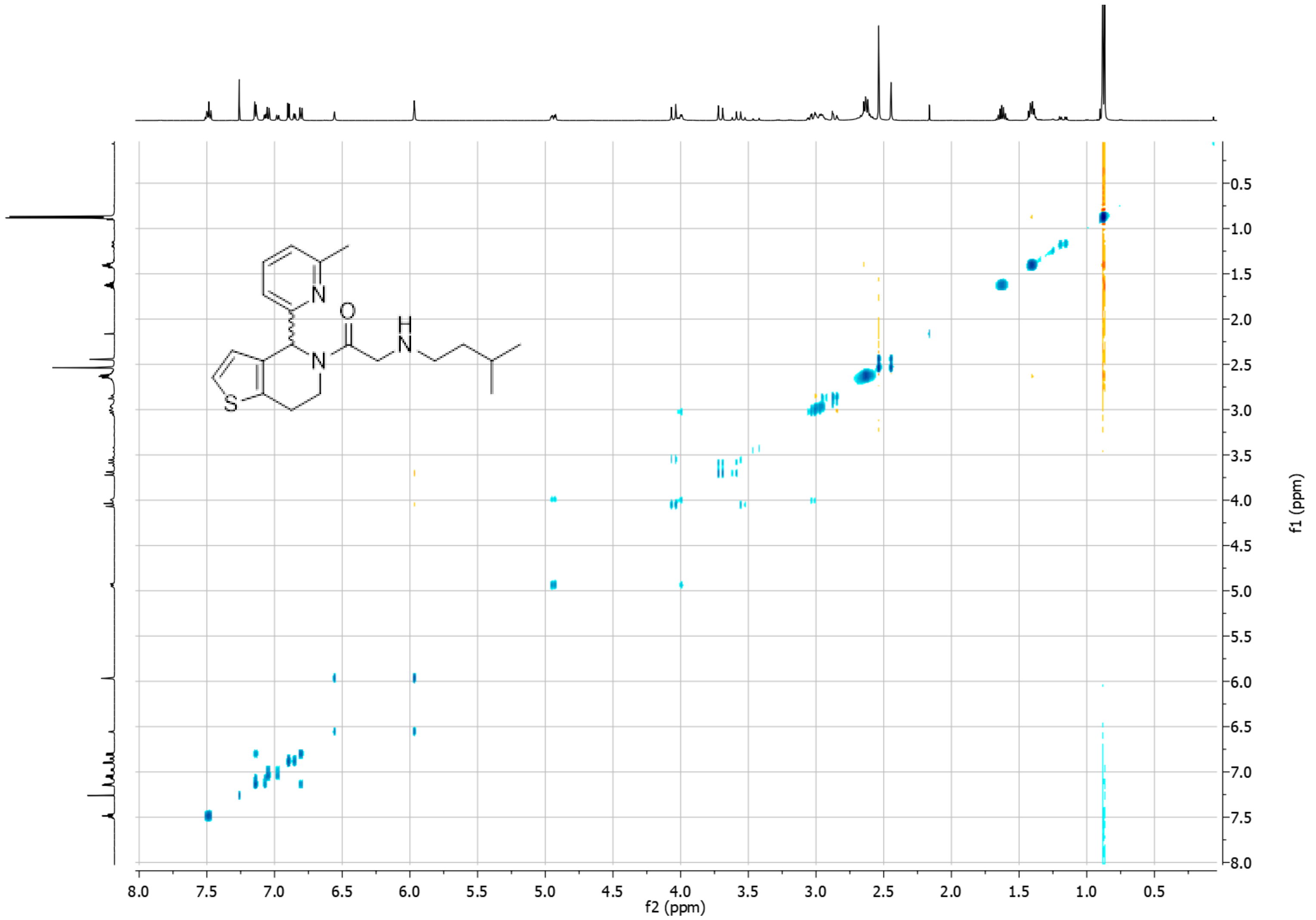

Figure S51. 2D-NOESY (500 MHz, $CDCl_3$, 298K) o **25**.

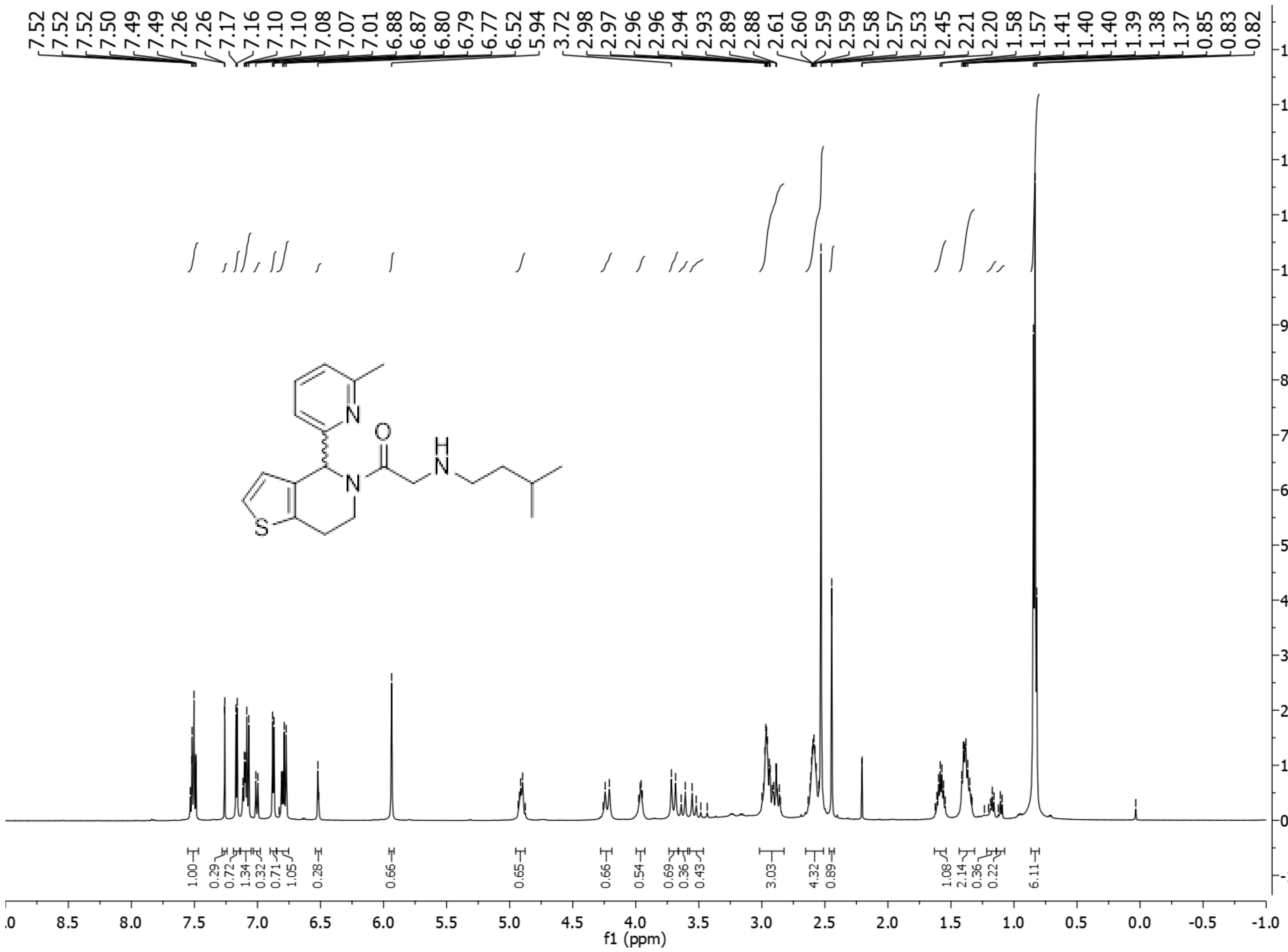

Figure S52. ^{13}C NMR (500 MHz, $CDCl_3$, 218K) of **25**.

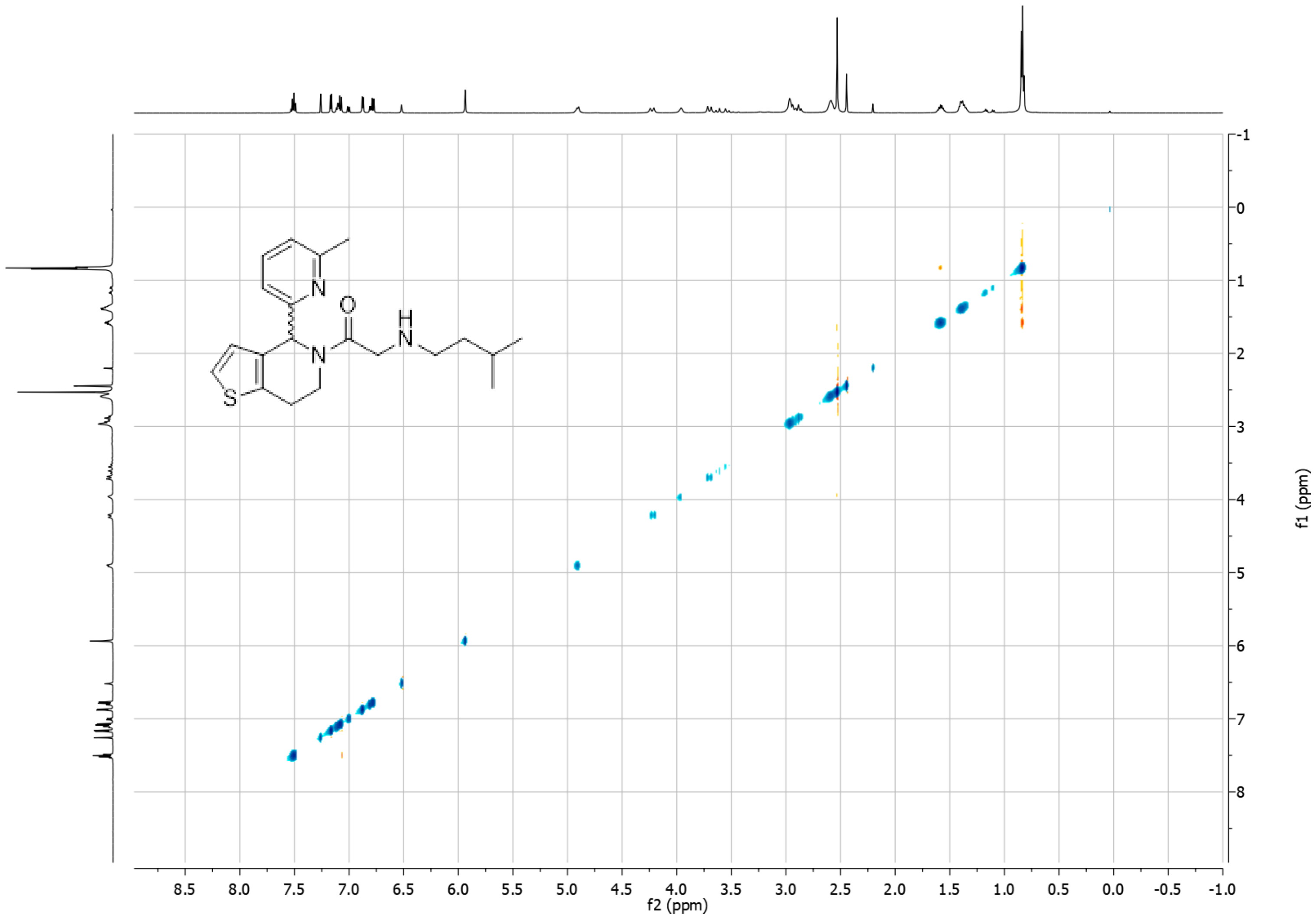

Figure S53. 2D-NOESY (500 MHz, $CDCl_3$, 218K) o **25**.

N-isopentyl-4-(6-methylpyridin-2-yl)-6,7-dihydrothieno[3,2-c]pyridine-5(4H)-carboxamide (26)

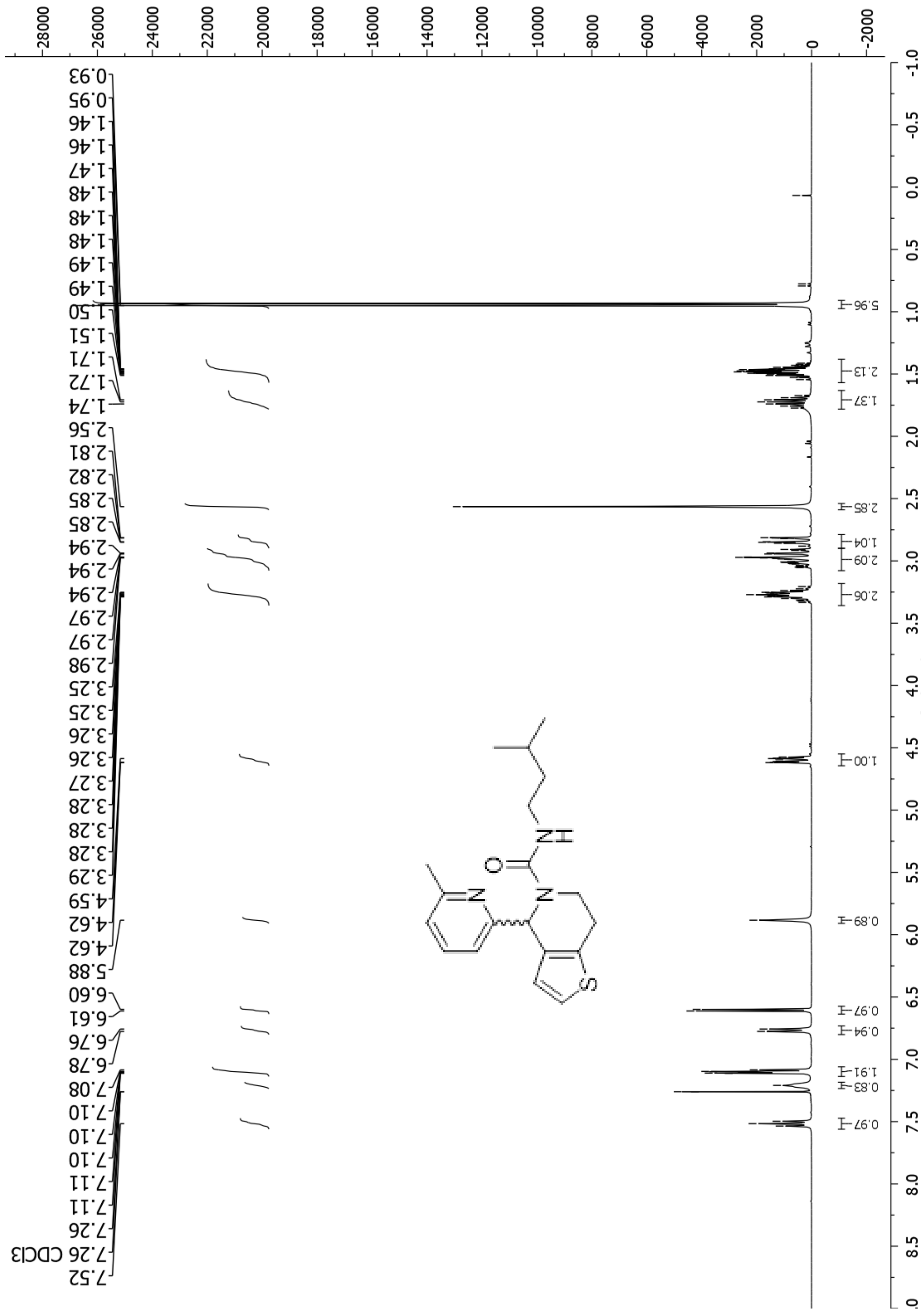

Figure S54. ^{1}H NMR (400 MHz, $CDCl_3$, 298K) of **26**.

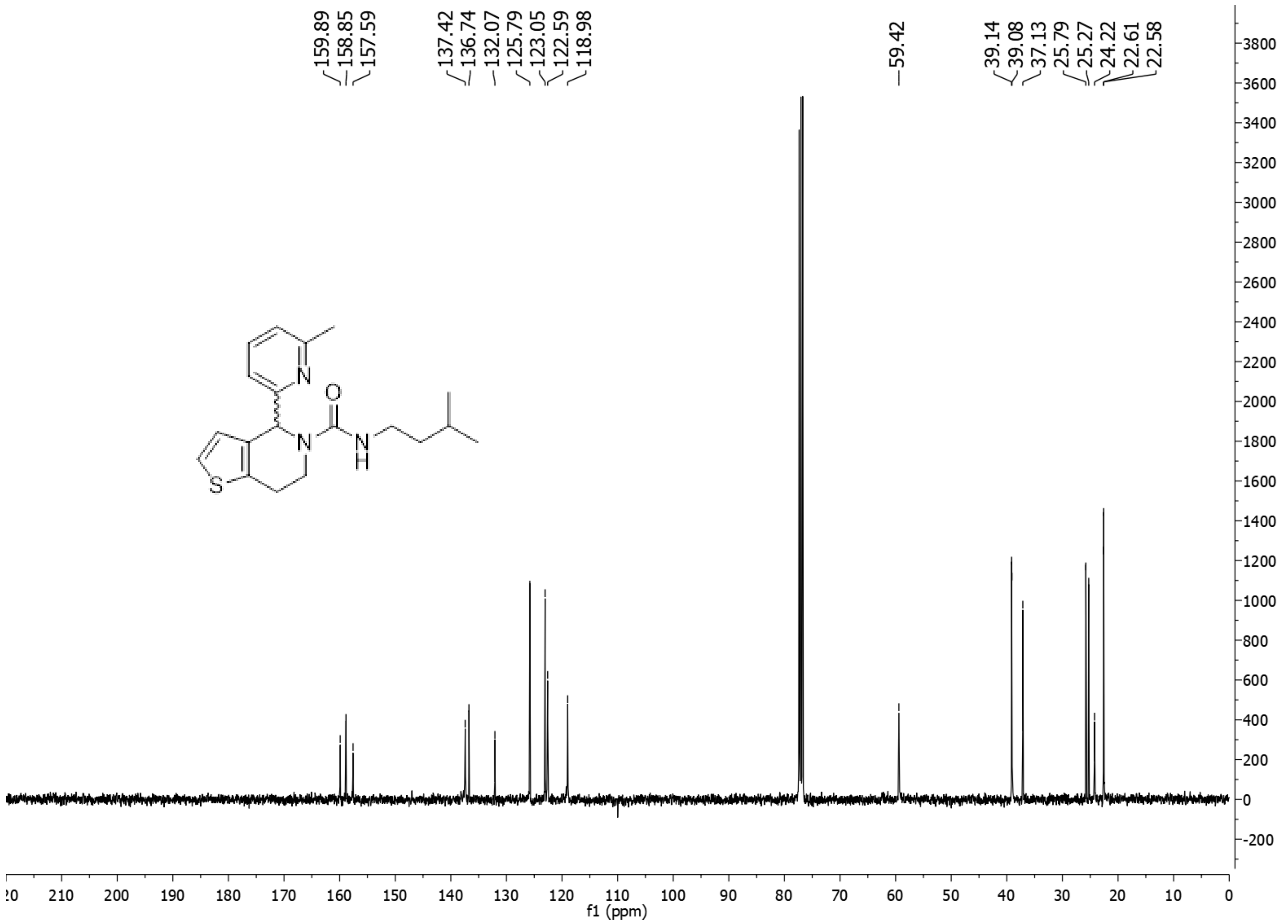

Figure S55. ^{13}C NMR (400 MHz, $CDCl_3$, 298K) of **26**.

(*S*)-1-(4-(6-methylpyridin-2-yl)-6,7-dihydrothieno[3,2-*c*]pyridin-5(4*H*)-yl)ethan-1-one (27)

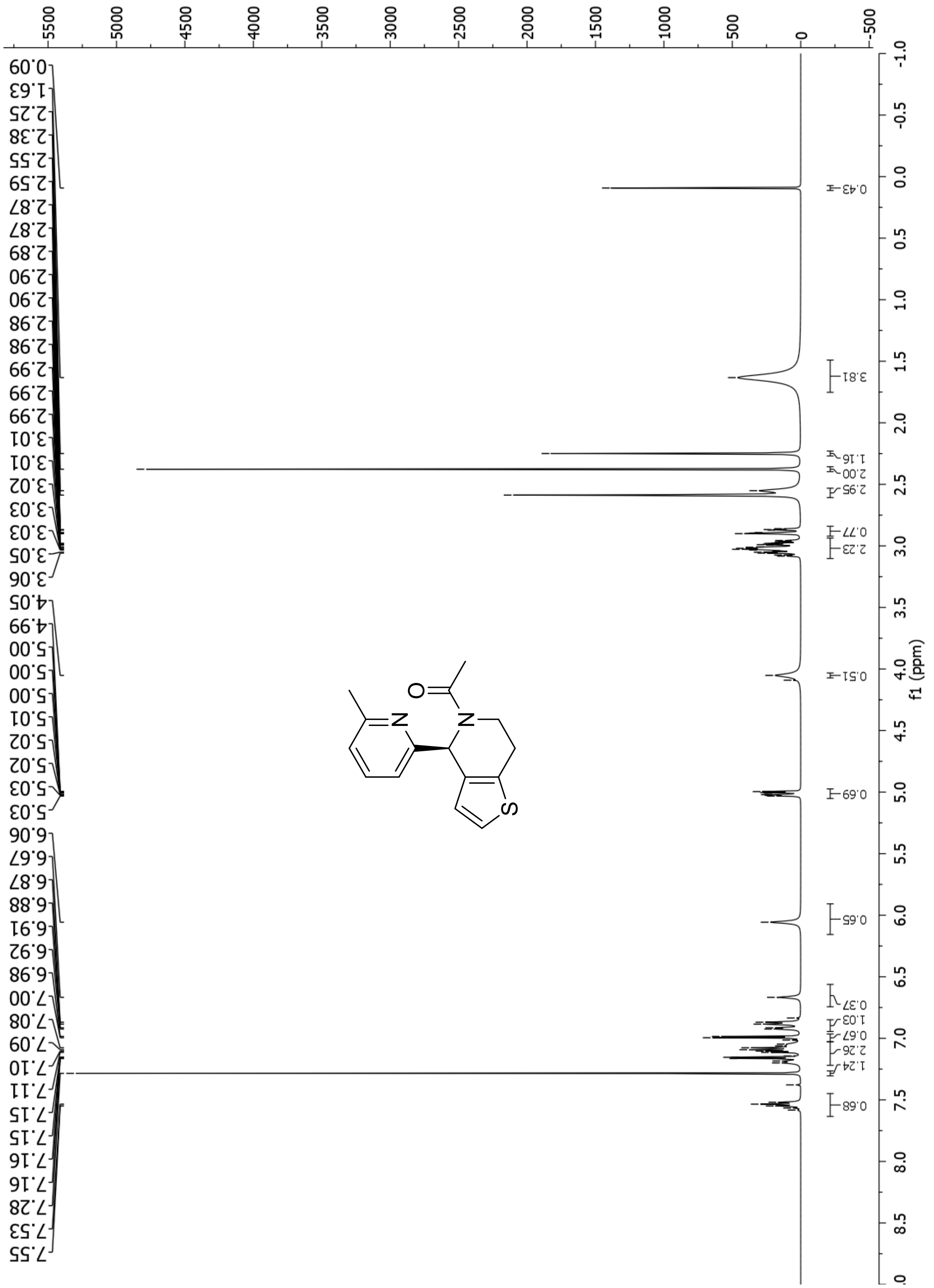

Figure S56. ^{1}H NMR (500 MHz, $CDCl_3$, 298K) of **27**.

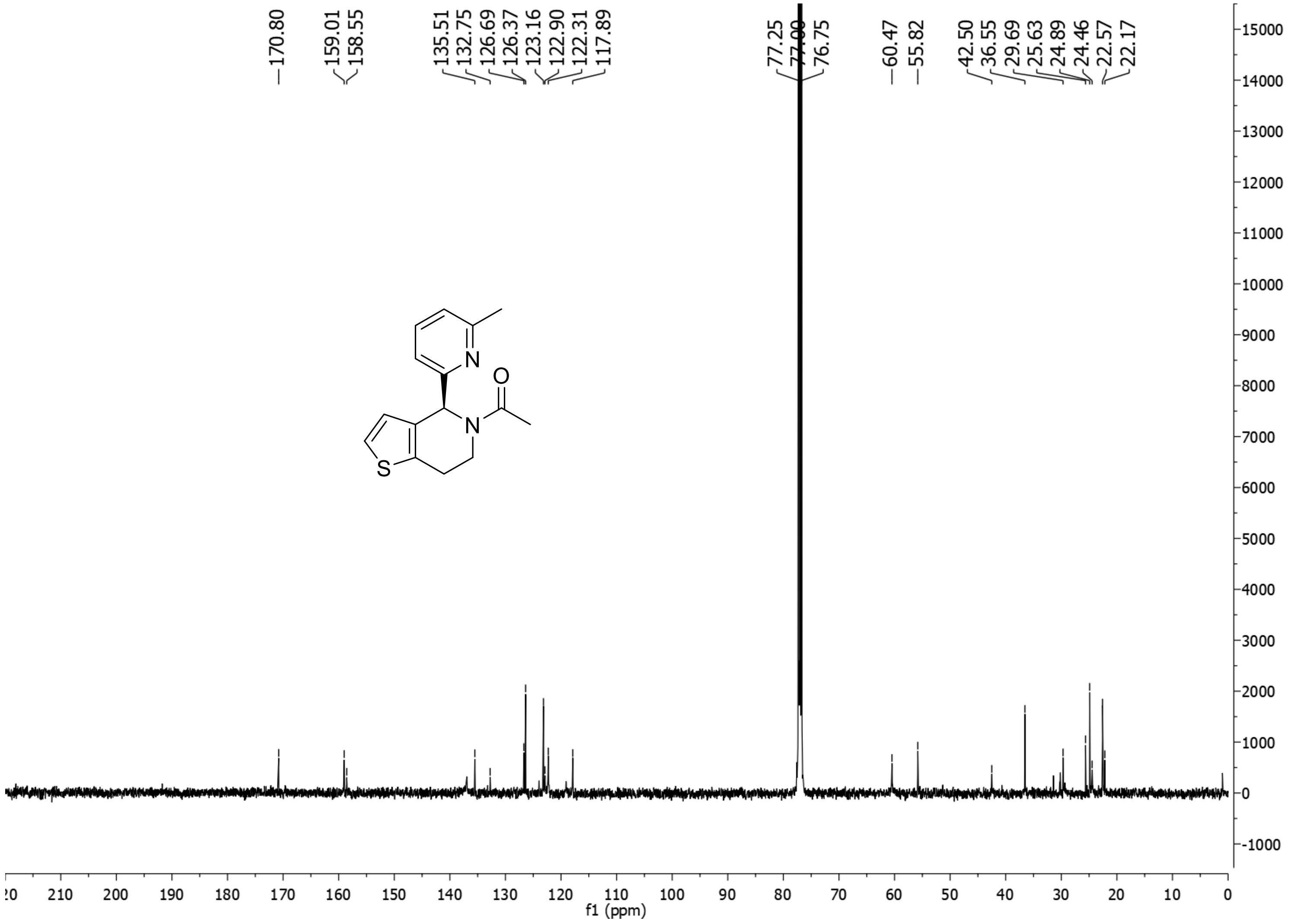

Figure S57. ^{13}C NMR (500 MHz, $CDCl_3$, 298K) of **27**.

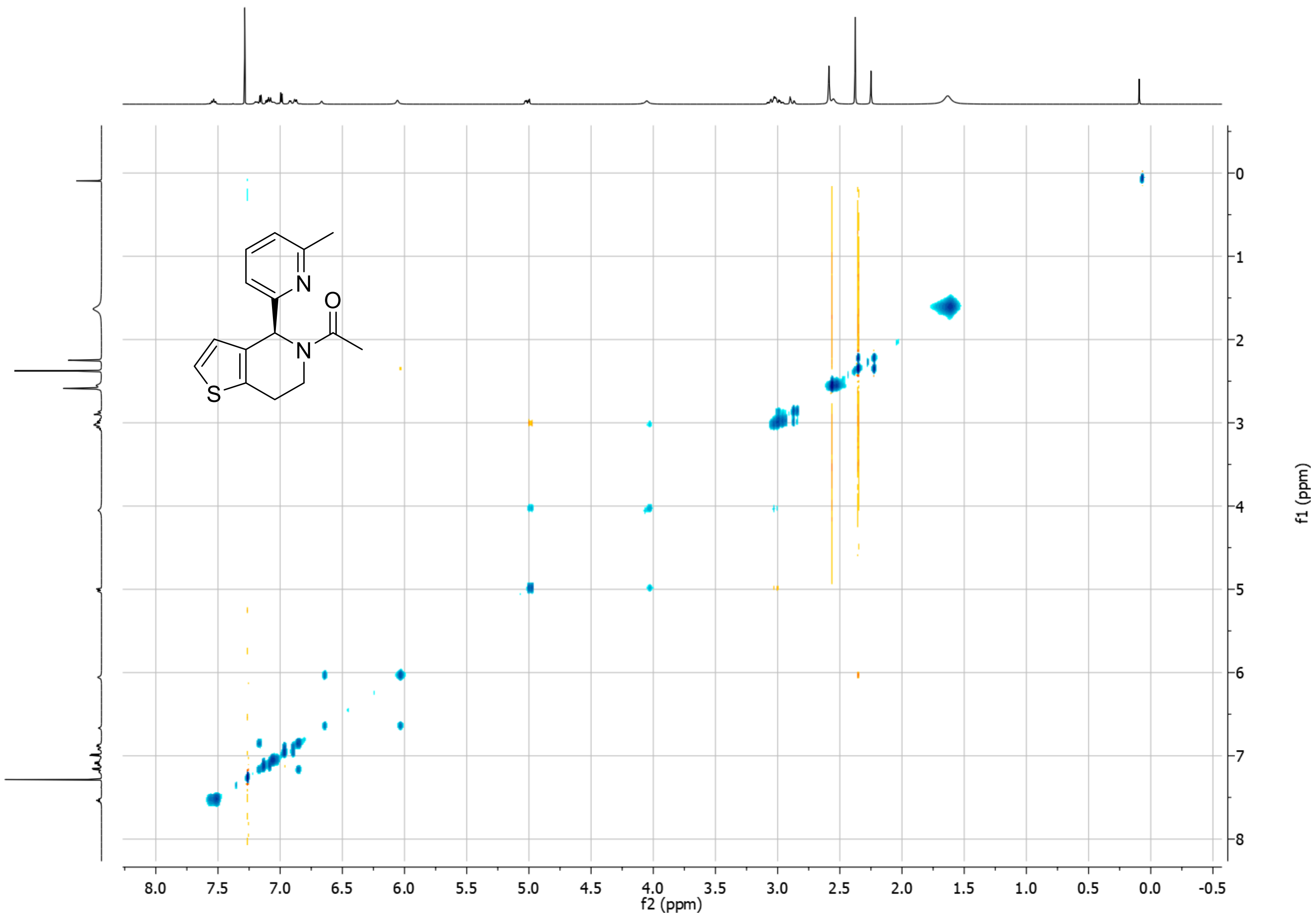

Figure S58. 2D-NOESY (500 MHz, $CDCl_3$, 298K) o **27**.

(*S*)-2-(2-methylpropylamino)-1-[4-(6-methylpyridin-2-yl)-6,7-dihydro-4*H*-thieno[3,2-*c*]pyridin-5-yl]ethenone (28)

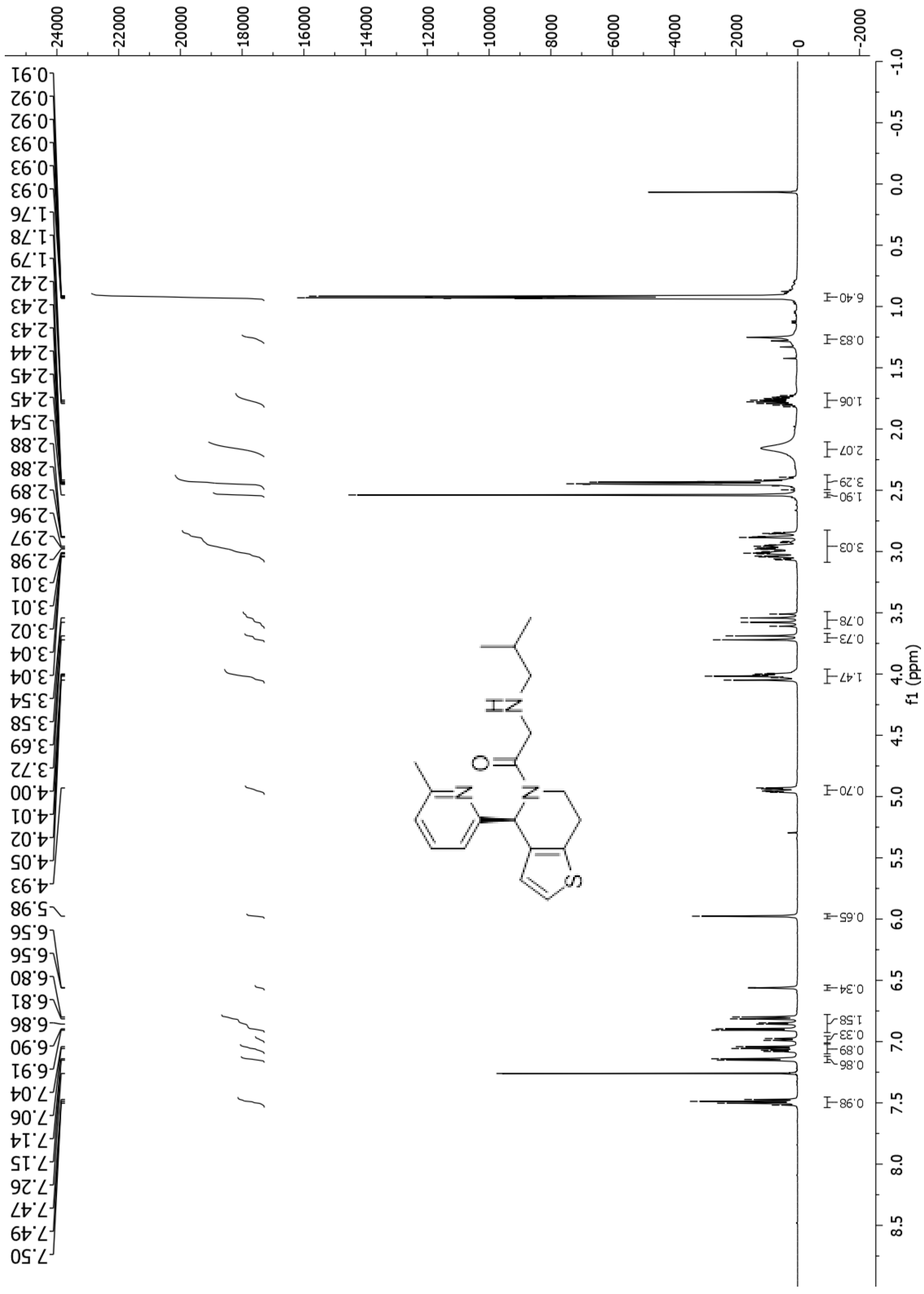

Figure S59. ^{1}H NMR (500 MHz, $CDCl_3$, 298K) of **28**.

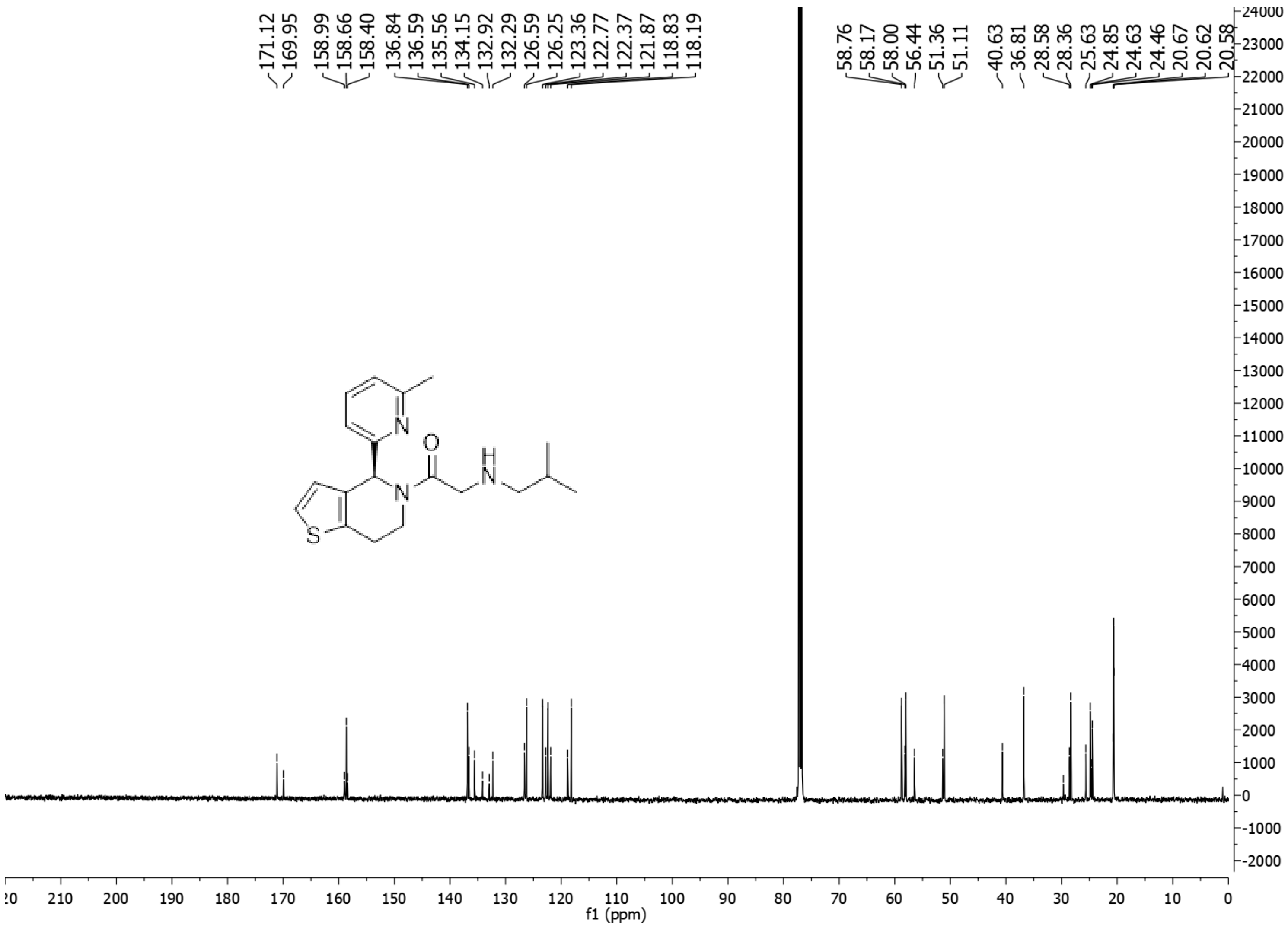

Figure S60. ^{13}C NMR (500 MHz, $CDCl_3$, 298K) of **28**.

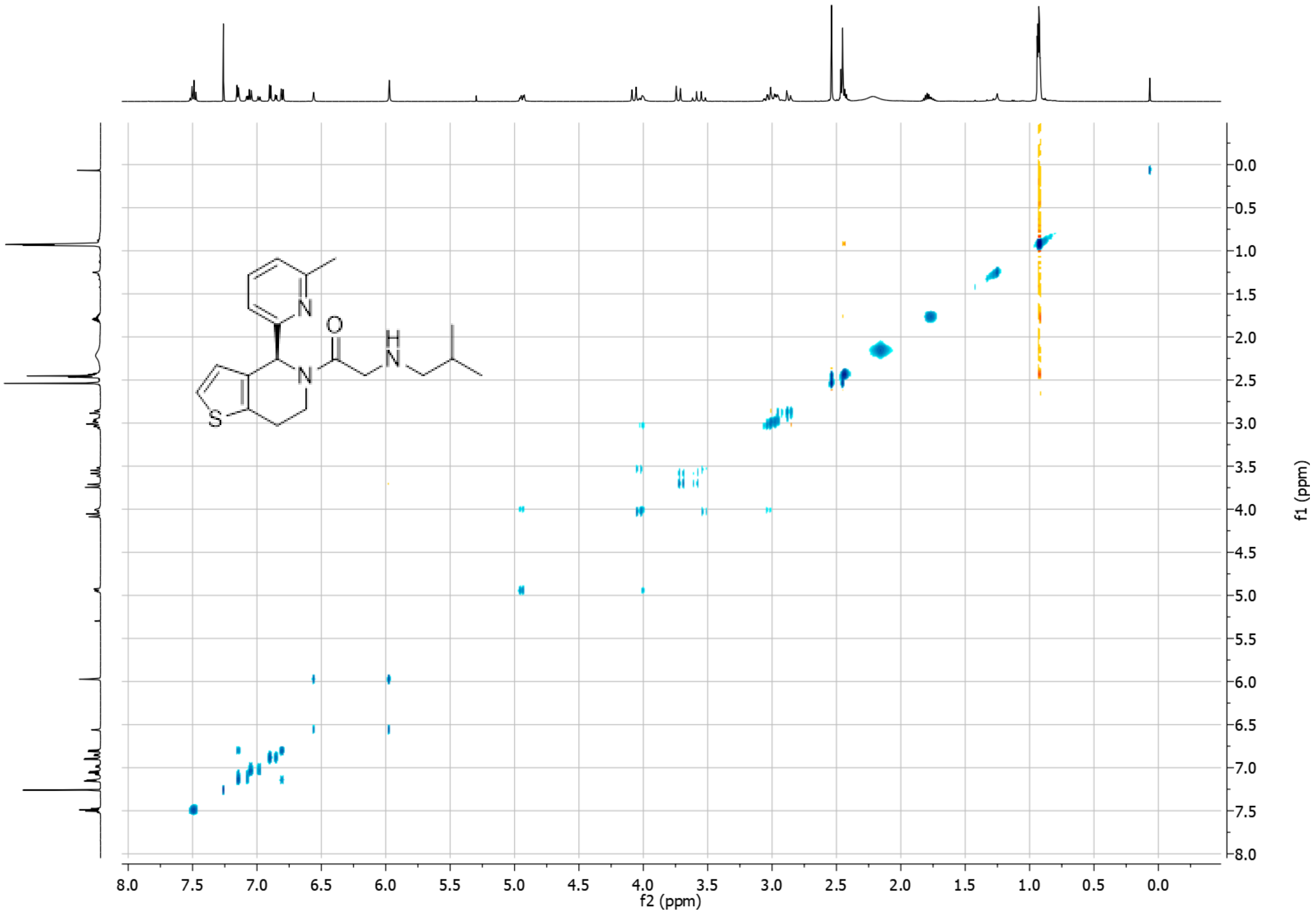

Figure S61. 2D-NOESY (500 MHz, $CDCl_3$, 298K) o **28**.

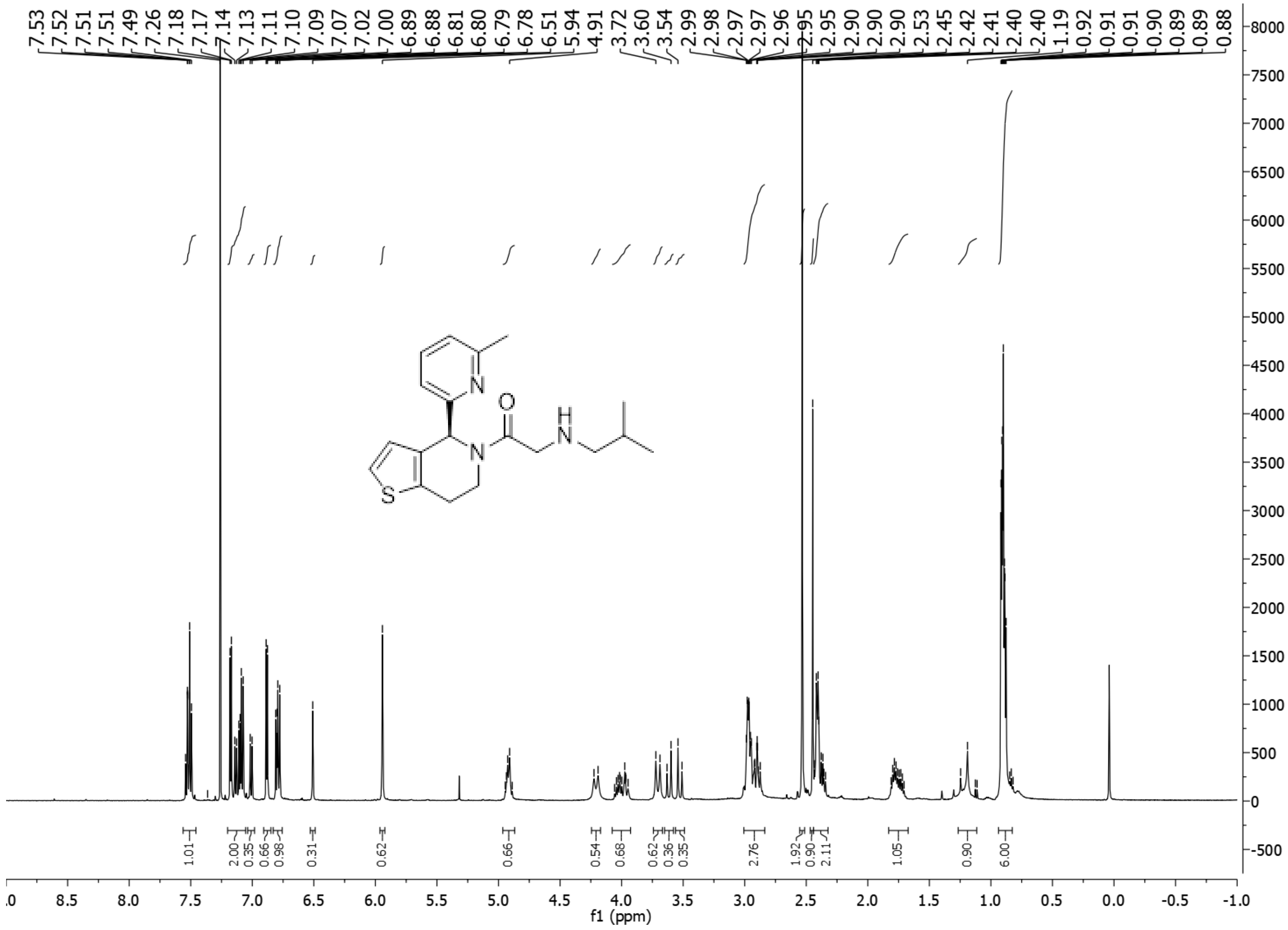

Figure S62. ^{1}H NMR (500 MHz, $CDCl_3$, 218K) of **28**.

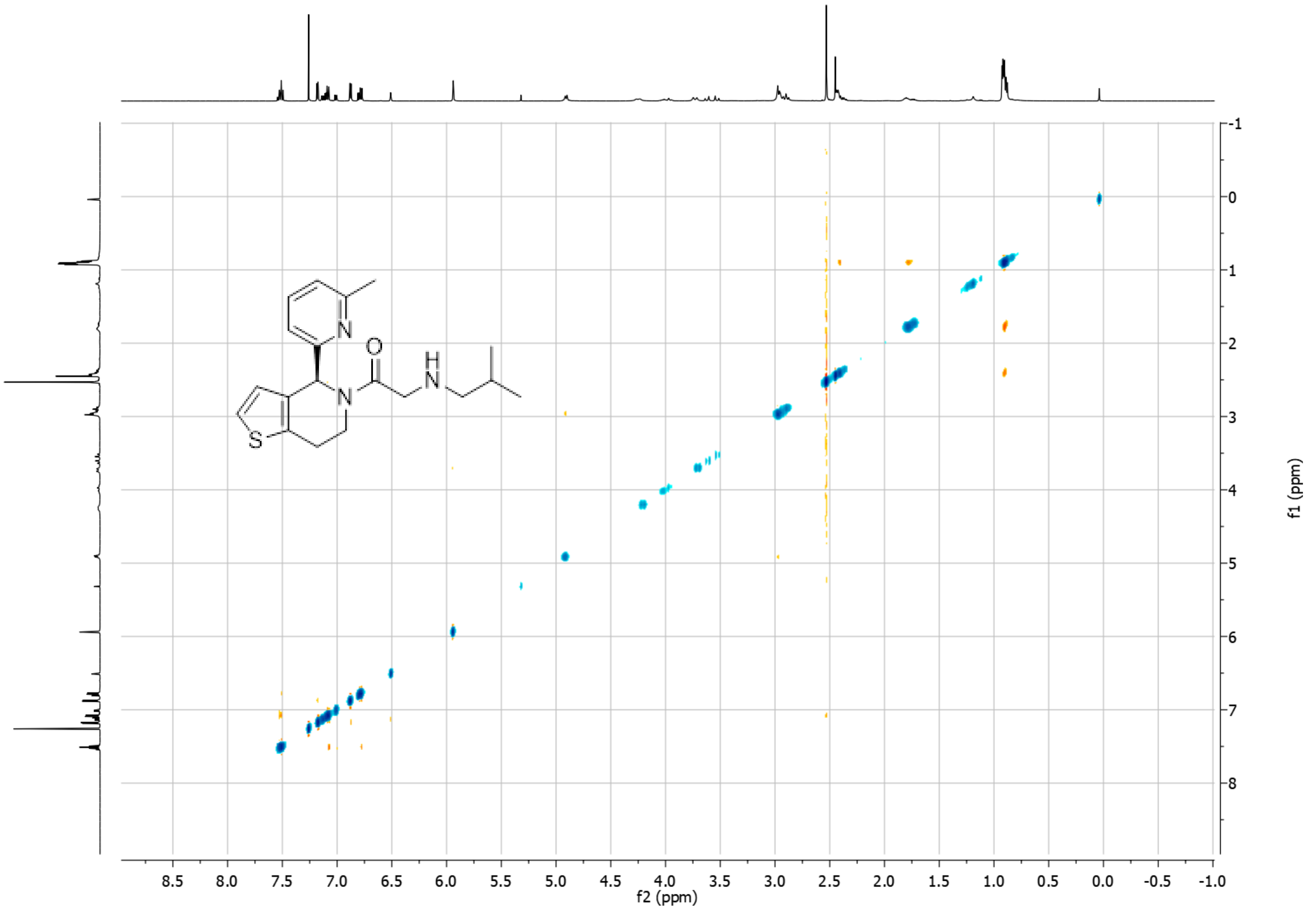

Figure S63. 2D-NOESY (500 MHz, $CDCl_3$, 218K) o **28**.

(*R*)-1-(4-(6-methylpyridin-2-yl)-6,7-dihydrothieno[3,2-*c*]pyridin-5(4*H*)-yl)ethan-1-one (29)

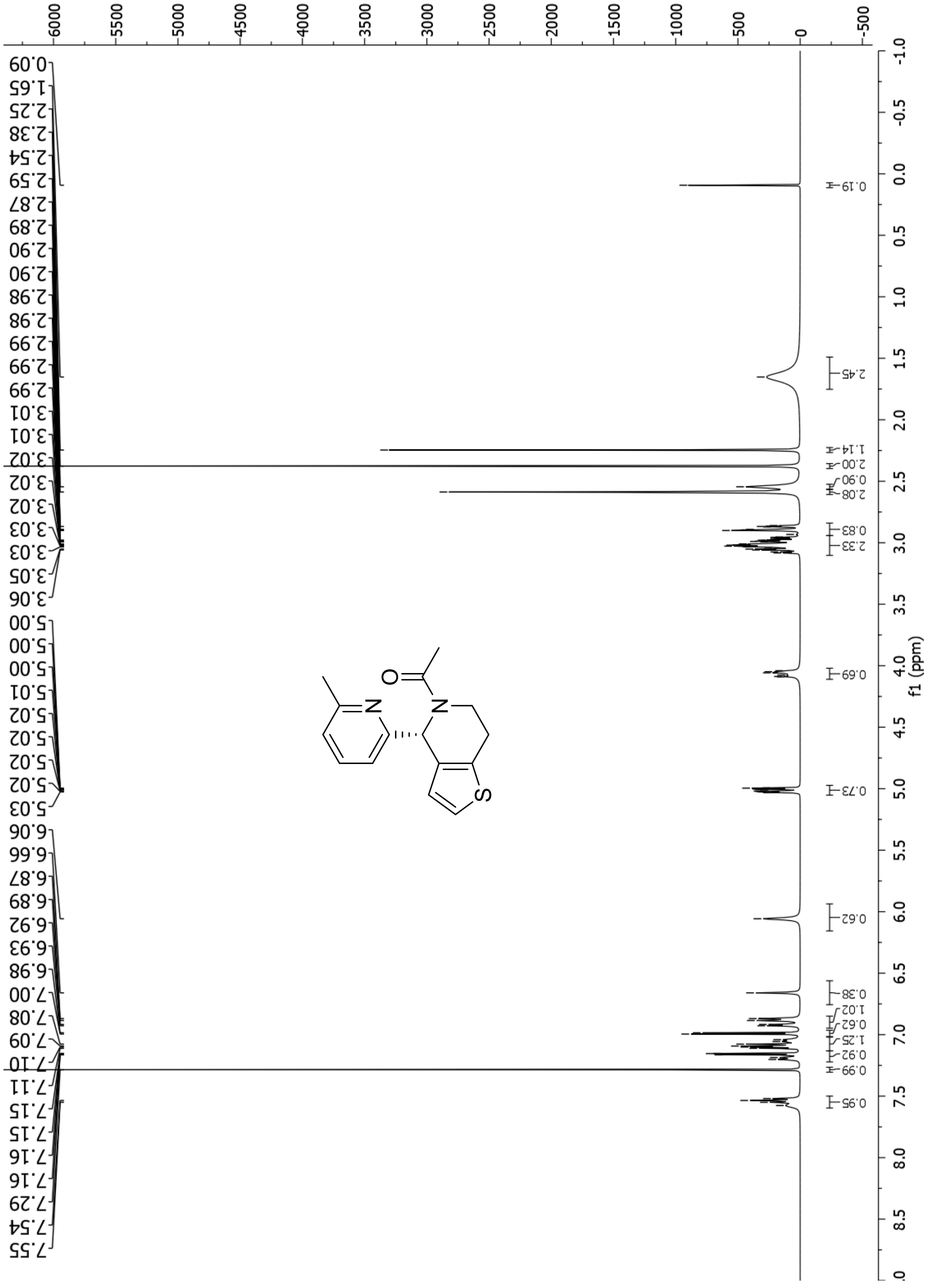

Figure S64. ^{1}H NMR (500 MHz, $CDCl_3$, 298K) of **29**.

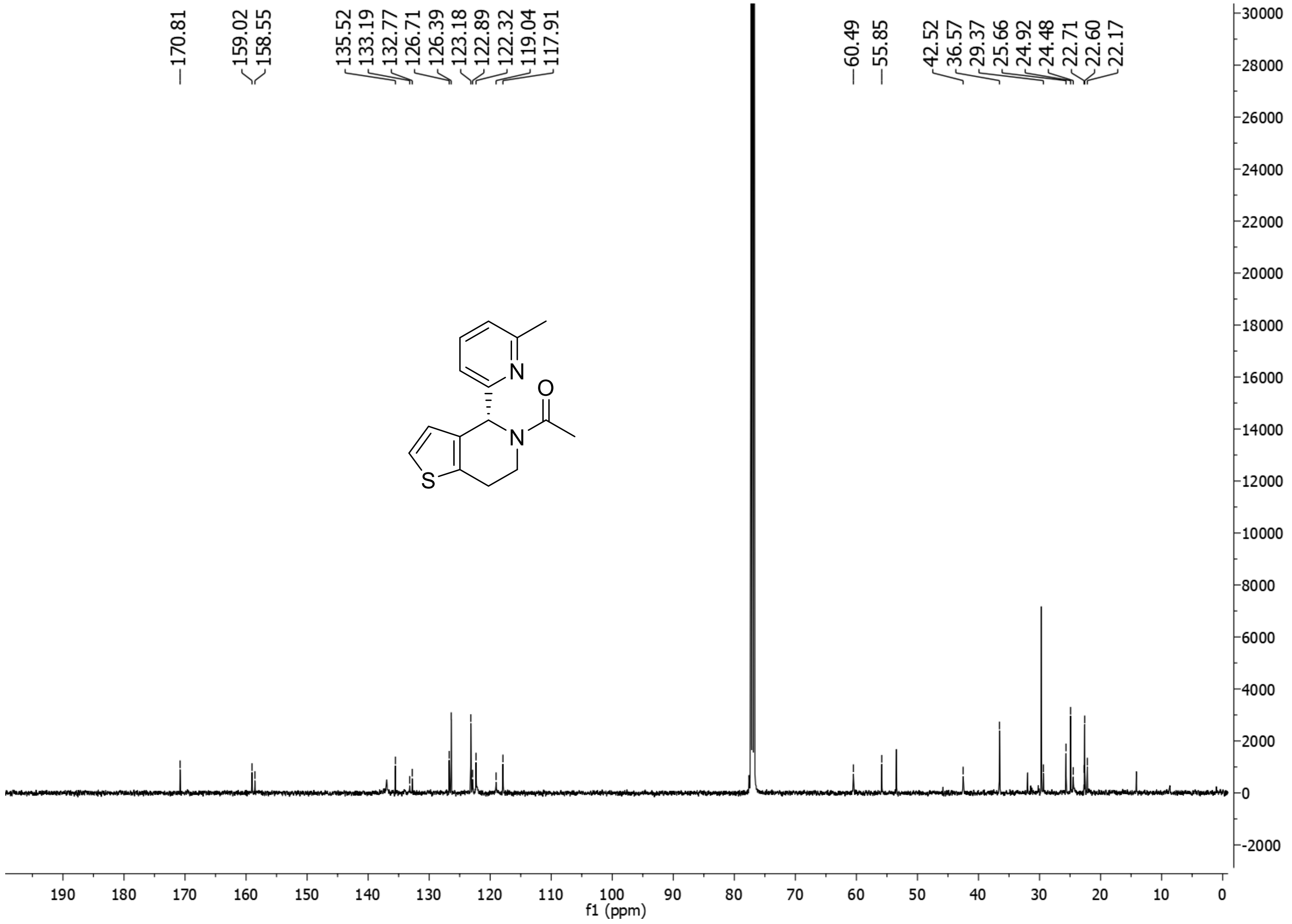

Figure S65. ^{13}C NMR (500 MHz, $CDCl_3$, 298K) of **29**.

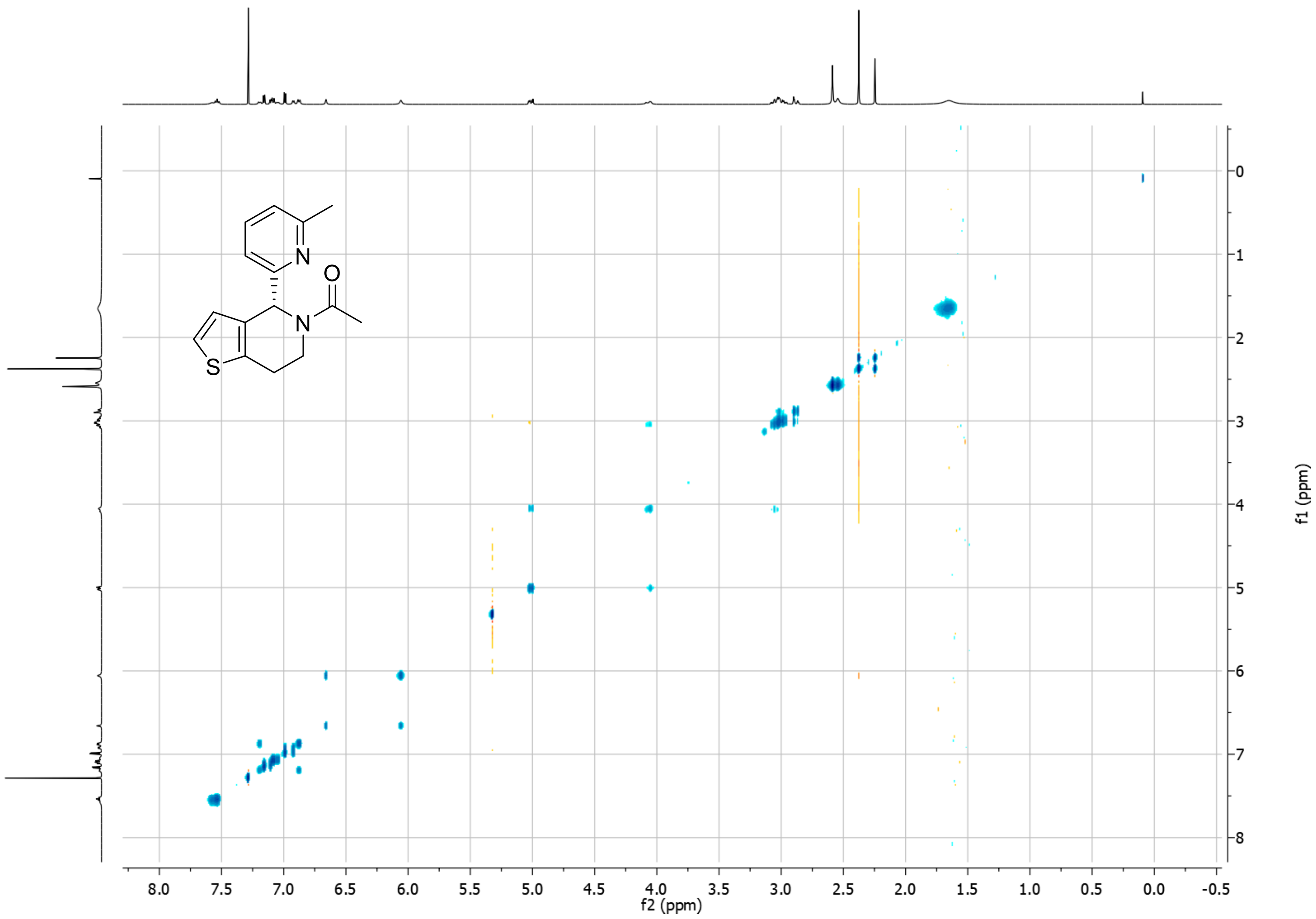

Figure S66. 2D-NOESY (500 MHz, $CDCl_3$, 298K) o **29**.

(*R*)-2-(2-methylpropylamino)-1-[4-(6-methylpyridin-2-yl)-6,7-dihydro-4*H*-thieno[3,2-*c*]pyridin-5-yl]ethanone (30)

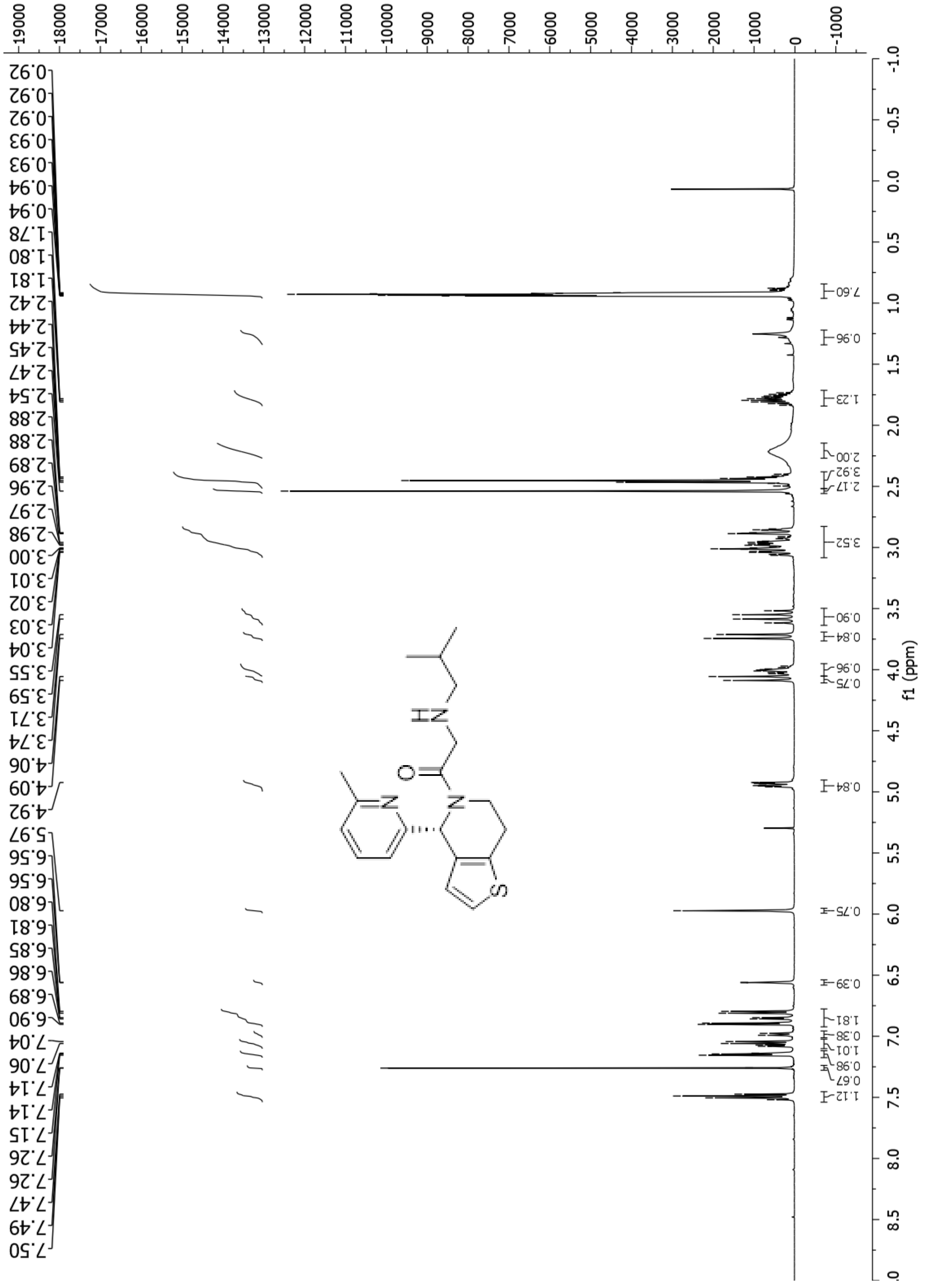

Figure S67. ^{1}H NMR (500 MHz, $CDCl_3$, 298K) of **30**.

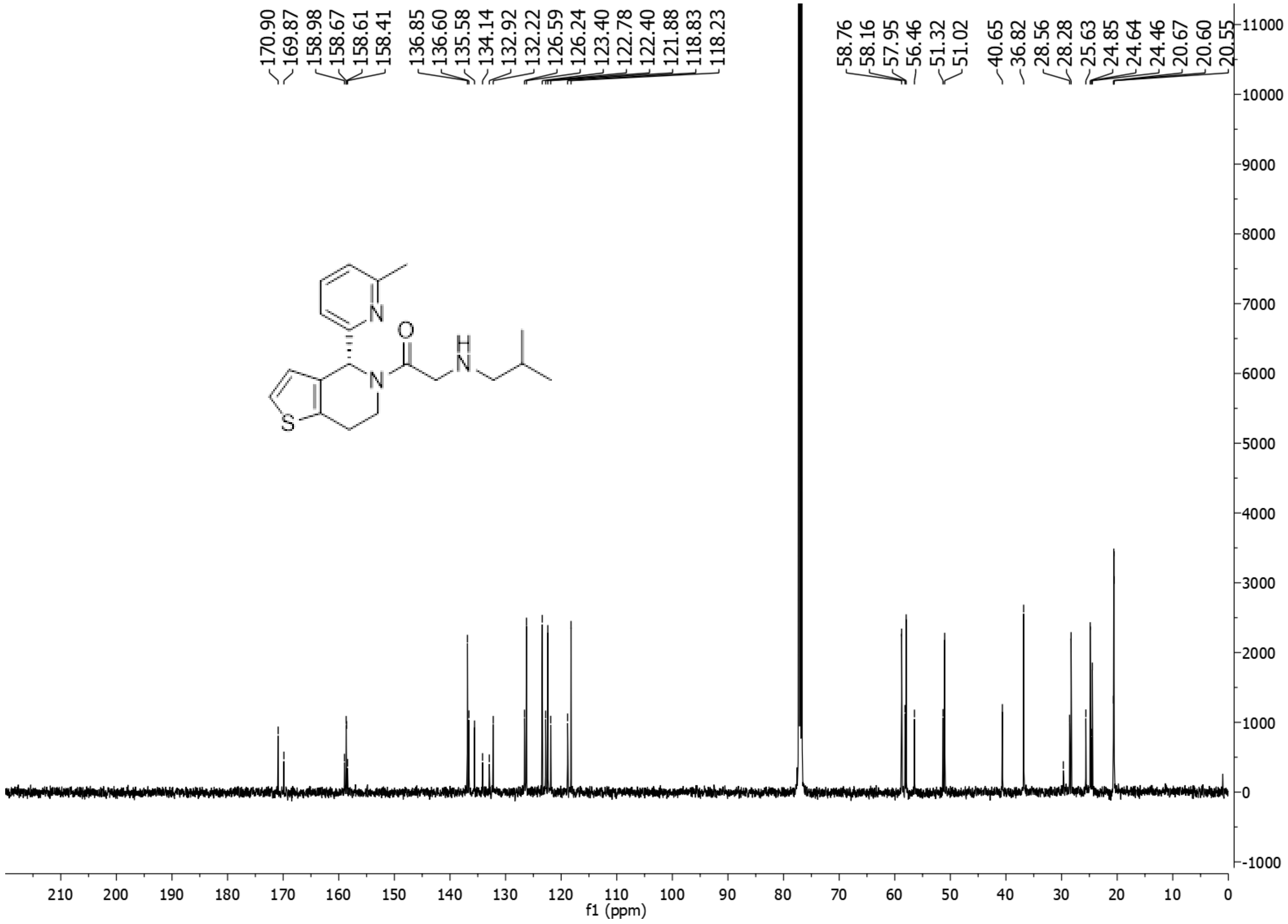

Figure S68. ^{13}C NMR (500 MHz, $CDCl_3$, 298K) of **30**.

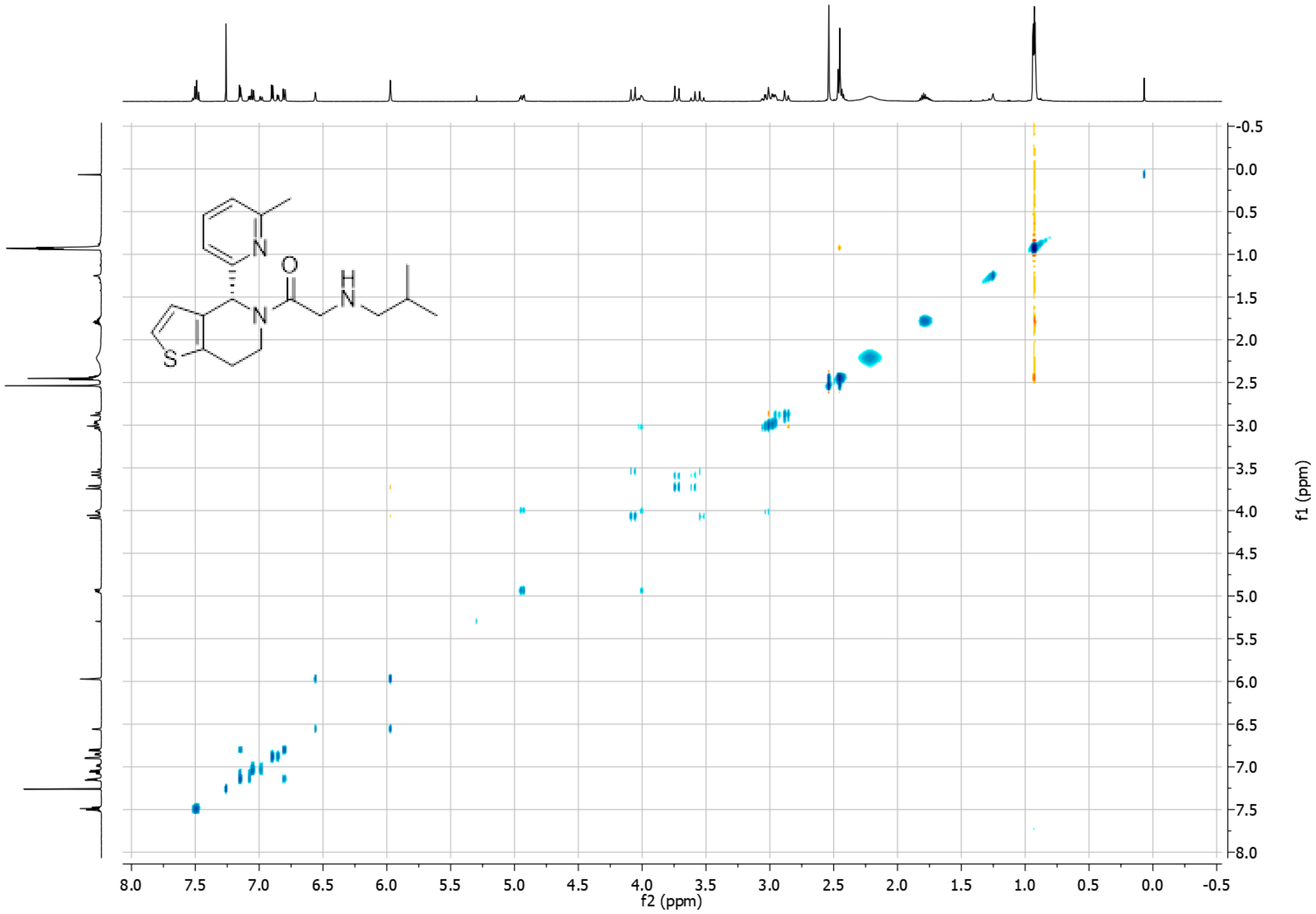

Figure S69. 2D-NOESY (500 MHz, $CDCl_3$, 298K) o **30**.

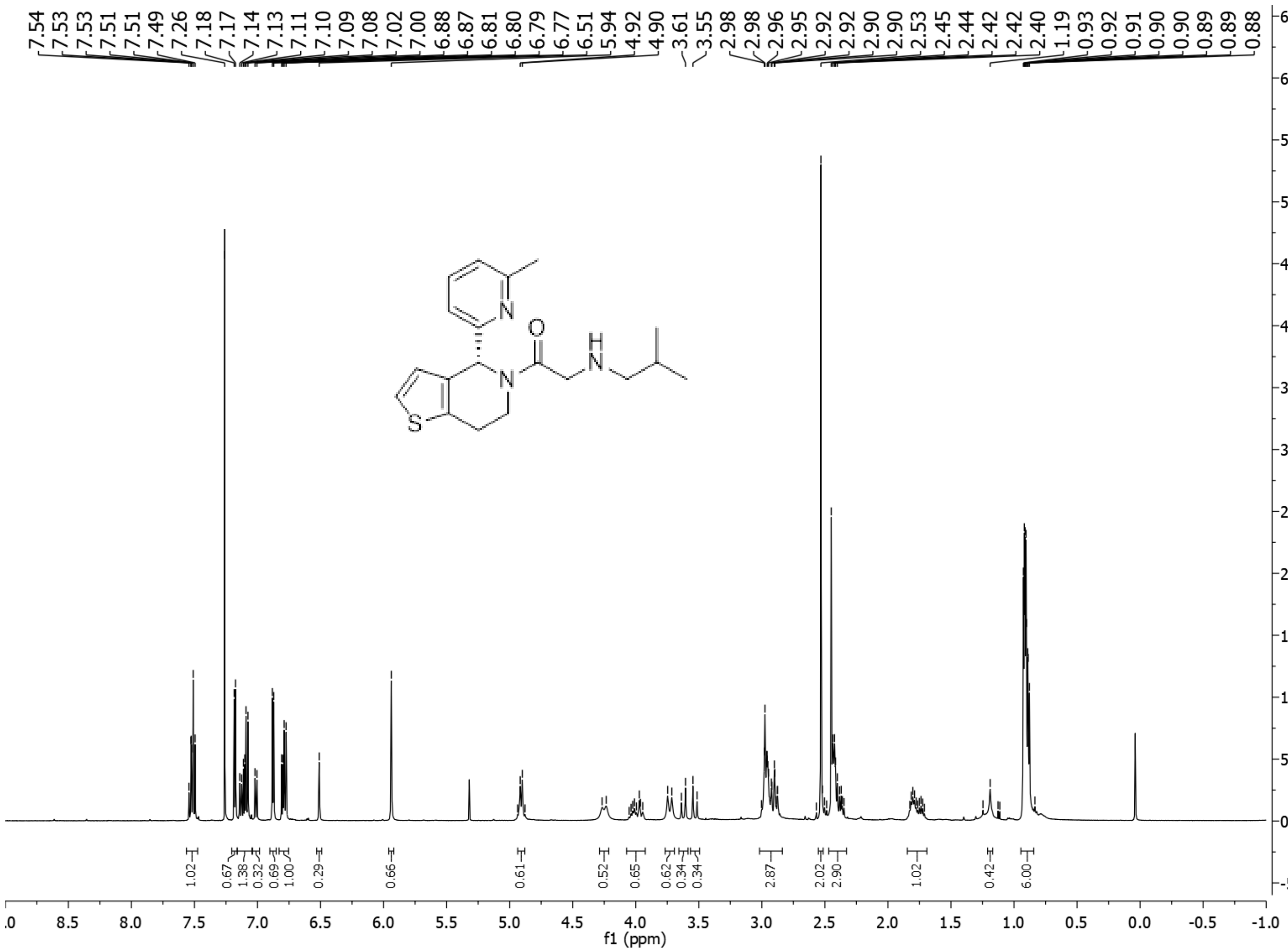

Figure S70. ^{1}H NMR (500 MHz, $CDCl_3$, 218K) of **30**.

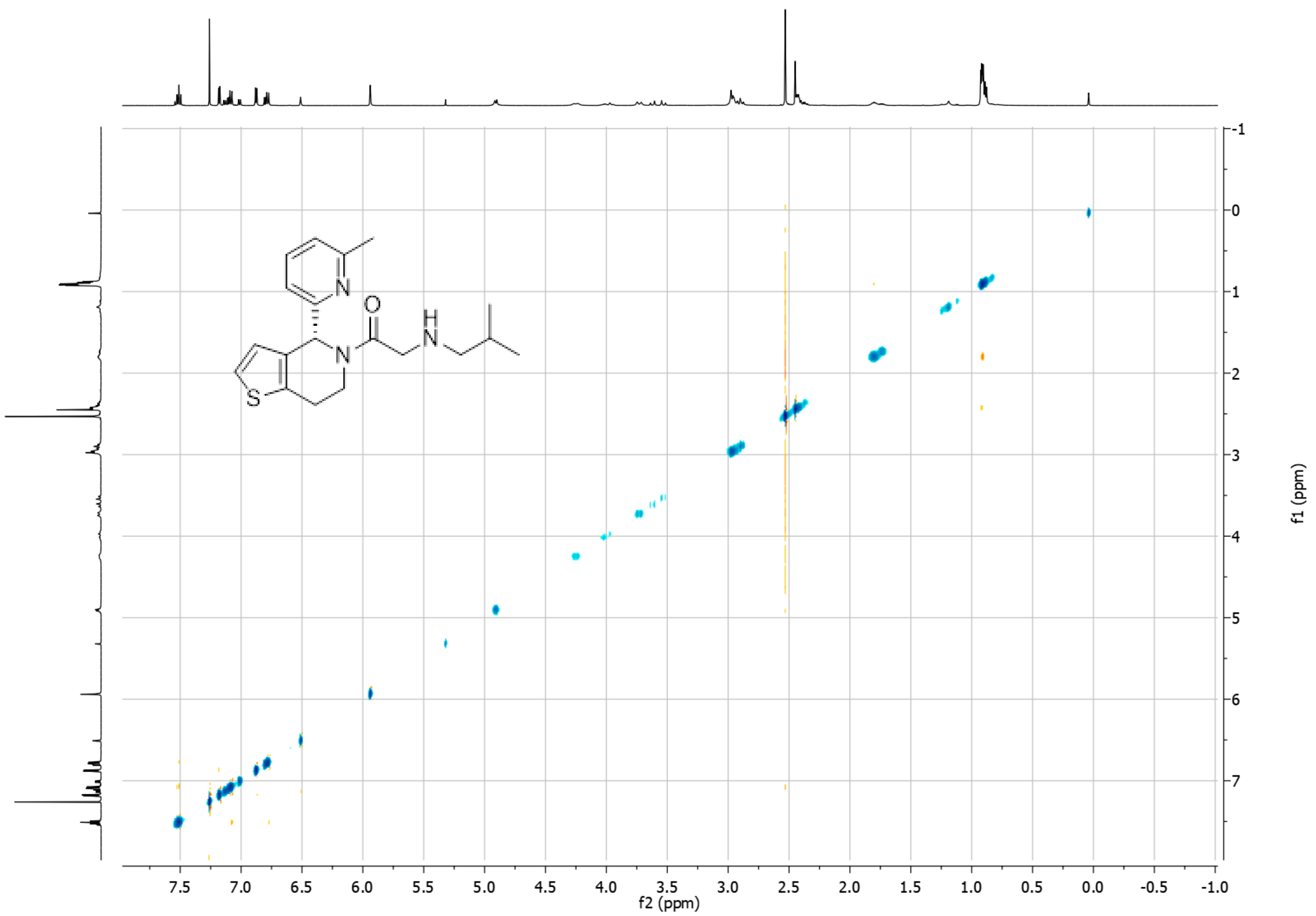

Figure S71. 2D-NOESY (500 MHz, $CDCl_3$, 218K) o **30**.

1-(4-(2-methoxyphenyl)-6,7-dihydrothieno[3,2-*c*]pyridin-5(4*H*)-yl)-2-((2-methylbutyl)amino)ethan-1-one (34)

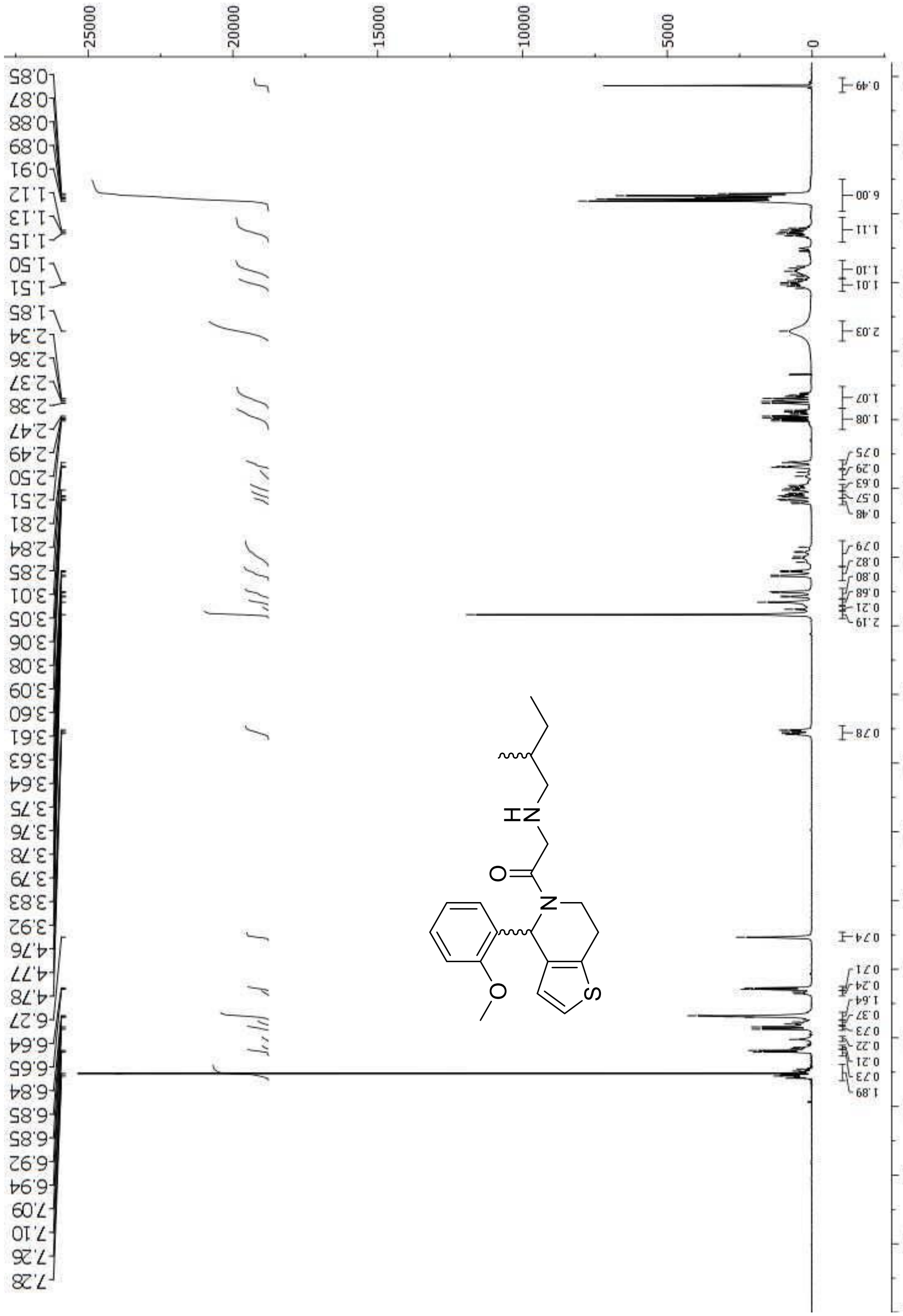

Figure S72. ^{1}H NMR (500 MHz, $CDCl_3$, 298K) of **34**.

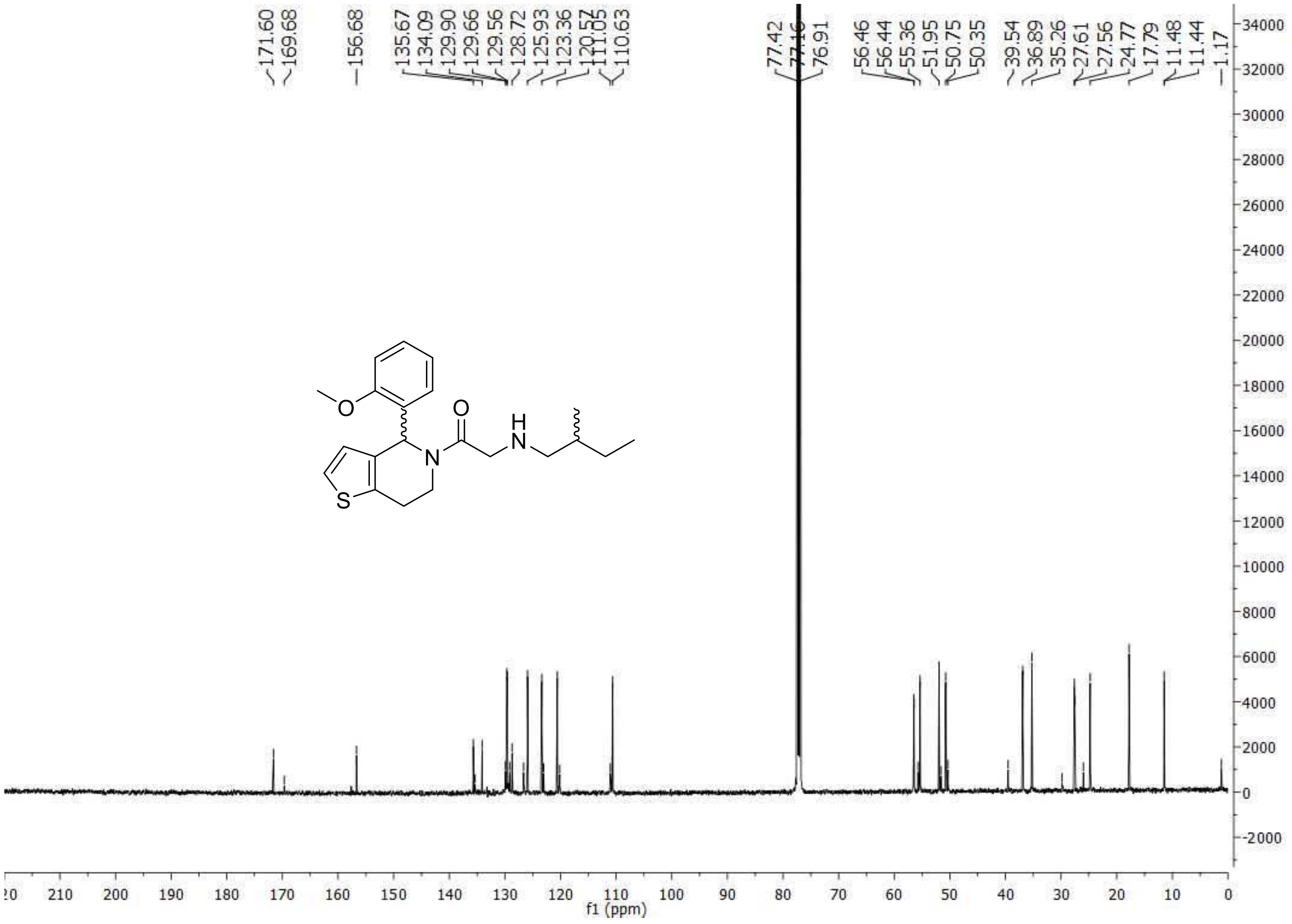

Figure S73. ^{13}C NMR (500 MHz, $CDCl_3$, 298K) of **34**.

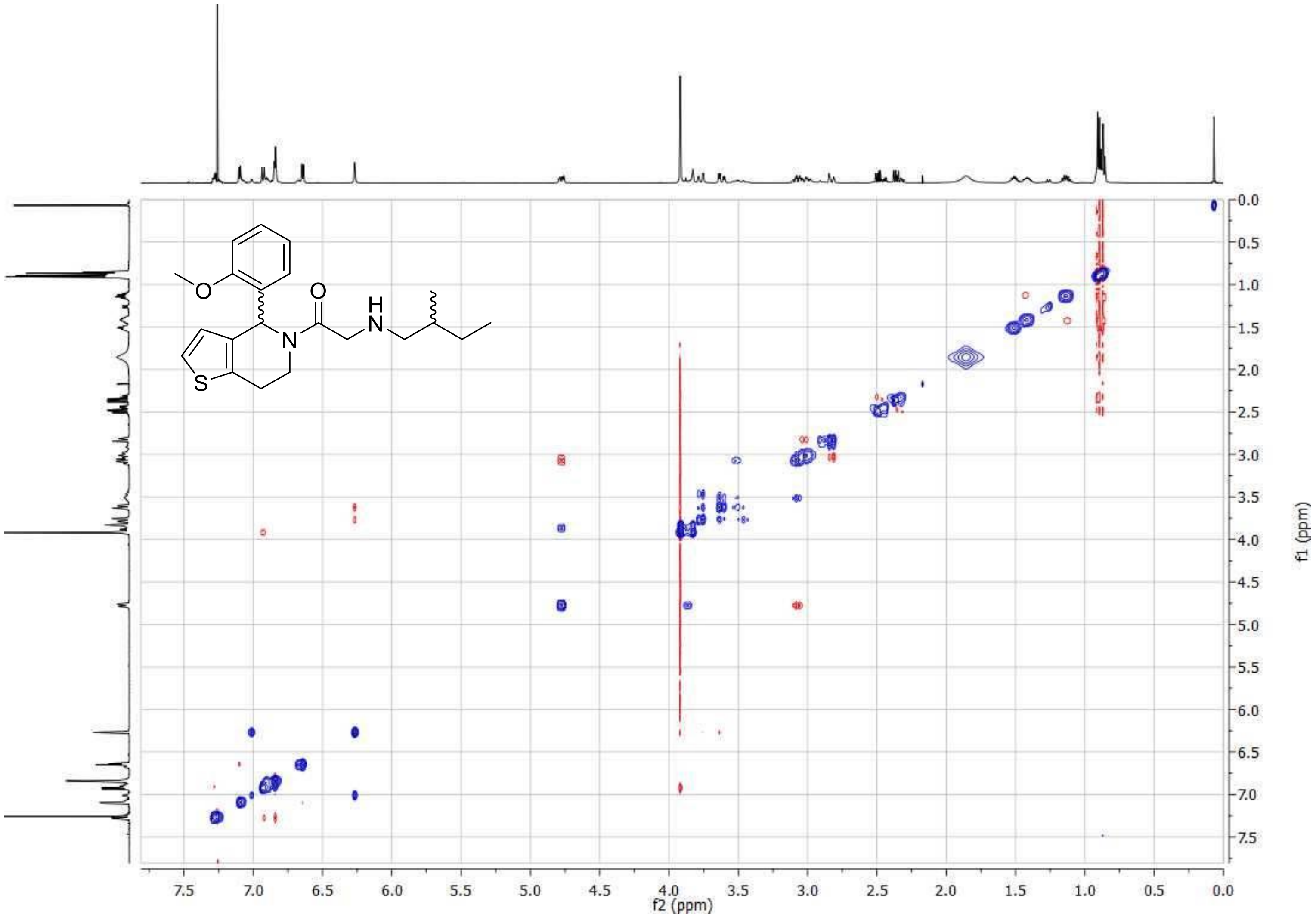

Figure S74. 2D-NOESY (500 MHz, $CDCl_3$, 298K) o **34**.

1-(4-(2-hydroxyphenyl)-6,7-dihydrothieno[3,2-*c*]pyridin-5(4*H*)-yl)-2-((2-methylbutyl)amino)ethan-1-one (35)

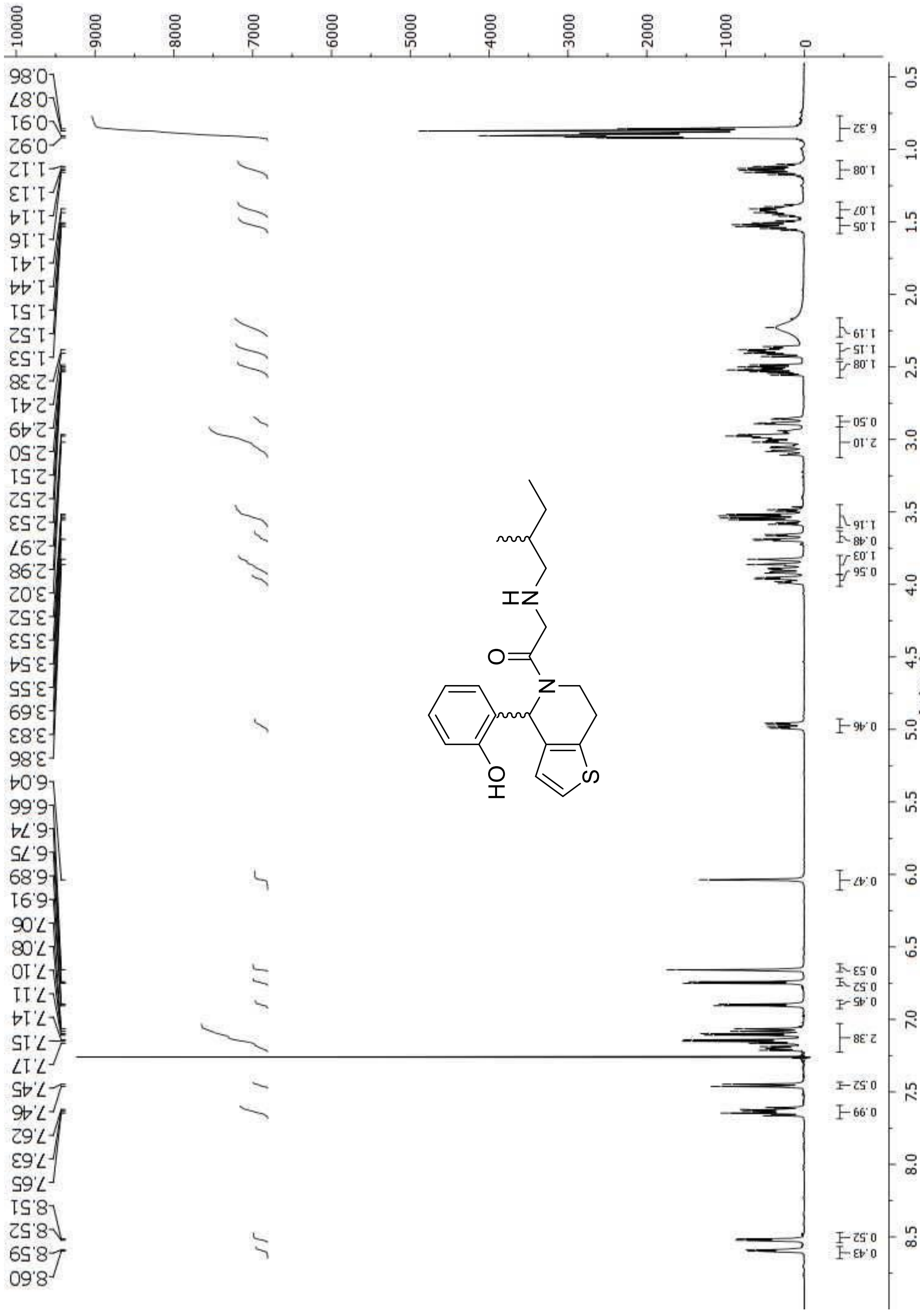

Figure S75. ^{1}H NMR (500 MHz, $CDCl_3$, 298K) of **35**.

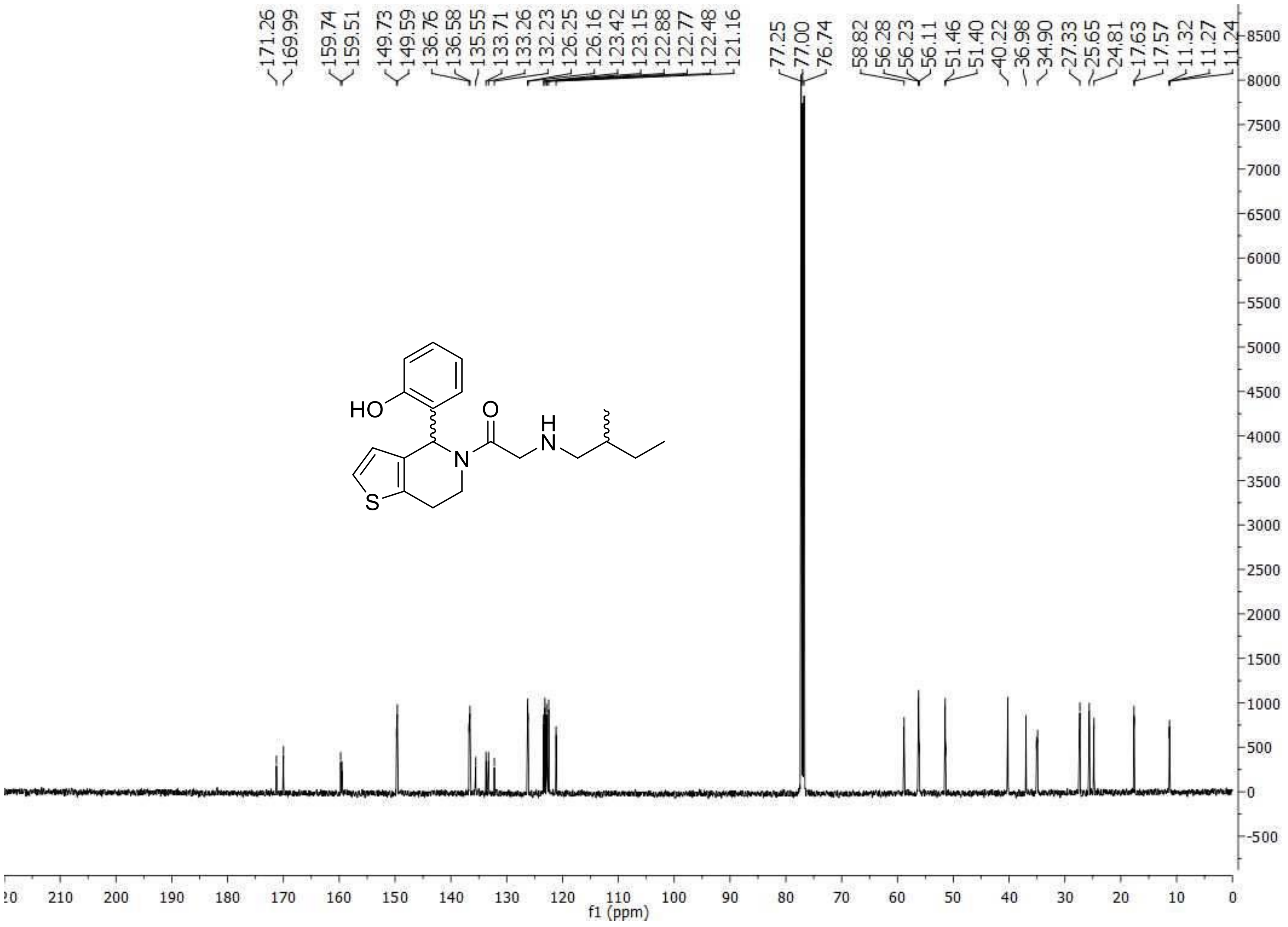

Figure S76. ^{13}C NMR (500 MHz, $CDCl_3$, 298K) of **35**.

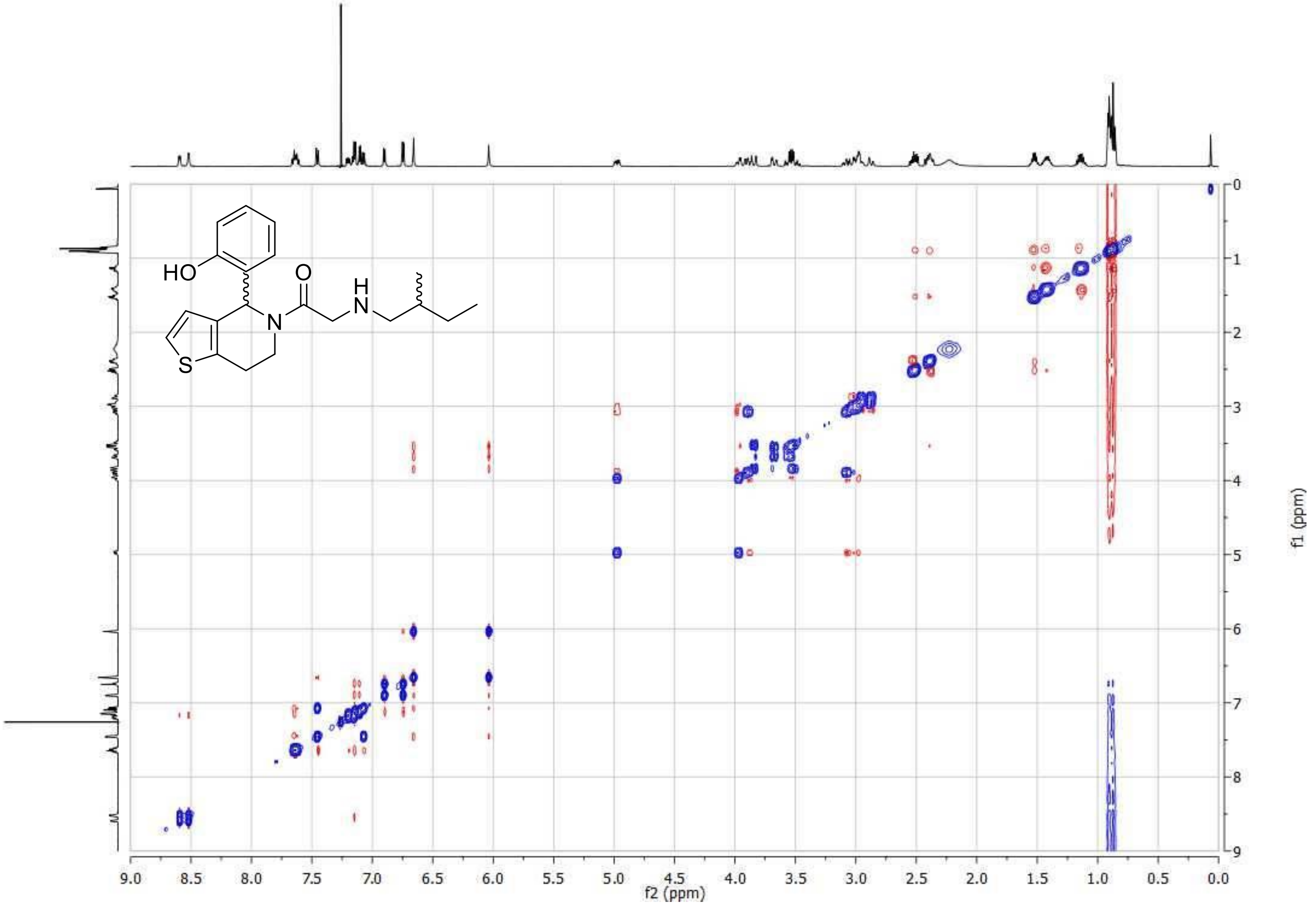

Figure S77. 2D-NOESY (500 MHz, $CDCl_3$, 298K) of **35**.

1-(4-cyclohexyl-6,7-dihydrothieno[3,2-*c*]pyridin-5(4*H*)-yl)-2-((2-methylbutyl)amino)ethan-1-one (36)

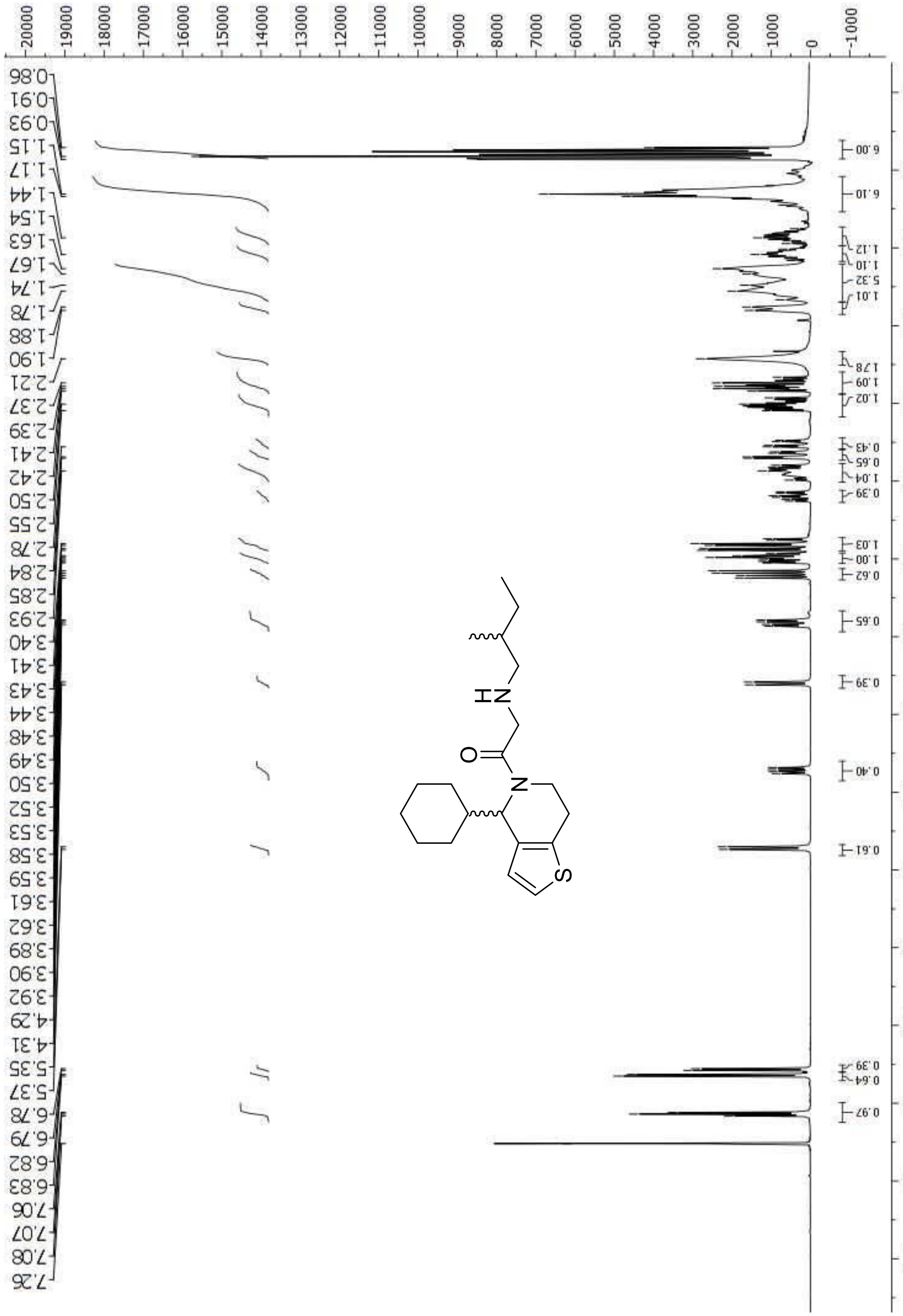

Figure S78. ^{1}H NMR (500 MHz, $CDCl_3$, 298K) of **36**.

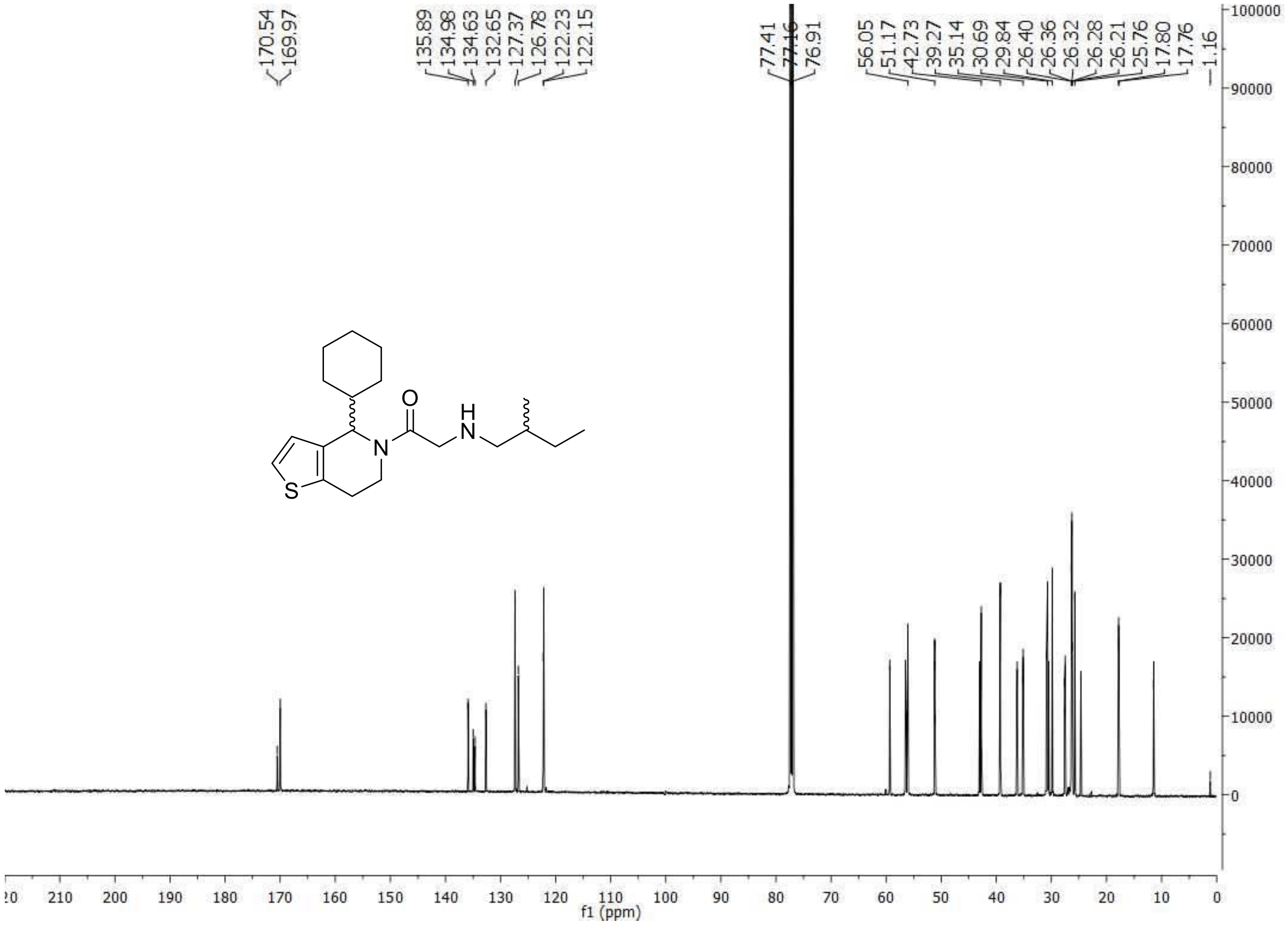

Figure S79. ^{13}C NMR (500 MHz, $CDCl_3$, 298K) of **36**.

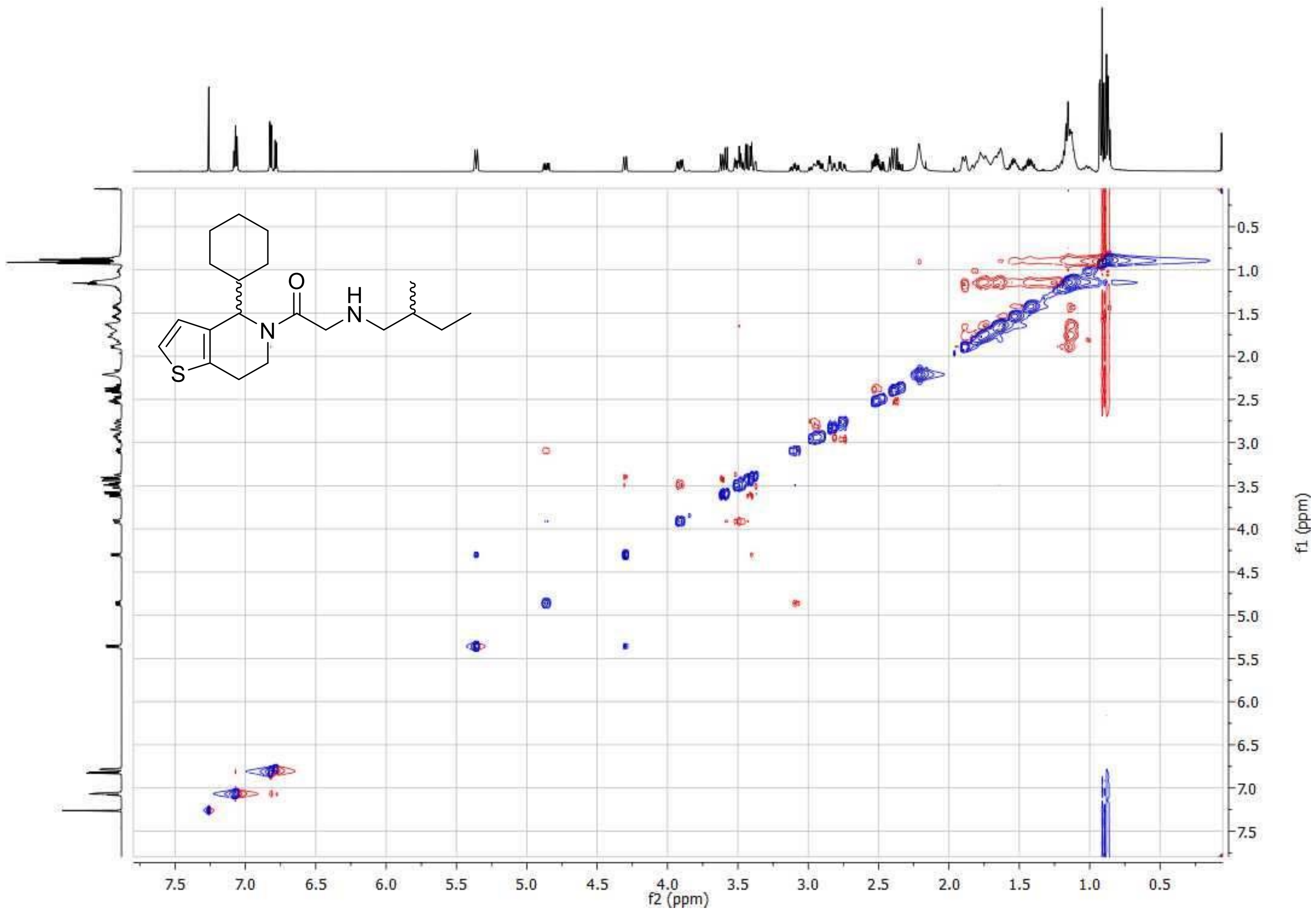

Figure S80. 2D-NOESY (500 MHz, $CDCl_3$, 298K) of **36**.

1-(1-phenyl-3,4-dihydroisoquinolin-2(1*H*)-yl)ethan-1-one (37)

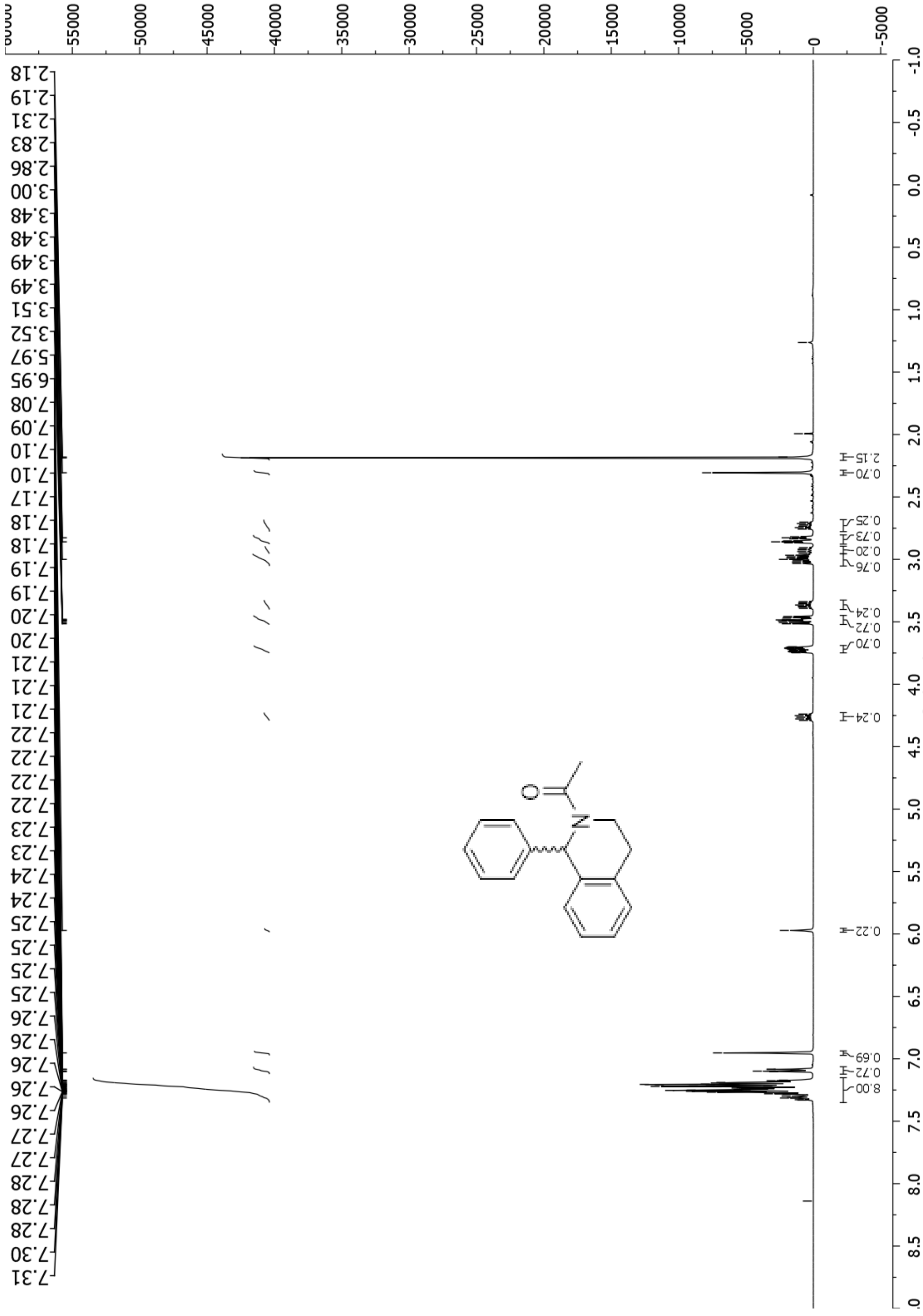

Figure S81. ^{1}H NMR (500 MHz, $CDCl_3$, 298K) of **37**.

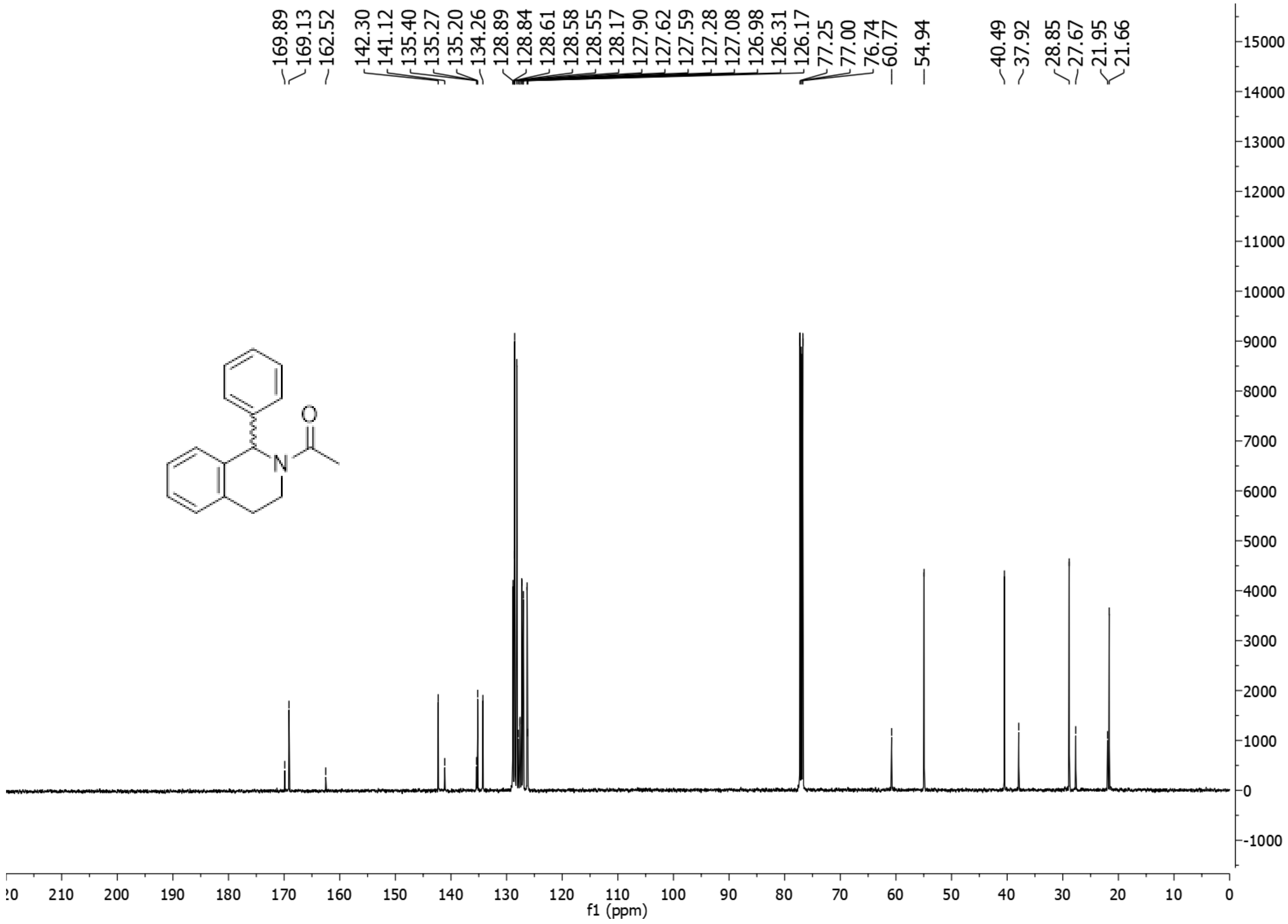

Figure S82. ^{13}C NMR (500 MHz, $CDCl_3$, 298K) of **37**.

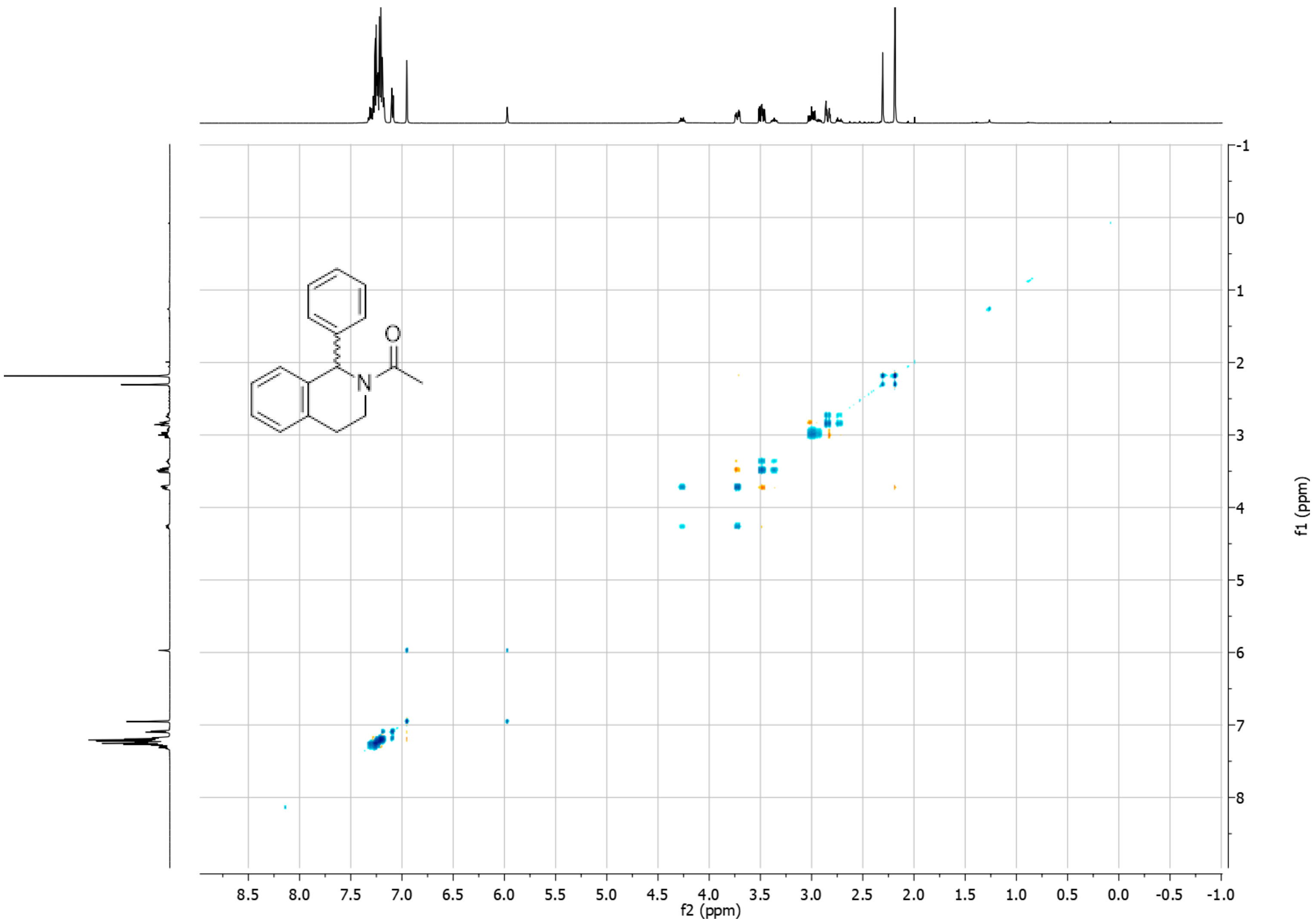

Figure S83. 2D-NOESY (500 MHz, $CDCl_3$, 298K) of **37**.

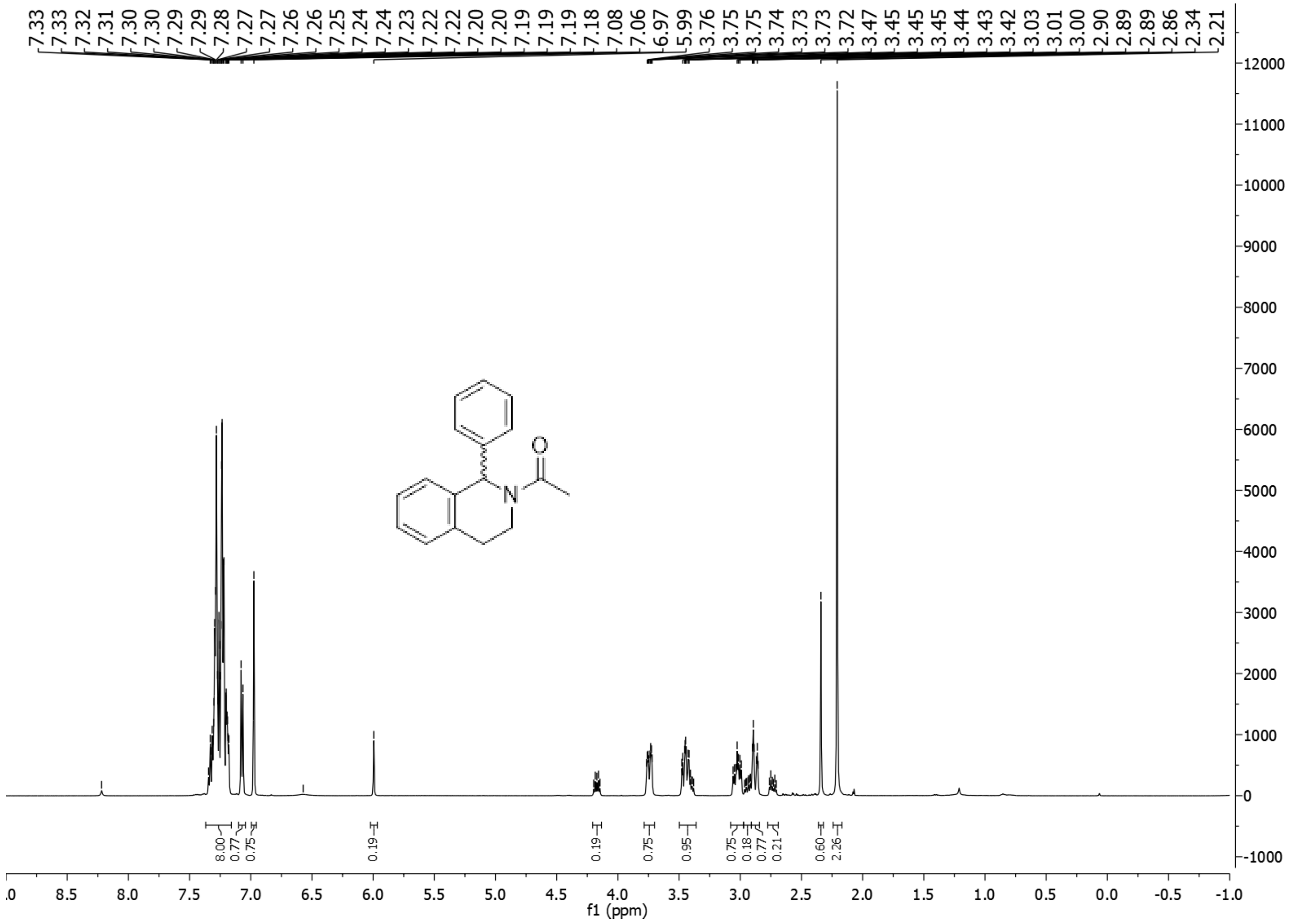

Figure S84. ^{1}H NMR (500 MHz, $CDCl_3$, 218K) of **37**.

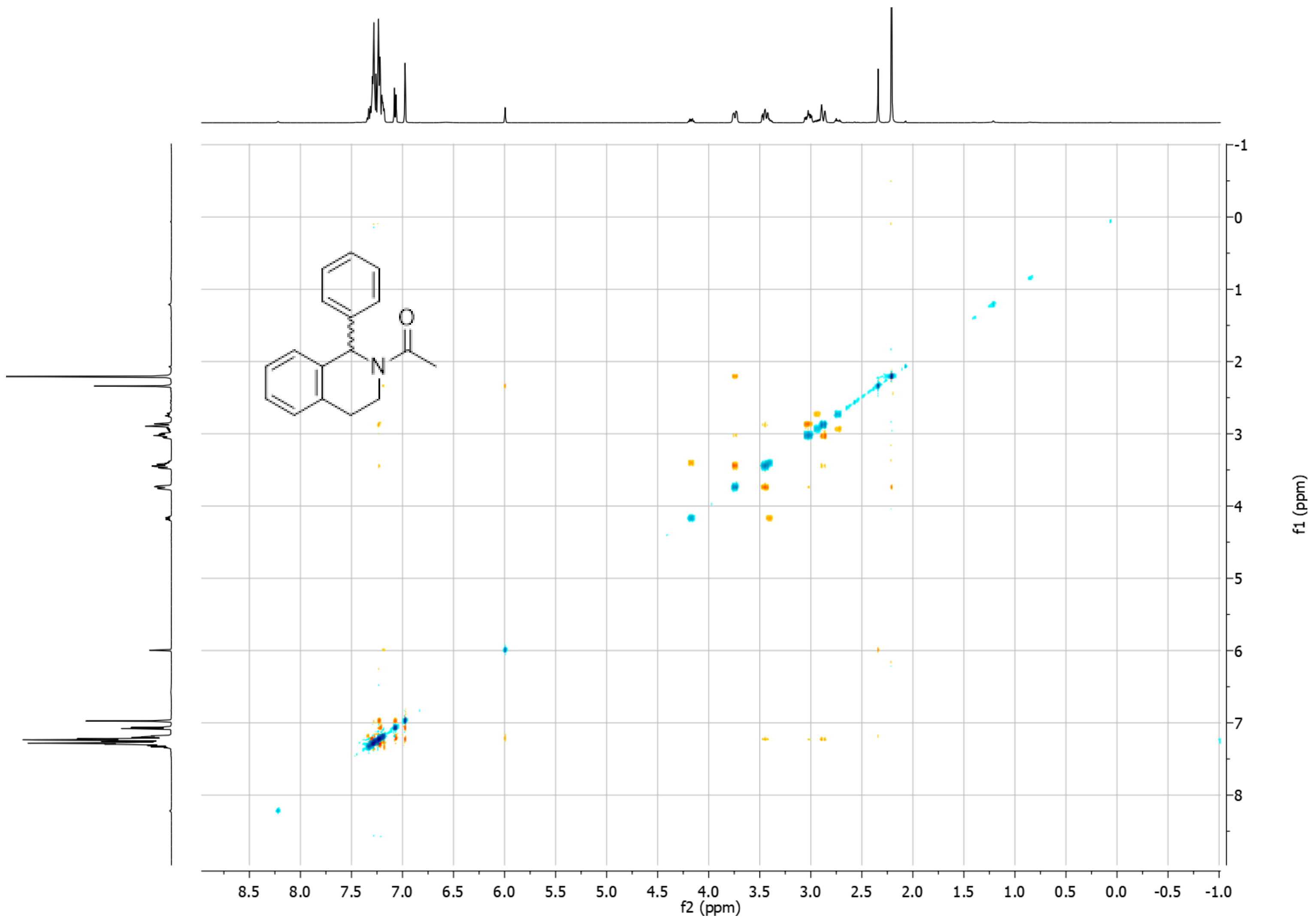

Figure S85. 2D-NOESY (500 MHz, $CDCl_3$, 218K) of **37**.

2-((2-methylbutyl)amino)-1-(1-phenyl-3,4-dihydroisoquinolin-2(1*H*)-yl)ethan-1-one (38)

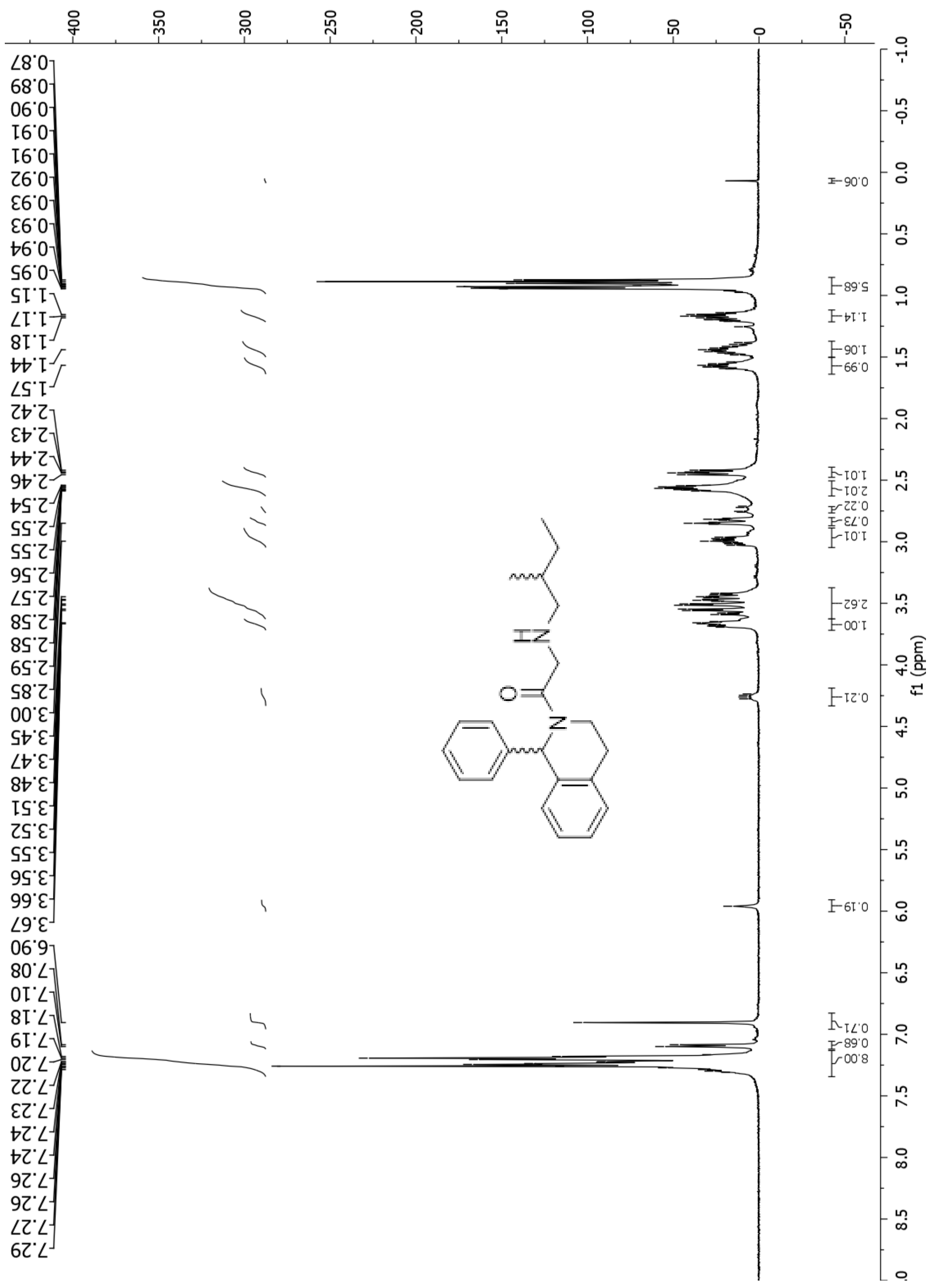

Figure S86. ^{1}H NMR (500 MHz, $CDCl_3$, 298K) of **38**.

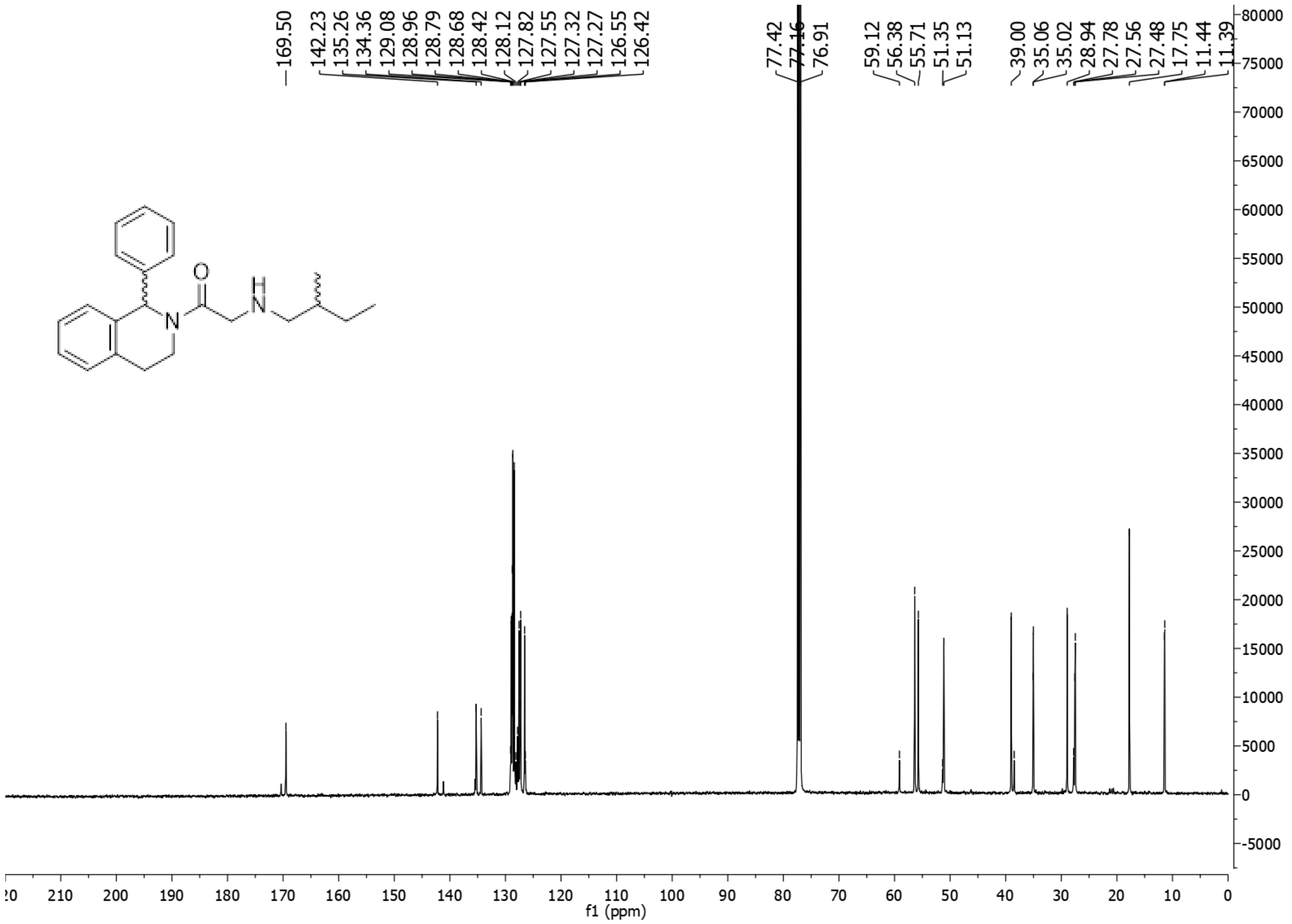

7Figure S87. ^{13}C NMR (500 MHz, $CDCl_3$, 298K) of **38**.

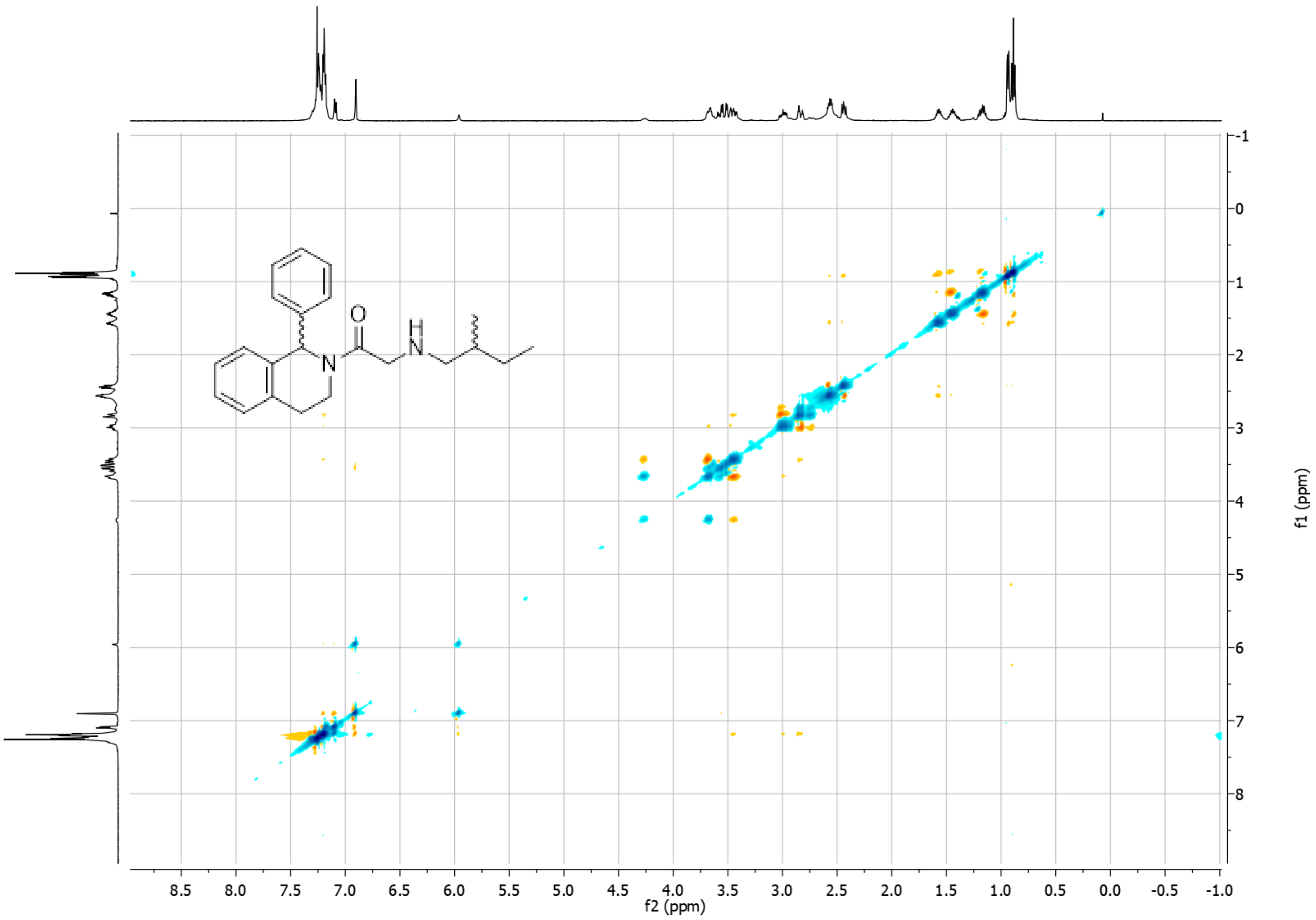

Figure S88. 2D-NOESY (500 MHz, $CDCl_3$, 298K) of **38**.

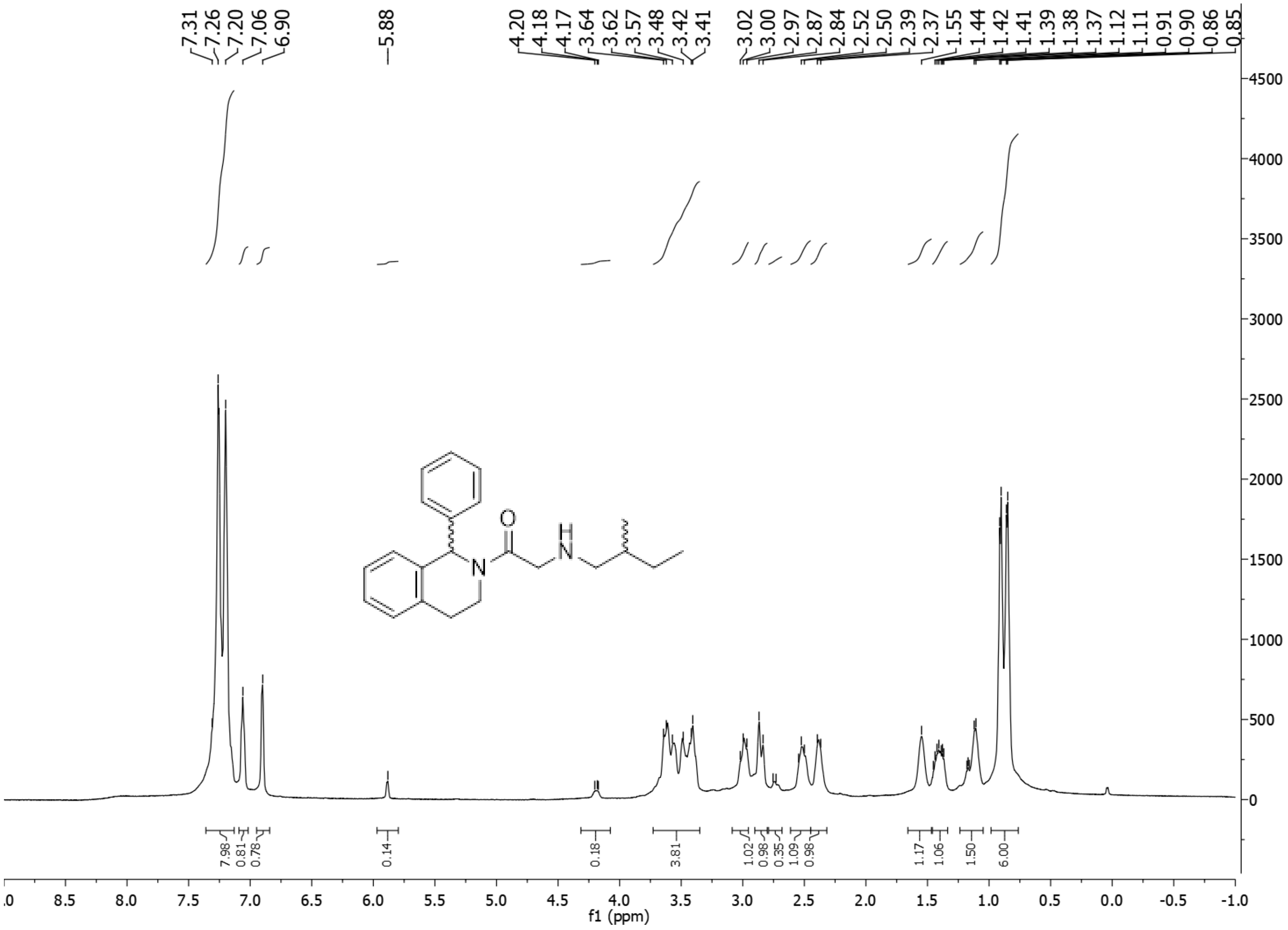

Figure S89. ^{13}C NMR (500 MHz, $CDCl_3$, 218K) of **38**.

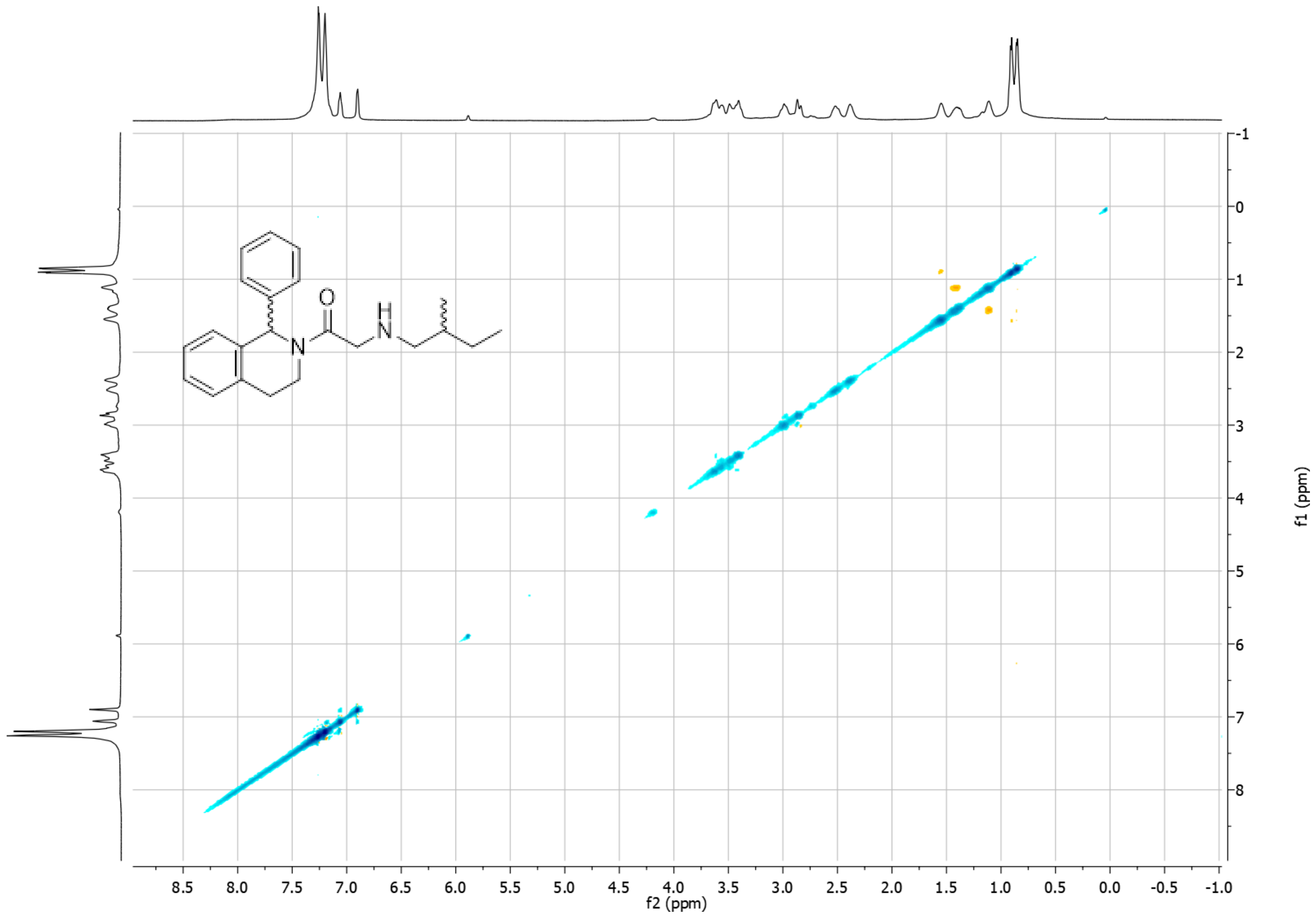

Figure S90. 2D-NOESY (500 MHz, $CDCl_3$, 218K) of **38**.

2-((2-methylpropyl)amino)-1-(1-phenyl-3,4-dihydroisoquinolin-2(1*H*)-yl)ethan-1-one (39)

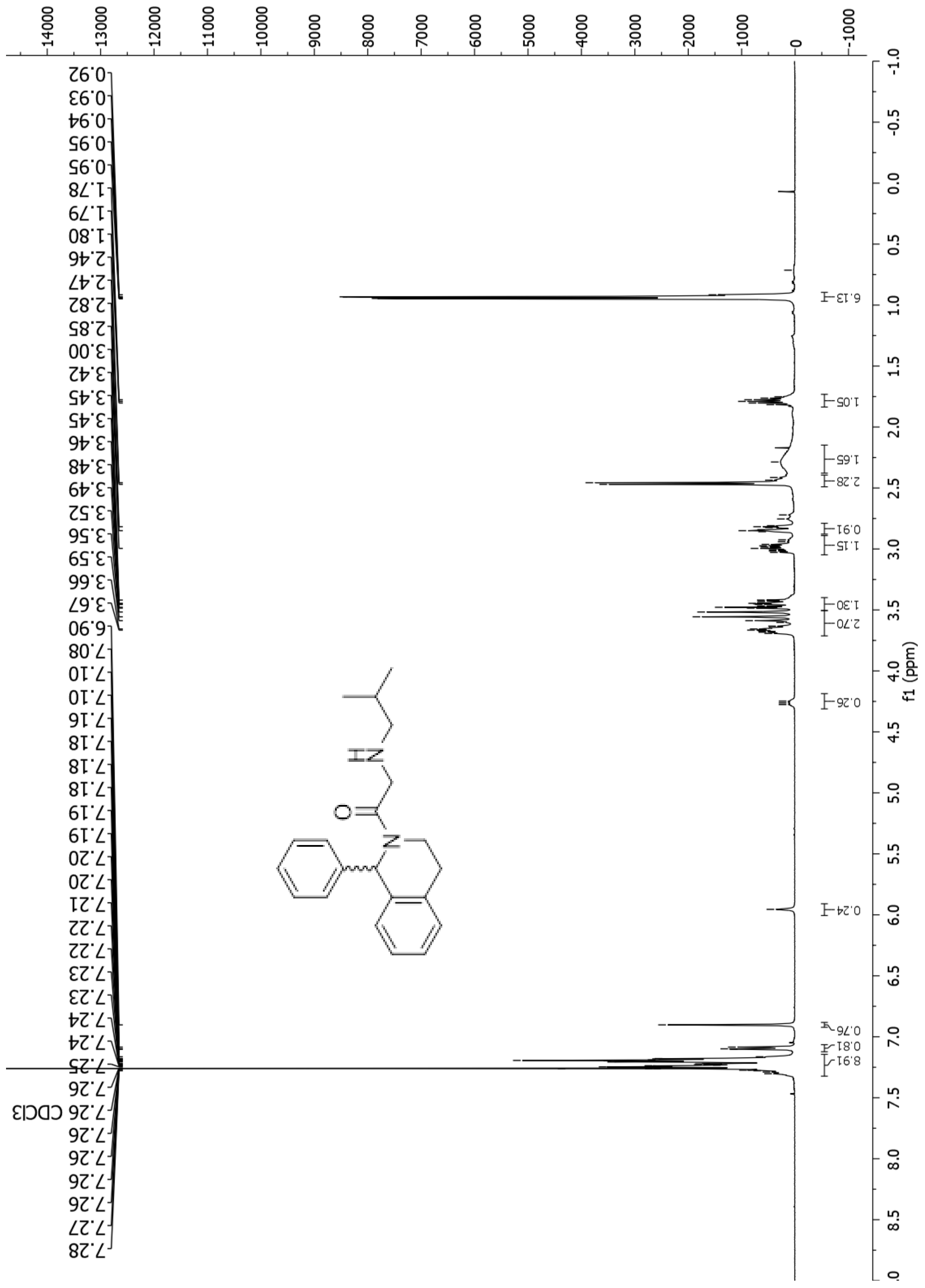

Figure S91. ^{1}H NMR (500 MHz, $CDCl_3$, 298K) of **39**.

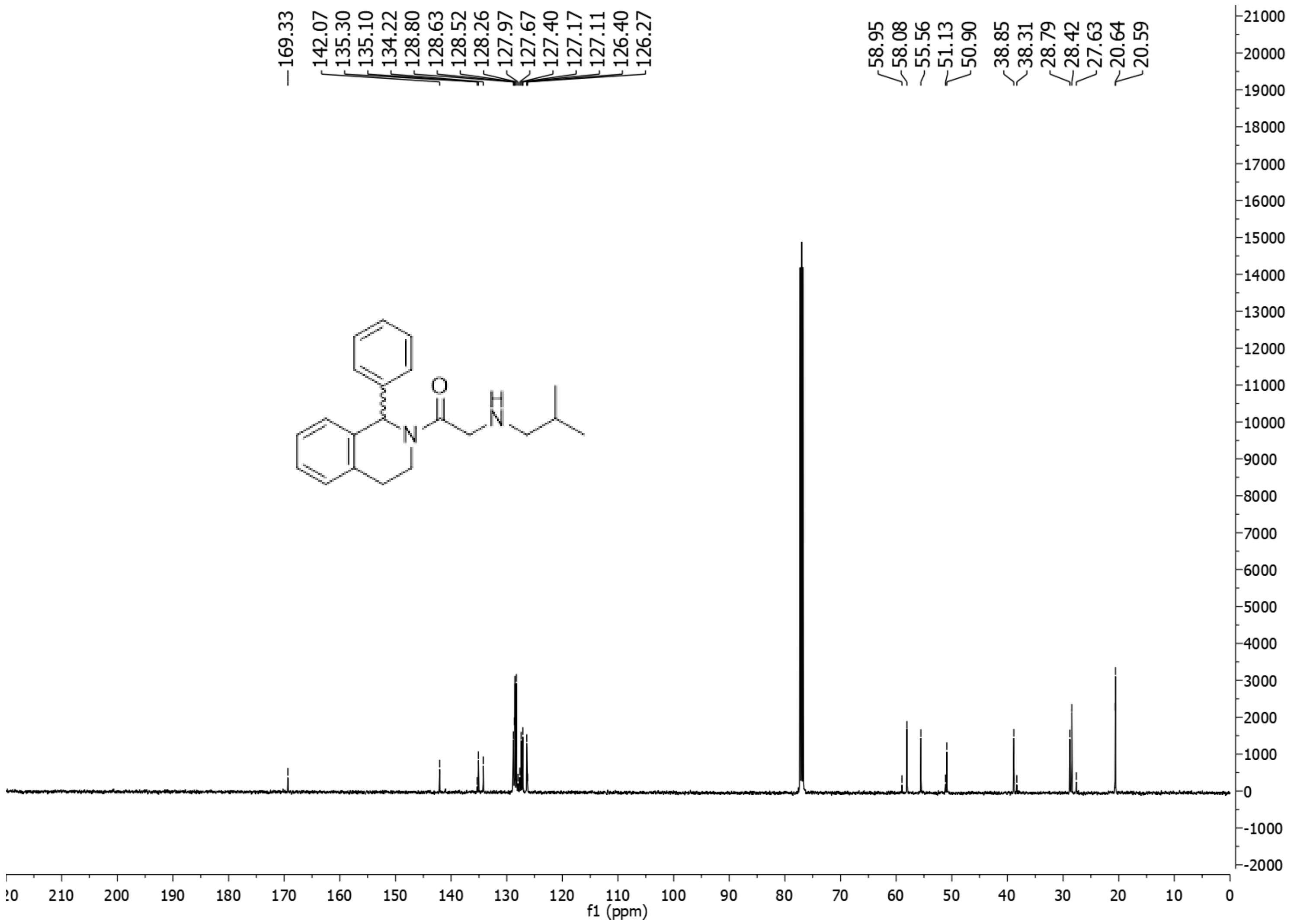

Figure S92. ^{13}C NMR (500 MHz, $CDCl_3$, 298K) of **39**.

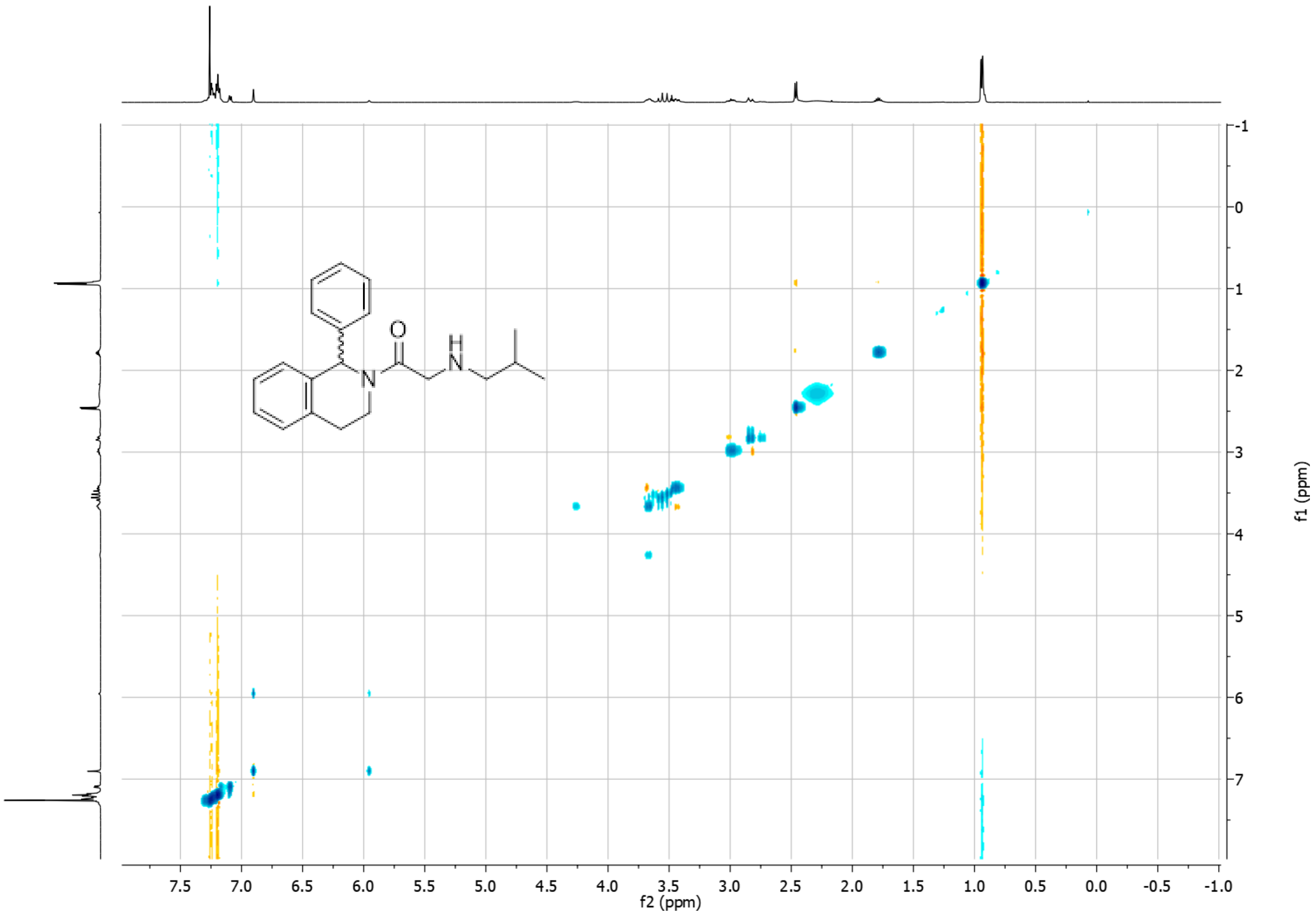

Figure S93. 2D-NOESY (500 MHz, $CDCl_3$, 298K) of **39**.

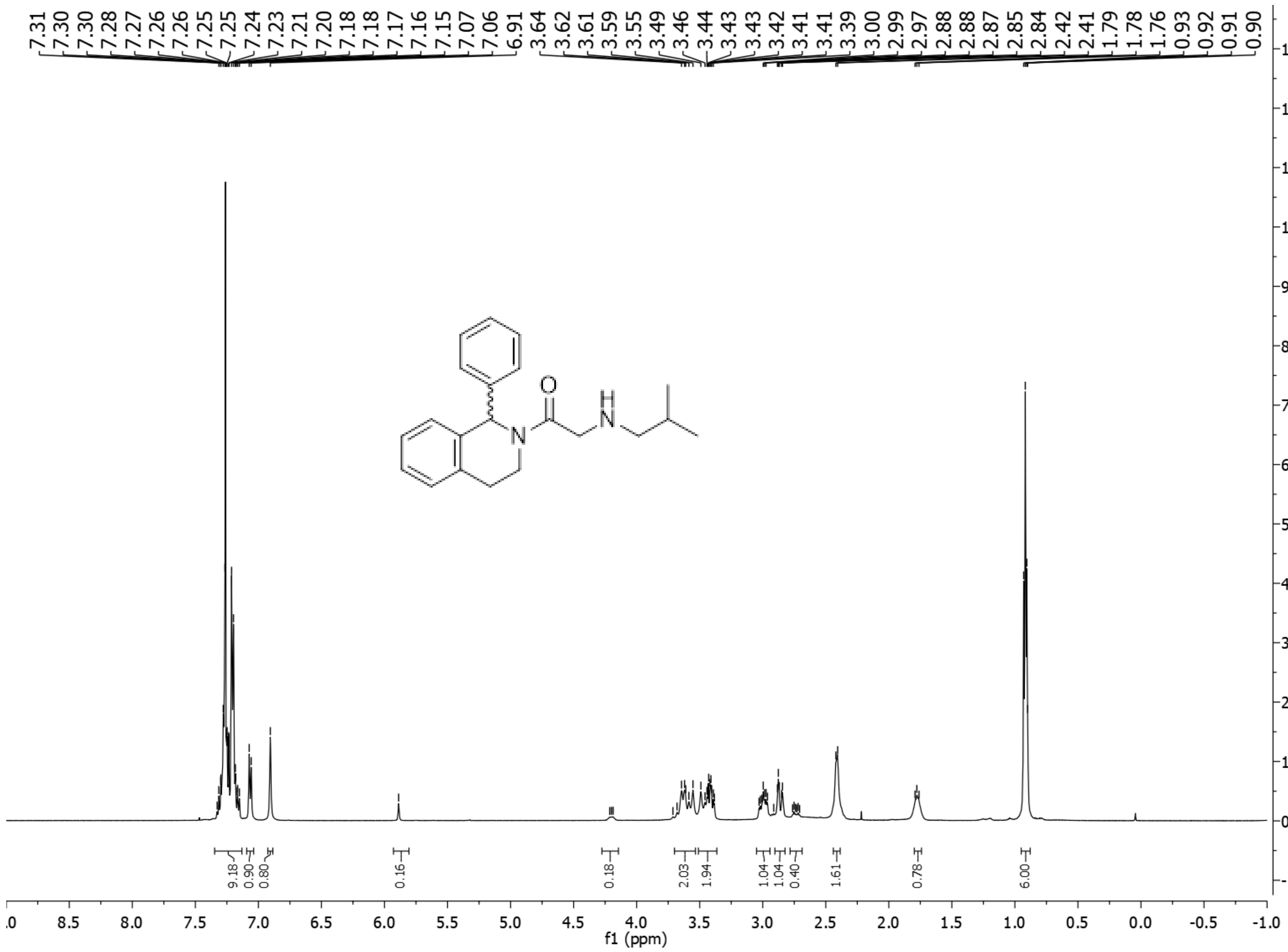

Figure S94. ^{1}H NMR (500 MHz, $CDCl_3$, 218K) of **39**.

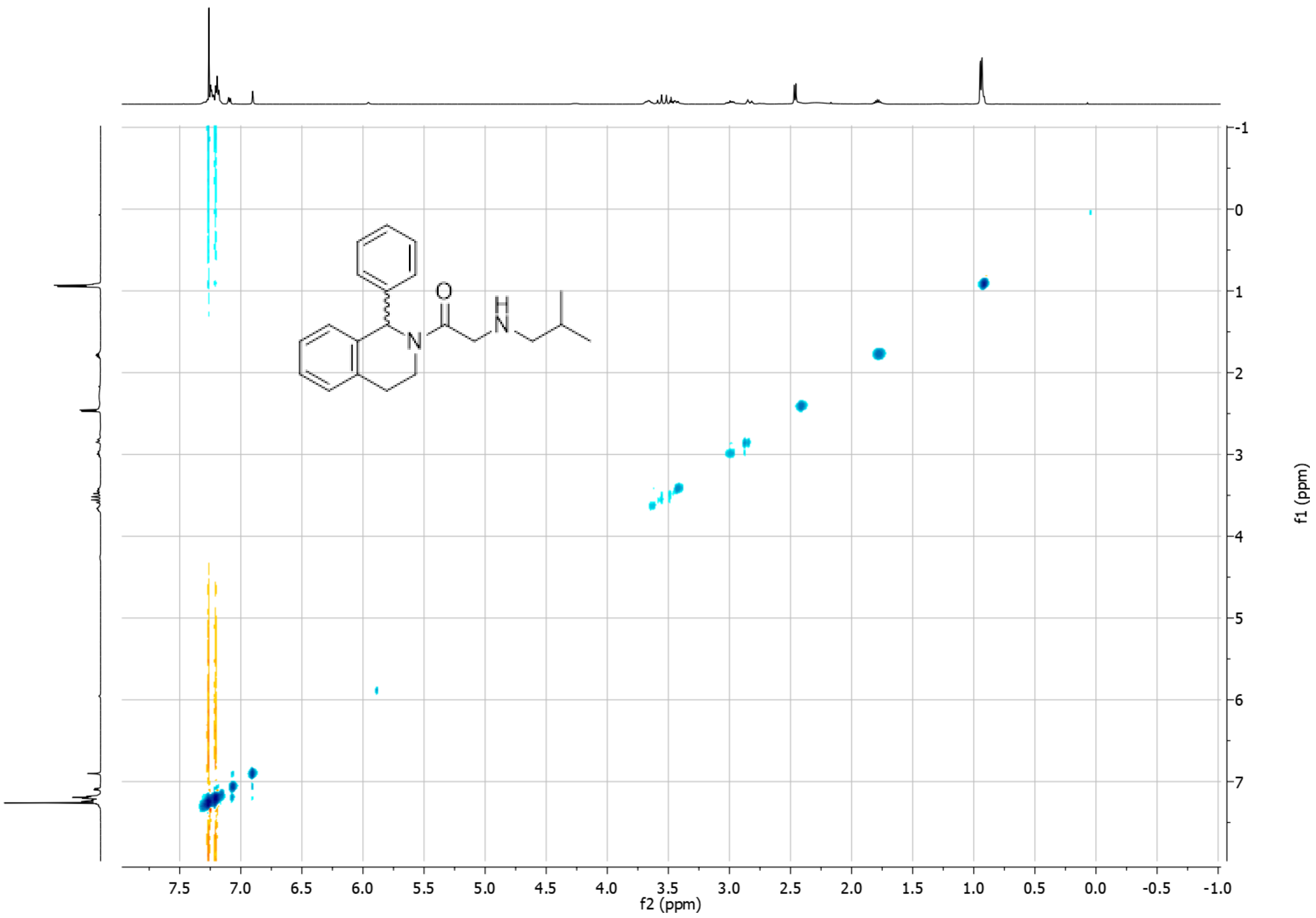

Figure S95. 2D-NOESY (500 MHz, $CDCl_3$, 218K) of **39**.

***N*-isopentyl-1-phenyl-3,4-dihydroisoquinoline-2(1*H*)-carboxamide (40)**

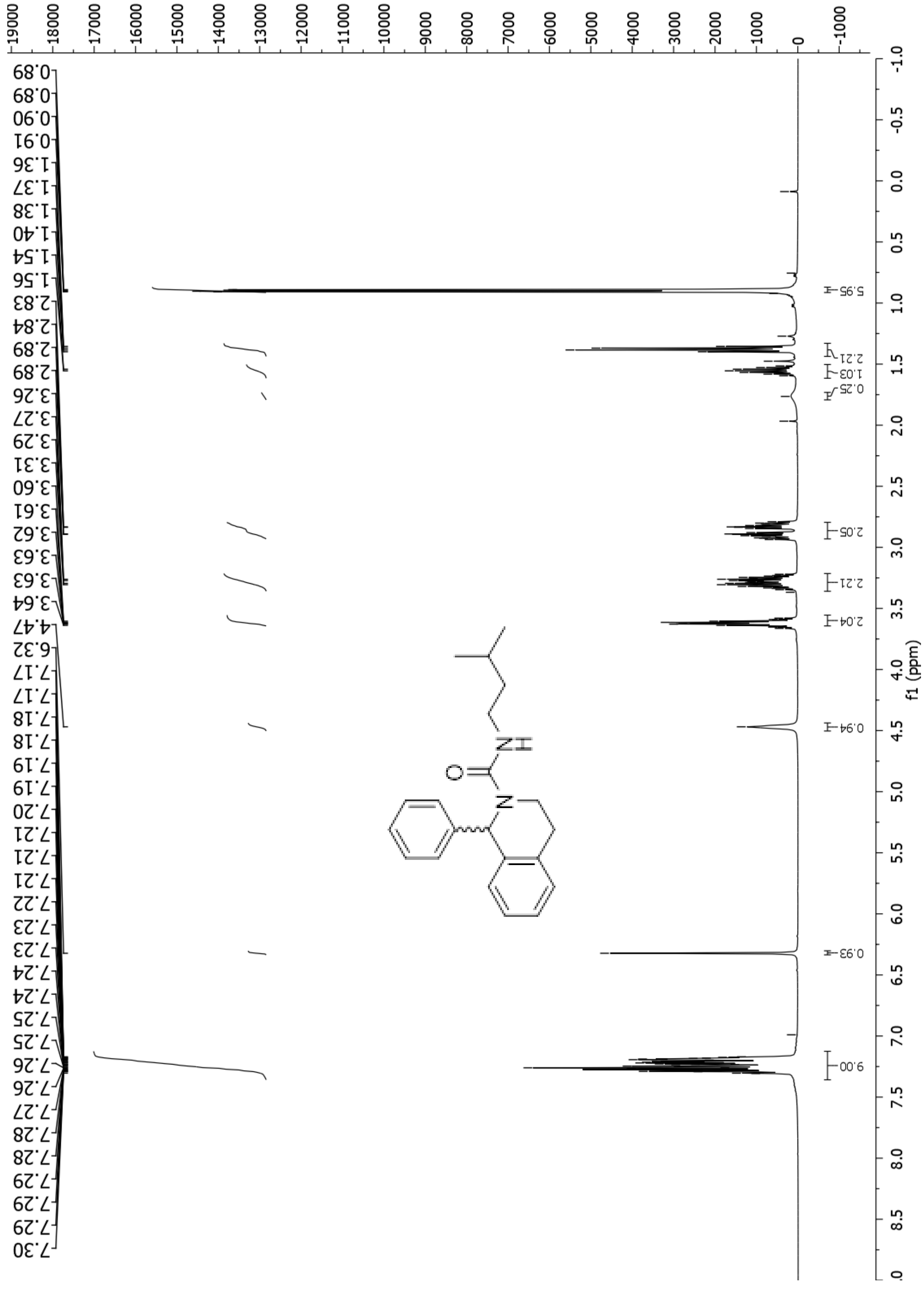

Figure S96. ^{1}H NMR (500 MHz, $CDCl_3$, 298K) of **40**.

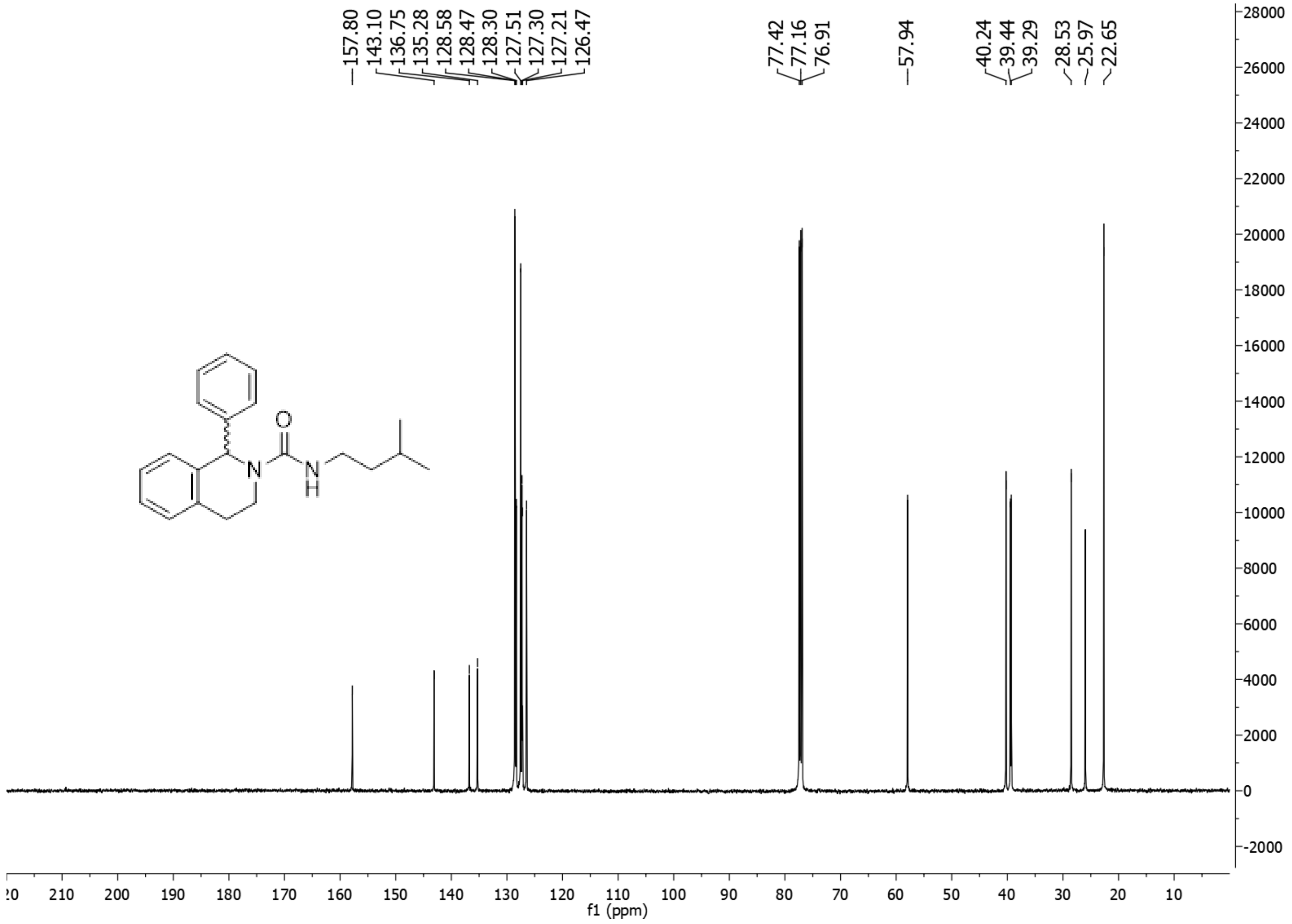

Figure S97. ^{13}C NMR (500 MHz, $CDCl_3$, 298K) of **40**.

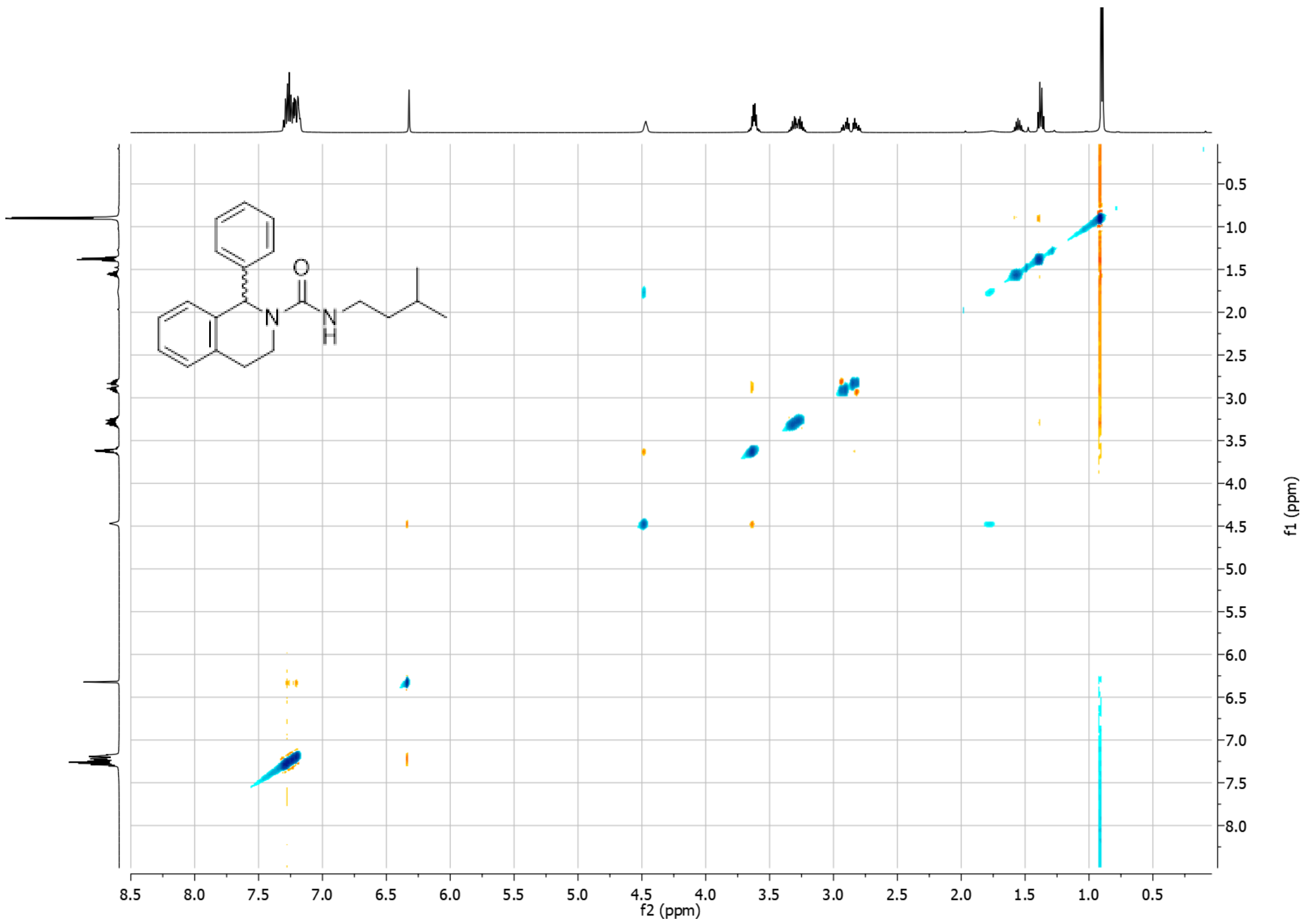

Figure S98. 2D-NOESY (500 MHz, $CDCl_3$, 298K) of **40**.

_N_-(2-methoxyethyl)-1-phenyl-3,4-dihydroisoquinoline-2(1*H*)-carboxamide (41)

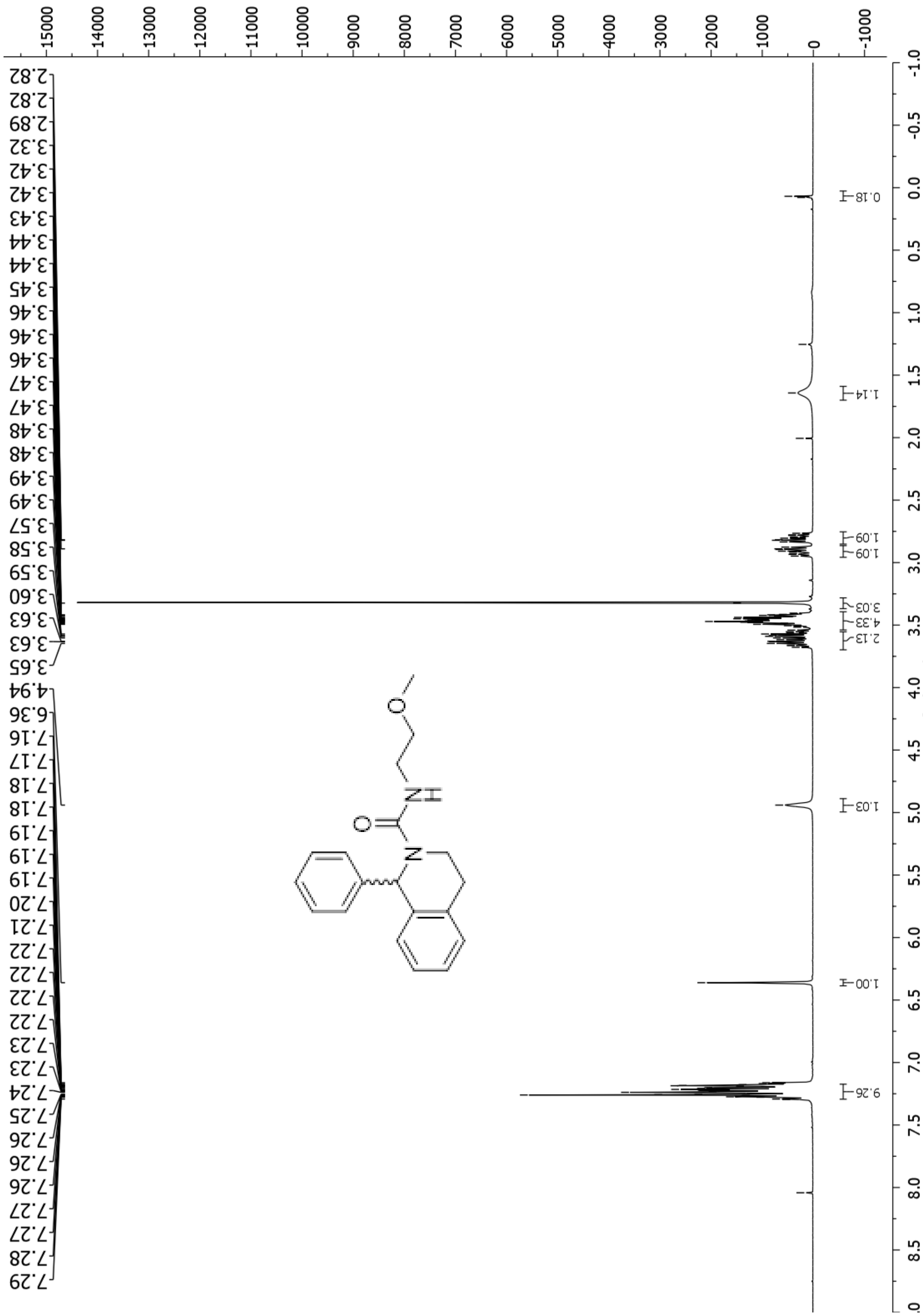

Figure S99. ^{1}H NMR (400 MHz, $CDCl_3$, 298K) of **41**.

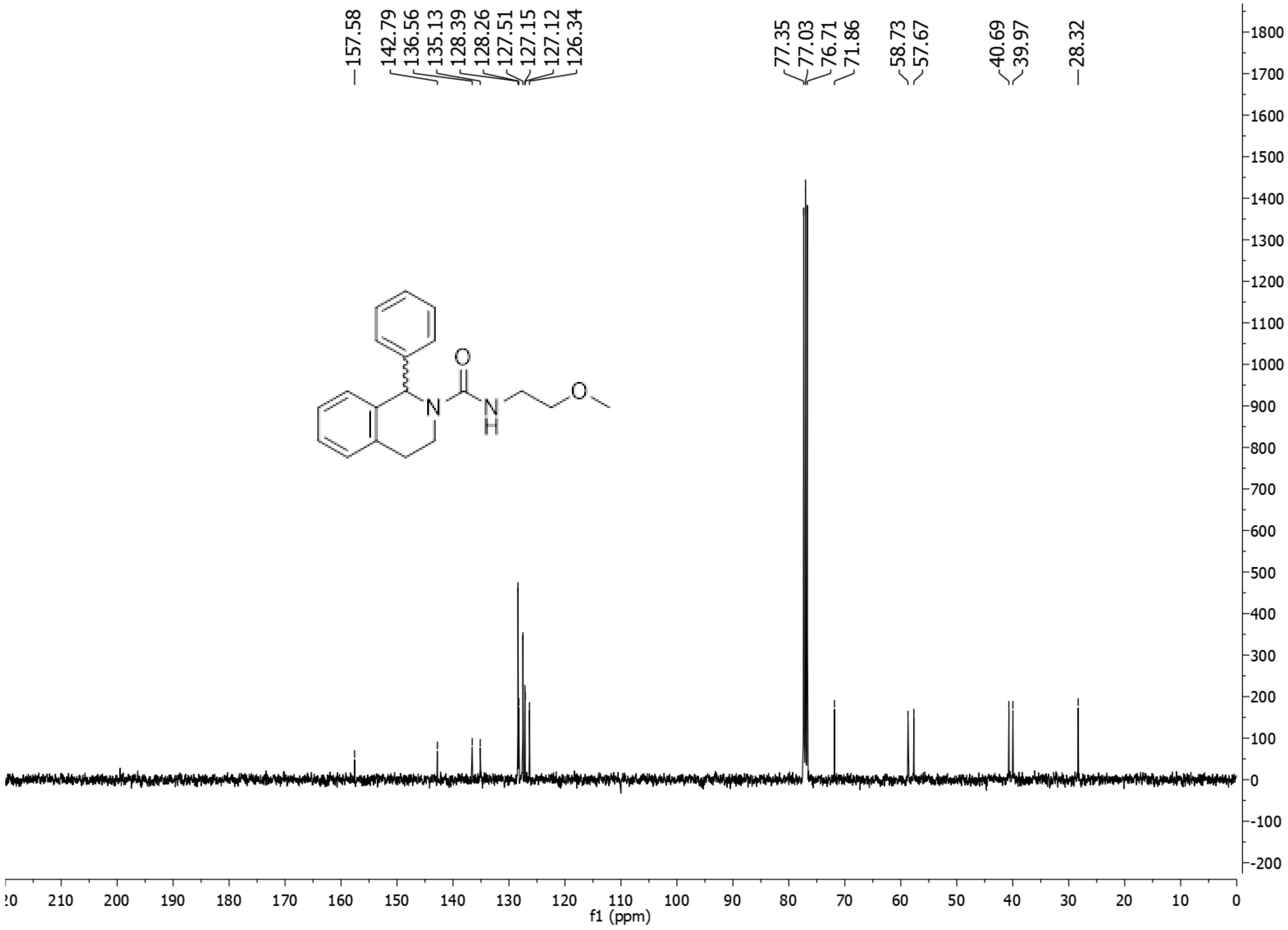

Figure S100. ^{13}C NMR (400 MHz, $CDCl_3$, 298K) of **41**.

(*R*)-2-((2-methylpropyl)amino)-1-(1-phenyl-3,4-dihydroisoquinolin-2(1*H*)-yl)ethan-1-one (42)

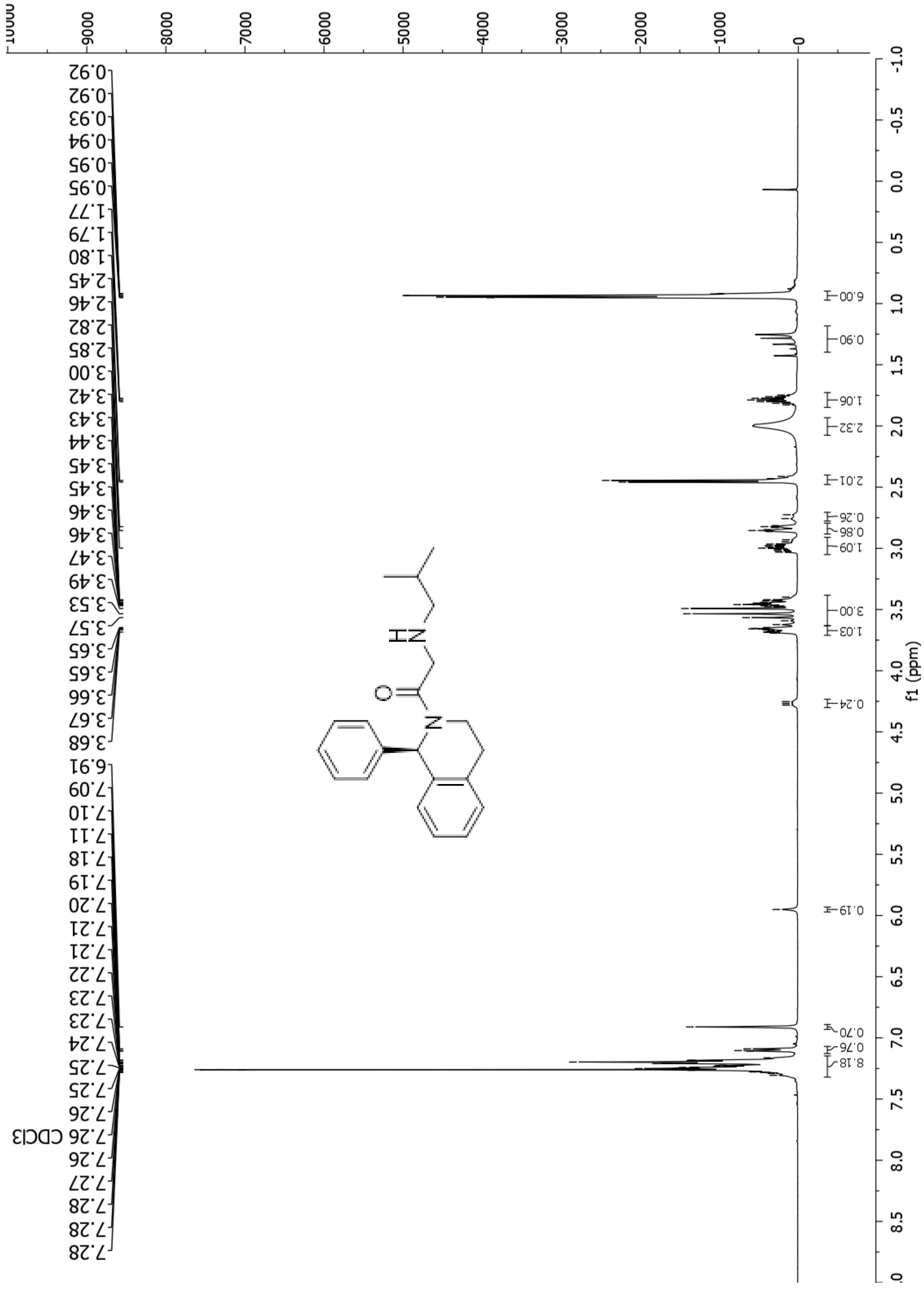

Figure S101. ^{1}H NMR (500 MHz, $CDCl_3$, 298K) of **42**.

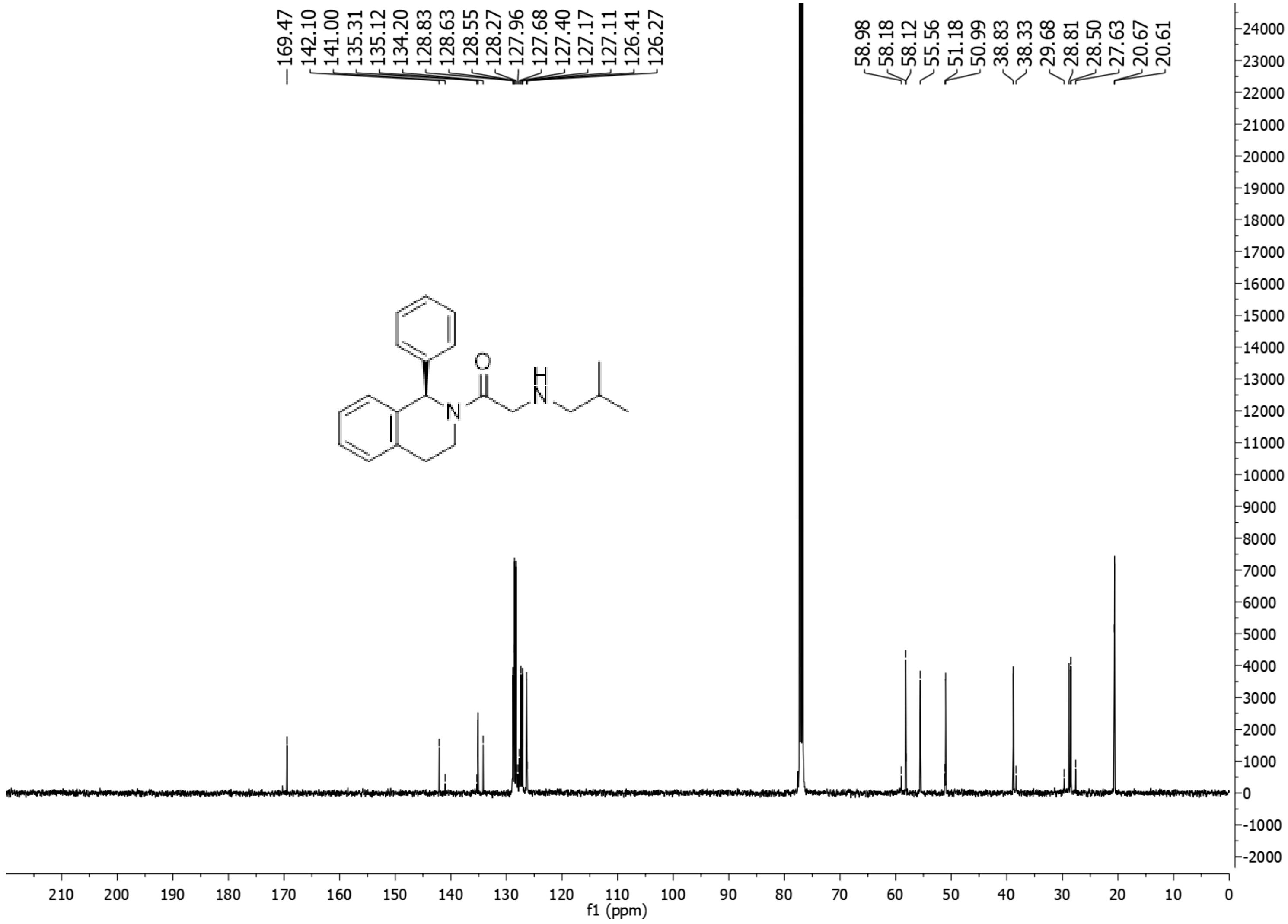

Figure S102. ^{13}C NMR (500 MHz, $CDCl_3$, 298K) of **42**.

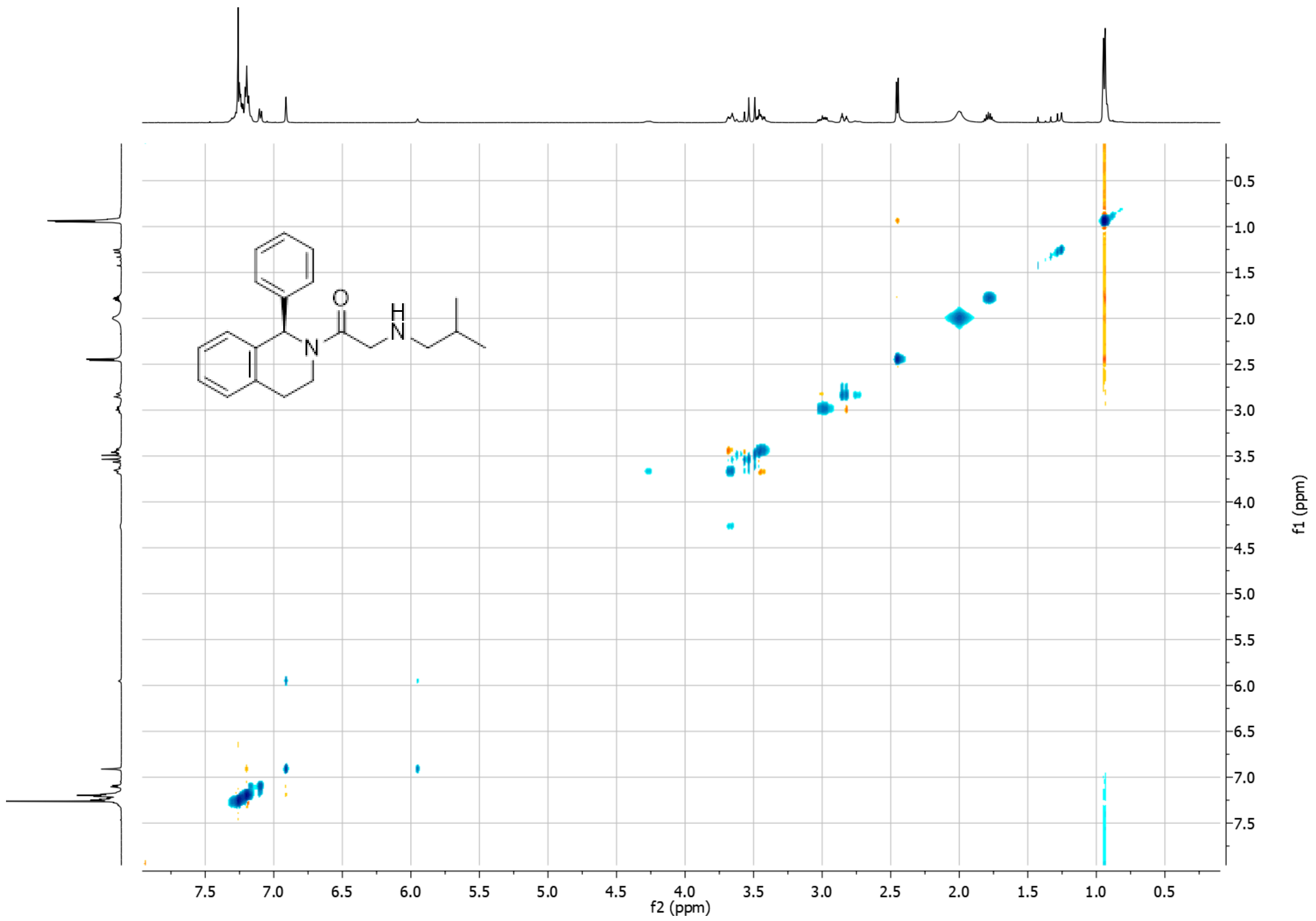

Figure S103. 2D-NOESY (500 MHz, $CDCl_3$, 298K) of **42**.

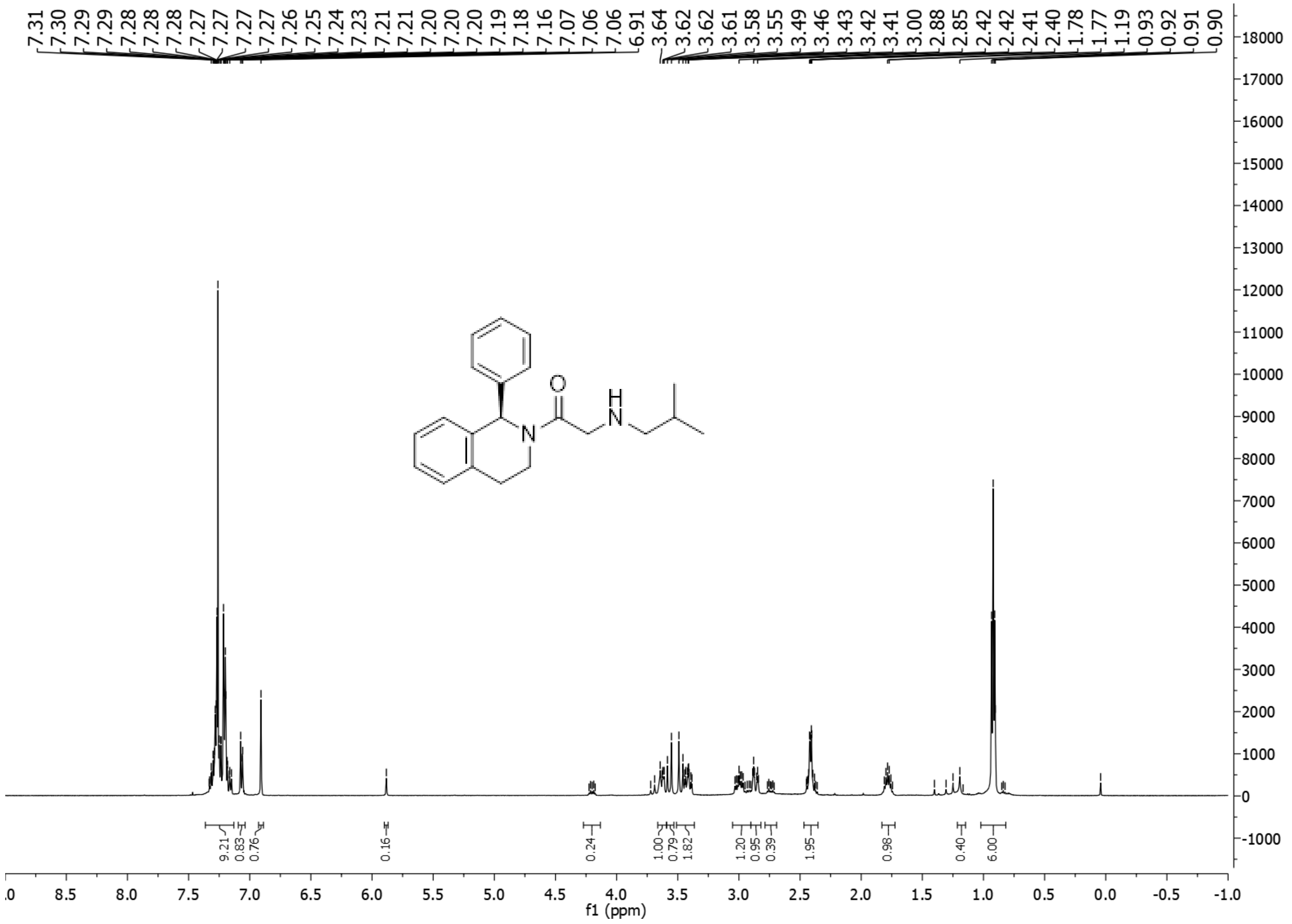

Figure S104. ^{1}H NMR (500 MHz, $CDCl_3$, 218K) of **42**.

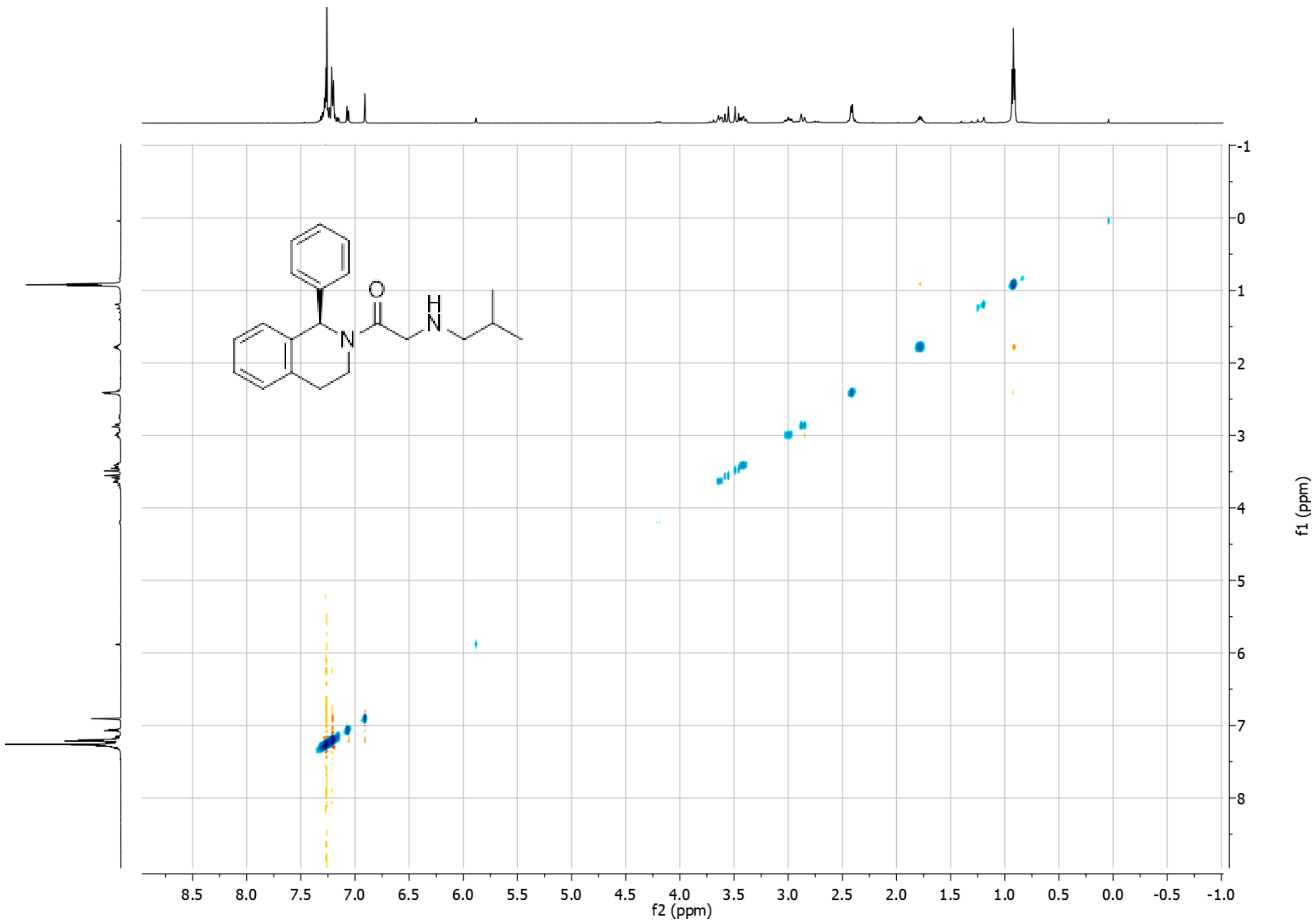

Figure S105. 2D-NOESY (500 MHz, $CDCl_3$, 218K) of **42**.

(*S*)-2-((2-methylpropyl)amino)-1-(1-phenyl-3,4-dihydroisoquinolin-2(1H)-yl)ethan-1-one (43)

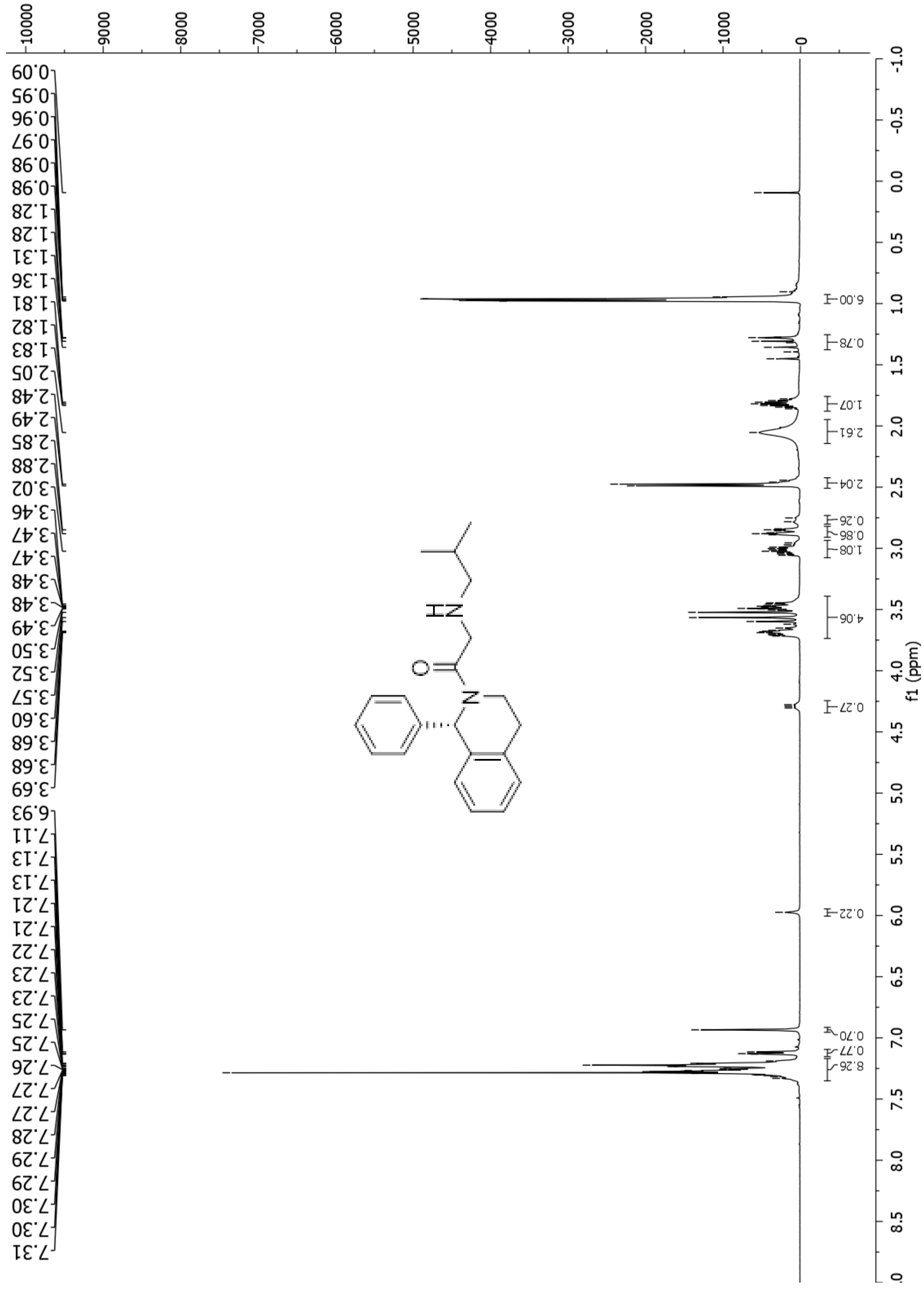

Figure S106. ^{1}H NMR (500 MHz, $CDCl_3$, 298K) of **43**.

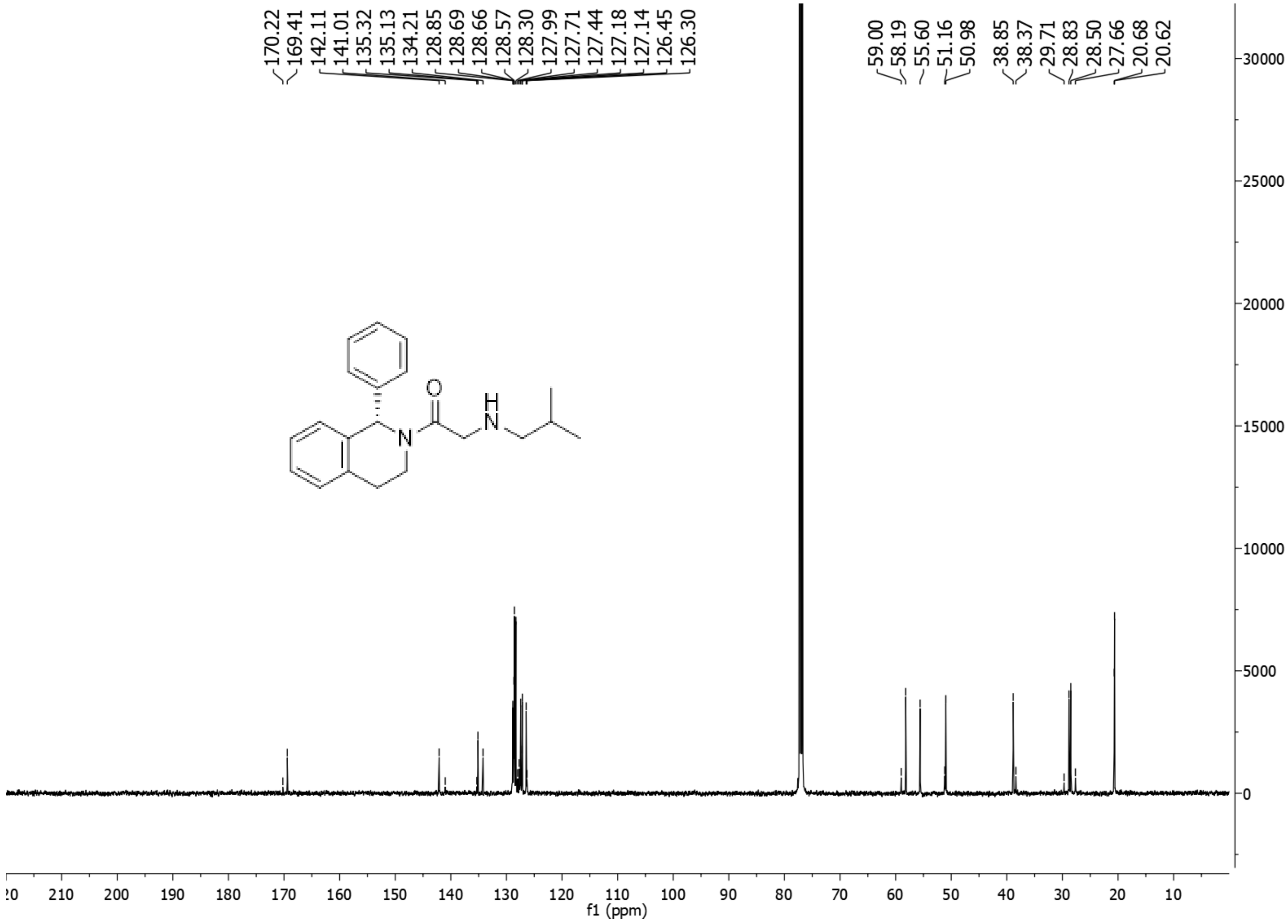

Figure S107. ^{13}C NMR (500 MHz, $CDCl_3$, 298K) of **43**.

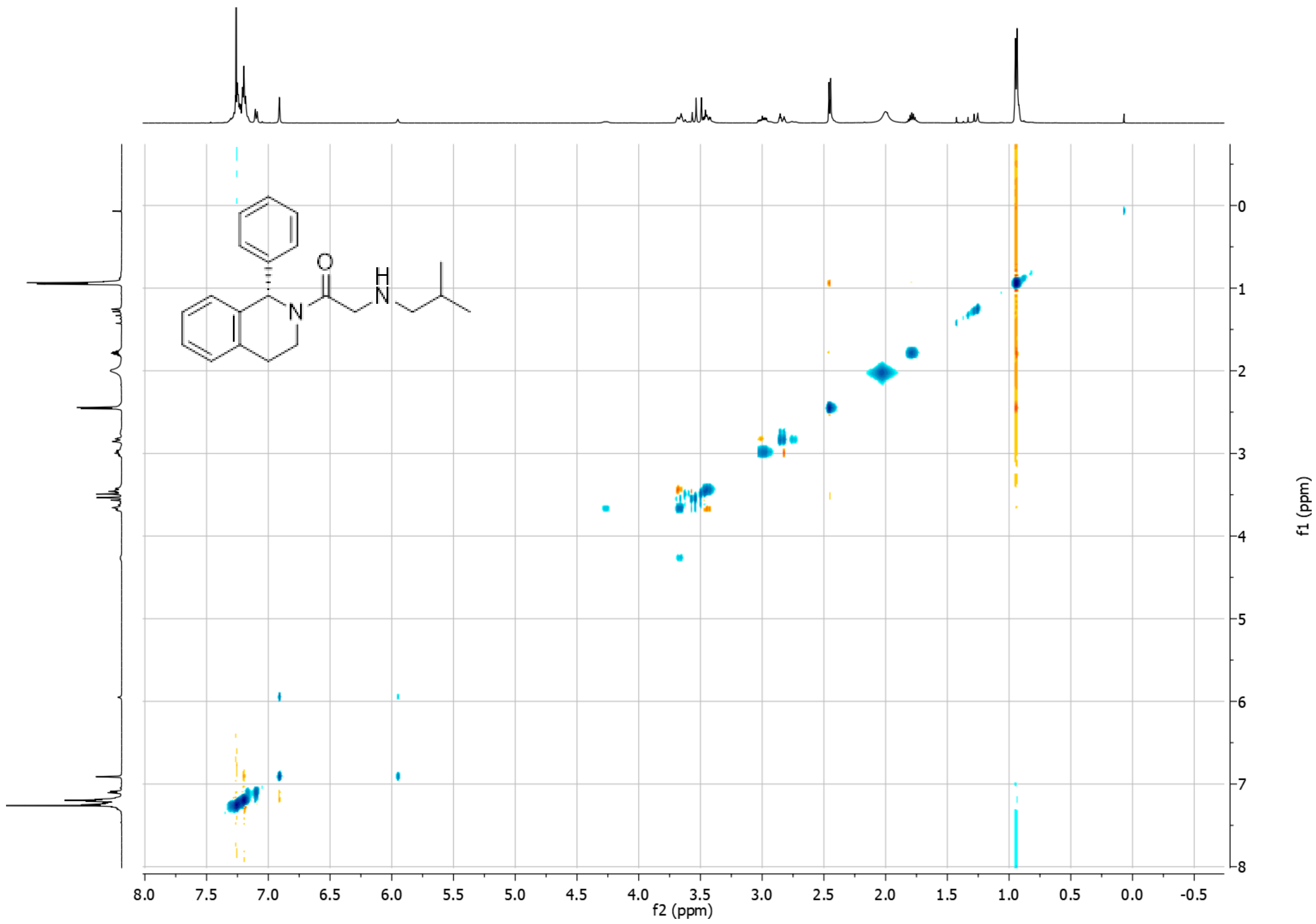

Figure S108. 2D-NOESY (500 MHz, $CDCl_3$, 298K) of **43**.

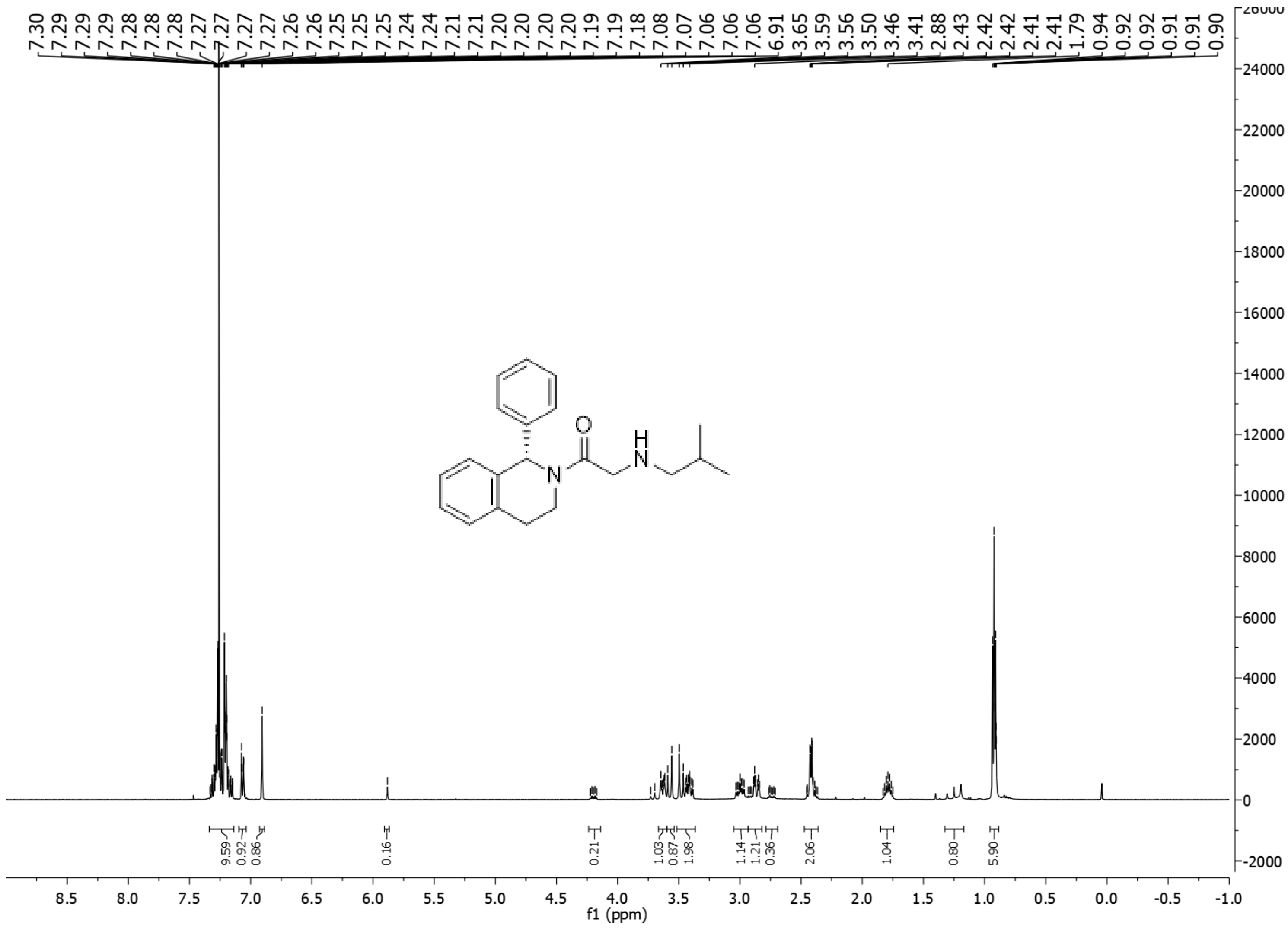

Figure S109. ^{1}H NMR (500 MHz, $CDCl_3$, 218K) of **43**.

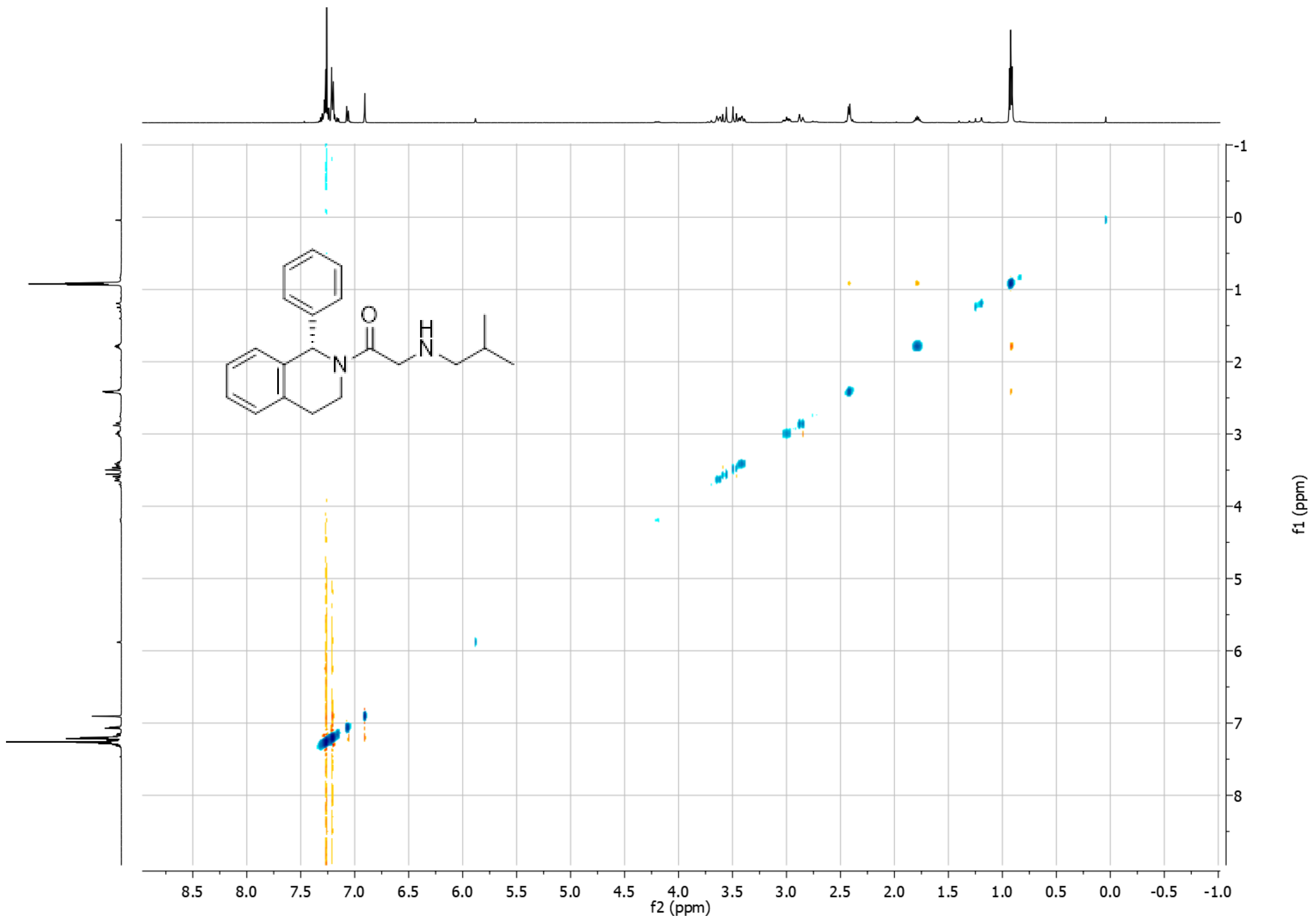

Figure S110. 2D-NOESY (500 MHz, $CDCl_3$, 218K) of **43**.

1-(6,7-dimethoxy-1-phenyl-3,4-dihydroisoquinolin-2(1*H*)-yl)-2-((2-methylbutyl)amino)ethan-1-one (44)

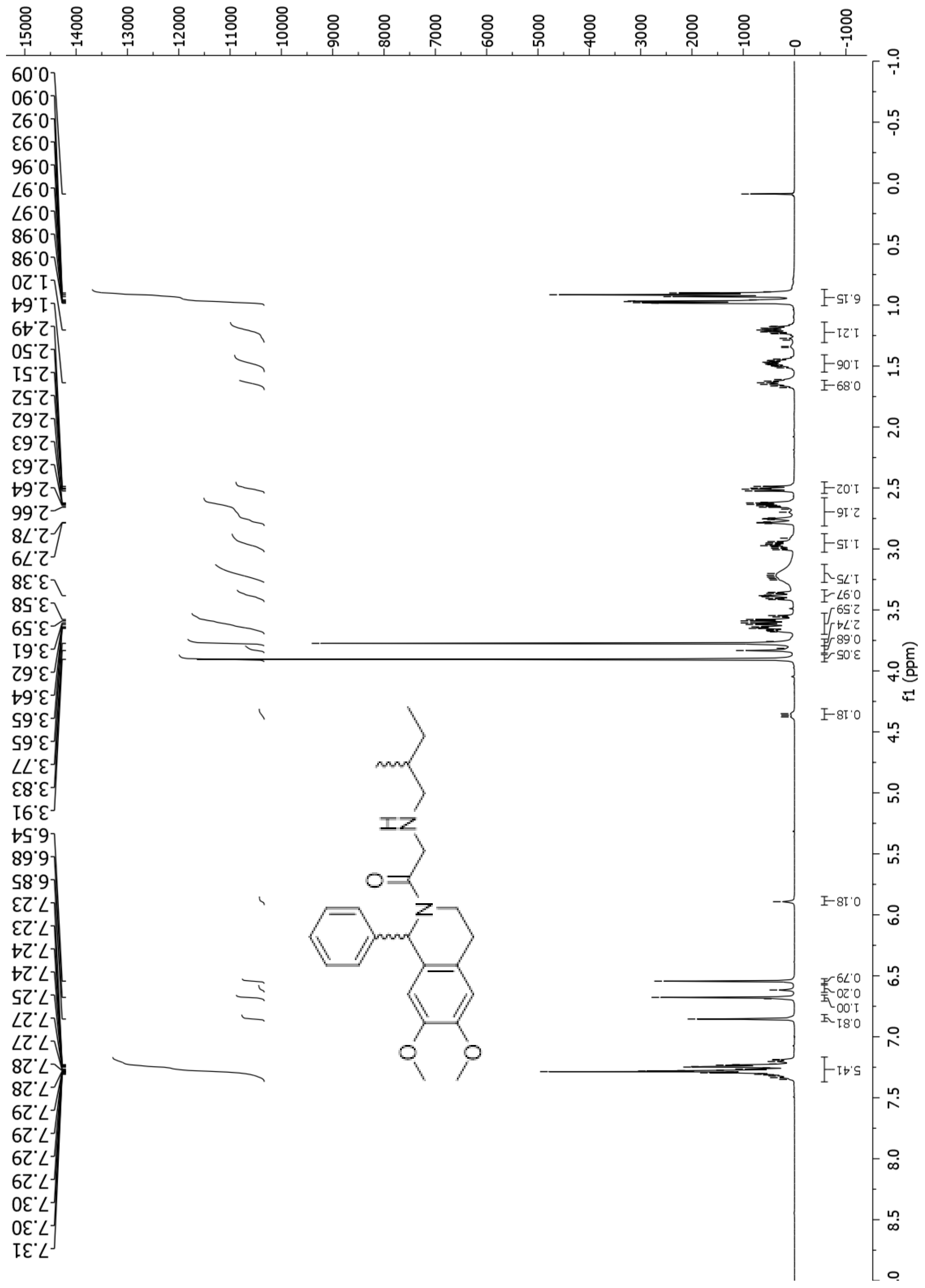

Figure S111. ^{1}H NMR (500 MHz, $CDCl_3$, 298K) of **44**.

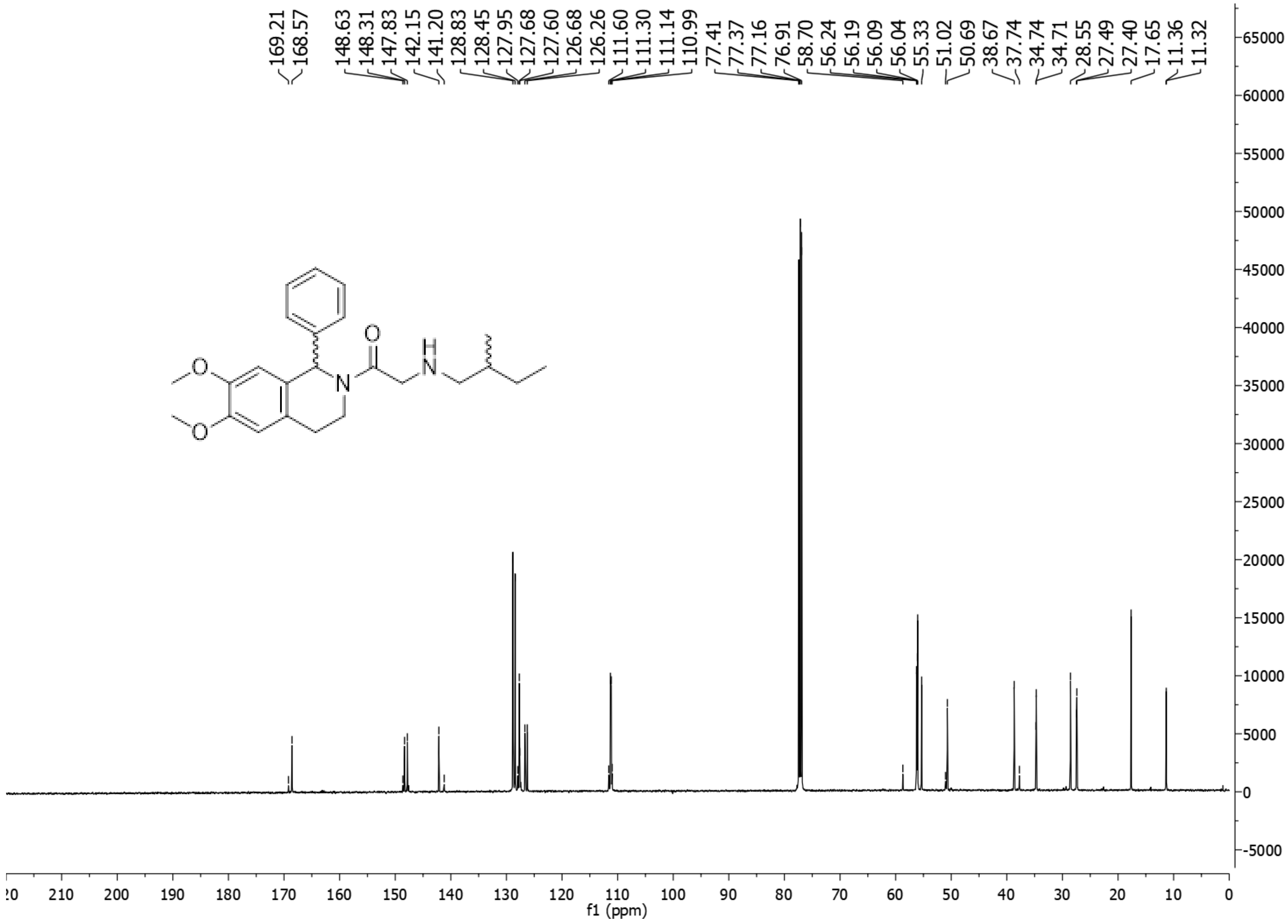

Figure S112. ^{13}C NMR (500 MHz, $CDCl_3$, 298K) of **44**.

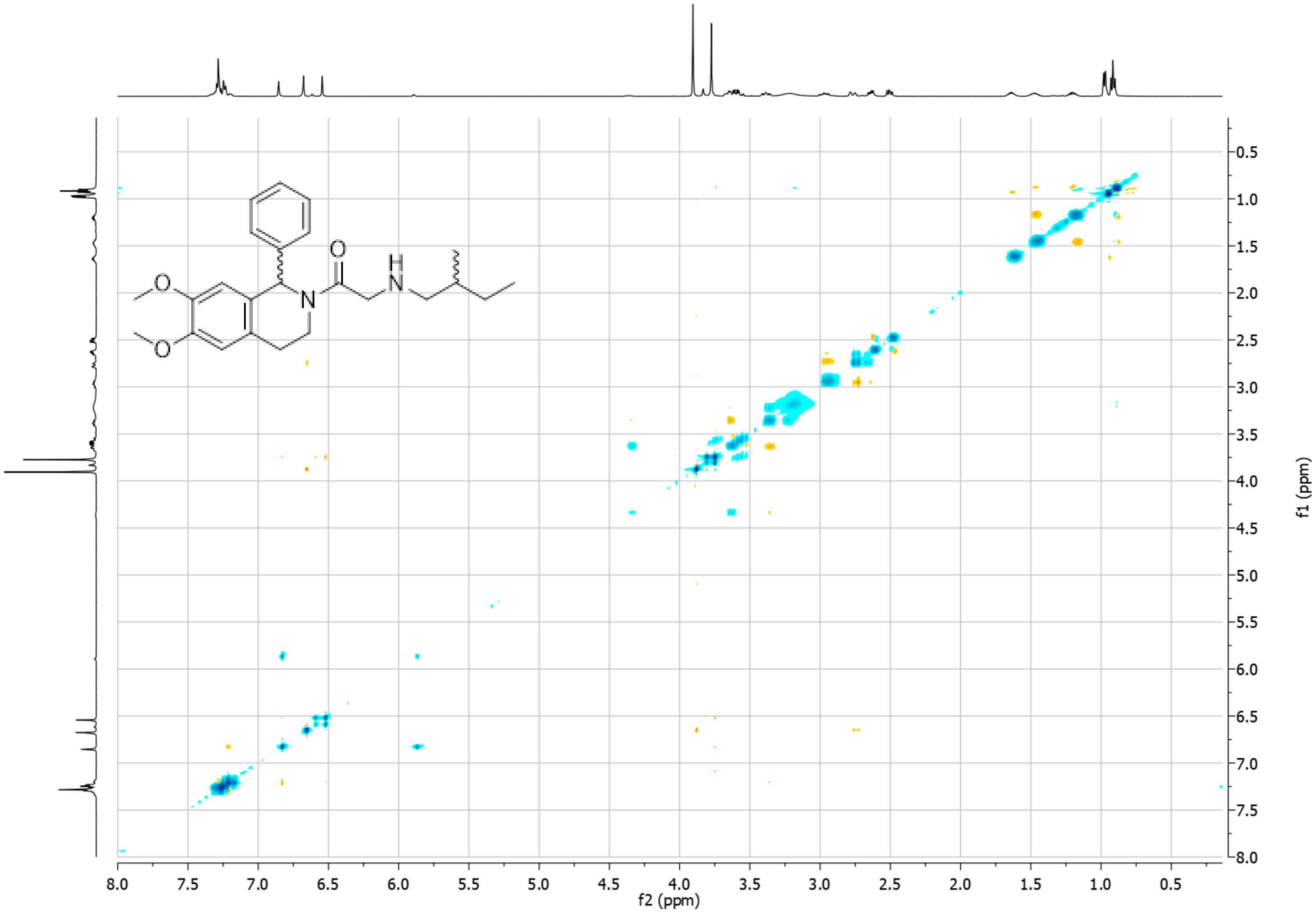

Figure S113. 2D-NOESY (500 MHz, $CDCl_3$, 298K) of **44**.

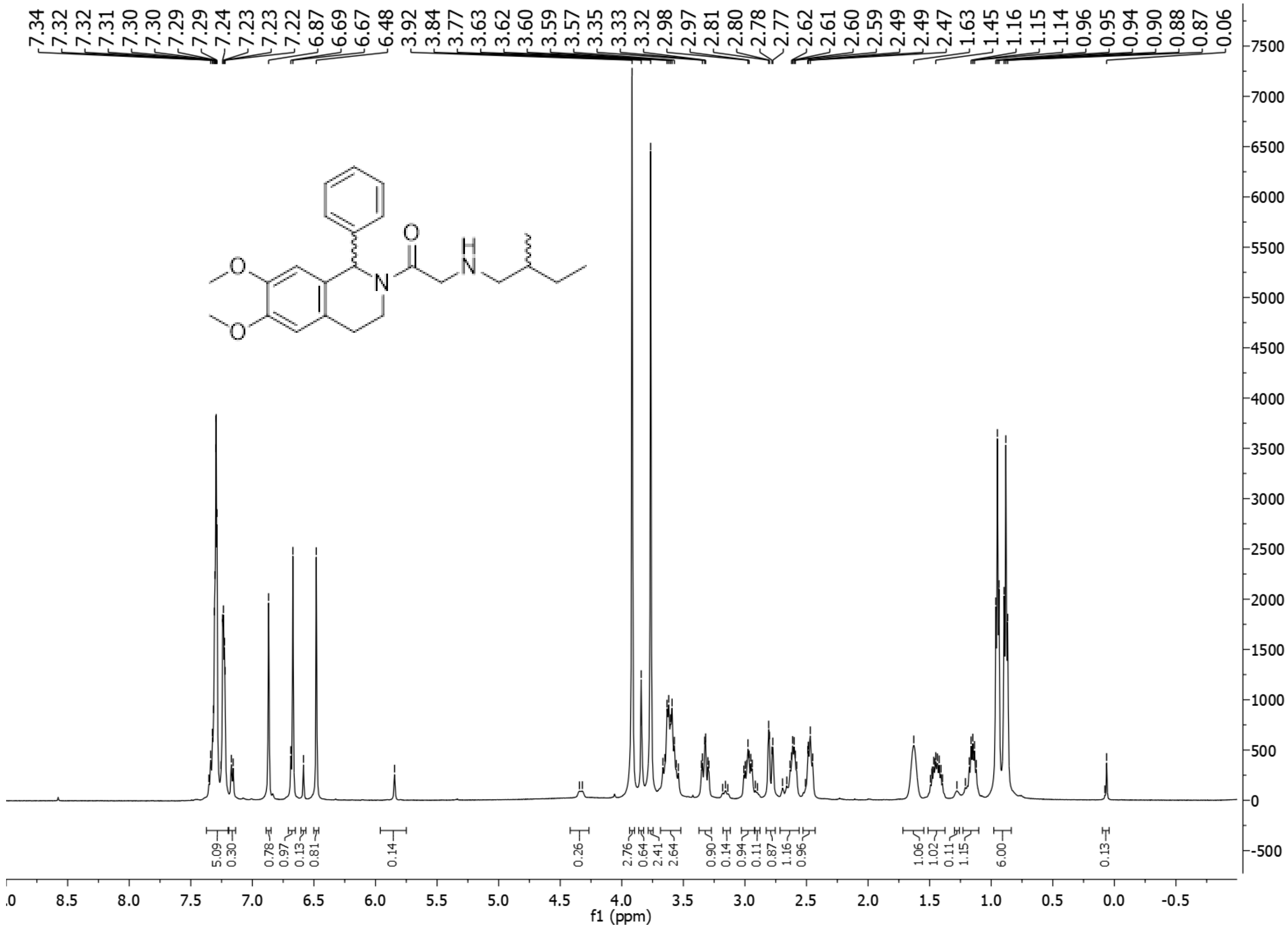

Figure S114. ^{1}H NMR (500 MHz, $CDCl_3$, 218K) of **44**.

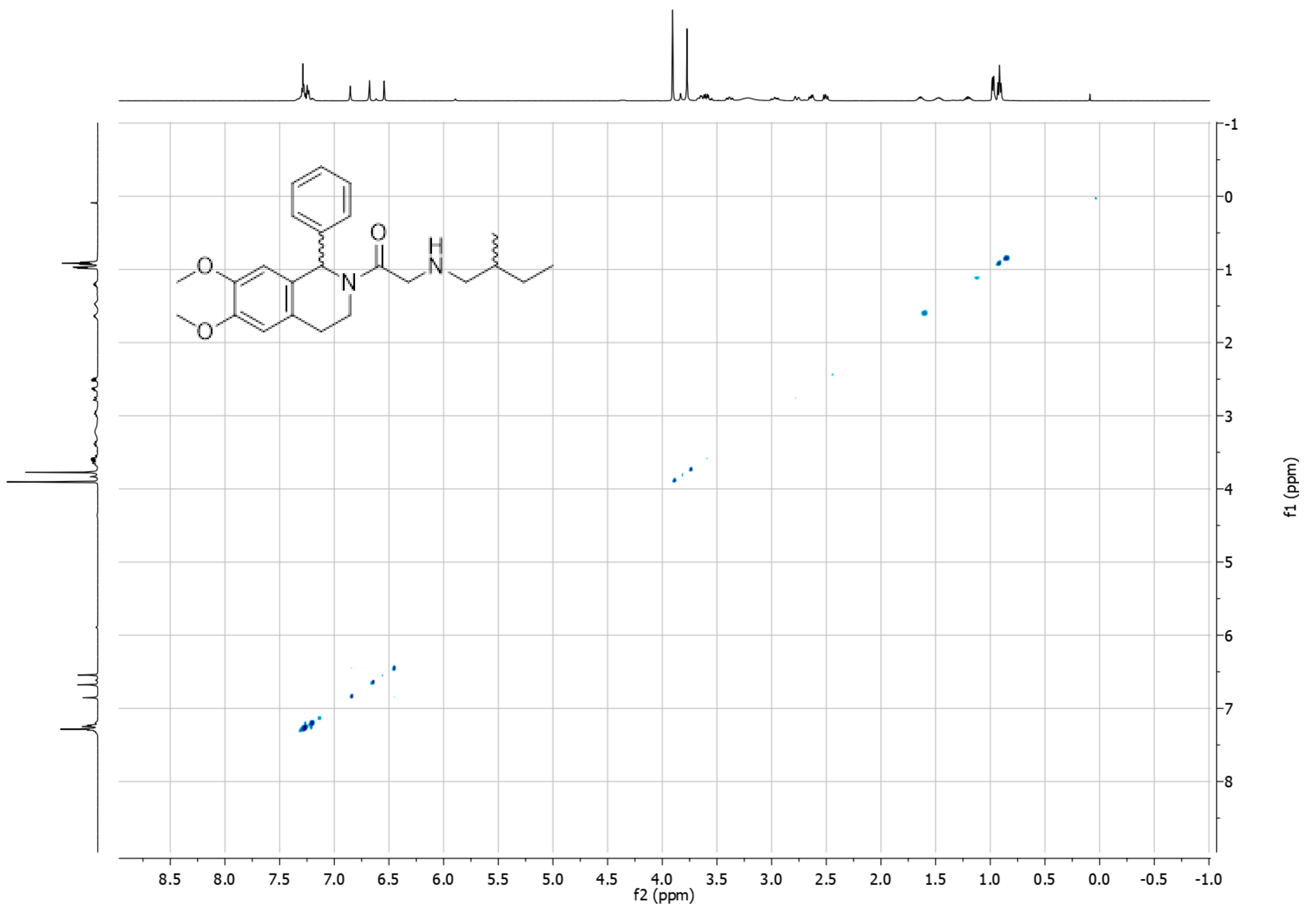

Figure S115. 2D-NOESY (500 MHz, $CDCl_3$, 218K) of **44**.

1-(7-chloro-1-phenyl-3,4-dihydroisoquinolin-2(1*H*)-yl)-2-((2-methylbutyl)amino)ethan-1-one (45)

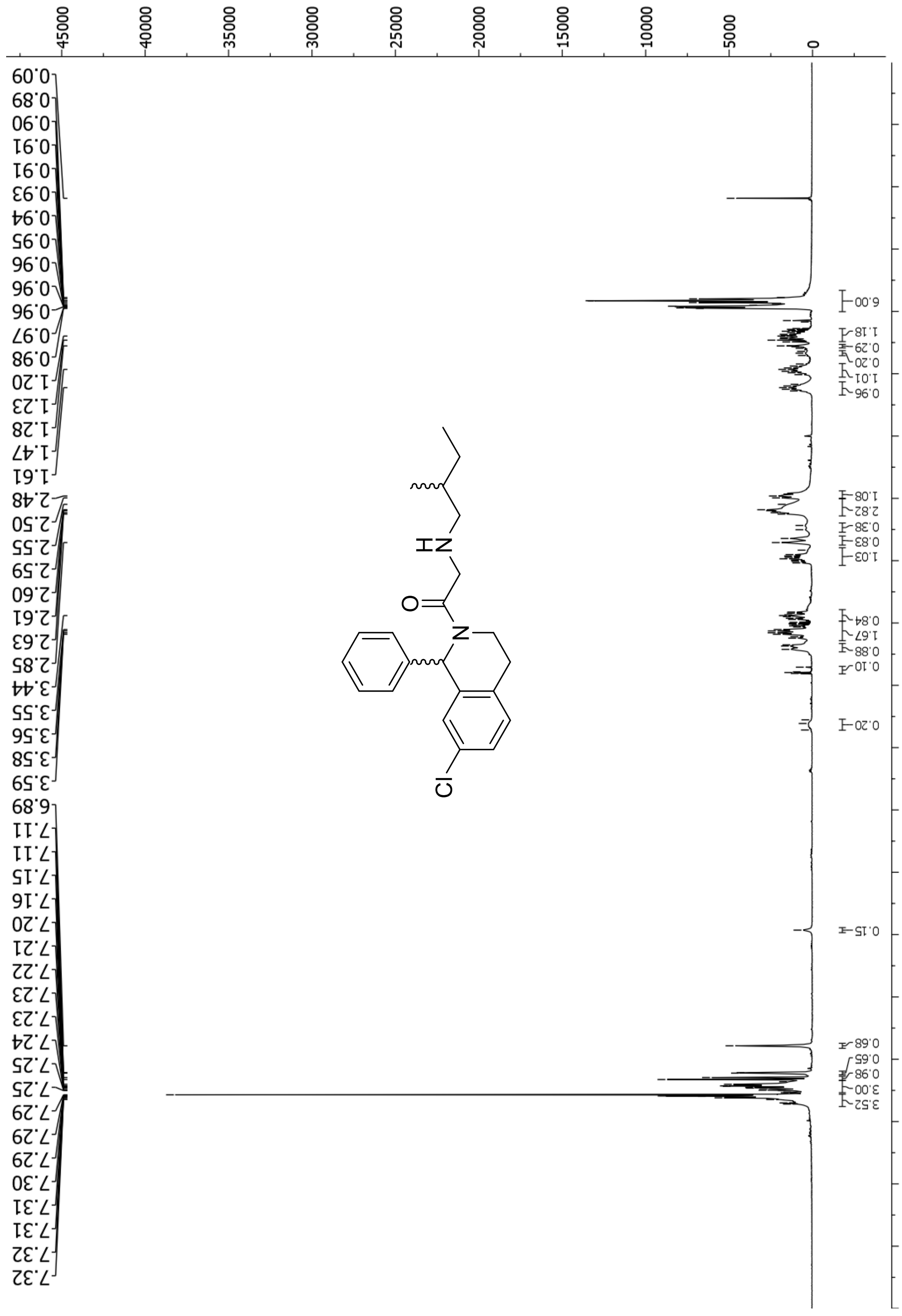

Figure S116. ^{1}H NMR (500 MHz, $CDCl_3$, 298K) of **45**.

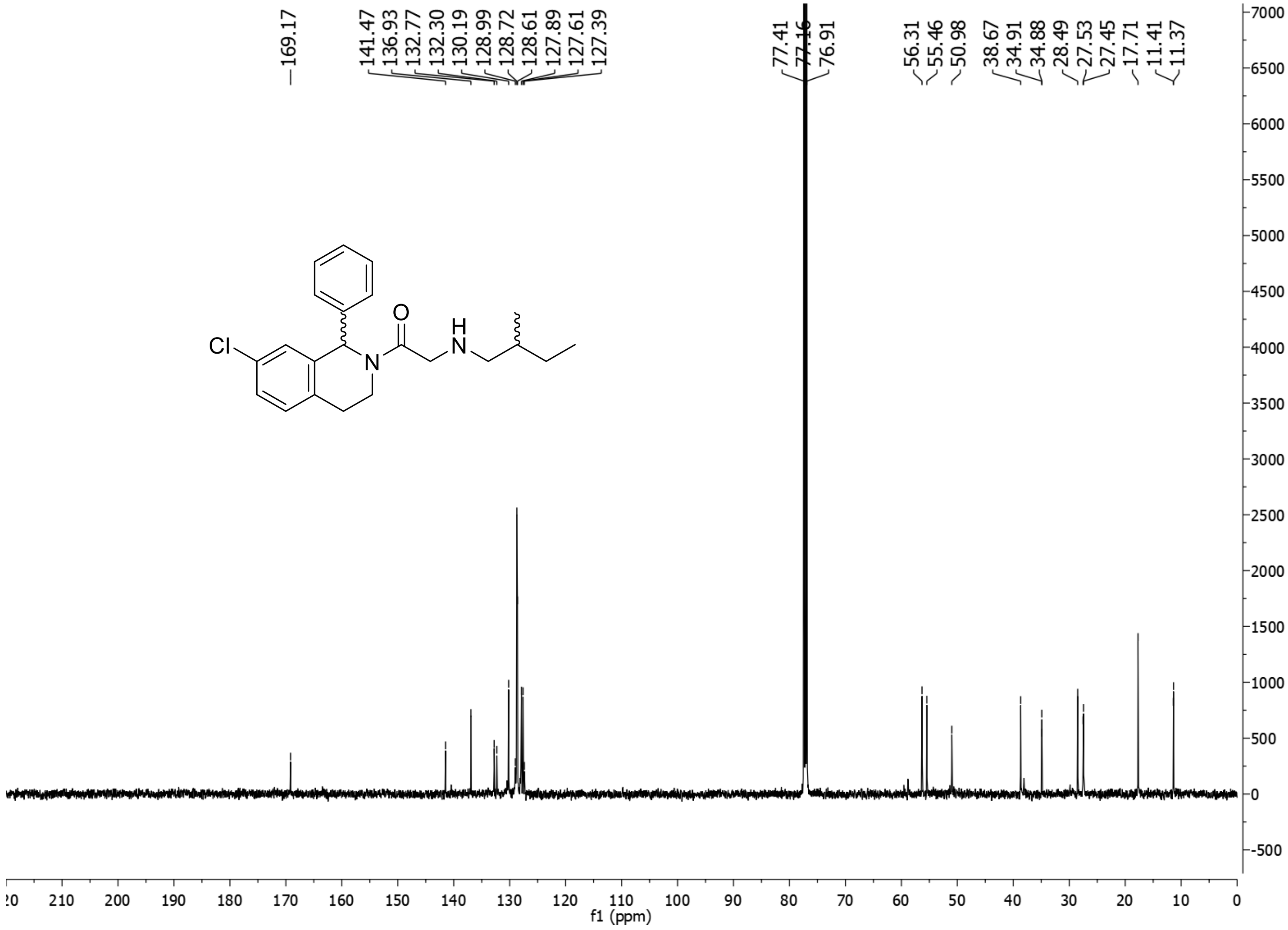

Figure S117. ^{13}C NMR (500 MHz, $CDCl_3$, 298K) of **45**.

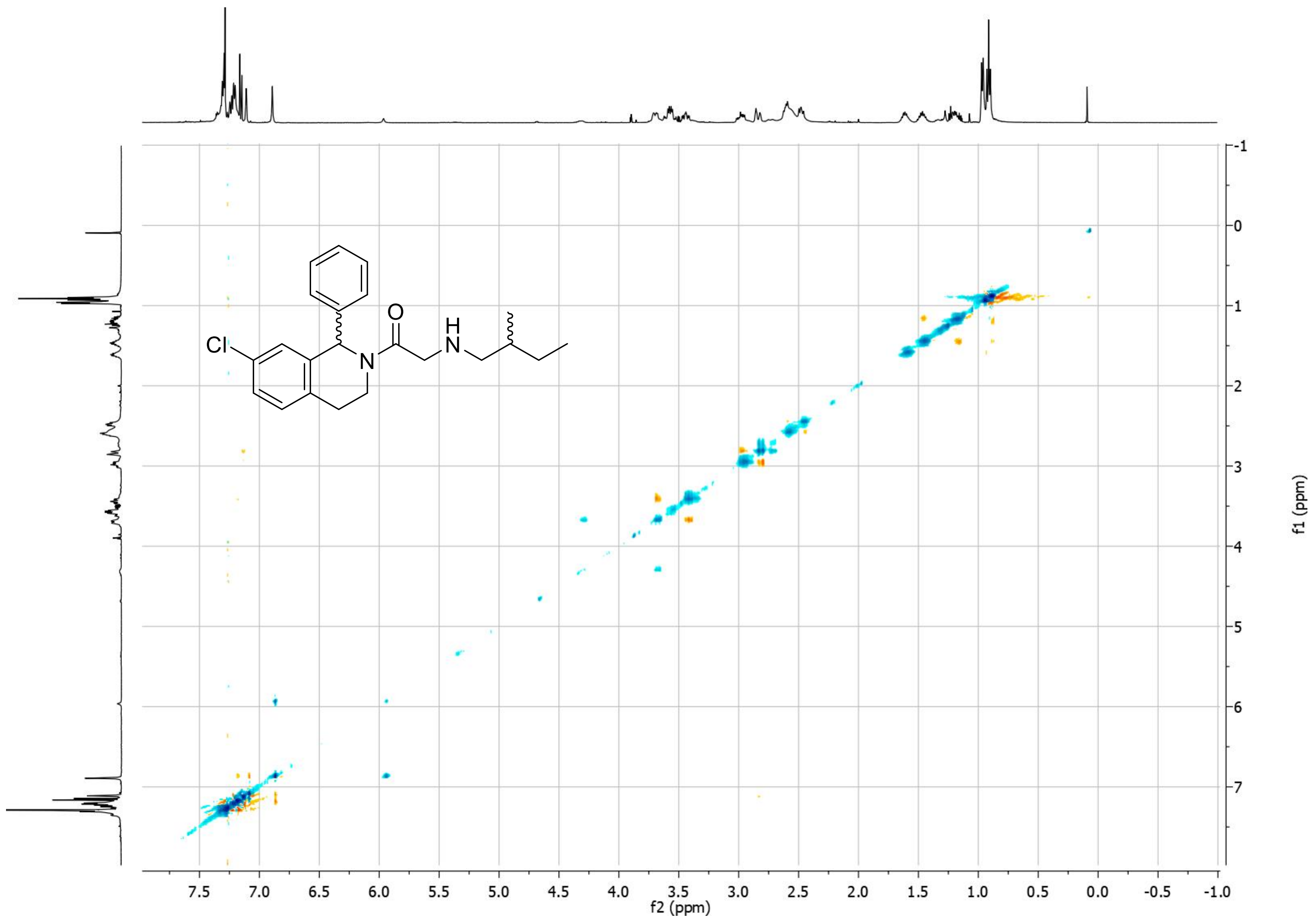

Figure S118. 2D-NOESY (500 MHz, $CDCl_3$, 298K) of **45**.

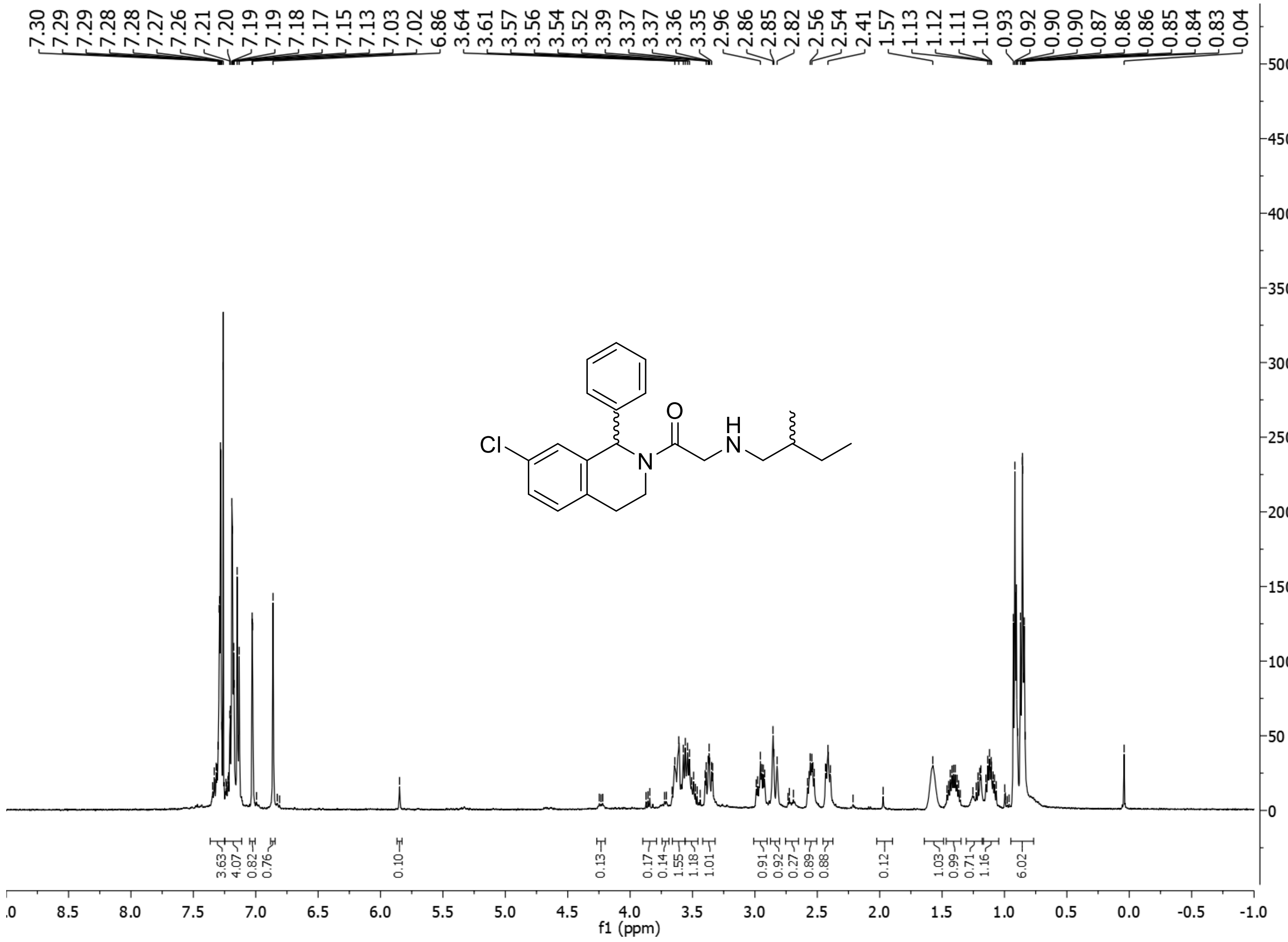

Figure S119. ^{1}H NMR (500 MHz, $CDCl_3$, 218K) of **45**.

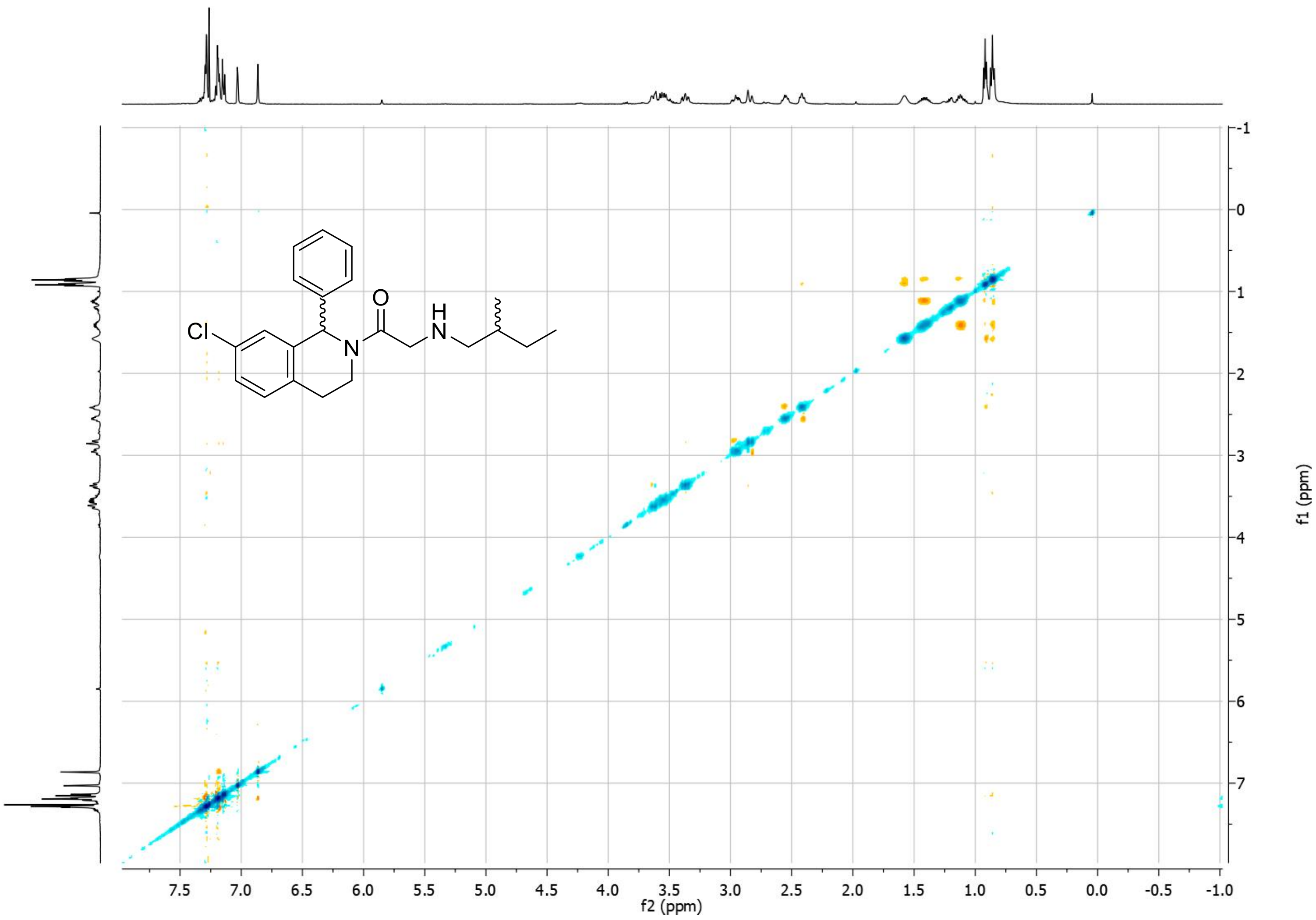

Figure S120. 2D-NOESY (500 MHz, $CDCl_3$, 218K) of **45**.

1-(1-phenyl-3,4-dihydropyrrolo[1,2-a]pyrazin-2(1H)-yl)prop-2-en-1-one (46)

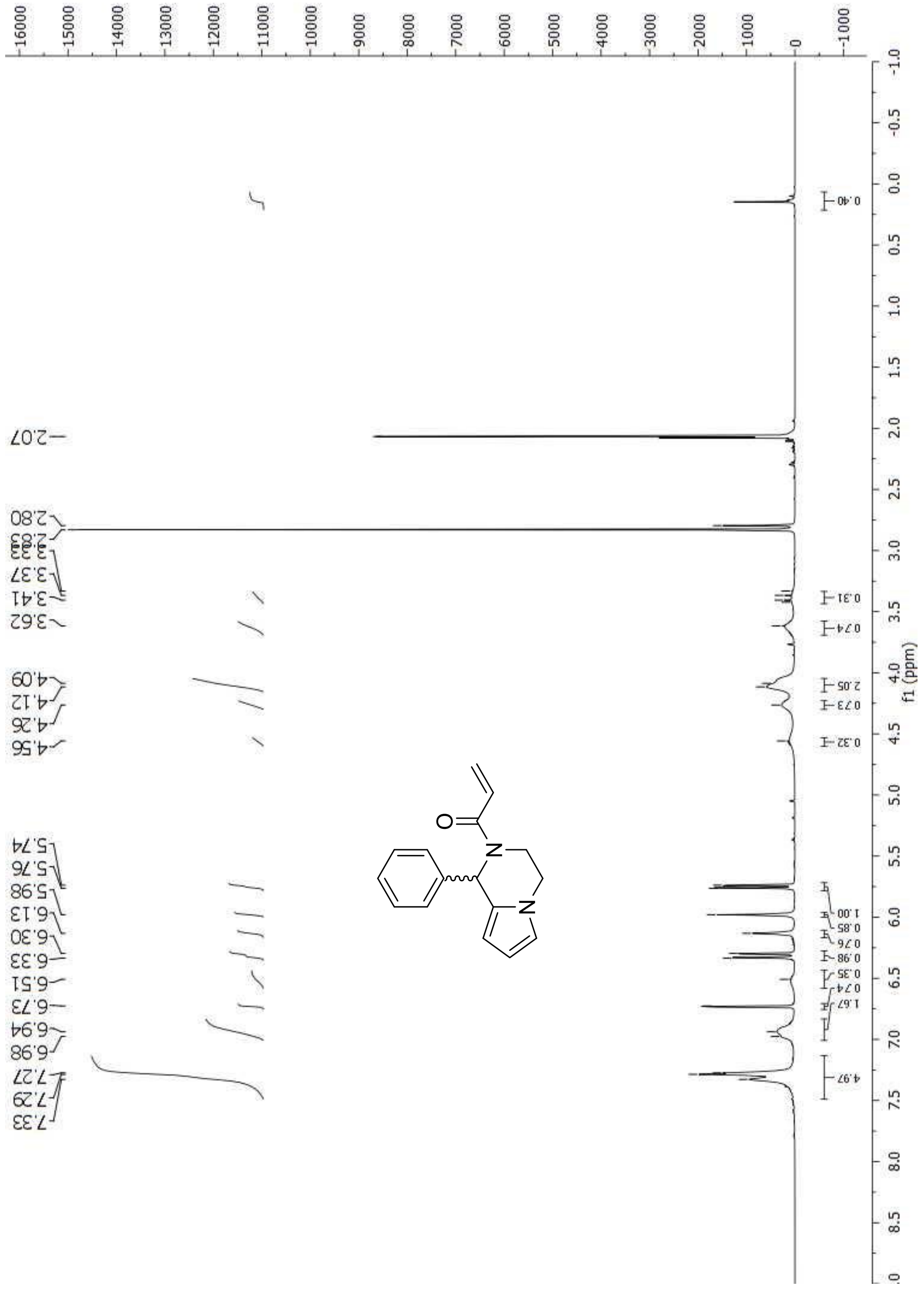

Figure S121. ^{1}H NMR (500 MHz, $(CD_3)_2CO_3$, 298K) of **46**.

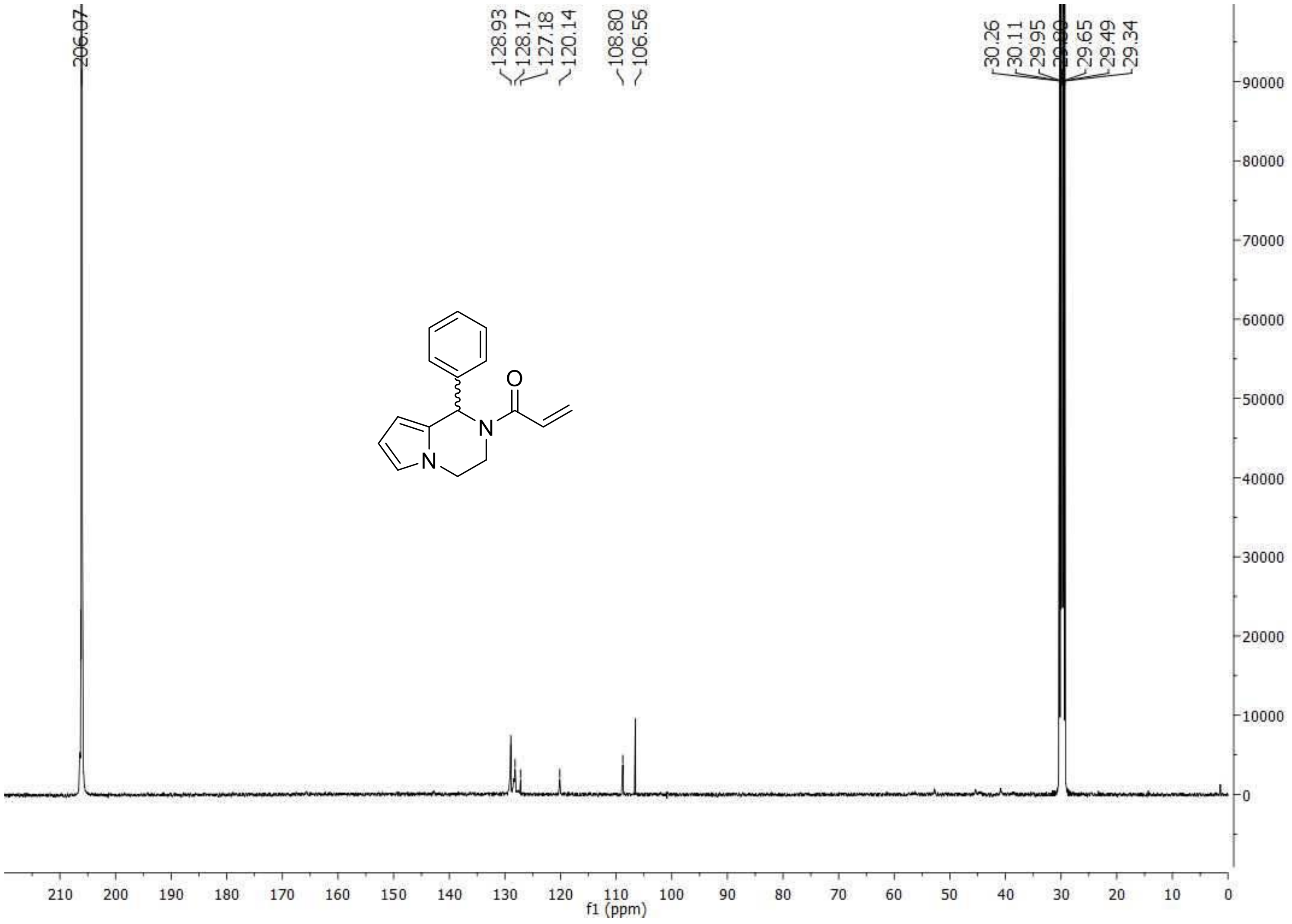

Figure S122. ^{13}C NMR (500 MHz, $CDCl_3$, 298K) of **46**.

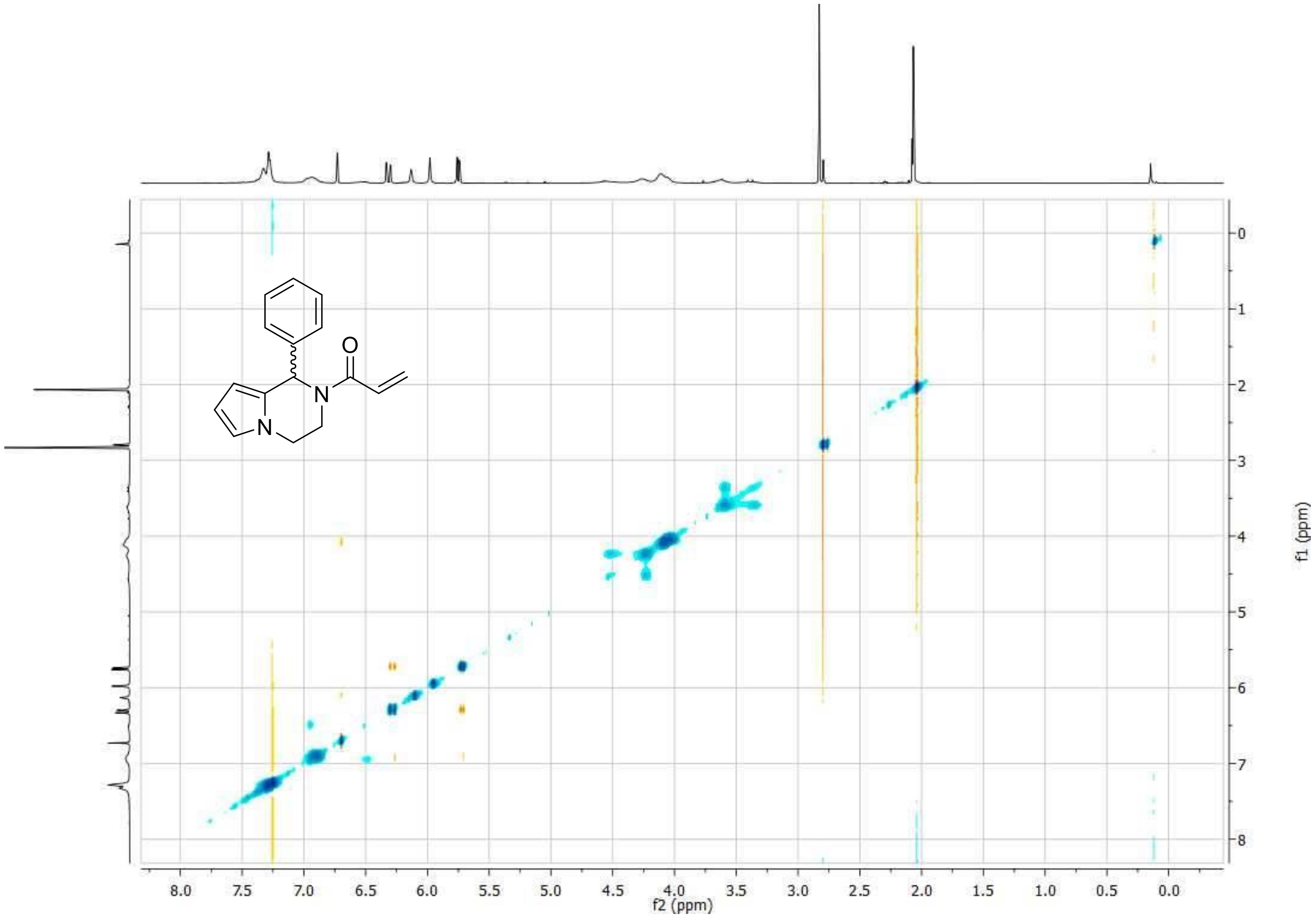

Figure S123. 2D-NOESY (500 MHz, $(CD_3)_2CO$, 298K) of **46**.

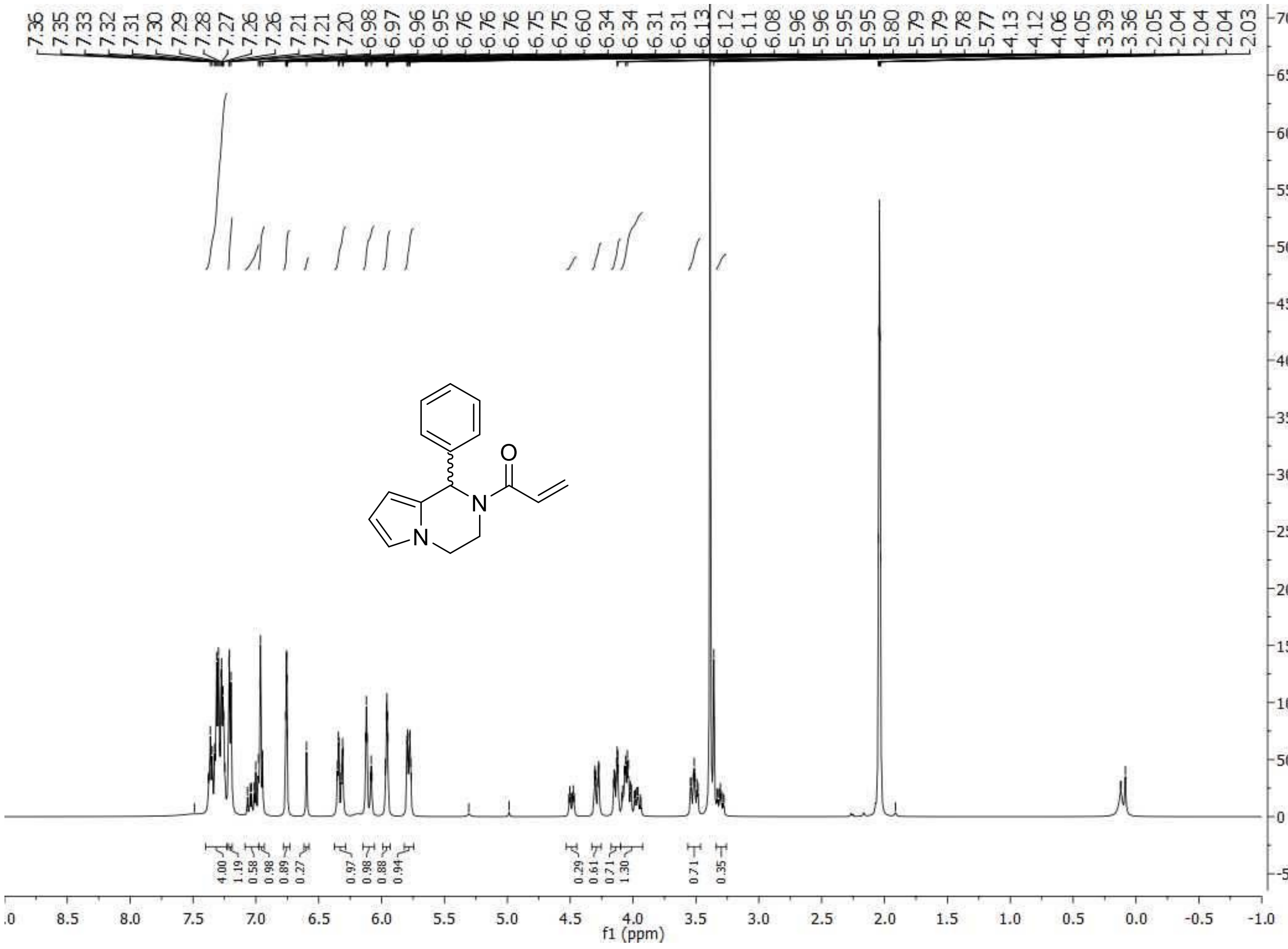

Figure S124. ^{1}H NMR (500 MHz, $(CD_3)_2CO$, 218K) of **46**.

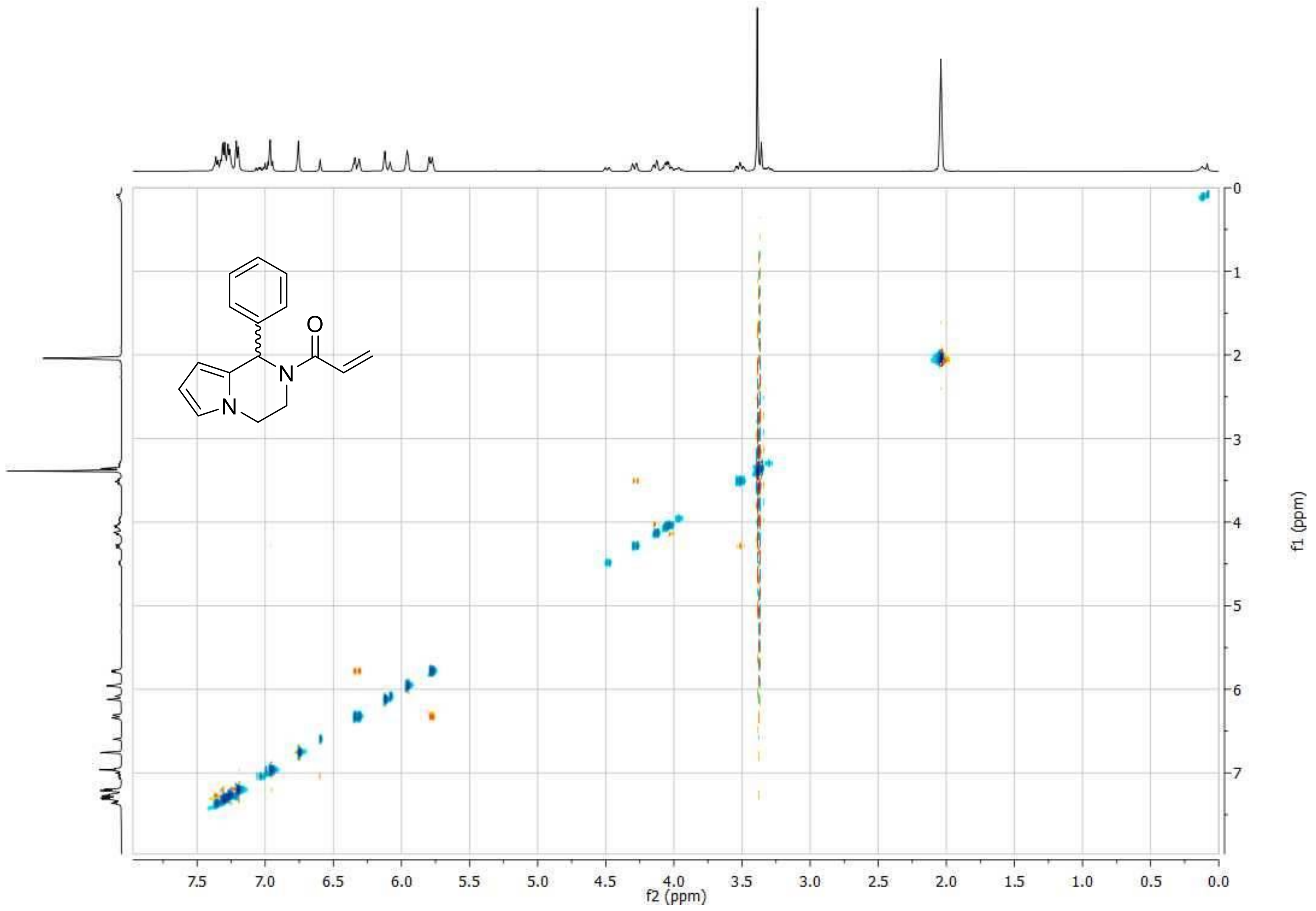

Figure S125. 2D-NOESY (500 MHz, $(CD_3)_2CO$, 218K) of **46**.

1-(2-phenylpiperidin-1-yl)ethan-1-one (47)

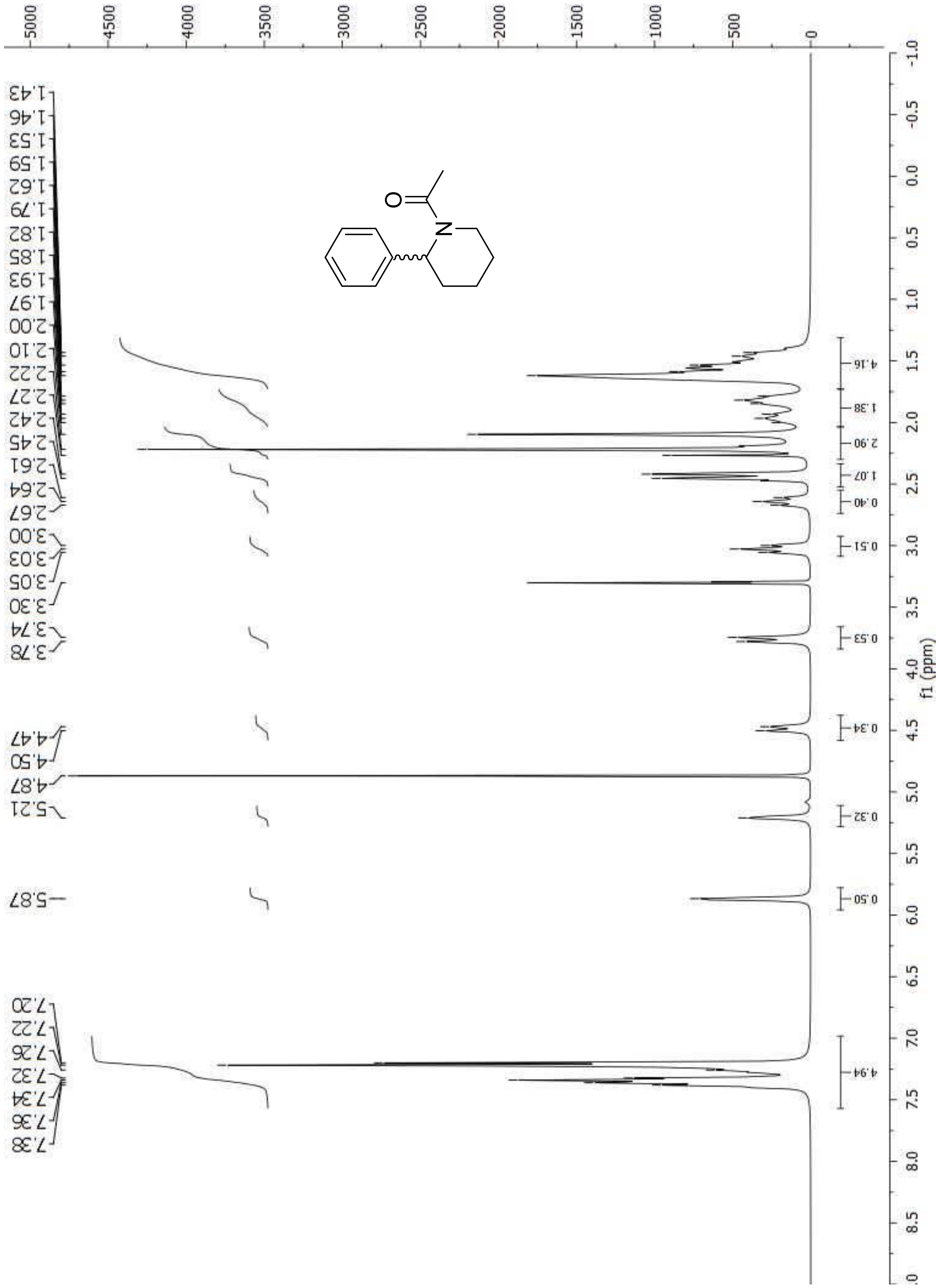

Figure S126. ^{1}H NMR (400 MHz, CD_3OD, 298K) of **47**.

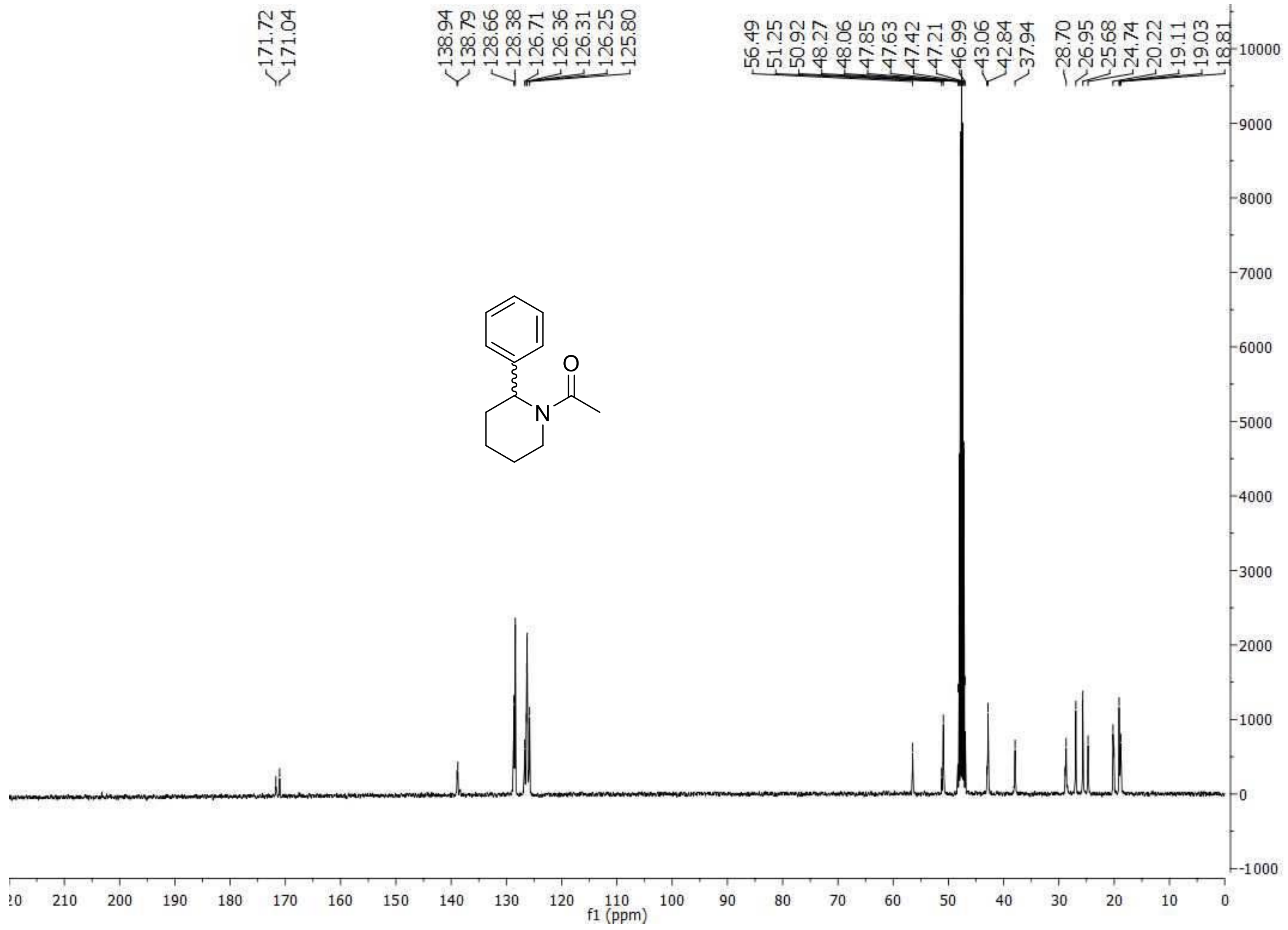

Figure S127. ^{13}C NMR (400 MHz, CD_3OD, 298K) of **47**.

1-(2-phenylpiperidin-1-yl)prop-2-en-1-one (48)

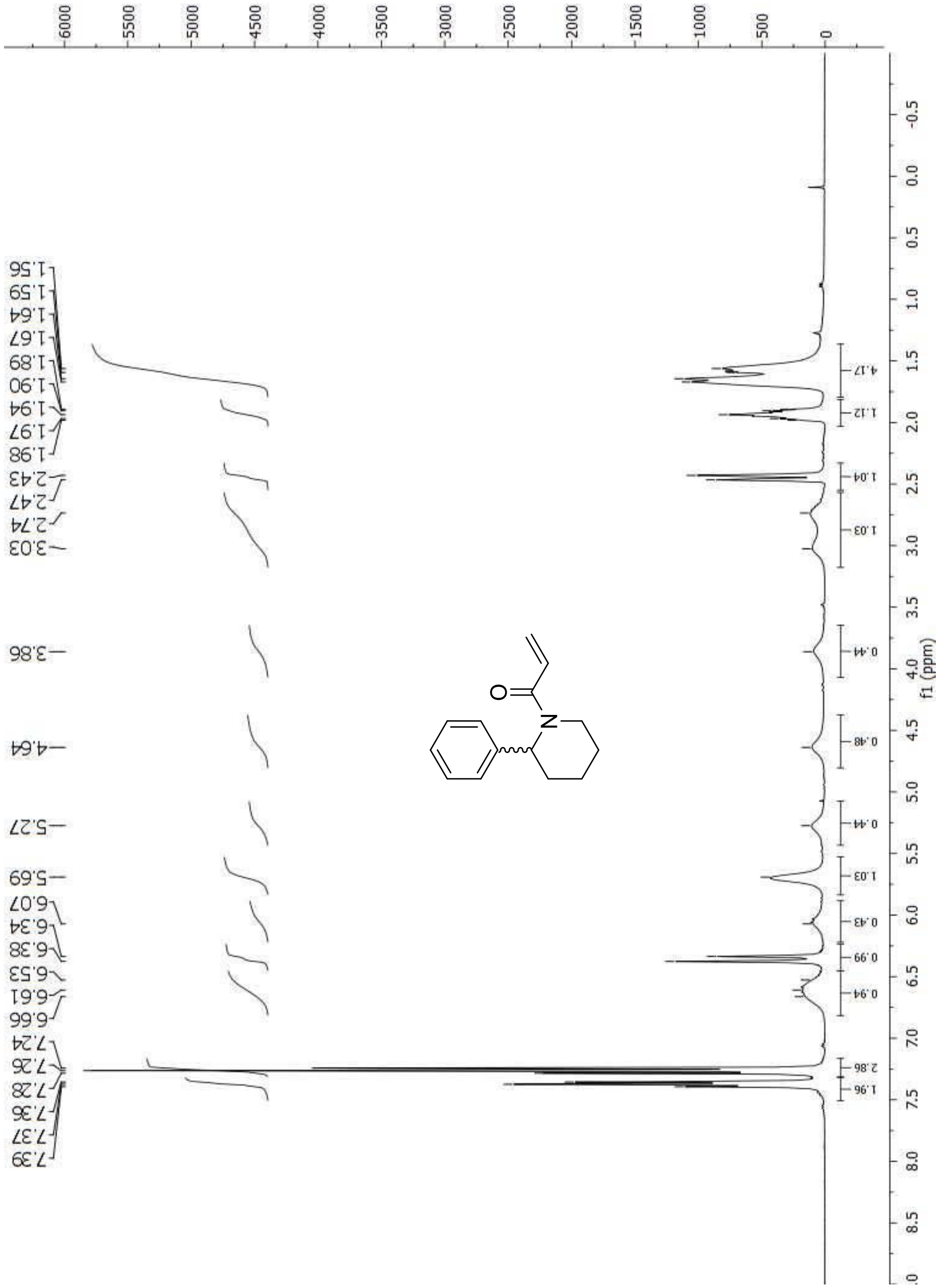

Figure S128. ^{1}H NMR (400 MHz, $CDCl_3$, 298K) of **48**.

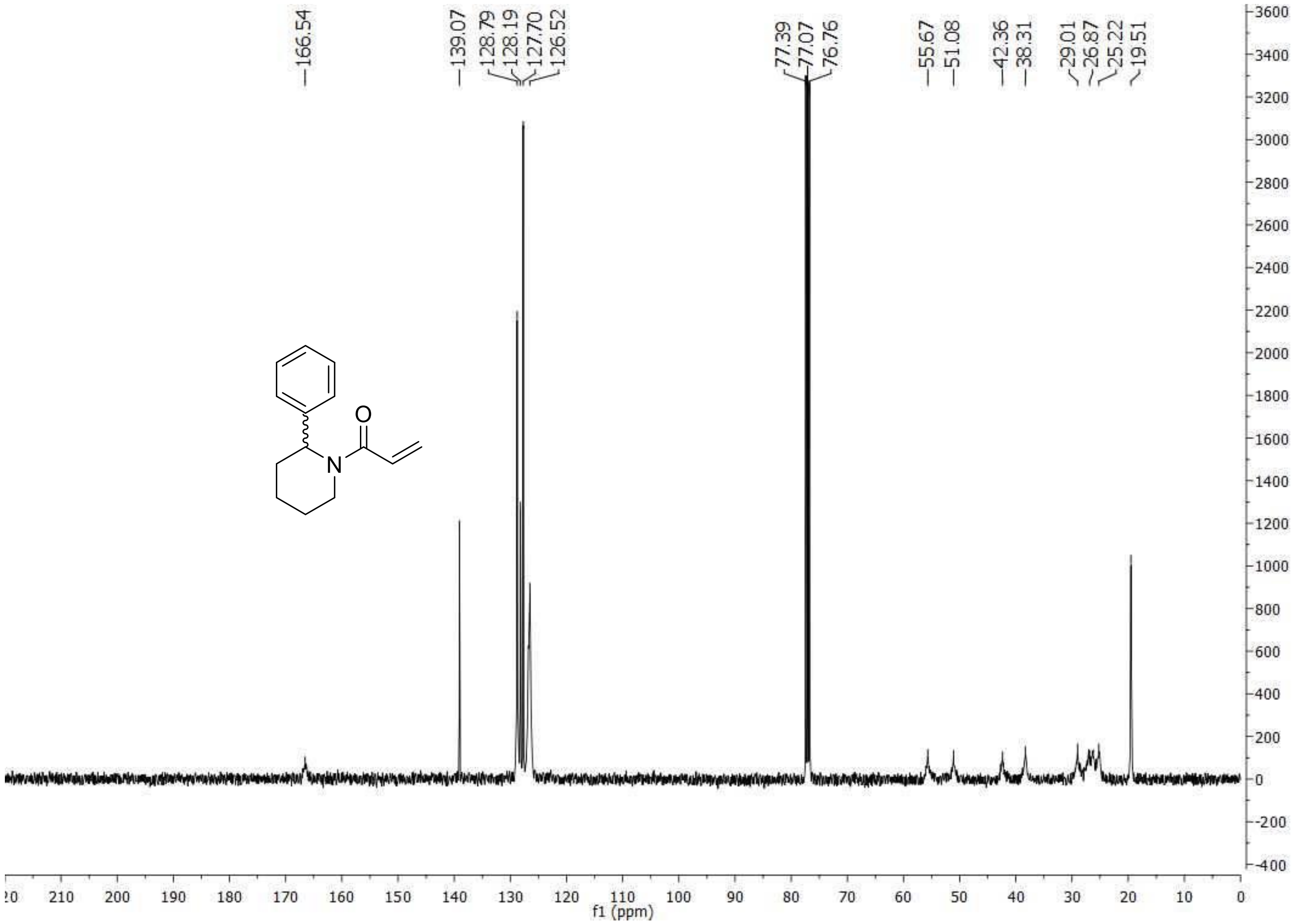

Figure S129. ^{13}C NMR (400 MHz, $CDCl_3$, 298K) of **48**.

1-(2-(pyridin-2-yl)piperidin-1-yl)prop-2-en-1-one (49)

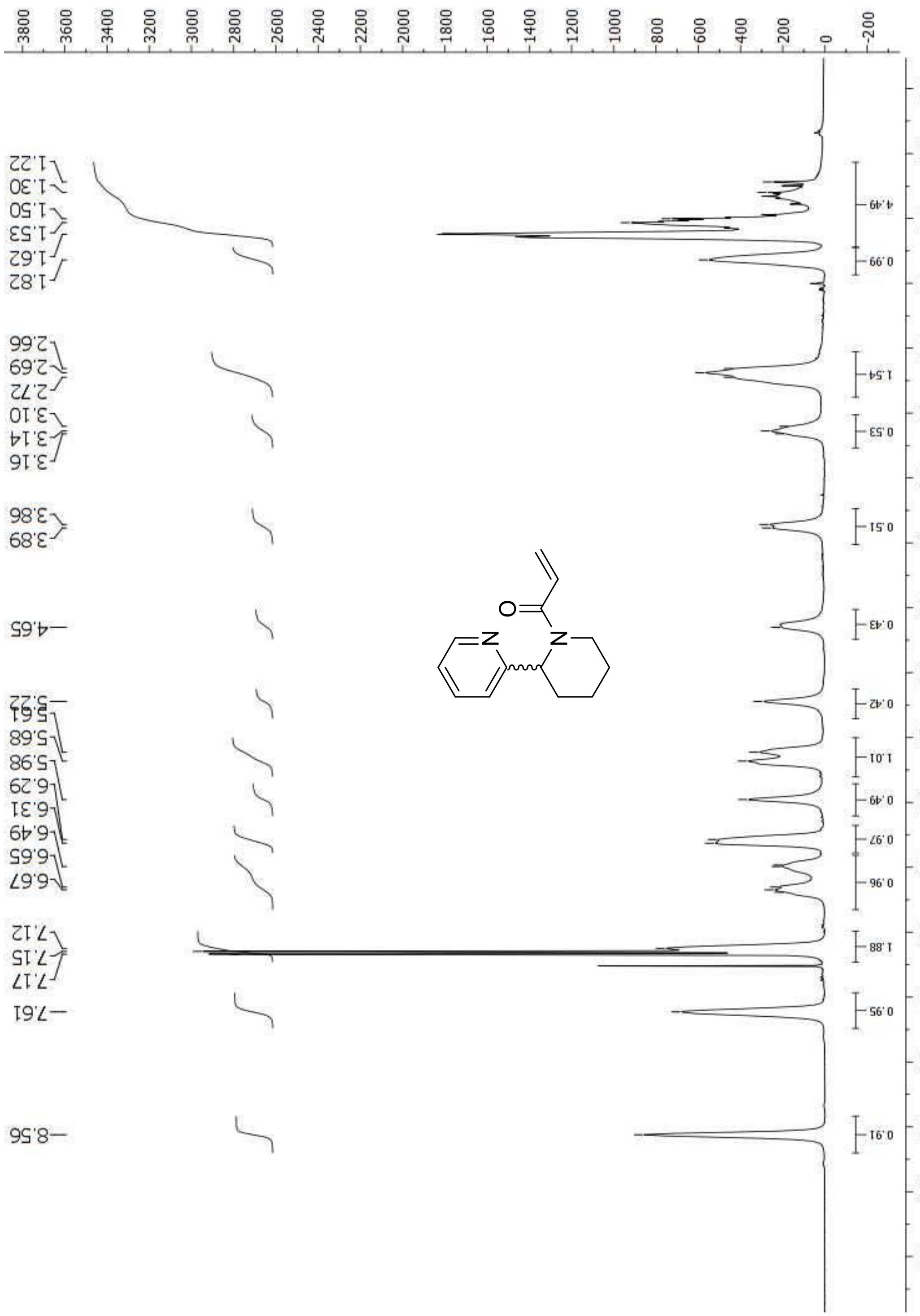

Figure S130. ^{1}H NMR (400 MHz, $CDCl_3$, 298K) of **49**.

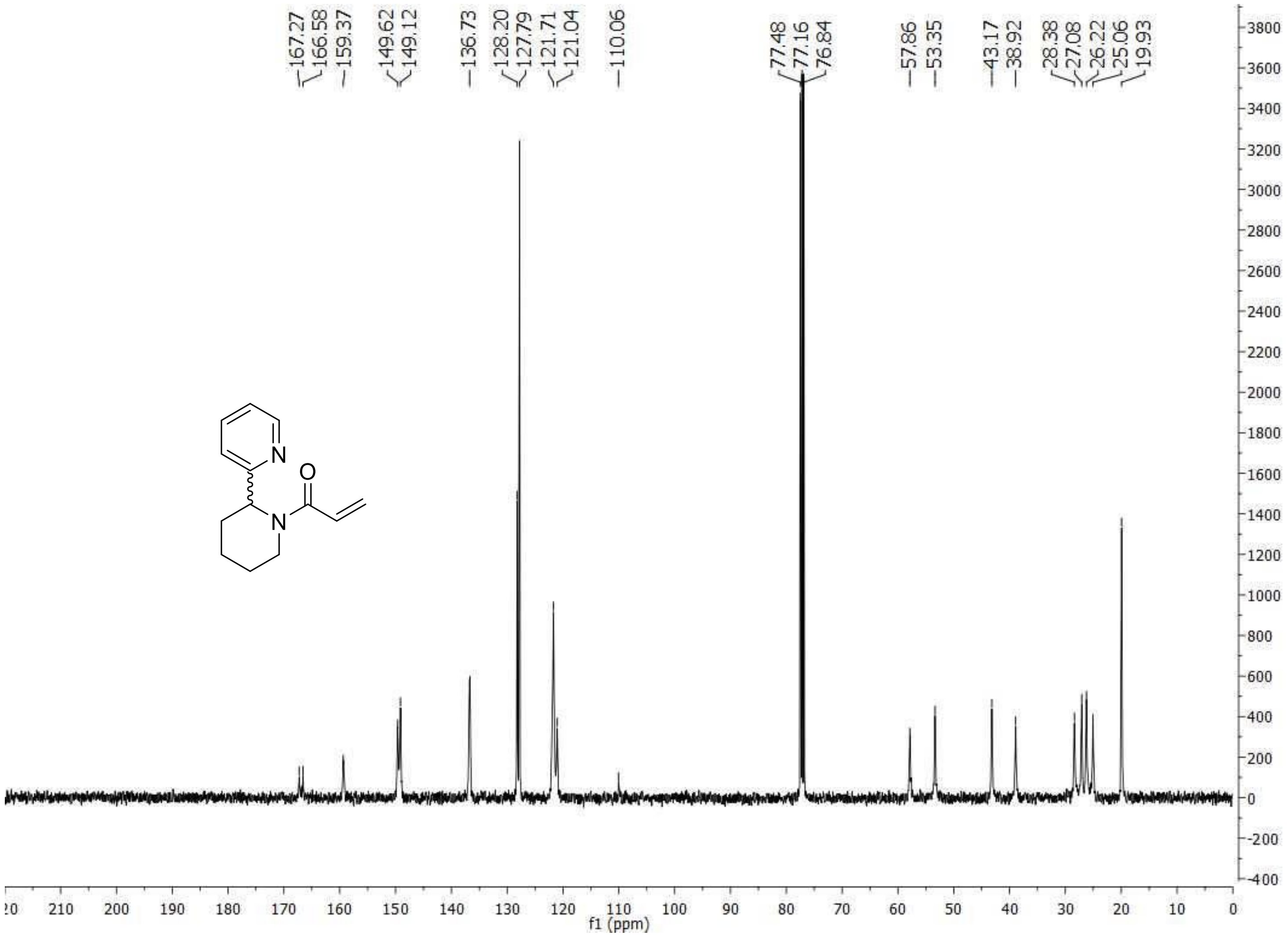

Figure S131. ^{13}C NMR (400 MHz, $CDCl_3$, 298K) of **49**.

2-((2-methylbutyl)amino)-1-(2-(pyridin-2-yl)piperidin-1-yl)ethan-1-one (50)

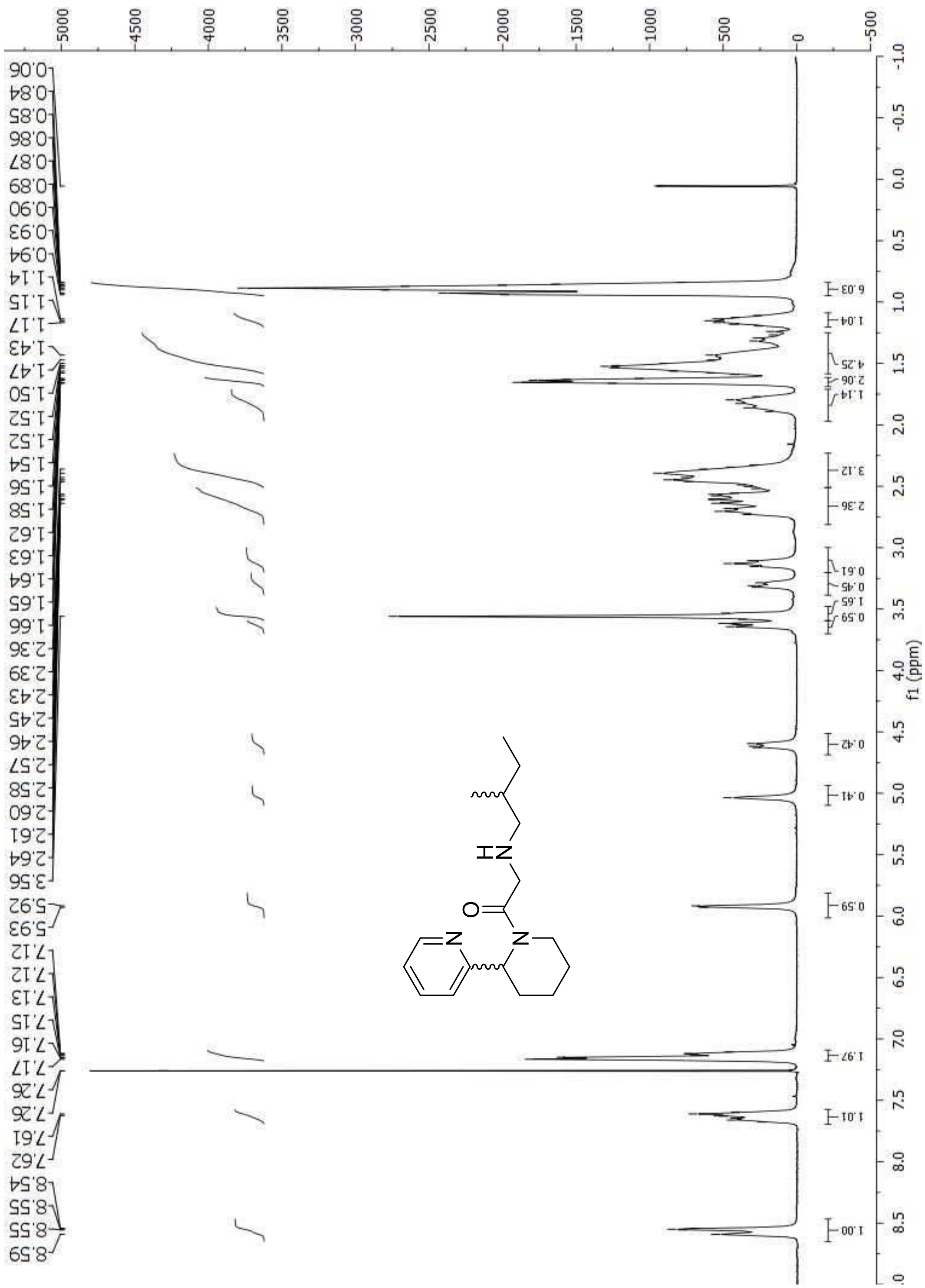

Figure S132. ^{1}H NMR (500 MHz, $CDCl_3$, 298K) of **50**.

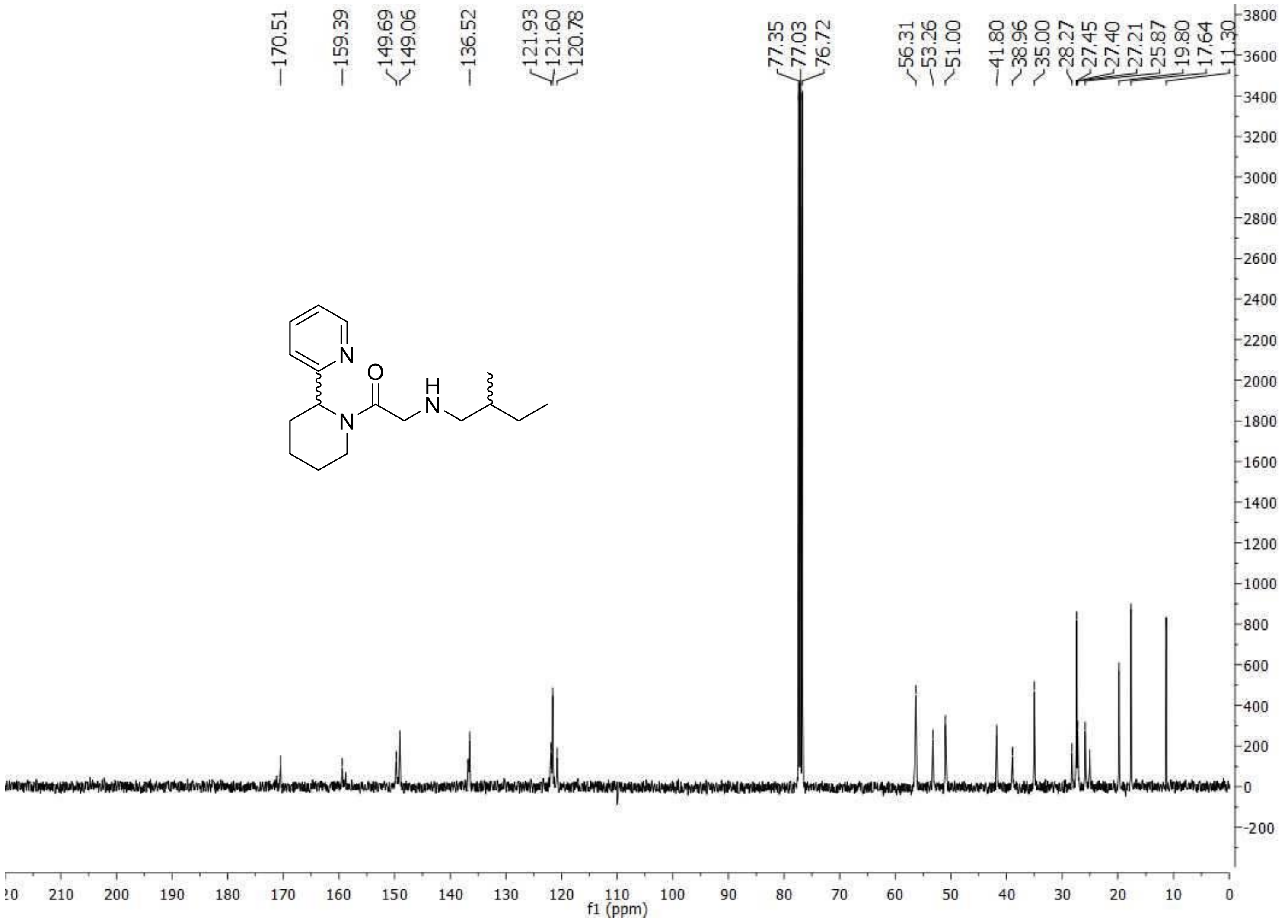

Figure S133. ^{13}C NMR (500 MHz, $CDCl_3$) of **50**.

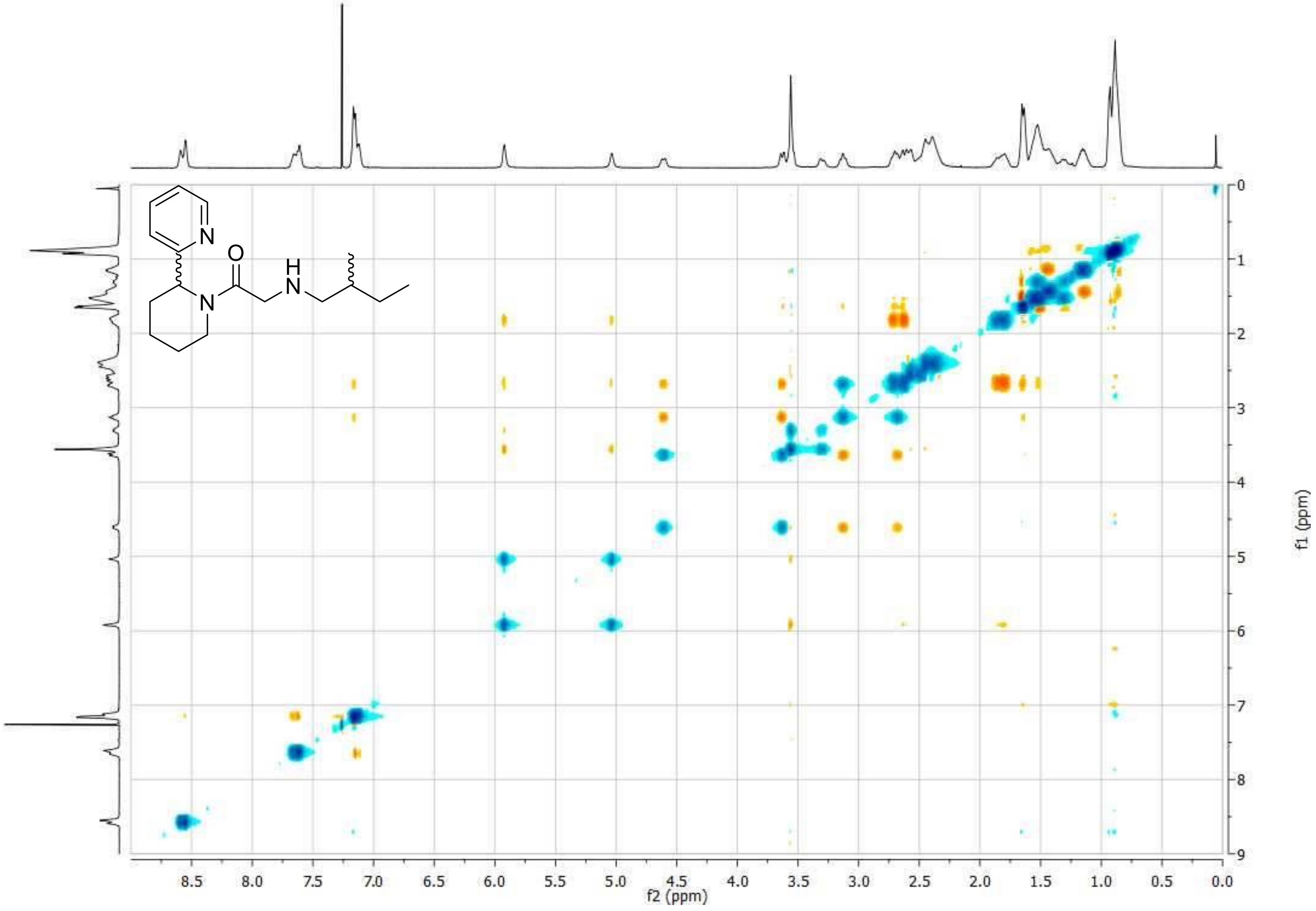

Figure S134.2D-NOESY (500 MHz, $CDCl_3$, 298K) of **50**.

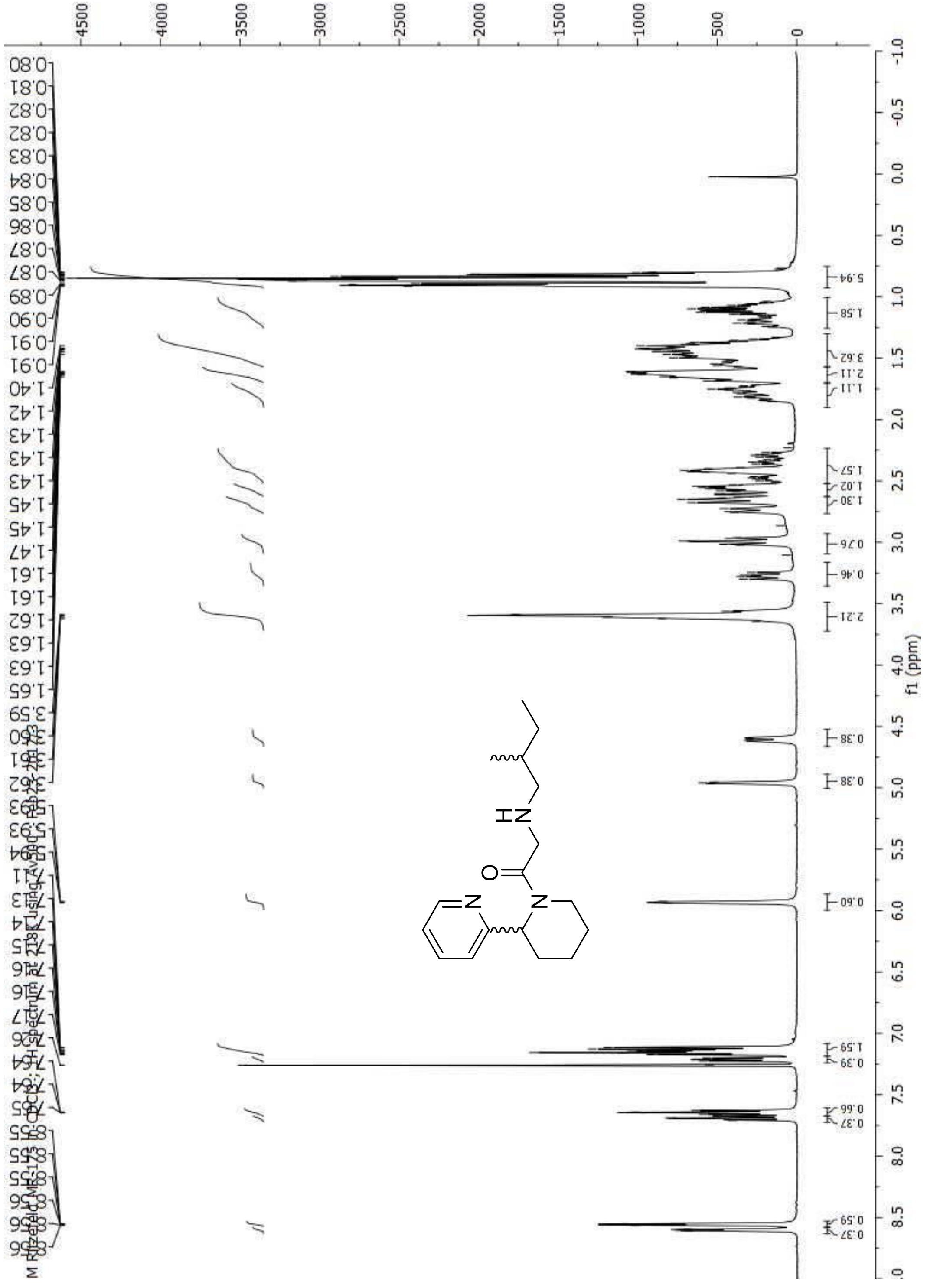

Figure S135. ^{1}H NMR (500 MHz, $CDCl_3$, 218K) of **50**.

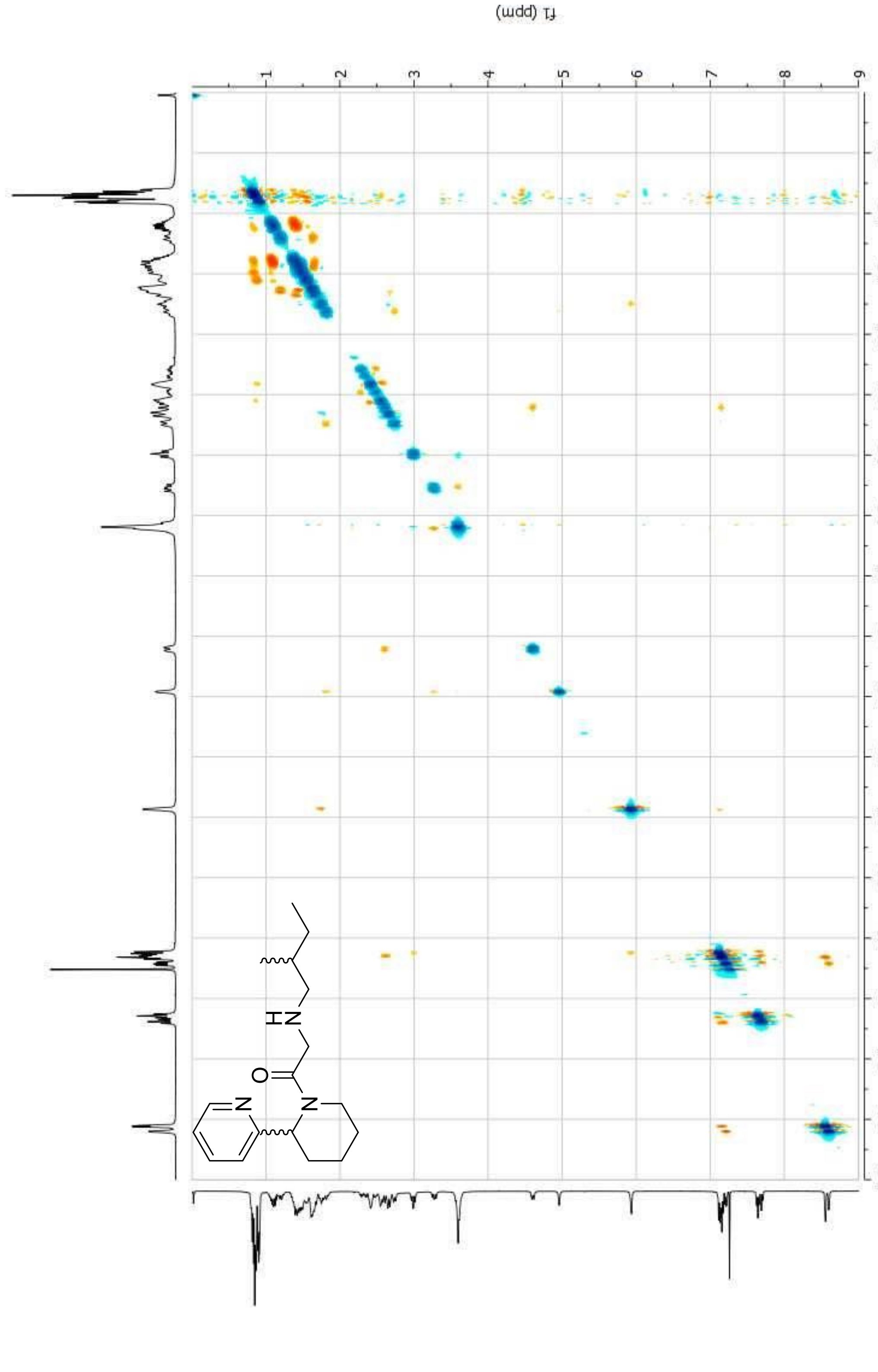

Figure S136.2D-NOESY (500 MHz, $CDCl_3$, 218K) of **50**.

12. References

(1) Lanyon-Hogg, T.; Masumoto, N.; Bodakh, G.; Konitsiotis, A. D.; Thinon, E.; Rodgers, U. R.; Owens, R. J.; Magee, A. I.; Tate, E. W. *Data Brief* **2016**, *7*, 257.

(2) Lanyon-Hogg, T.; Ritzefeld, M.; Masumoto, N.; Magee, A. I.; Rzepa, H. S.; Tate, E. W. *J. Org. Chem.* **2015**, *80* (9), 4370.

(3) Lanyon-Hogg, T.; Masumoto, N.; Bodakh, G.; Konitsiotis, A. D.; Thinon, E.; Rodgers, U. R.; Owens, R. J.; Magee, A. I.; Tate, E. W. *Anal. Biochem.* **2015**, *490*, 66.

(4) Broncel, M.; Serwa, R. A.; Ciepla, P.; Krause, E.; Dallman, M. J.; Magee, A. I.; Tate, E. W. *Angew. Chem. Int. Ed.* **2015**, *54* (20), 5948.

(5) Rodgers, U. R.; Lanyon-Hogg, T.; Masumoto, N.; Ritzefeld, M.; Burke, R.; Blagg, J.; Magee, A. I.; Tate, E. W. *ACS Chem. Biol.* **2016**, *11* (12), 3256.

(6) Hussain, S.; Leipold, F.; Man, H.; Wells, E.; France, S. P.; Mulholland, K. R.; Grogan, G.; Turner, N. J. *ChemCatChem* **2015**, *7* (4), 579.

(7) Heath, R. S.; Pontini, M.; Hussain, S.; Turner, N. J. *ChemCatChem* **2016**, *8* (1), 117.
